# Supplementary figures and images for: Interactive machine learning for fast and robust cell profiling (part 2 of 2)
Source: PLoS One. 2020 Sep 11;15(9):e0237972. doi: 10.1371/journal.pone.0237972 (PMC7485821; doi:10.1371/journal.pone.0237972)

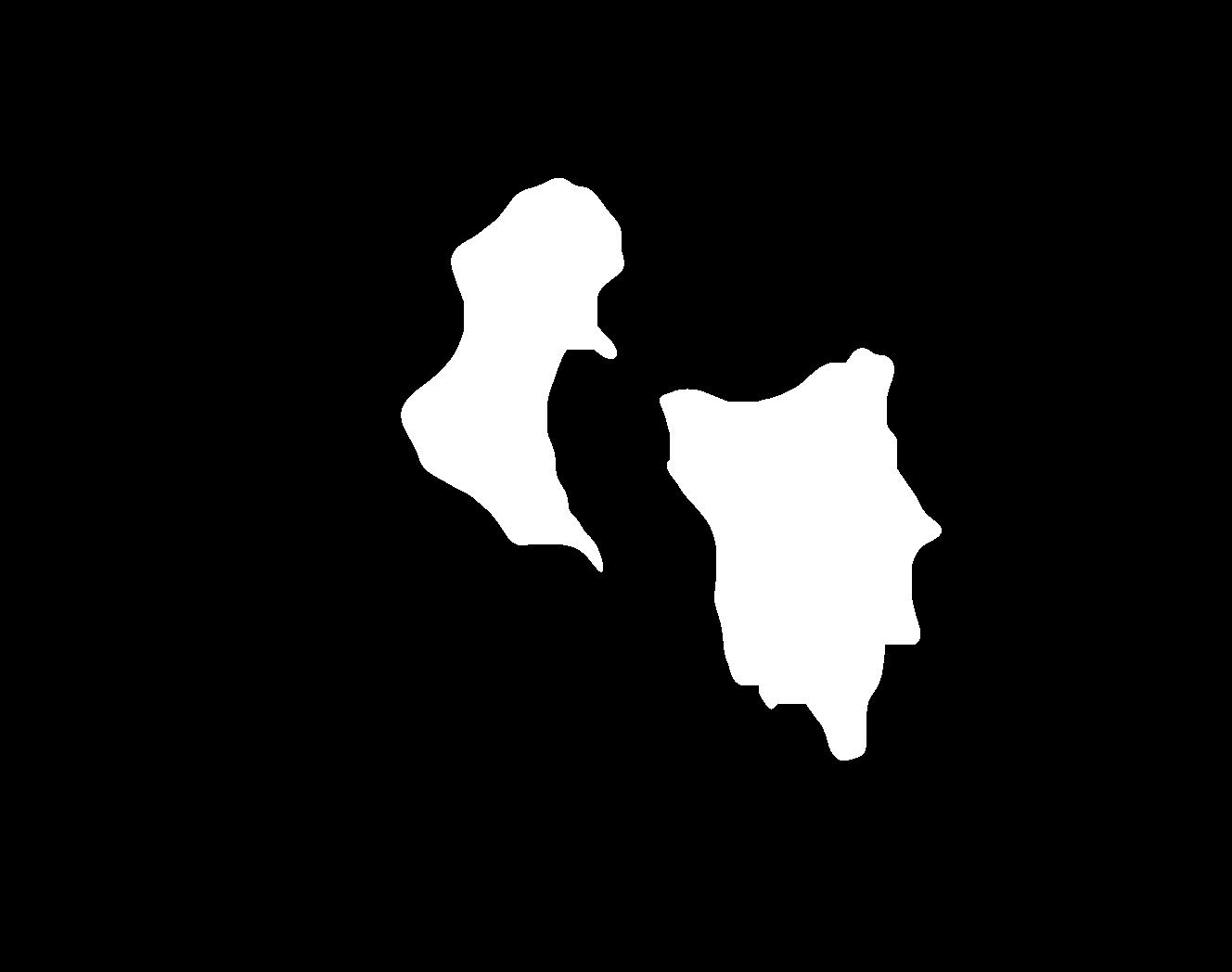

Supplement: S5 File — (ZIP) [file pone.0237972.s005.zip › S3_File IoU scores/masks/Experiment_1/cell/user_segmented/Composite/Composite_Participant15_mask_cell_I.jpg]

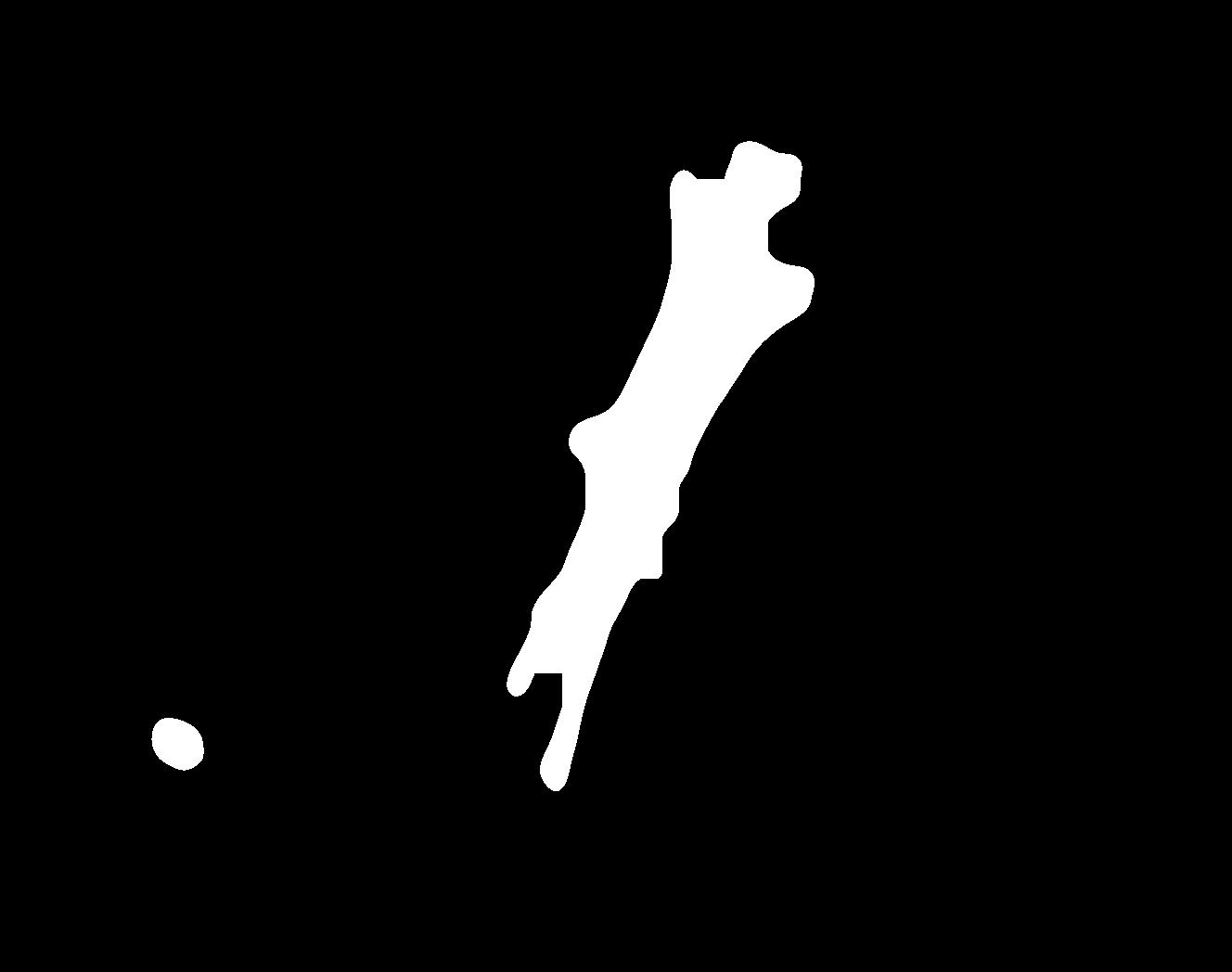

Supplement: S5 File — (ZIP) [file pone.0237972.s005.zip › S3_File IoU scores/masks/Experiment_1/cell/user_segmented/Composite/Composite_Participant15_mask_cell_J.jpg]

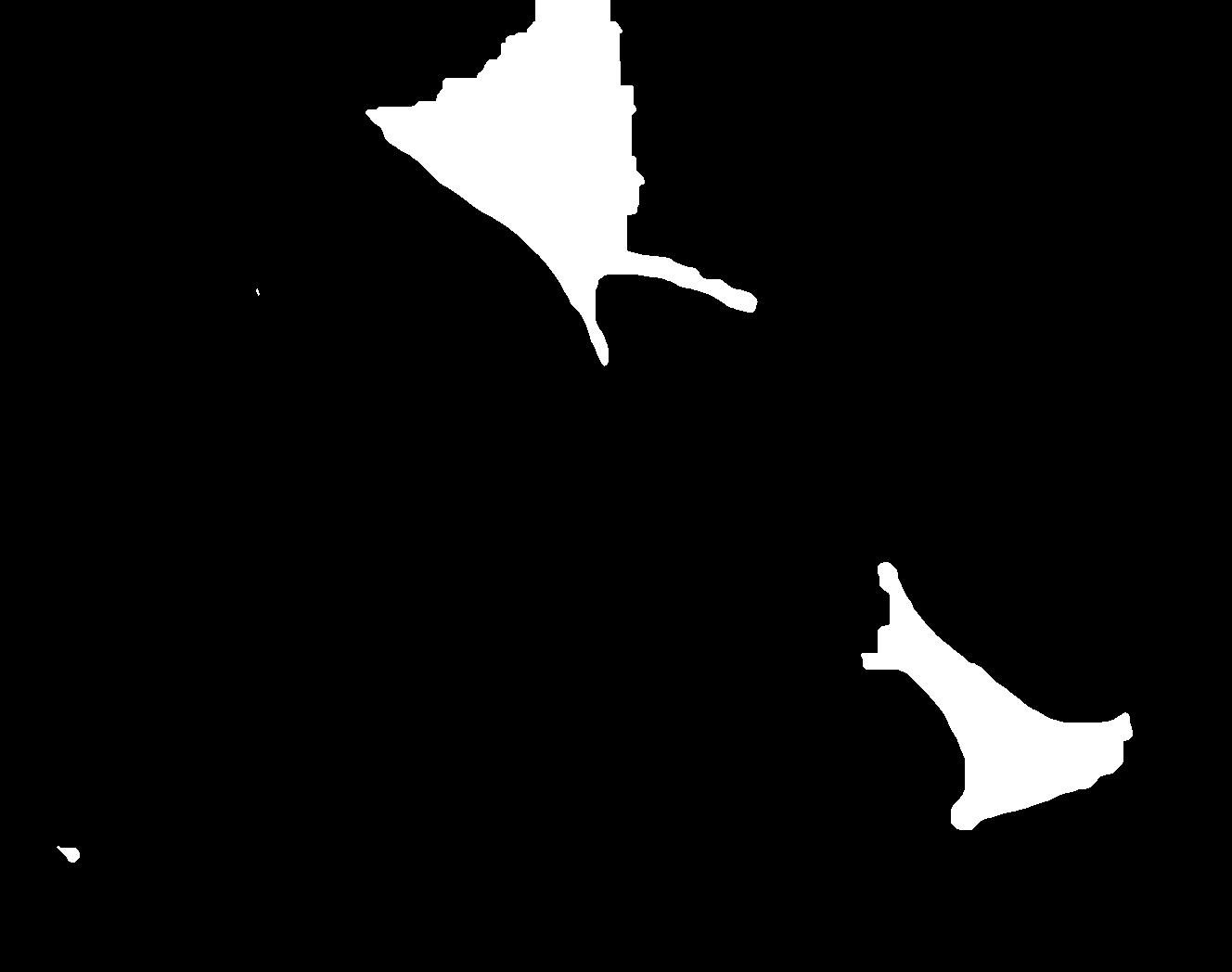

Supplement: S5 File — (ZIP) [file pone.0237972.s005.zip › S3_File IoU scores/masks/Experiment_1/cell/user_segmented/Composite/Composite_Participant6_mask_cell_A.jpg]

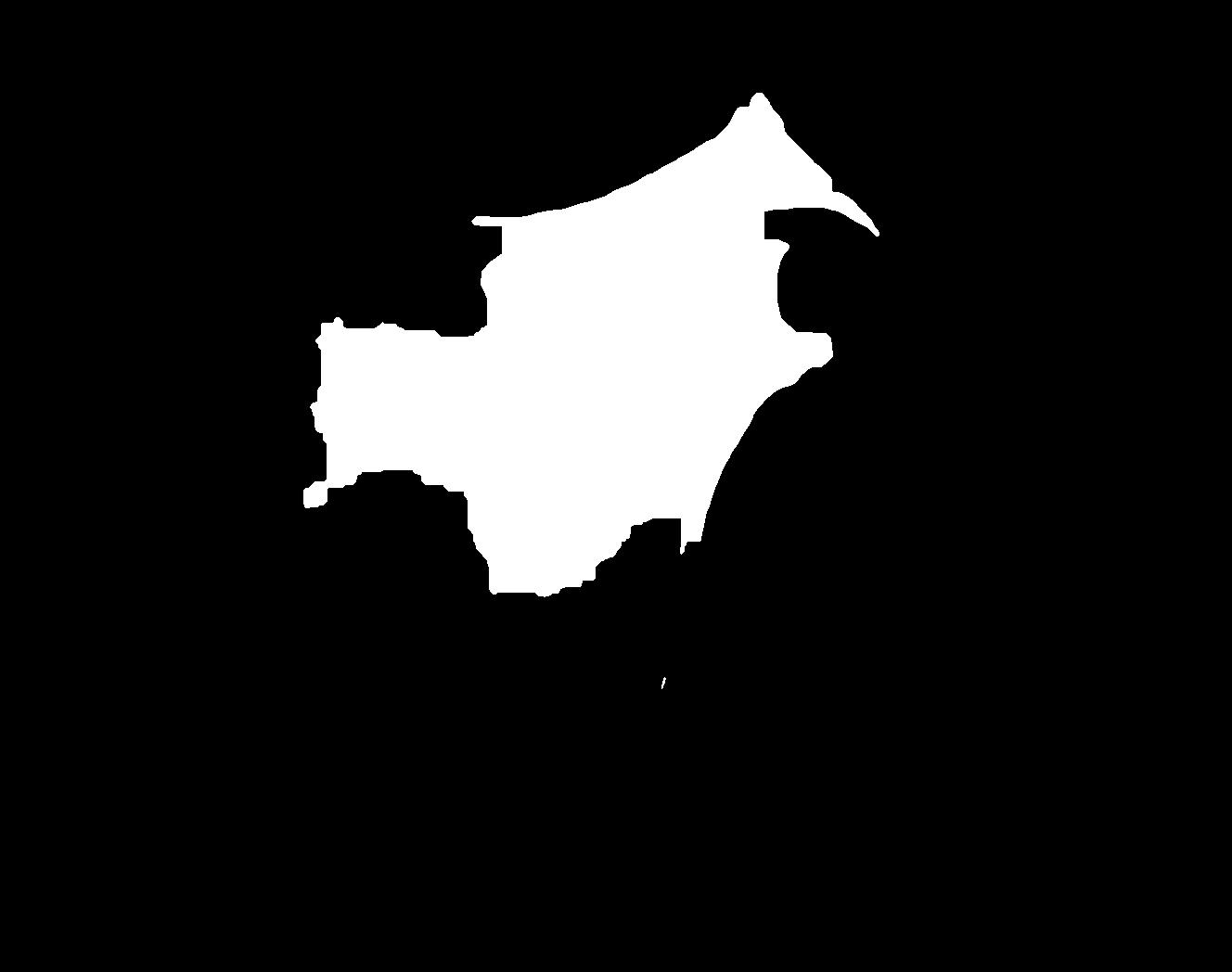

Supplement: S5 File — (ZIP) [file pone.0237972.s005.zip › S3_File IoU scores/masks/Experiment_1/cell/user_segmented/Composite/Composite_Participant6_mask_cell_E.jpg]

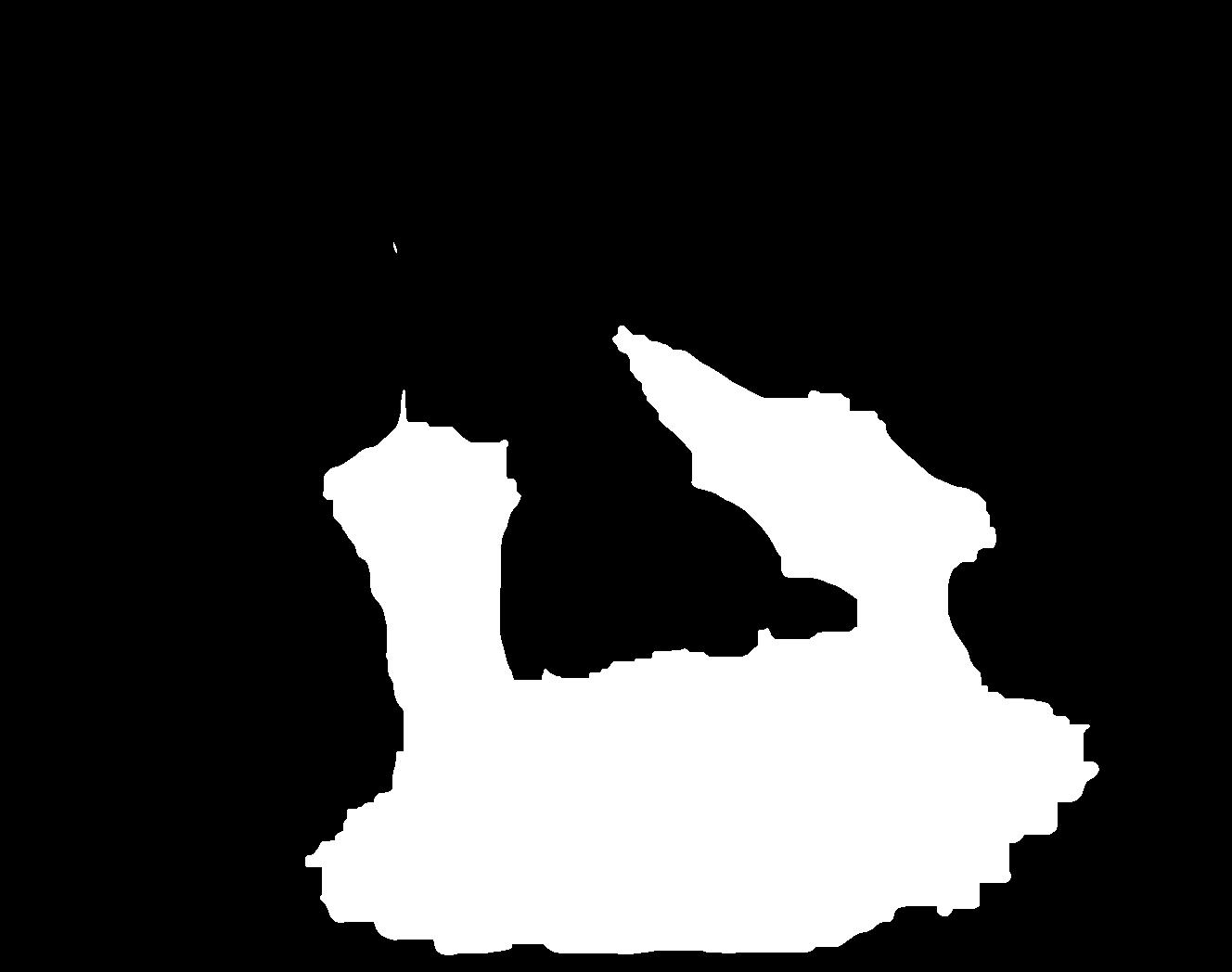

Supplement: S5 File — (ZIP) [file pone.0237972.s005.zip › S3_File IoU scores/masks/Experiment_1/cell/user_segmented/Composite/Composite_Participant6_mask_cell_F.jpg]

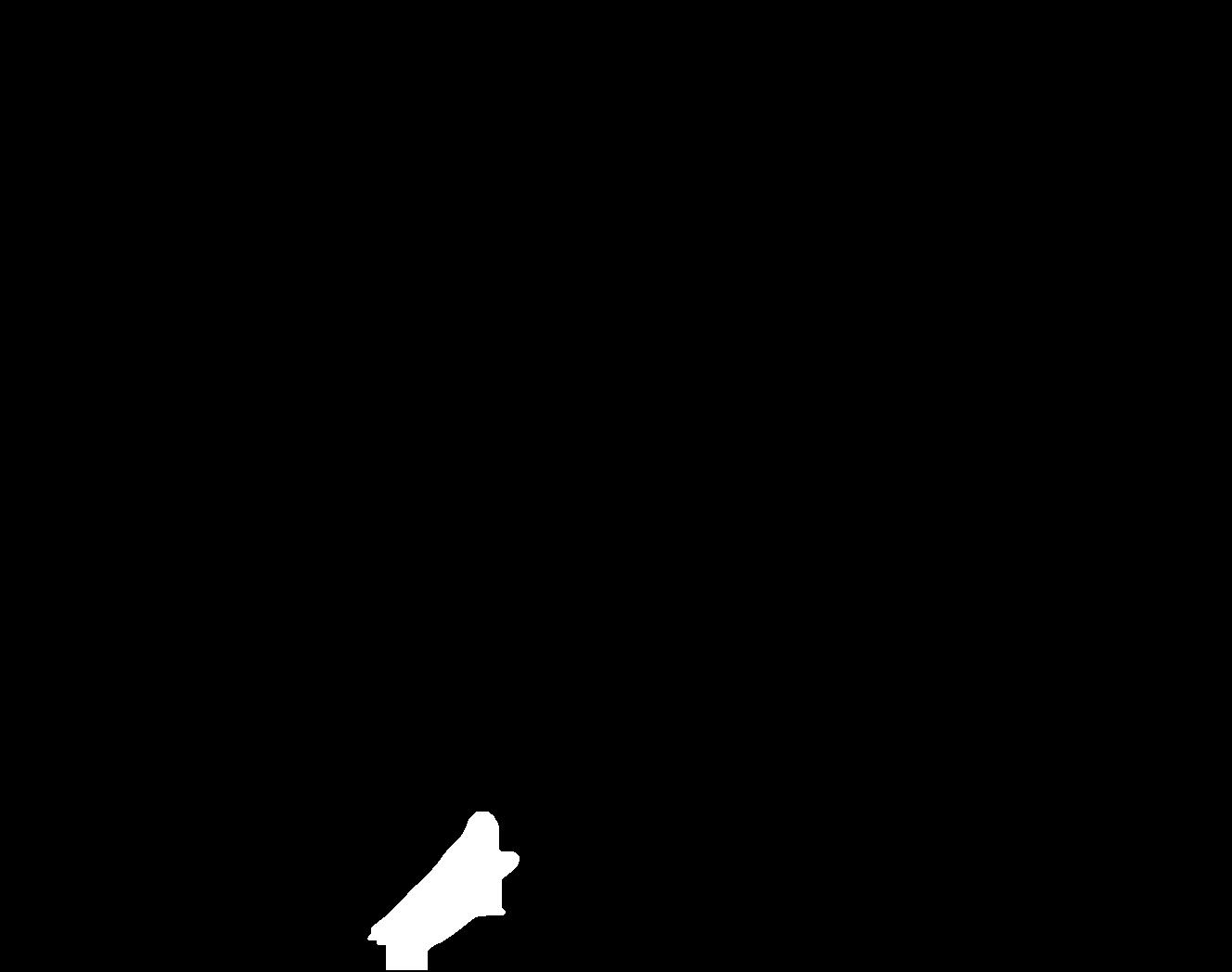

Supplement: S5 File — (ZIP) [file pone.0237972.s005.zip › S3_File IoU scores/masks/Experiment_1/cell/user_segmented/Composite/Composite_Participant6_mask_cell_G.jpg]

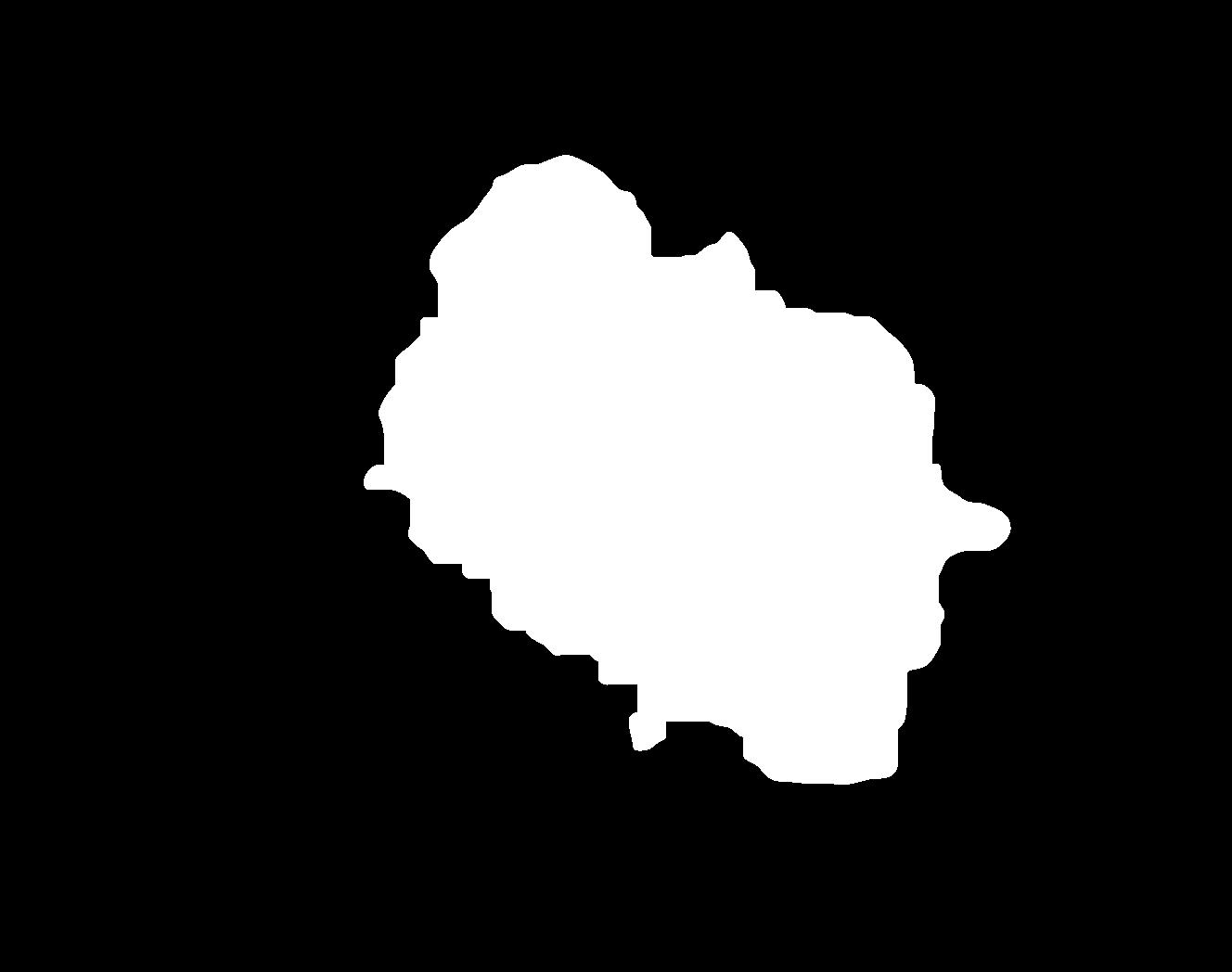

Supplement: S5 File — (ZIP) [file pone.0237972.s005.zip › S3_File IoU scores/masks/Experiment_1/cell/user_segmented/Composite/Composite_Participant6_mask_cell_I.jpg]

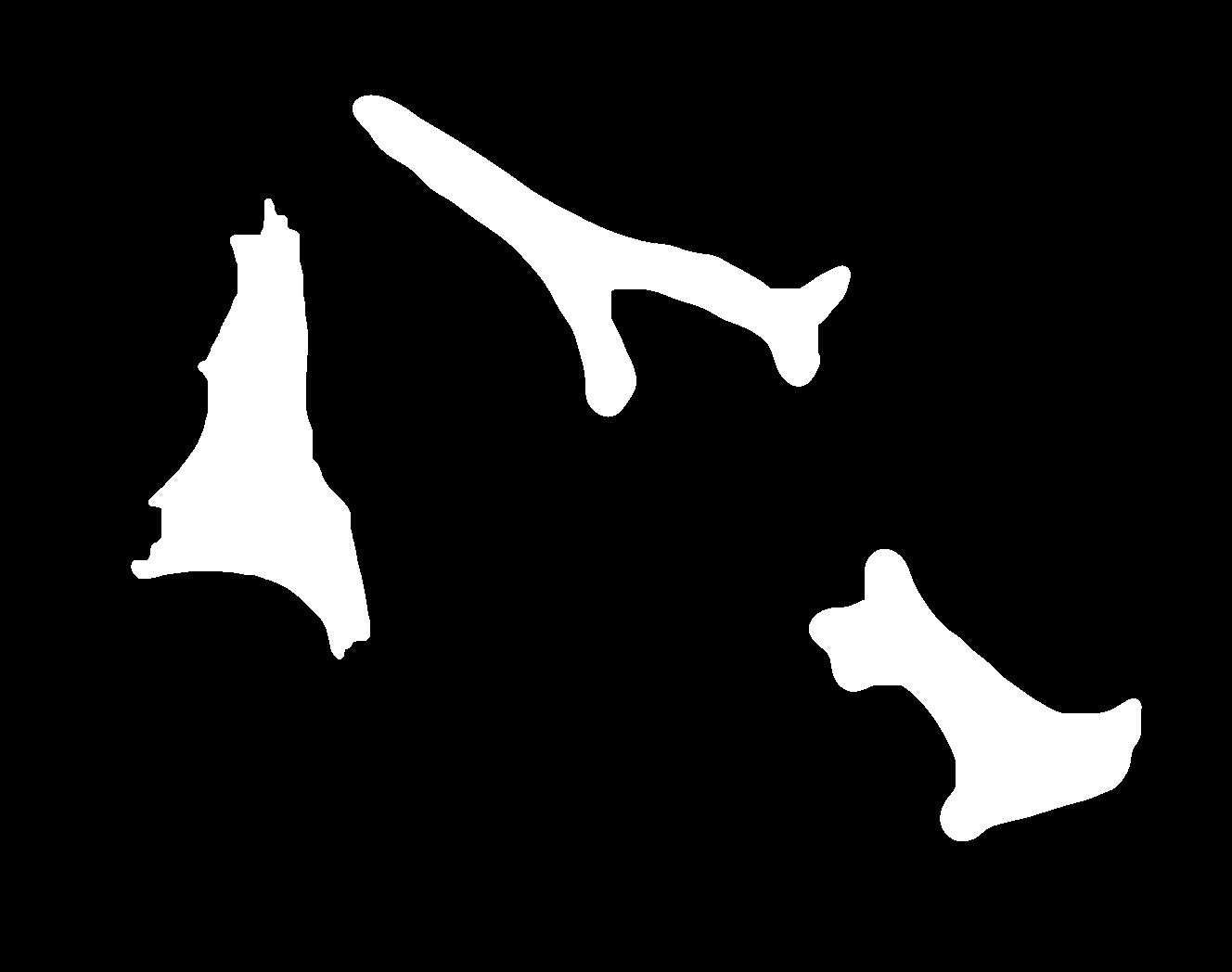

Supplement: S5 File — (ZIP) [file pone.0237972.s005.zip › S3_File IoU scores/masks/Experiment_1/cell/user_segmented/Composite/Composite_Participant9_mask_cell_A.jpg]

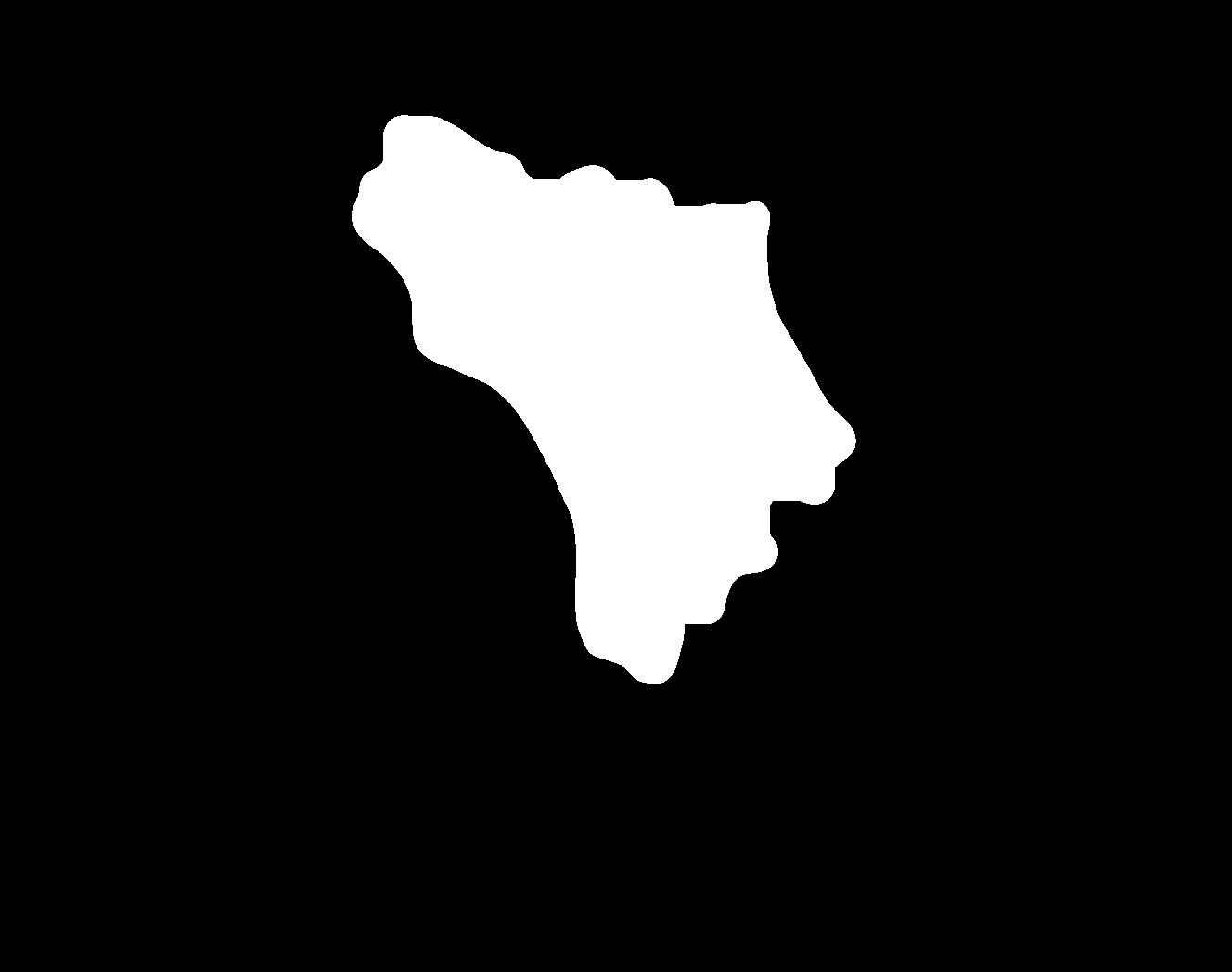

Supplement: S5 File — (ZIP) [file pone.0237972.s005.zip › S3_File IoU scores/masks/Experiment_1/cell/user_segmented/Composite/Composite_Participant9_mask_cell_B.jpg]

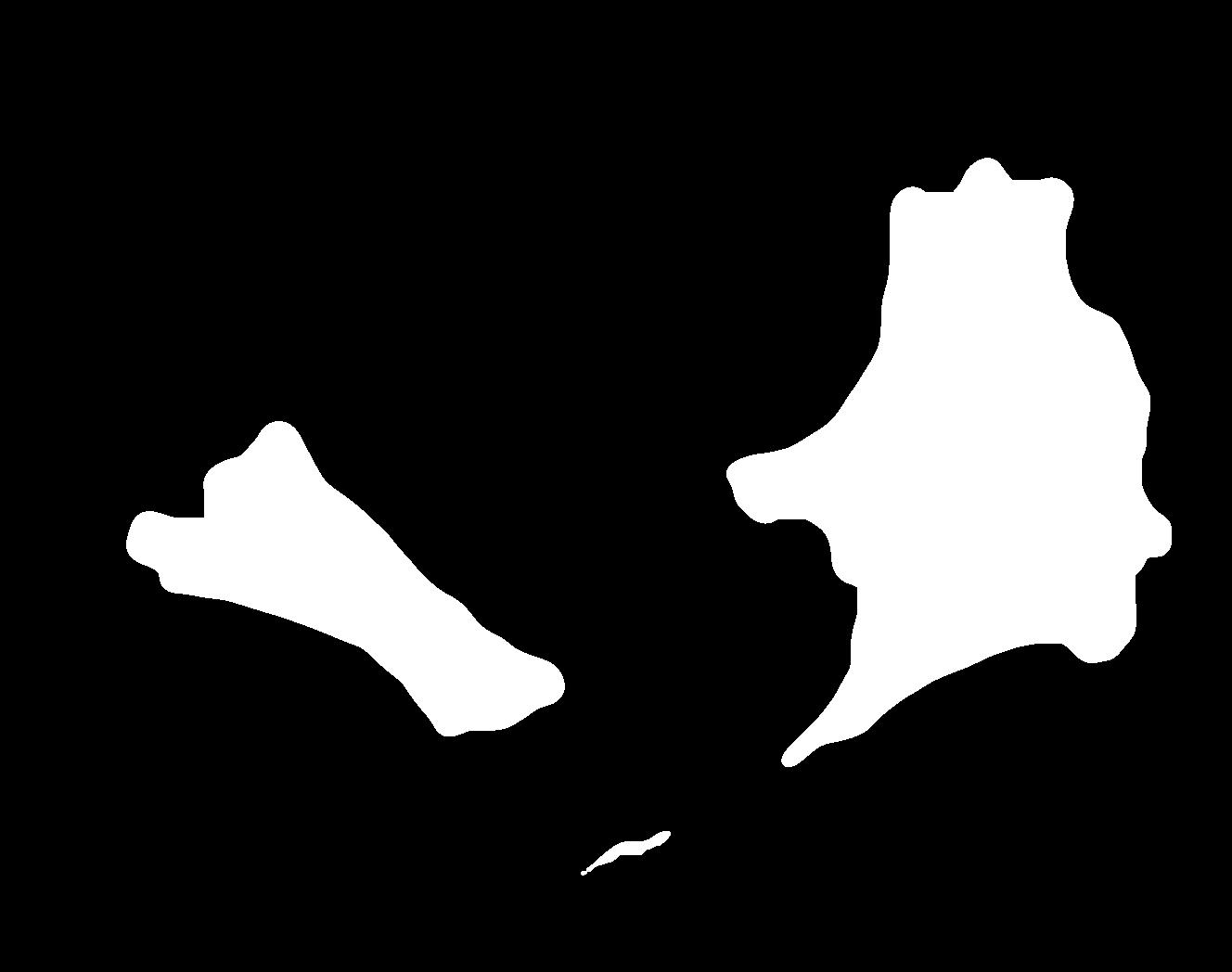

Supplement: S5 File — (ZIP) [file pone.0237972.s005.zip › S3_File IoU scores/masks/Experiment_1/cell/user_segmented/Composite/Composite_Participant9_mask_cell_C.jpg]

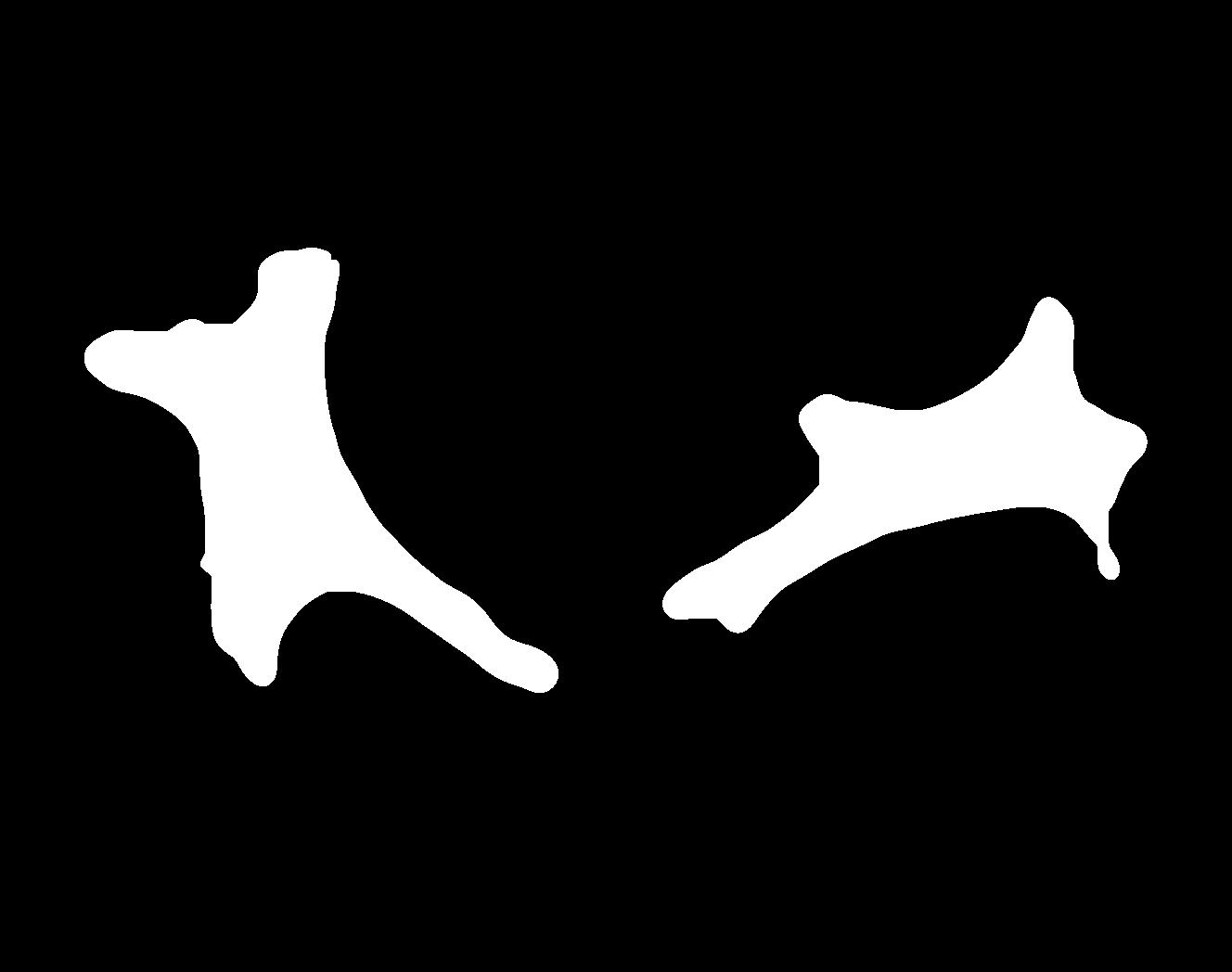

Supplement: S5 File — (ZIP) [file pone.0237972.s005.zip › S3_File IoU scores/masks/Experiment_1/cell/user_segmented/Composite/Composite_Participant9_mask_cell_D.jpg]

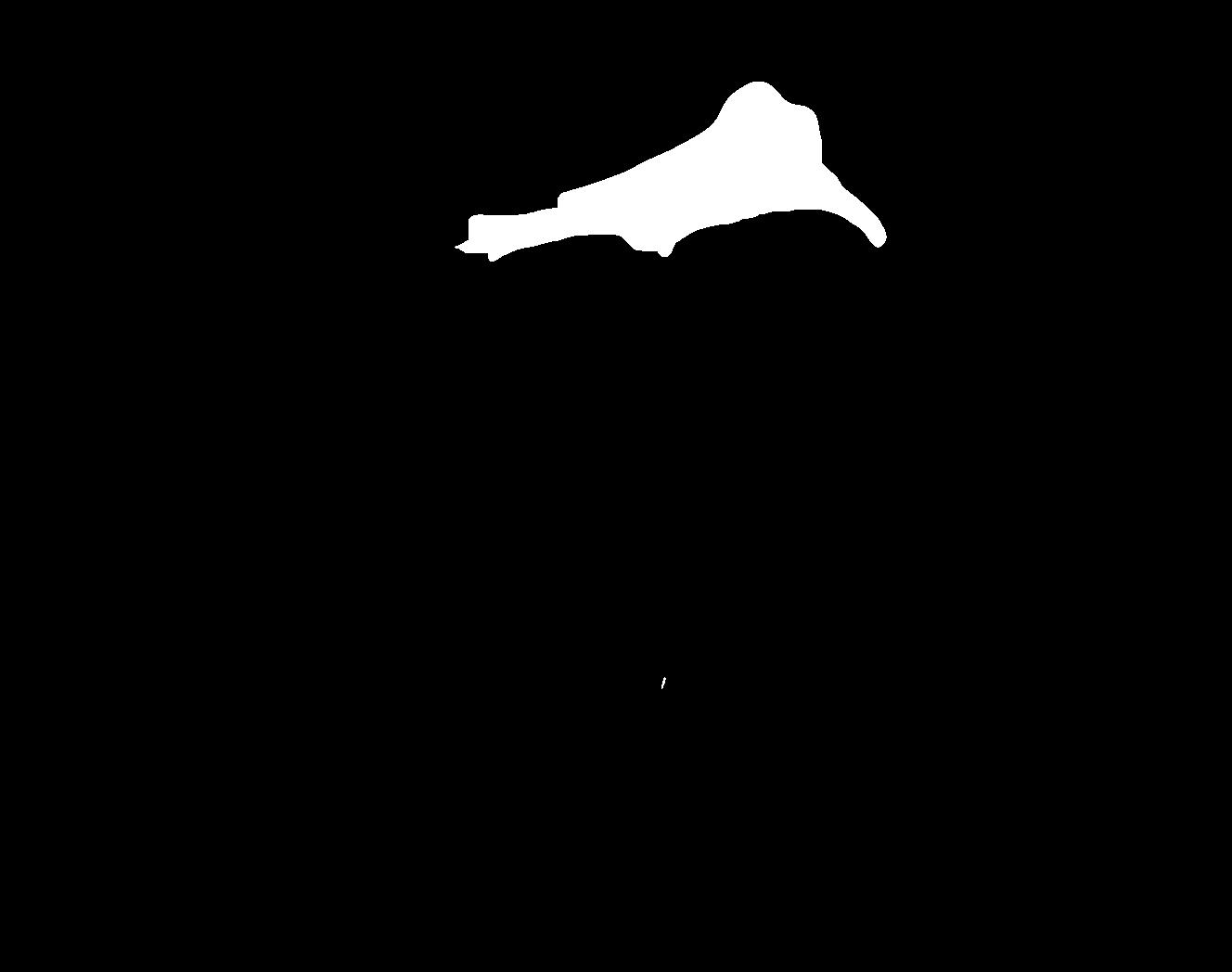

Supplement: S5 File — (ZIP) [file pone.0237972.s005.zip › S3_File IoU scores/masks/Experiment_1/cell/user_segmented/Composite/Composite_Participant9_mask_cell_E.jpg]

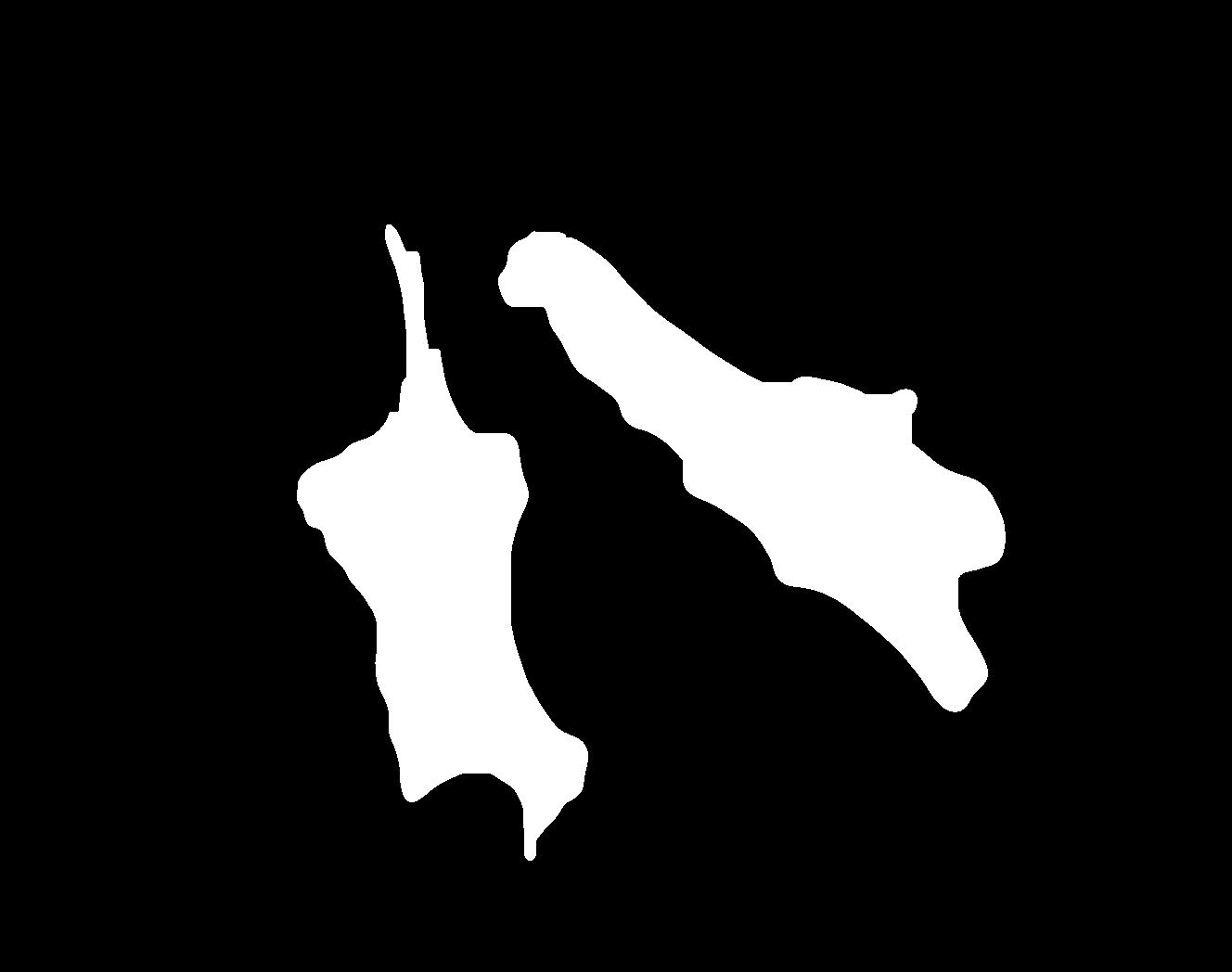

Supplement: S5 File — (ZIP) [file pone.0237972.s005.zip › S3_File IoU scores/masks/Experiment_1/cell/user_segmented/Composite/Composite_Participant9_mask_cell_F.jpg]

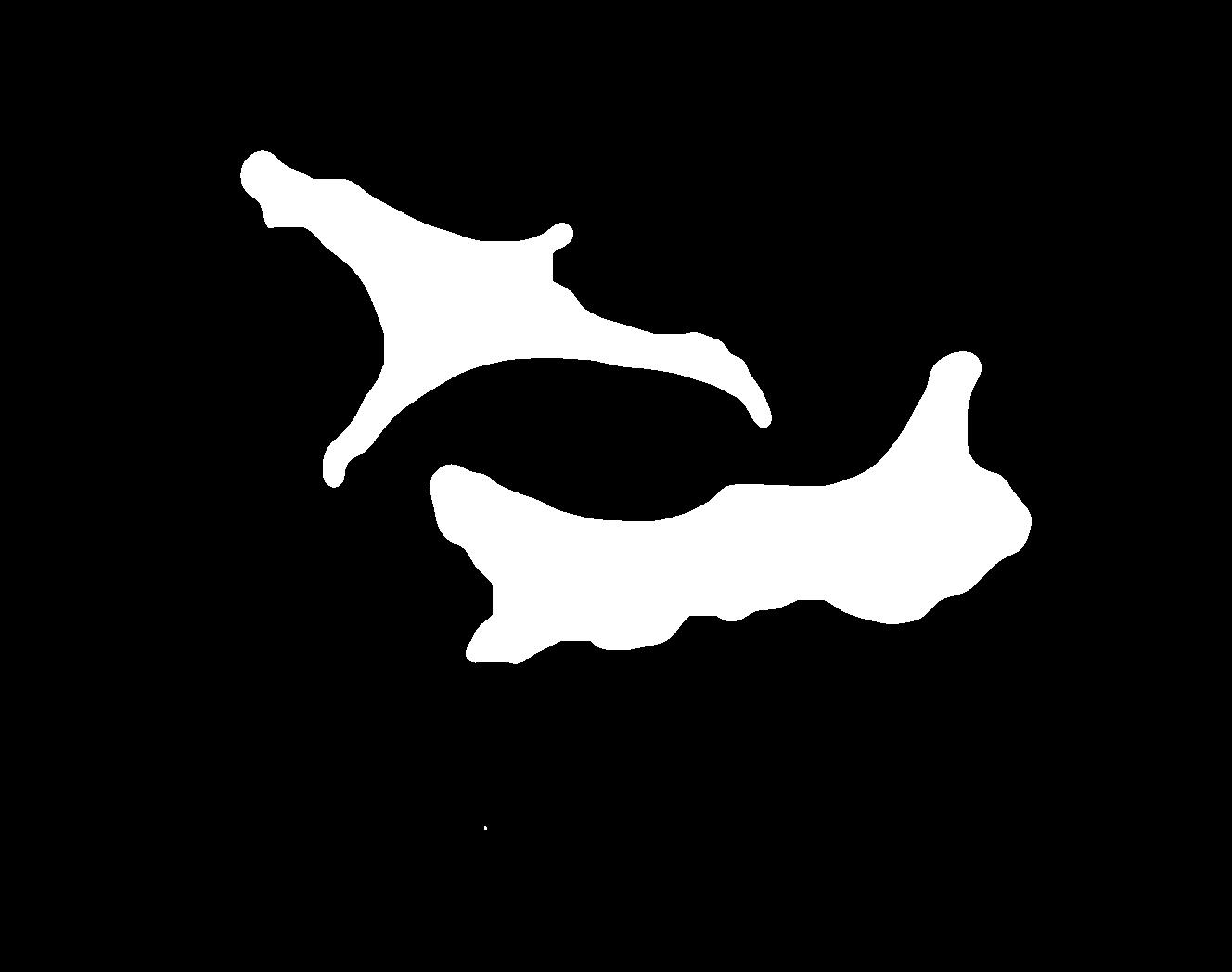

Supplement: S5 File — (ZIP) [file pone.0237972.s005.zip › S3_File IoU scores/masks/Experiment_1/cell/user_segmented/Composite/Composite_Participant9_mask_cell_G.jpg]

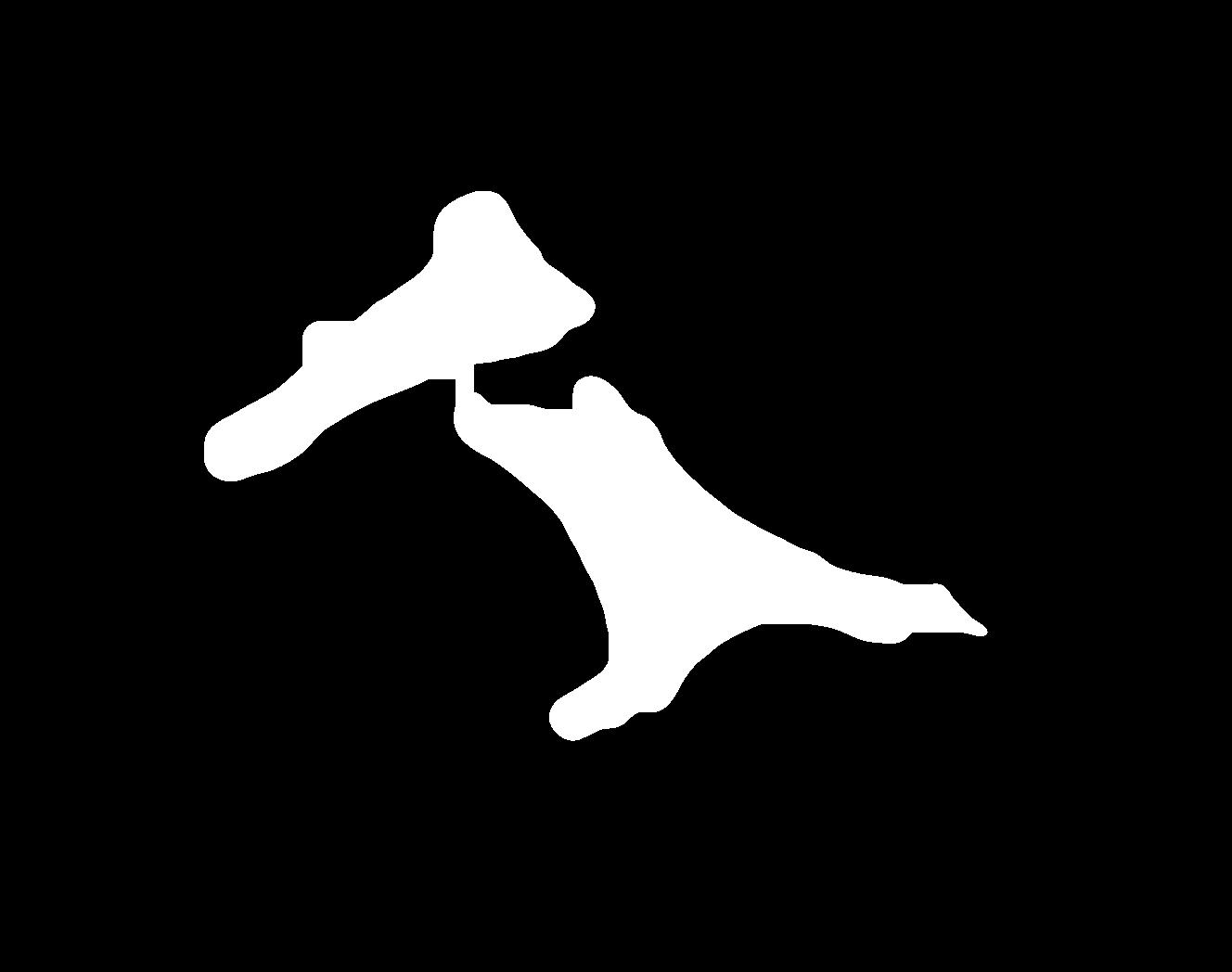

Supplement: S5 File — (ZIP) [file pone.0237972.s005.zip › S3_File IoU scores/masks/Experiment_1/cell/user_segmented/Composite/Composite_Participant9_mask_cell_H.jpg]

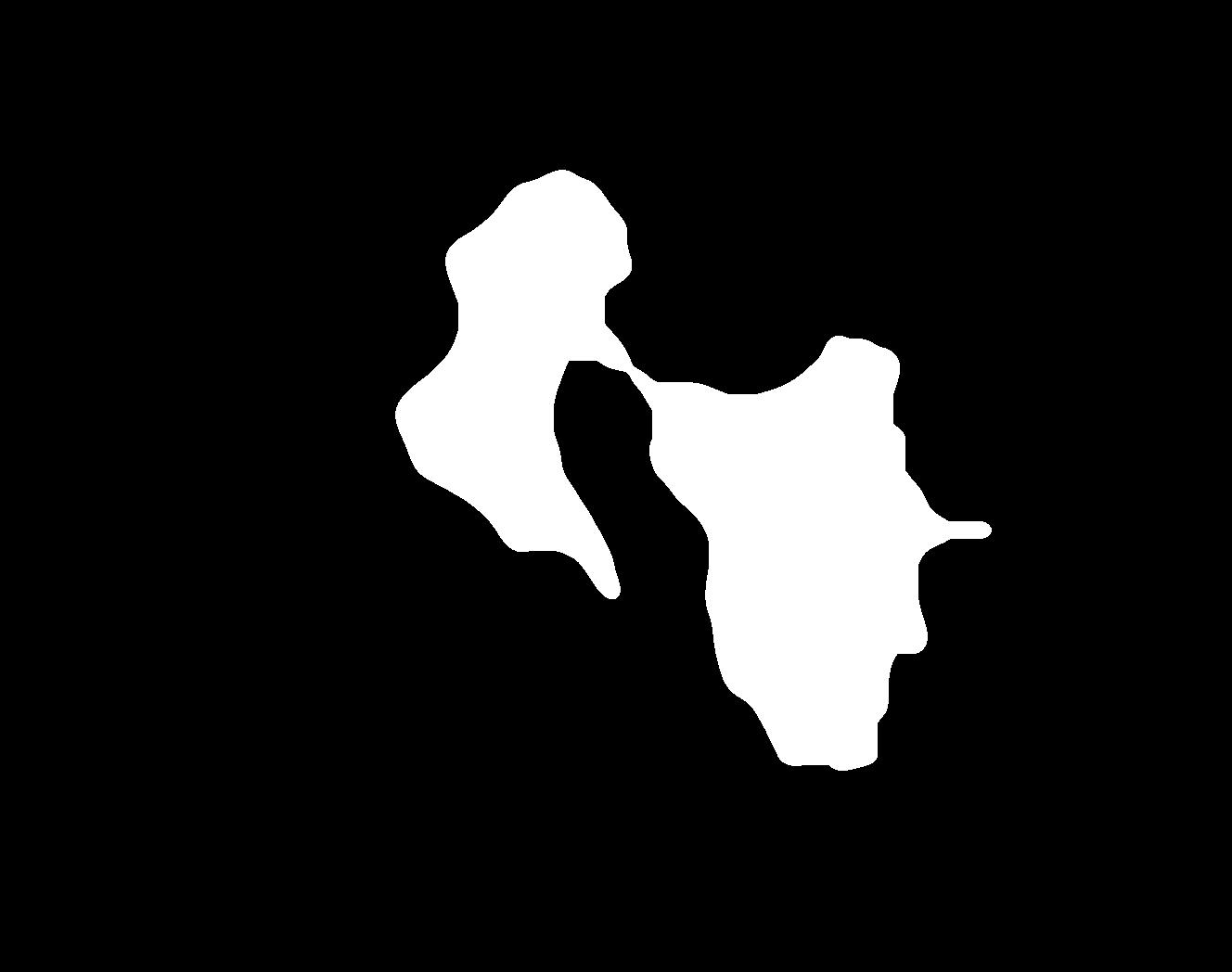

Supplement: S5 File — (ZIP) [file pone.0237972.s005.zip › S3_File IoU scores/masks/Experiment_1/cell/user_segmented/Composite/Composite_Participant9_mask_cell_I.jpg]

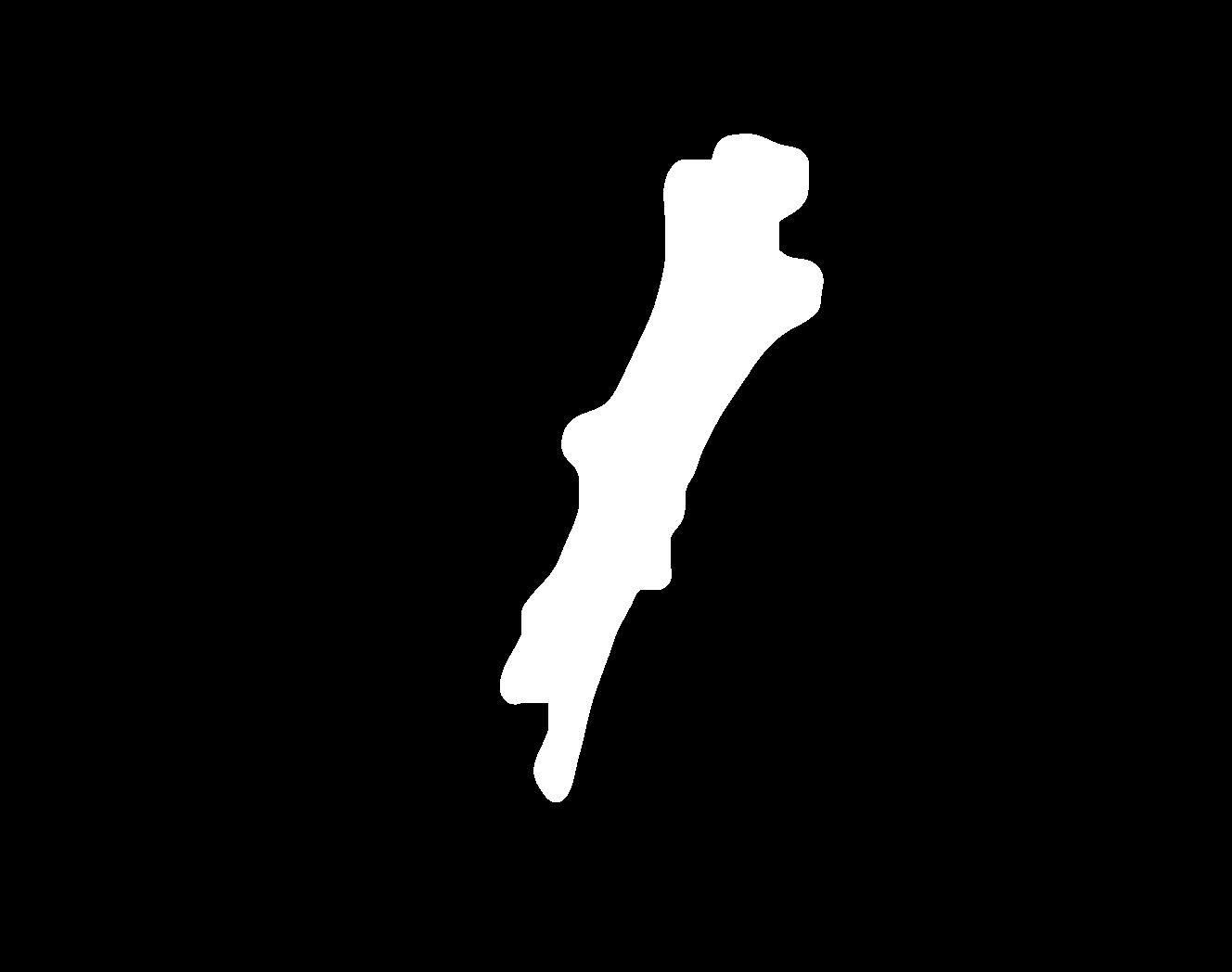

Supplement: S5 File — (ZIP) [file pone.0237972.s005.zip › S3_File IoU scores/masks/Experiment_1/cell/user_segmented/Composite/Composite_Participant9_mask_cell_J.jpg]

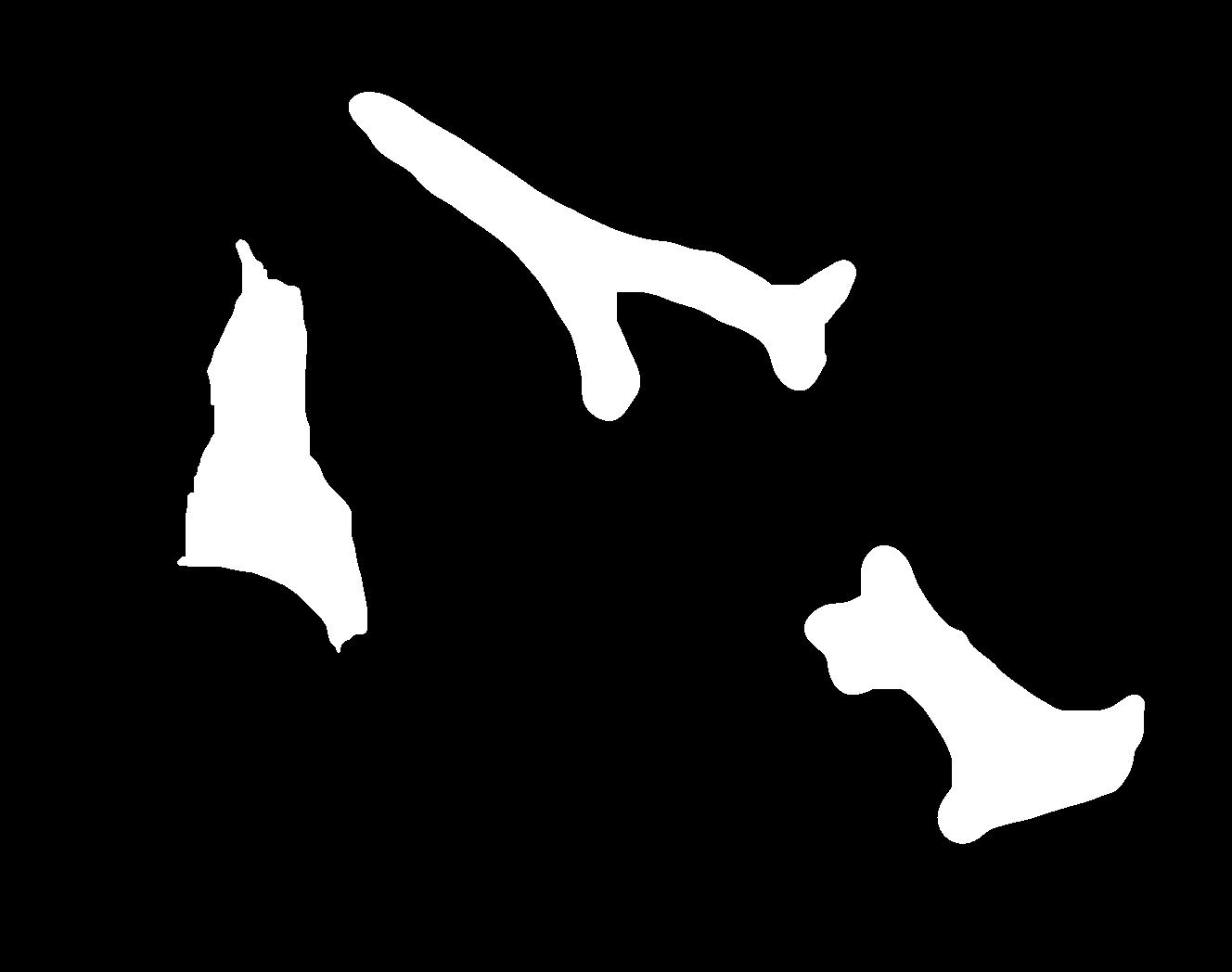

Supplement: S5 File — (ZIP) [file pone.0237972.s005.zip › S3_File IoU scores/masks/Experiment_1/cell/user_segmented/Manual/Manual_Participant13_mask_cell_A.jpg]

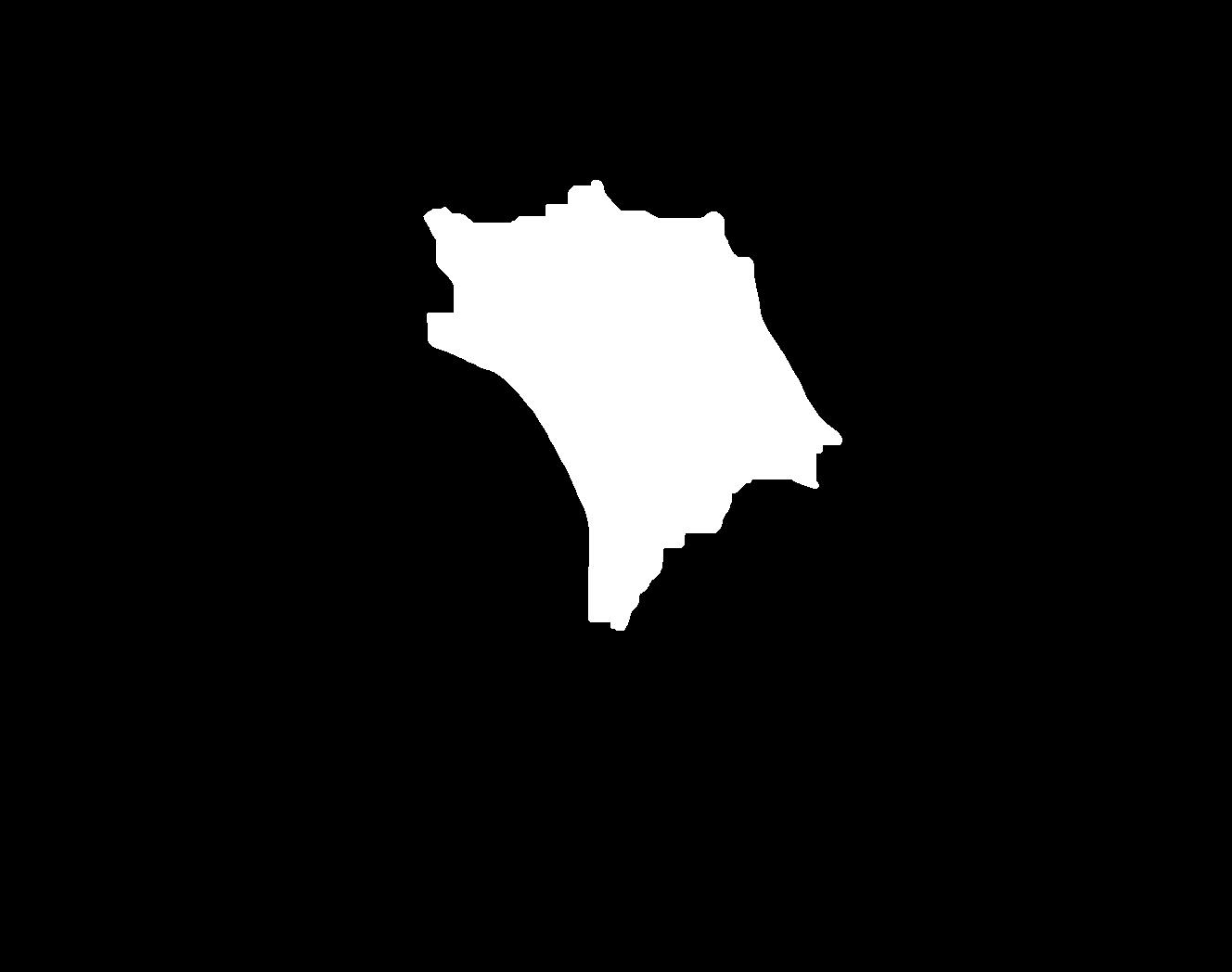

Supplement: S5 File — (ZIP) [file pone.0237972.s005.zip › S3_File IoU scores/masks/Experiment_1/cell/user_segmented/Manual/Manual_Participant13_mask_cell_B.jpg]

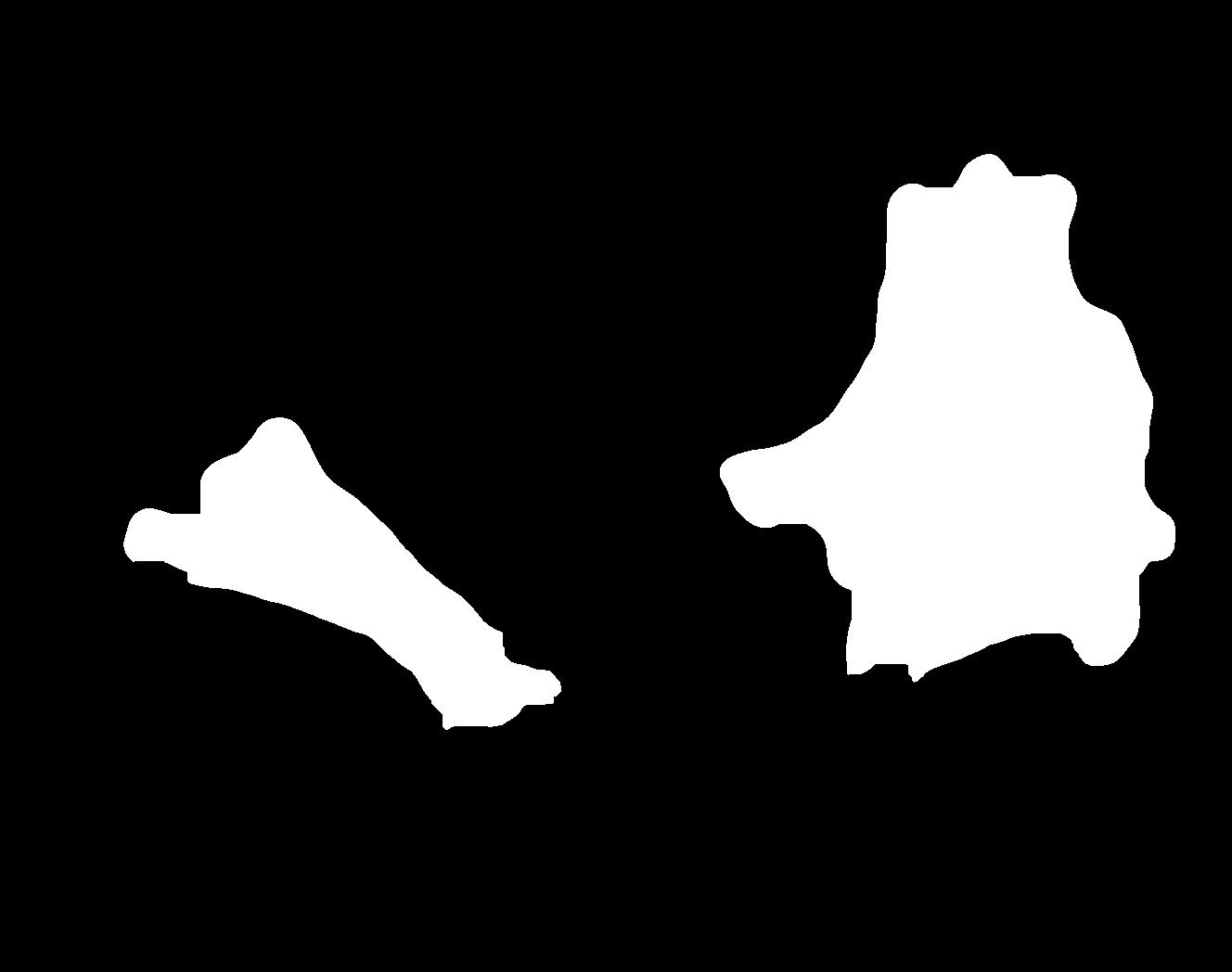

Supplement: S5 File — (ZIP) [file pone.0237972.s005.zip › S3_File IoU scores/masks/Experiment_1/cell/user_segmented/Manual/Manual_Participant13_mask_cell_C.jpg]

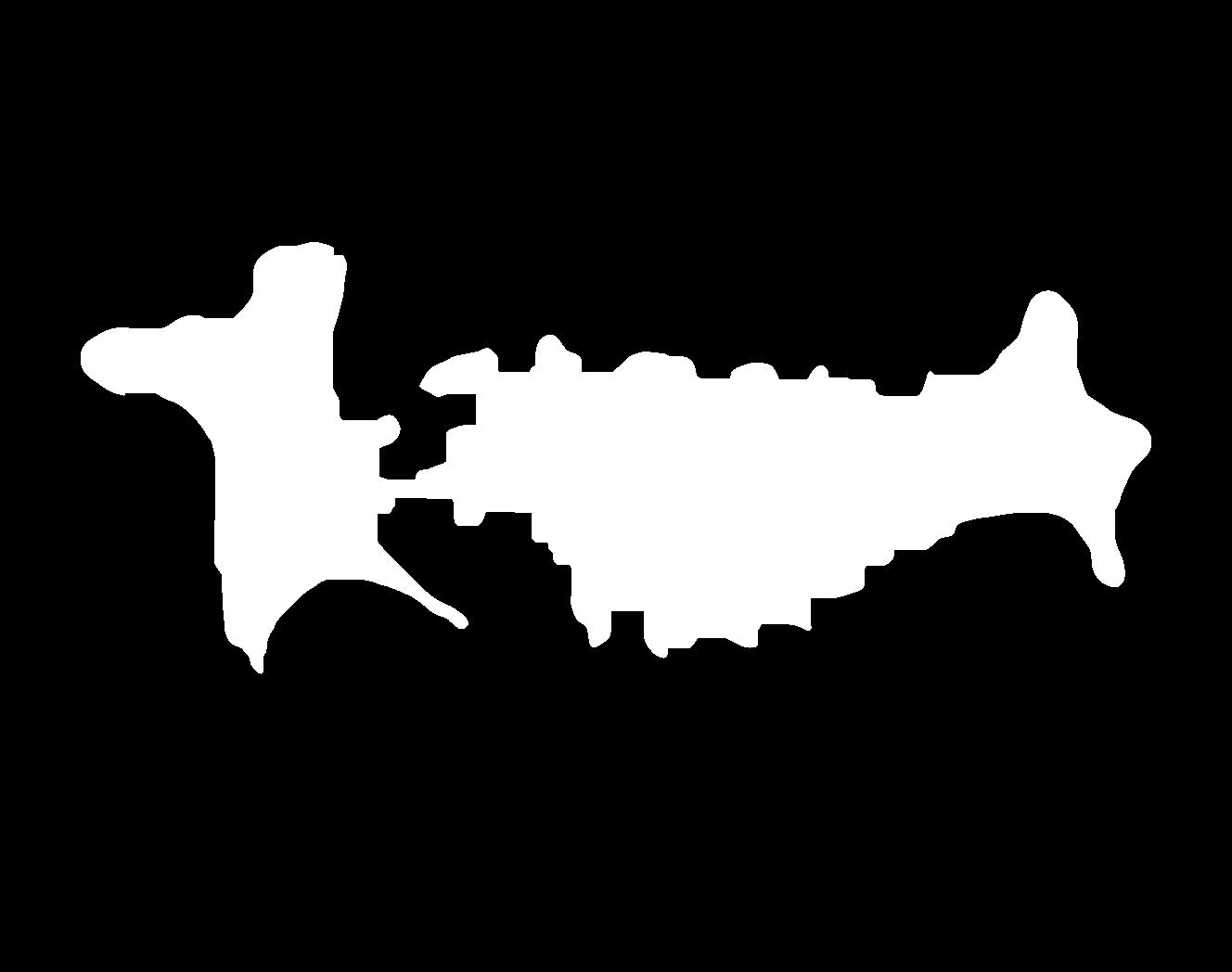

Supplement: S5 File — (ZIP) [file pone.0237972.s005.zip › S3_File IoU scores/masks/Experiment_1/cell/user_segmented/Manual/Manual_Participant13_mask_cell_D.jpg]

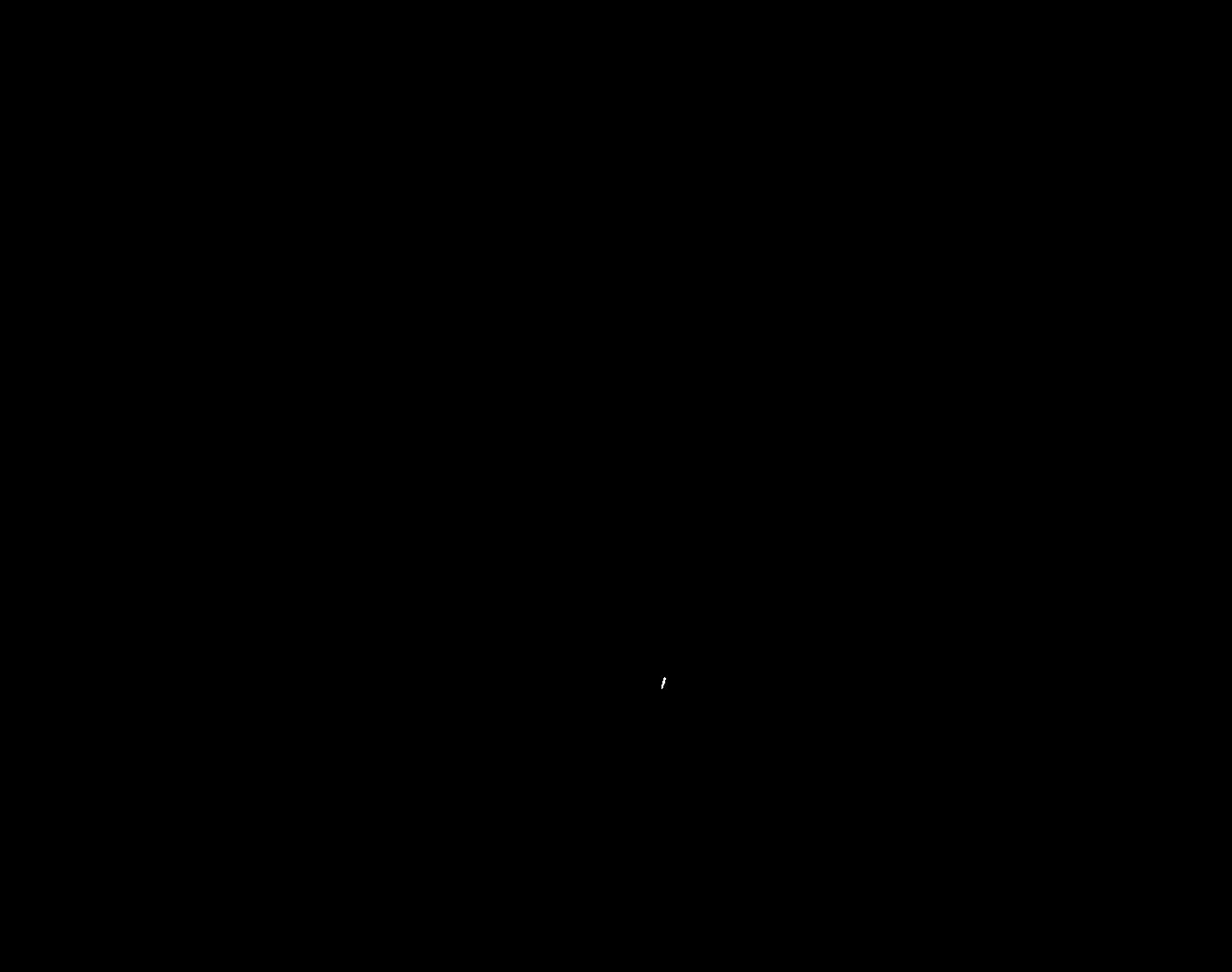

Supplement: S5 File — (ZIP) [file pone.0237972.s005.zip › S3_File IoU scores/masks/Experiment_1/cell/user_segmented/Manual/Manual_Participant13_mask_cell_E.jpg]
