# Supplementary figures and images for: Interactive machine learning for fast and robust cell profiling (part 1 of 2)
Source: PLoS One. 2020 Sep 11;15(9):e0237972. doi: 10.1371/journal.pone.0237972 (PMC7485821; doi:10.1371/journal.pone.0237972)

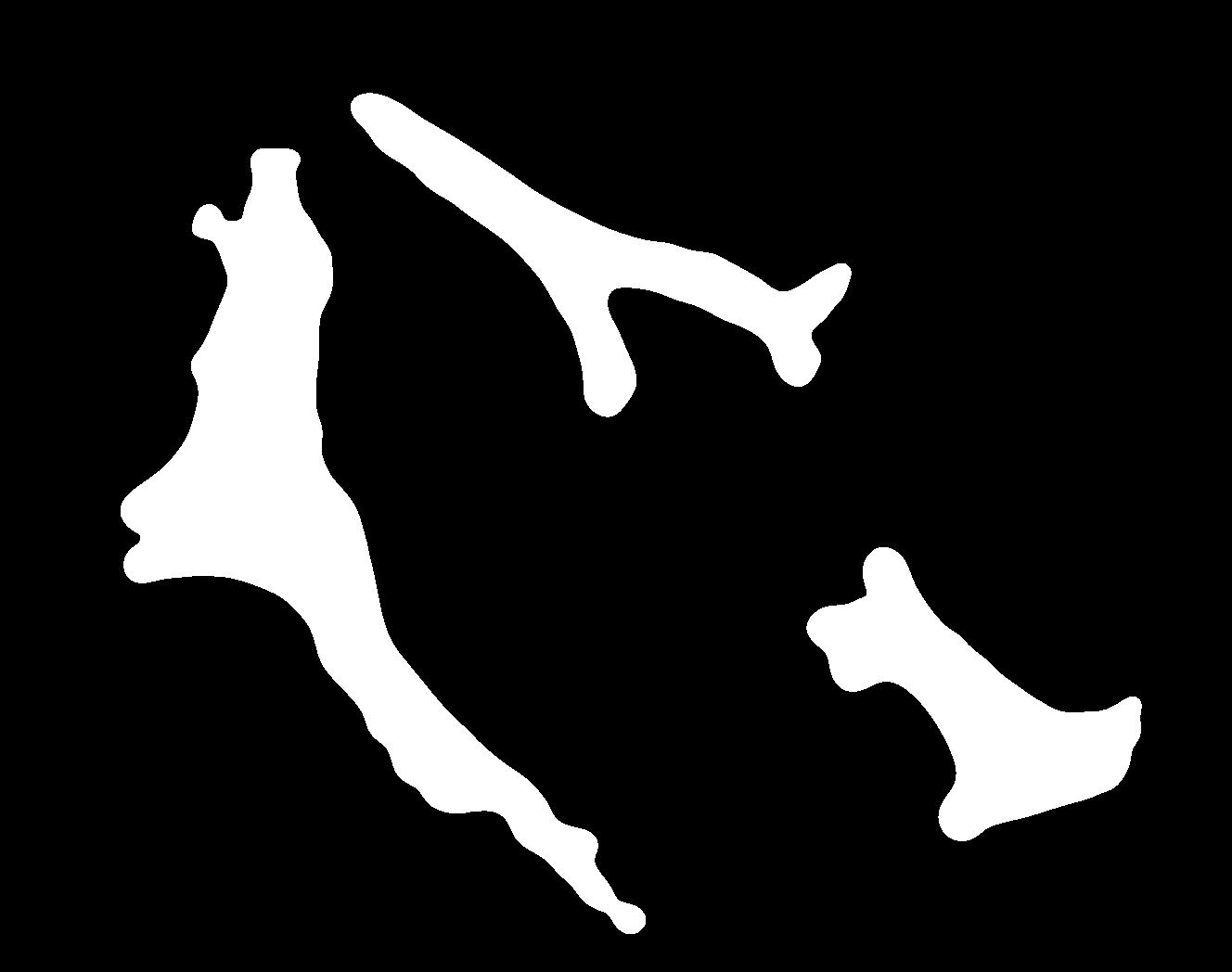

Supplement: S5 File — (ZIP) [file pone.0237972.s005.zip › S3_File IoU scores/masks/Experiment_1/cell/cp_expert/fixedPipeline_mask_cell_A.jpg]

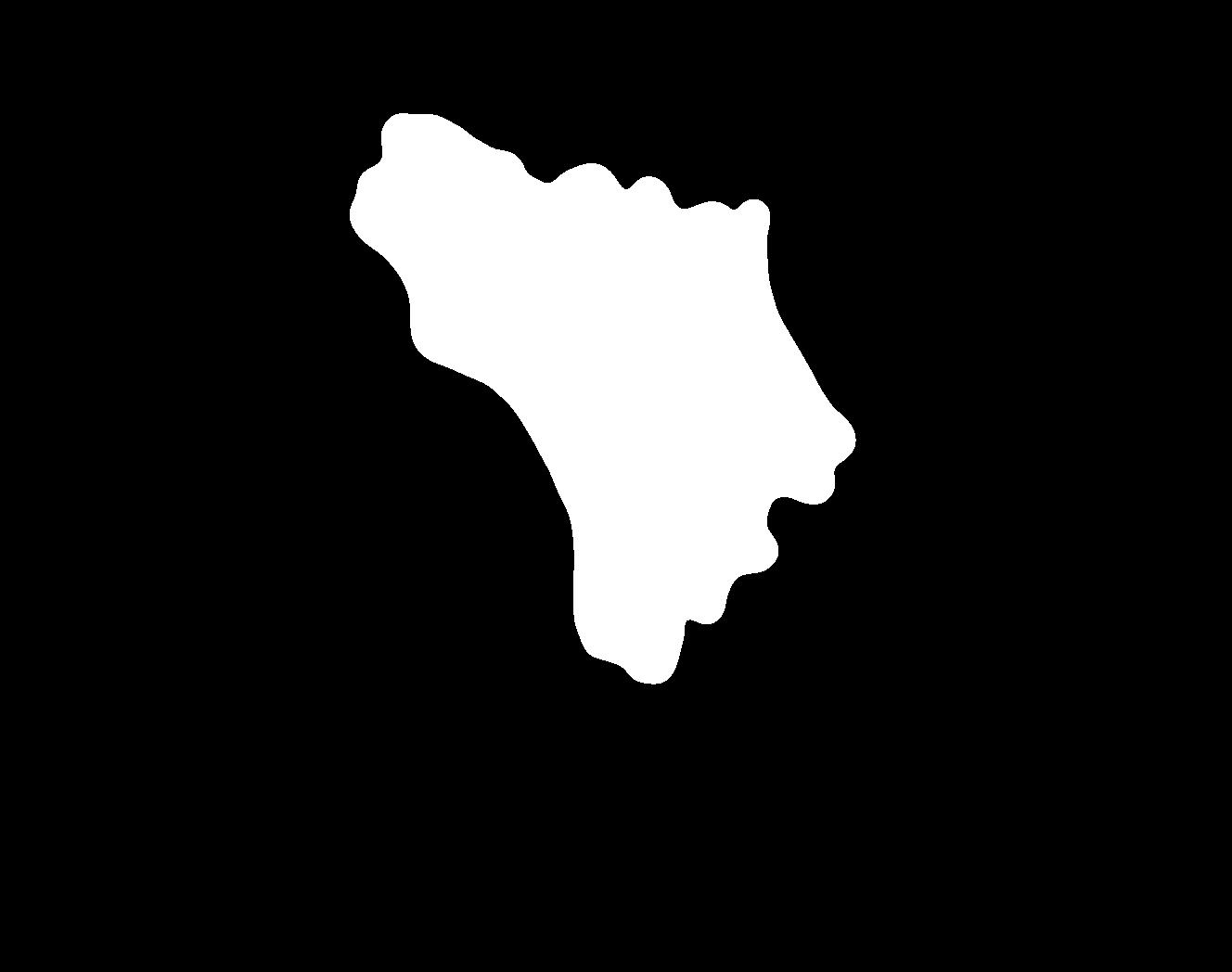

Supplement: S5 File — (ZIP) [file pone.0237972.s005.zip › S3_File IoU scores/masks/Experiment_1/cell/cp_expert/fixedPipeline_mask_cell_B.jpg]

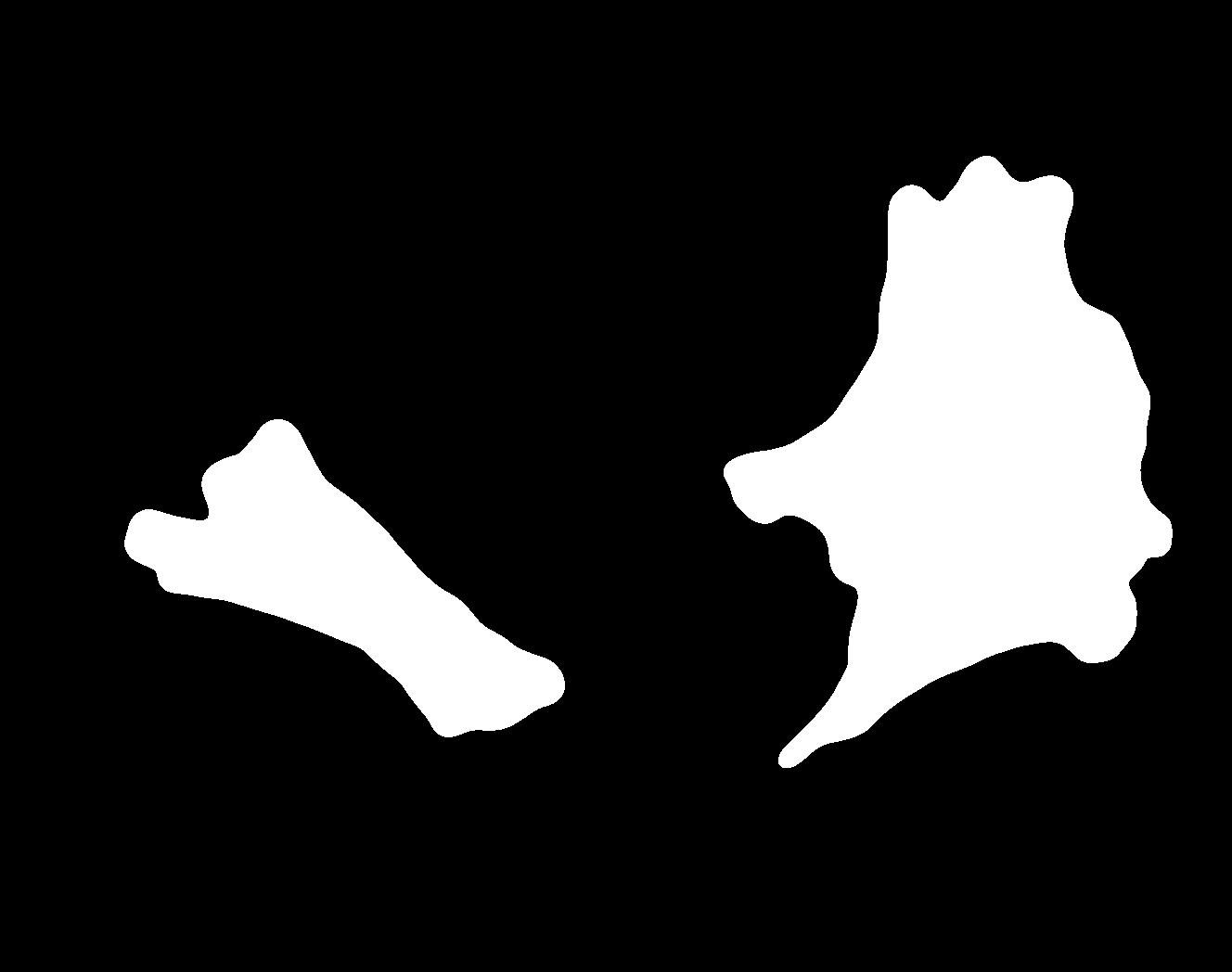

Supplement: S5 File — (ZIP) [file pone.0237972.s005.zip › S3_File IoU scores/masks/Experiment_1/cell/cp_expert/fixedPipeline_mask_cell_C.jpg]

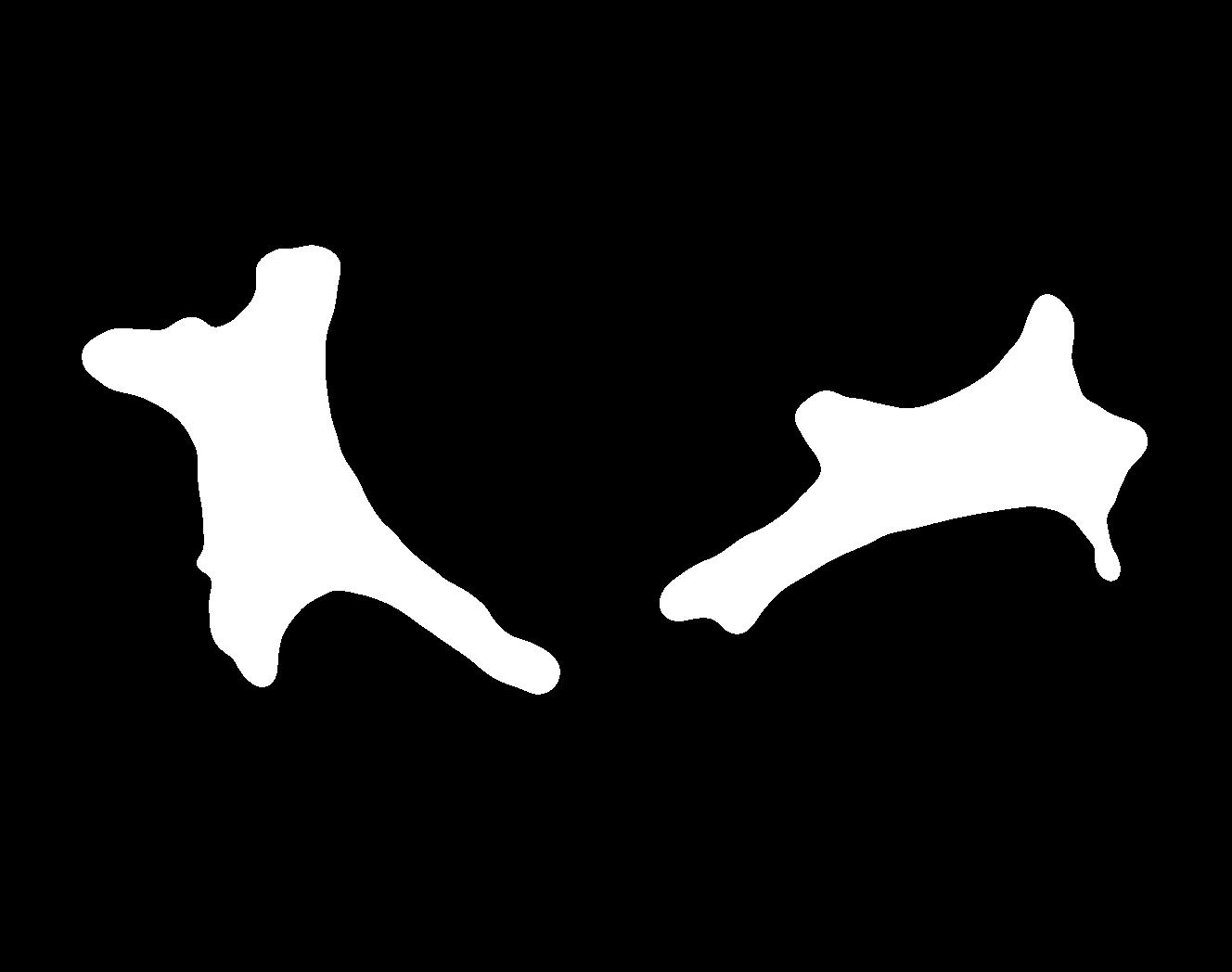

Supplement: S5 File — (ZIP) [file pone.0237972.s005.zip › S3_File IoU scores/masks/Experiment_1/cell/cp_expert/fixedPipeline_mask_cell_D.jpg]

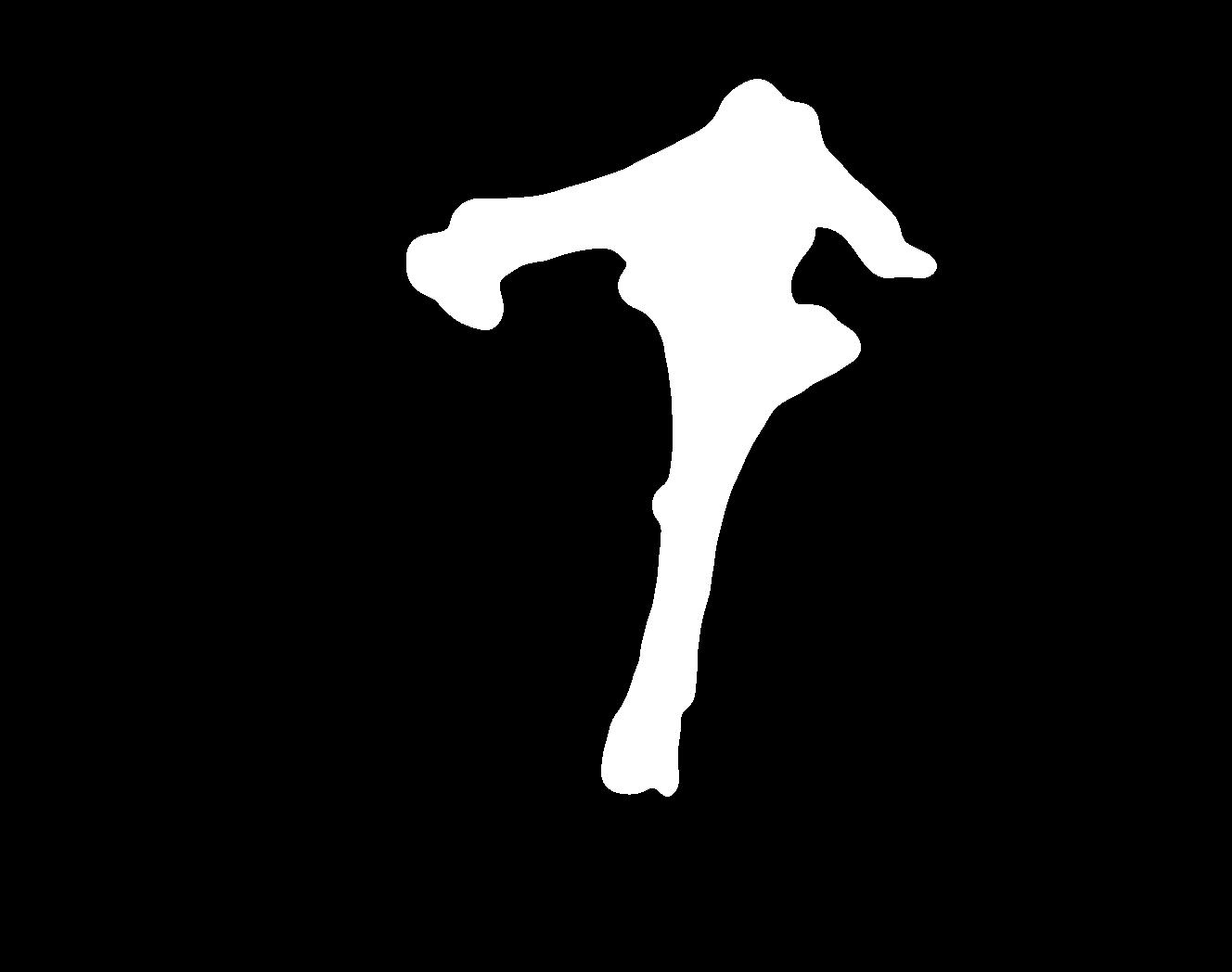

Supplement: S5 File — (ZIP) [file pone.0237972.s005.zip › S3_File IoU scores/masks/Experiment_1/cell/cp_expert/fixedPipeline_mask_cell_E.jpg]

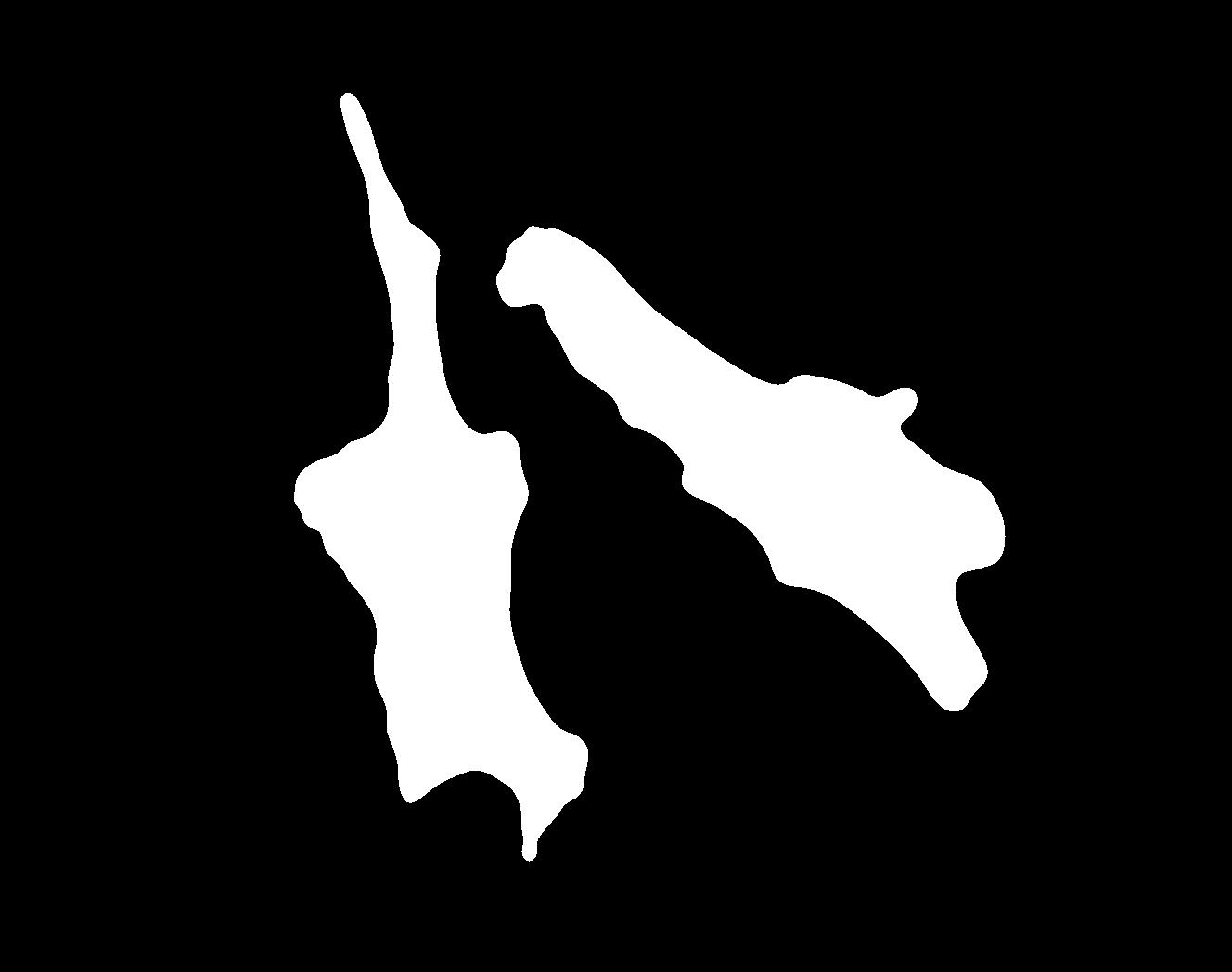

Supplement: S5 File — (ZIP) [file pone.0237972.s005.zip › S3_File IoU scores/masks/Experiment_1/cell/cp_expert/fixedPipeline_mask_cell_F.jpg]

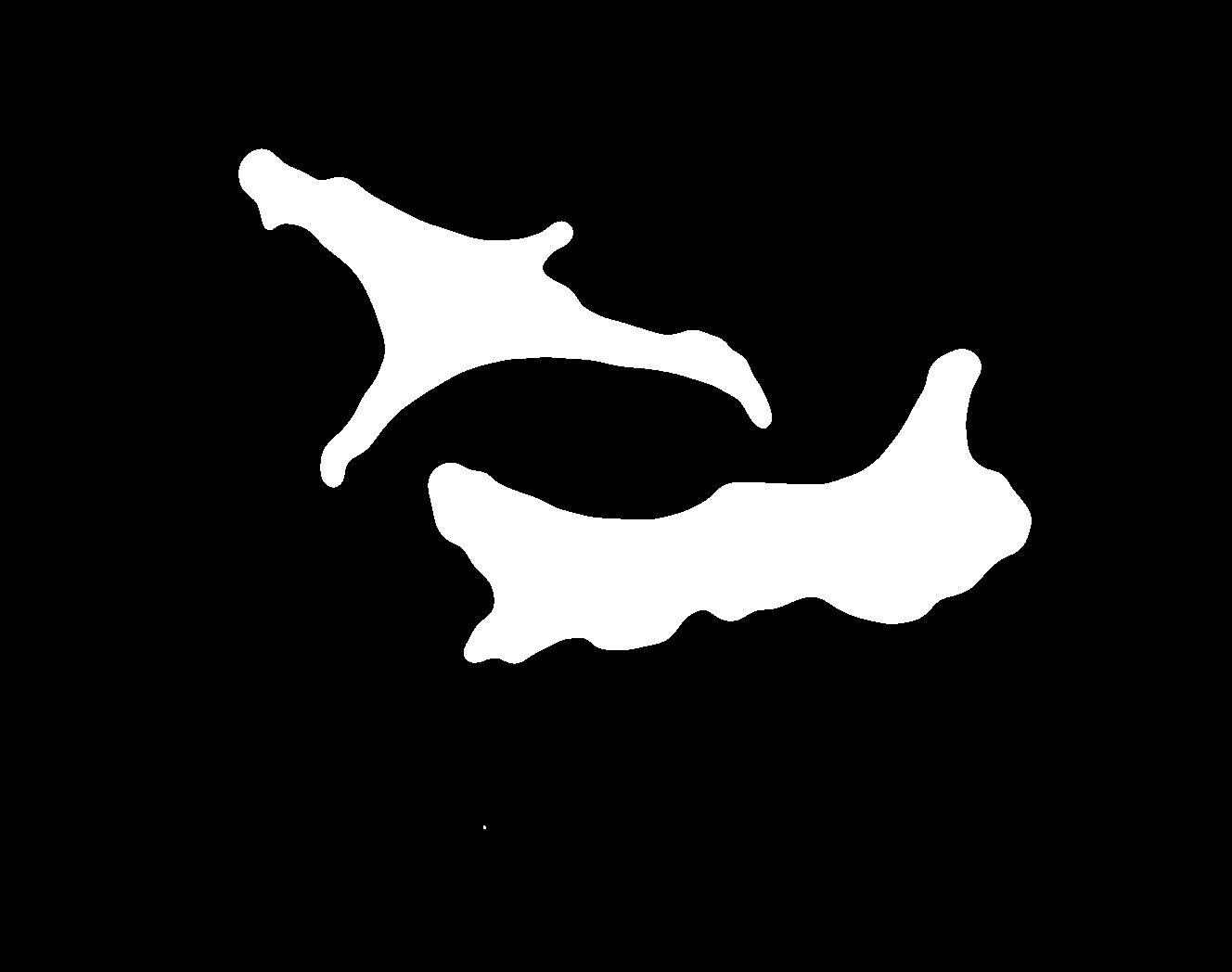

Supplement: S5 File — (ZIP) [file pone.0237972.s005.zip › S3_File IoU scores/masks/Experiment_1/cell/cp_expert/fixedPipeline_mask_cell_G.jpg]

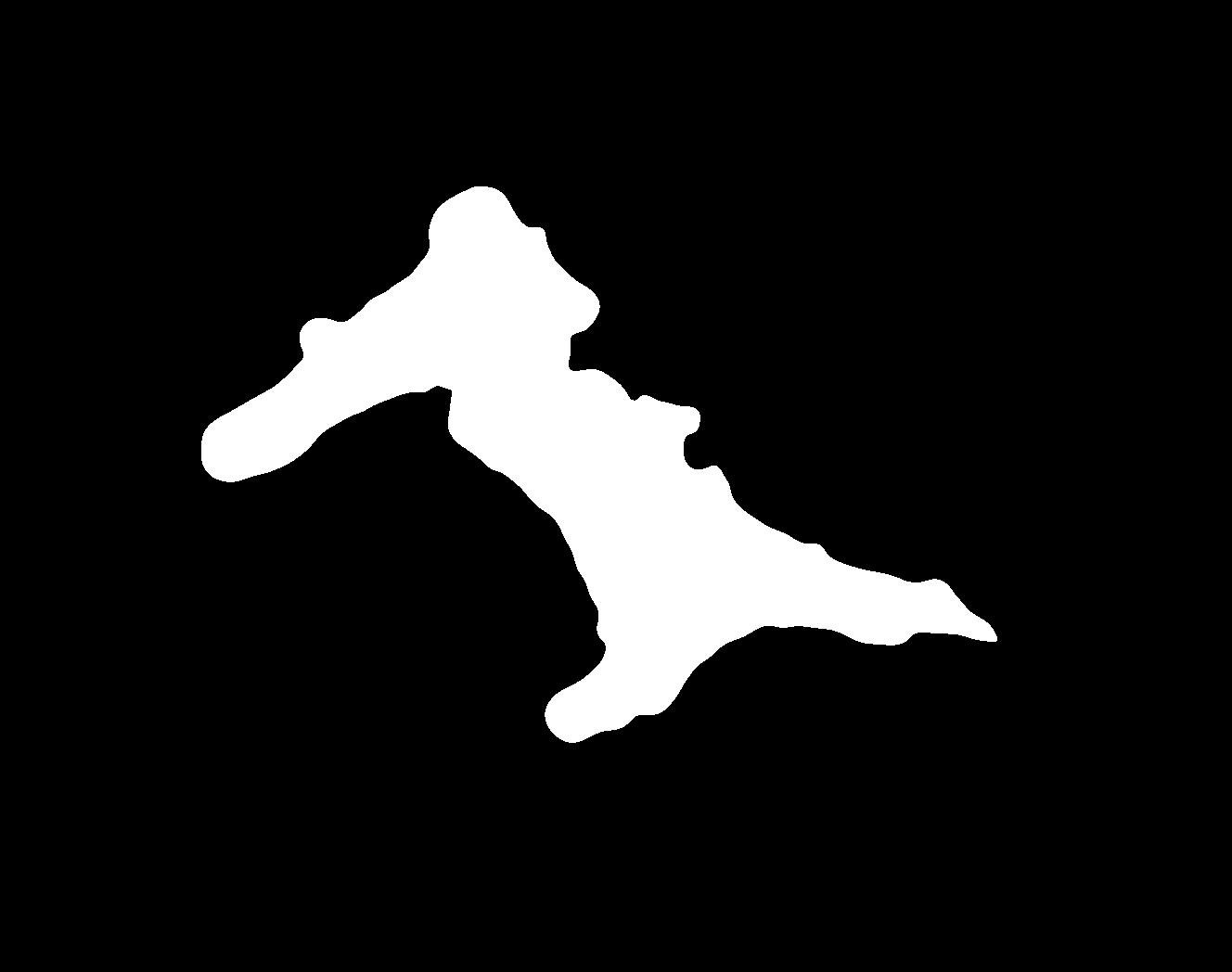

Supplement: S5 File — (ZIP) [file pone.0237972.s005.zip › S3_File IoU scores/masks/Experiment_1/cell/cp_expert/fixedPipeline_mask_cell_H.jpg]

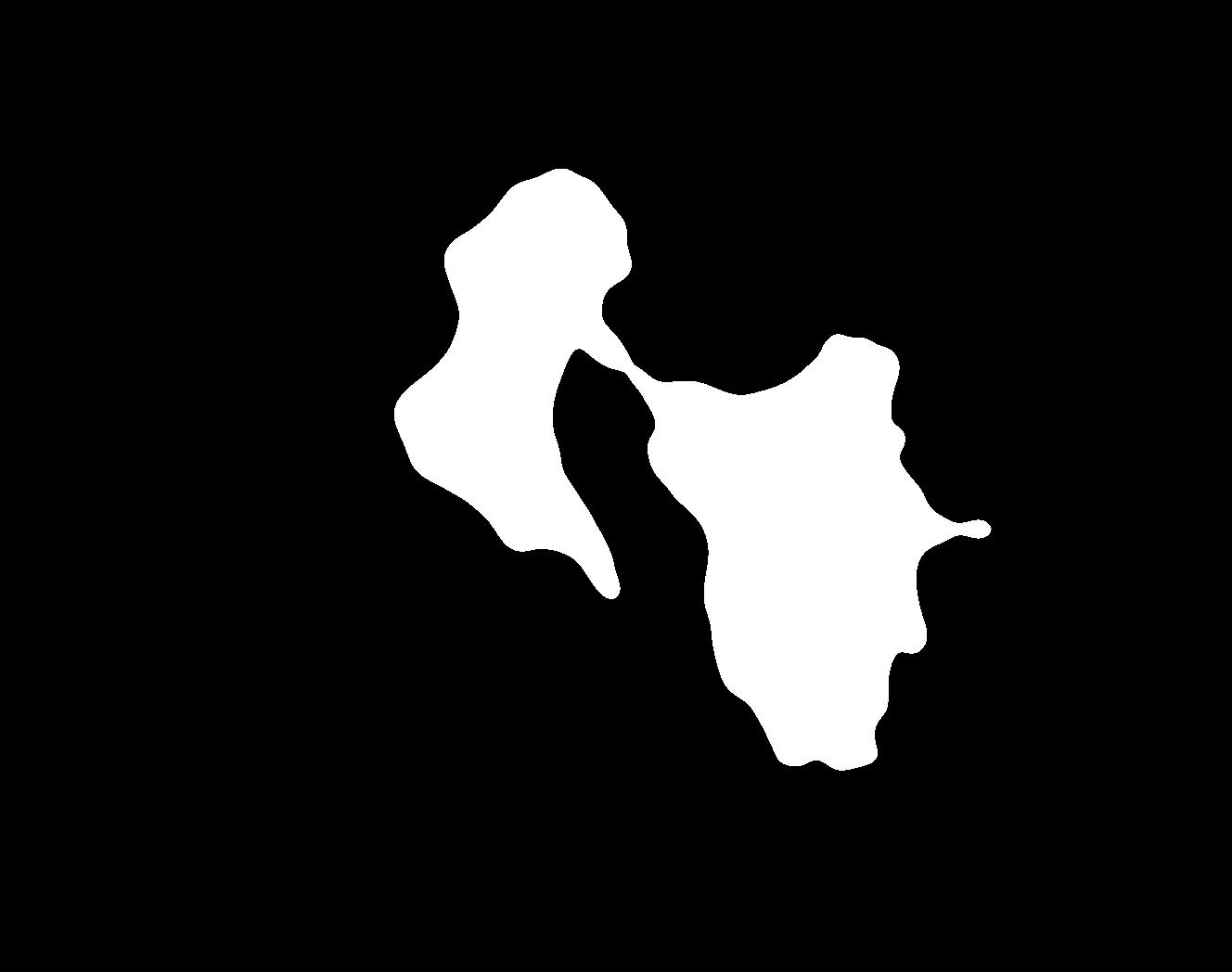

Supplement: S5 File — (ZIP) [file pone.0237972.s005.zip › S3_File IoU scores/masks/Experiment_1/cell/cp_expert/fixedPipeline_mask_cell_I.jpg]

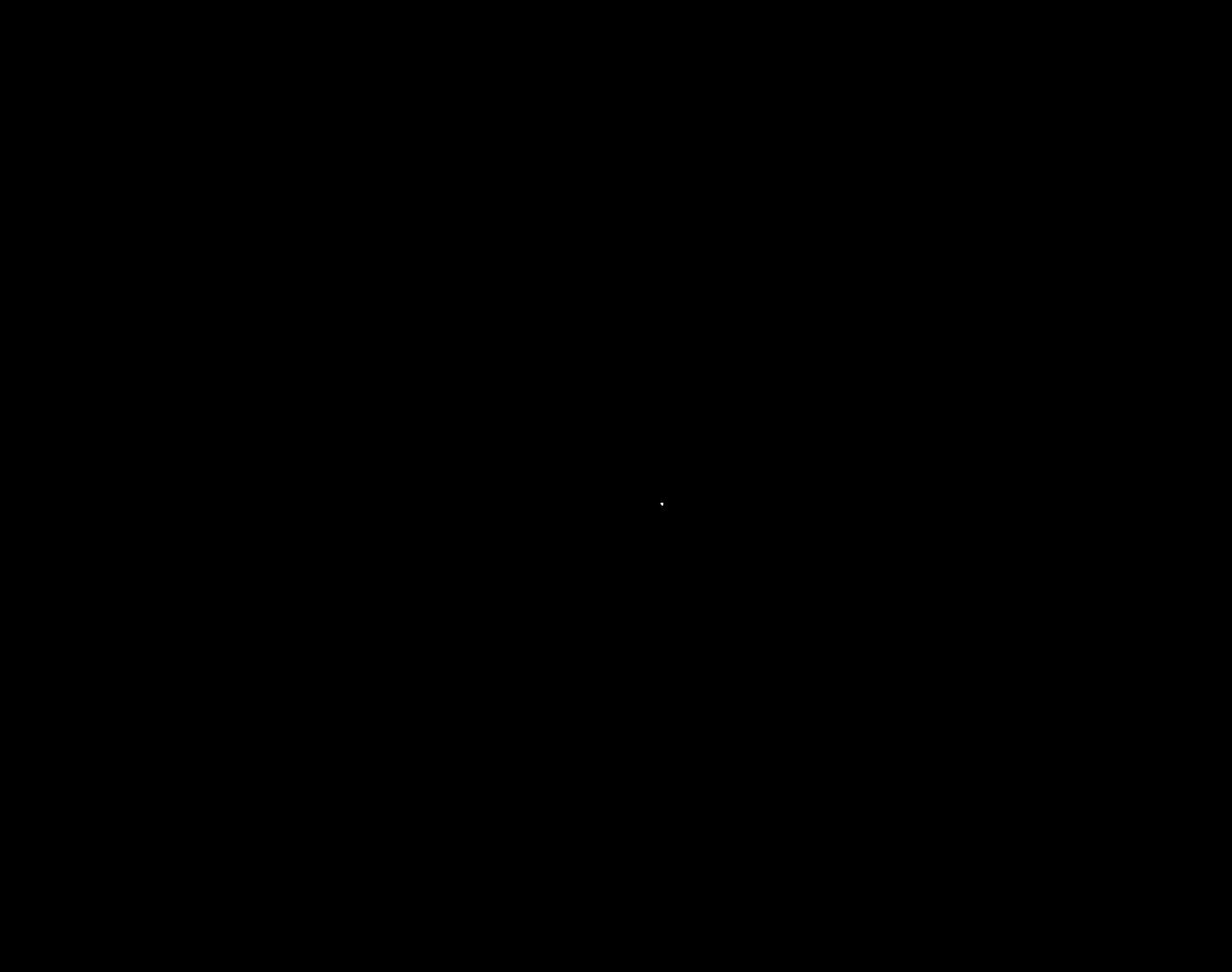

Supplement: S5 File — (ZIP) [file pone.0237972.s005.zip › S3_File IoU scores/masks/Experiment_1/cell/cp_expert/fixedPipeline_mask_cell_J.jpg]

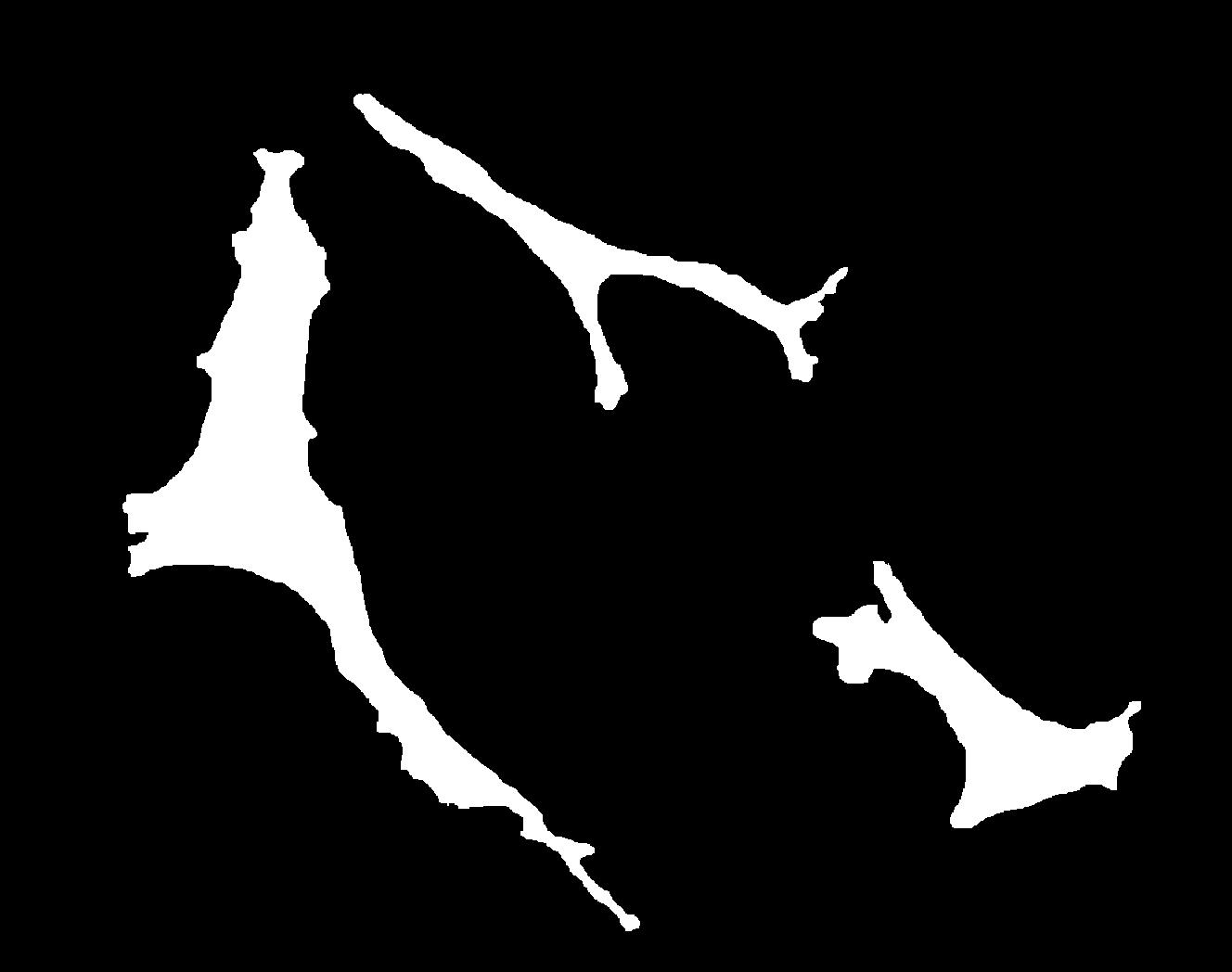

Supplement: S5 File — (ZIP) [file pone.0237972.s005.zip › S3_File IoU scores/masks/Experiment_1/cell/ground_truth/A_groundtruth.jpg]

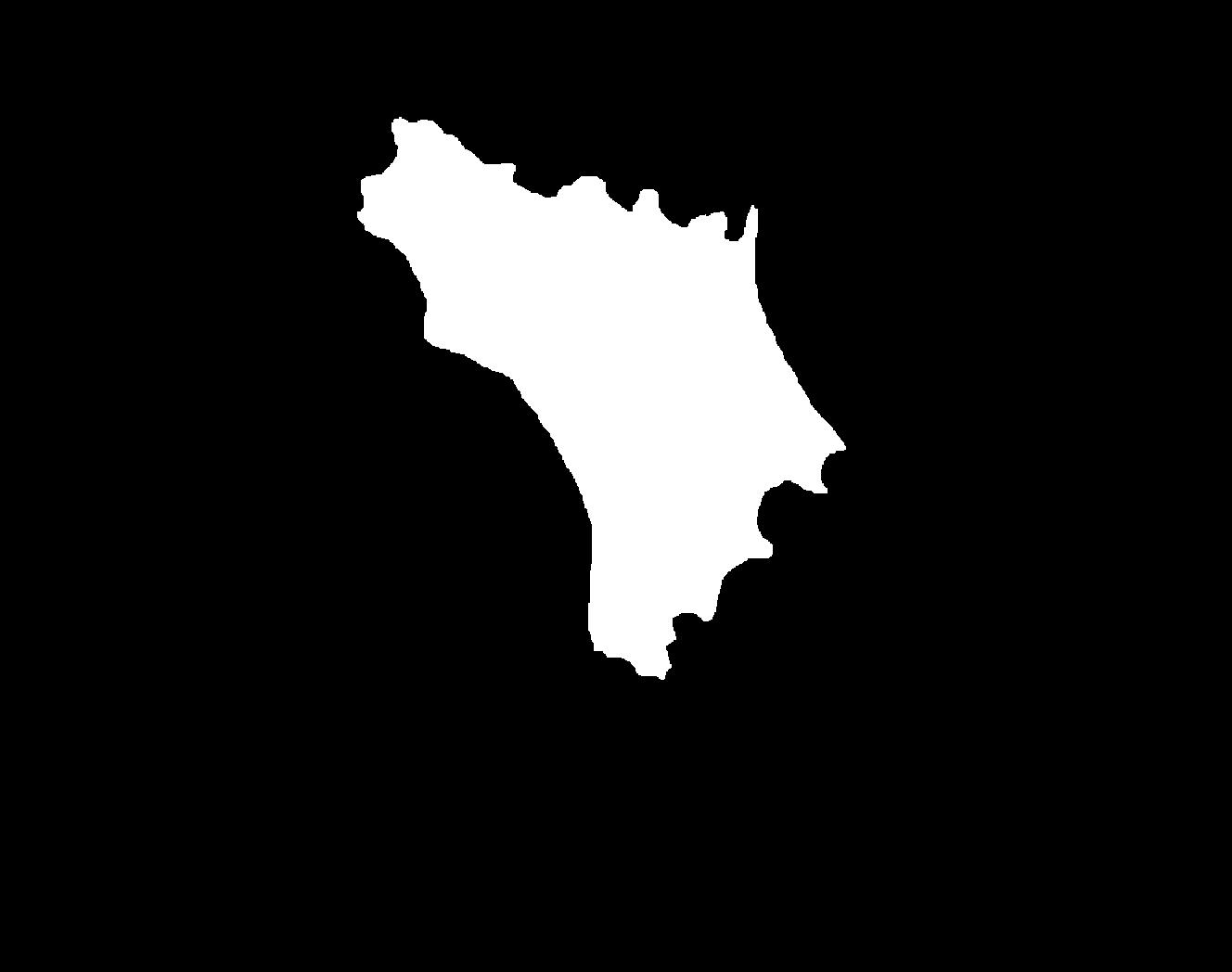

Supplement: S5 File — (ZIP) [file pone.0237972.s005.zip › S3_File IoU scores/masks/Experiment_1/cell/ground_truth/B_groundtruth.jpg]

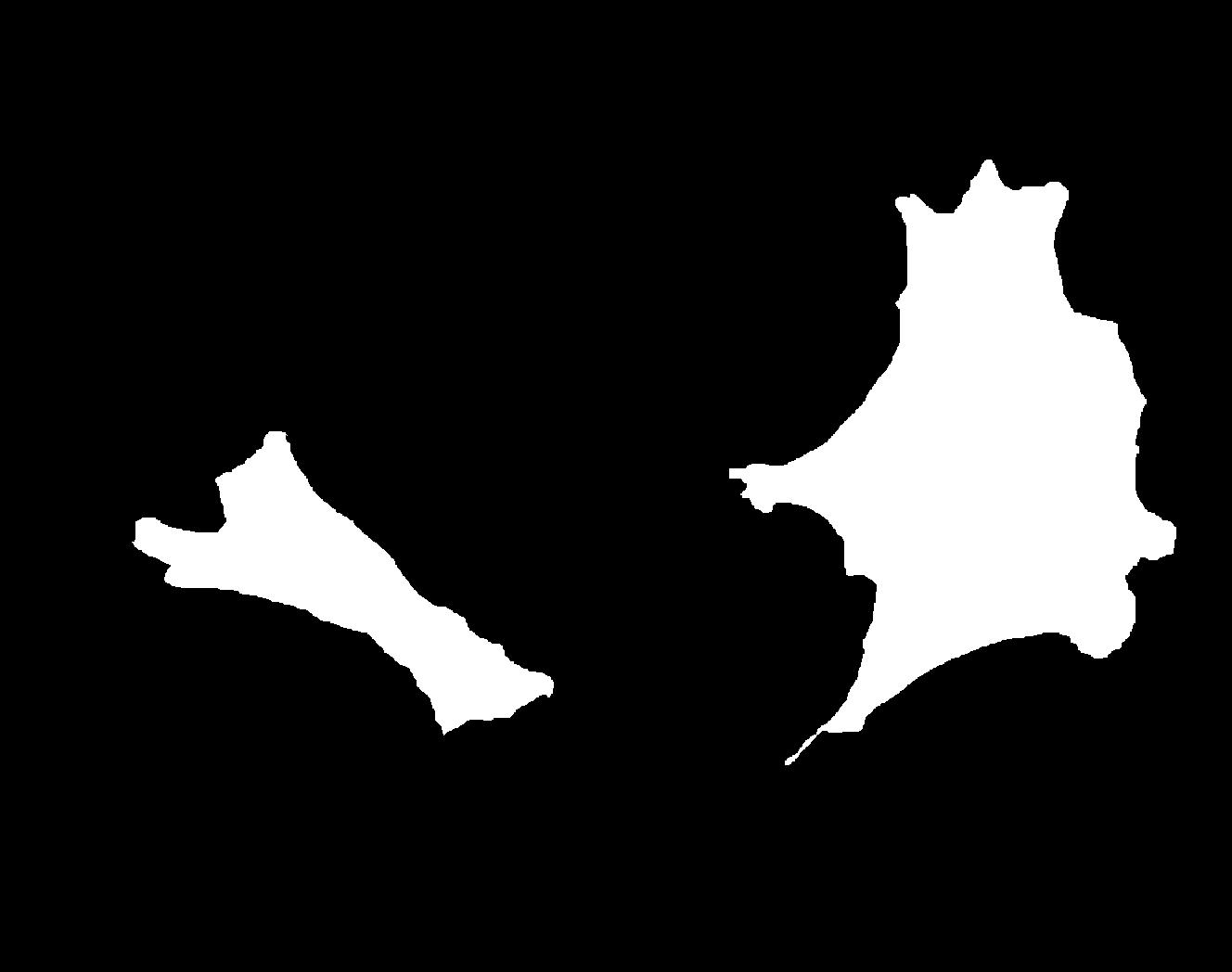

Supplement: S5 File — (ZIP) [file pone.0237972.s005.zip › S3_File IoU scores/masks/Experiment_1/cell/ground_truth/C_groundtruth.jpg]

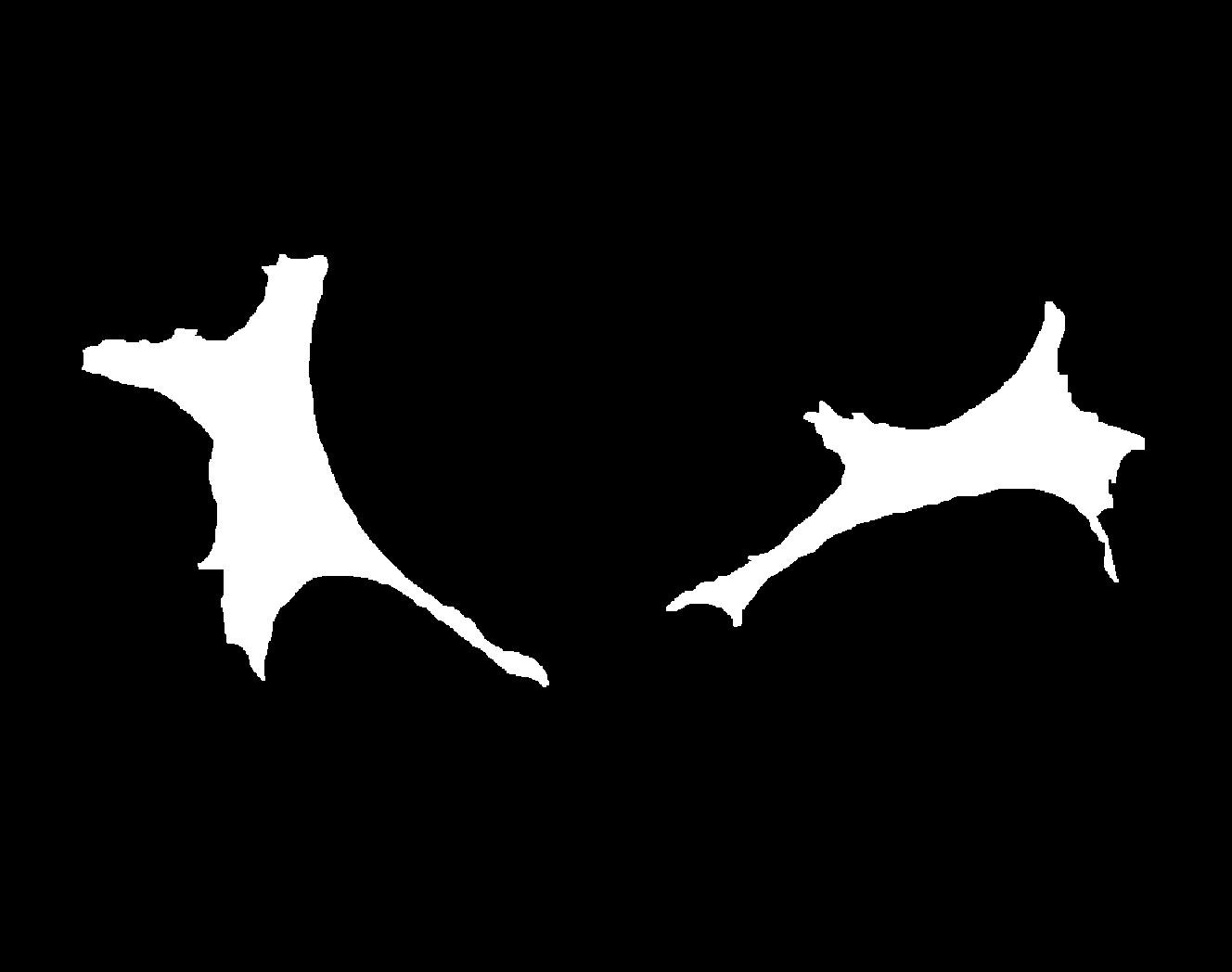

Supplement: S5 File — (ZIP) [file pone.0237972.s005.zip › S3_File IoU scores/masks/Experiment_1/cell/ground_truth/D_groundtruth.jpg]

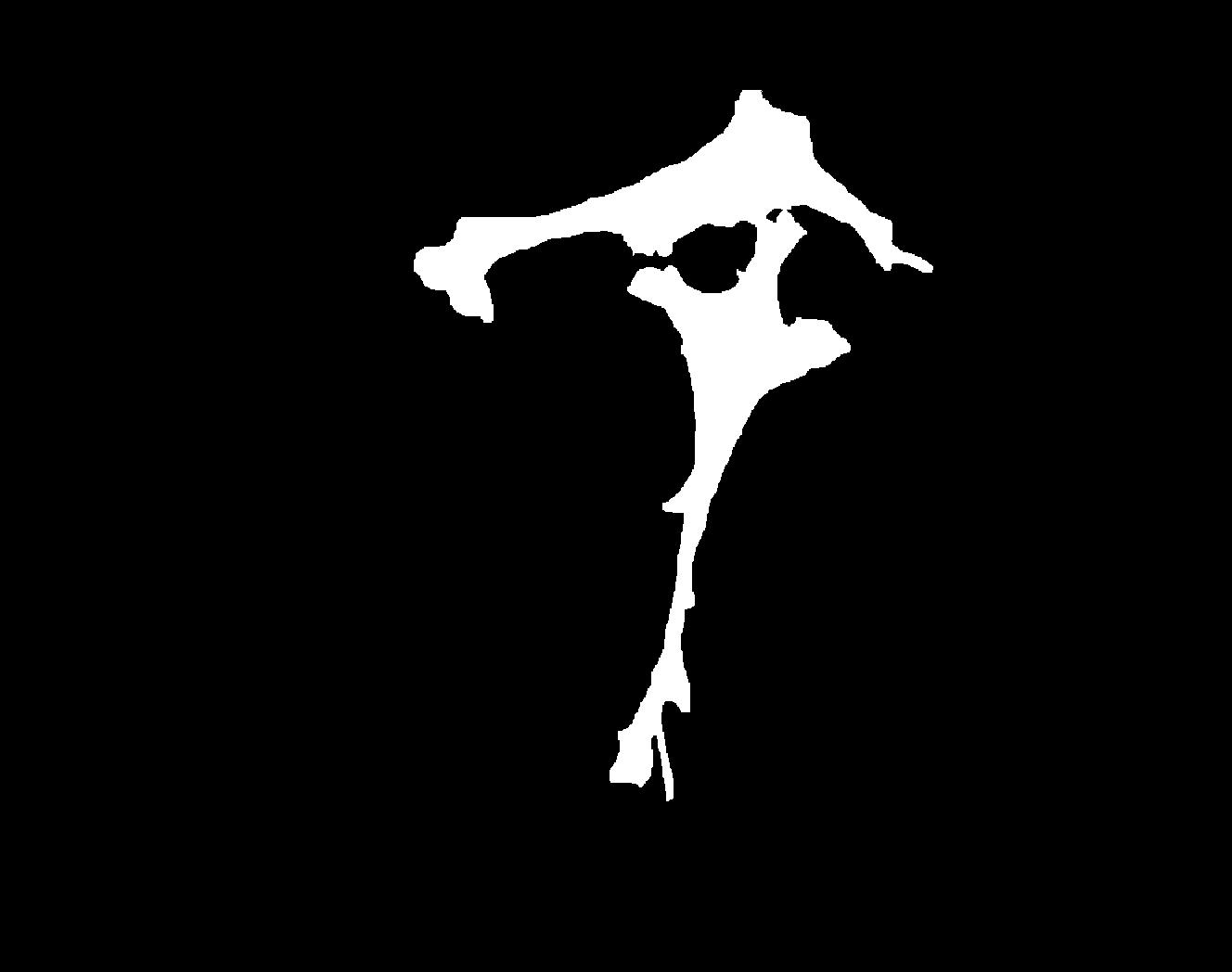

Supplement: S5 File — (ZIP) [file pone.0237972.s005.zip › S3_File IoU scores/masks/Experiment_1/cell/ground_truth/E_groundtruth.jpg]

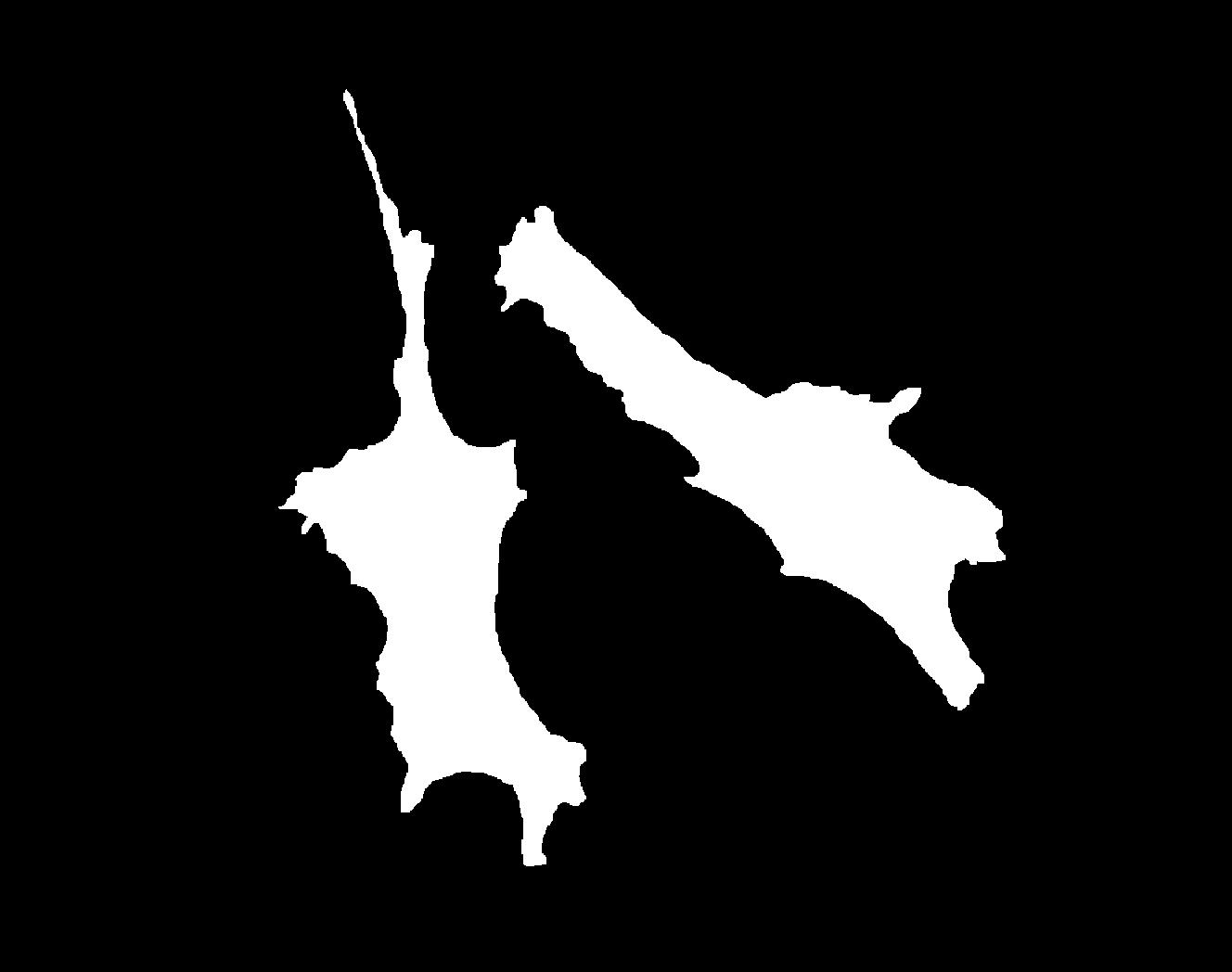

Supplement: S5 File — (ZIP) [file pone.0237972.s005.zip › S3_File IoU scores/masks/Experiment_1/cell/ground_truth/F_groundtruth.jpg]

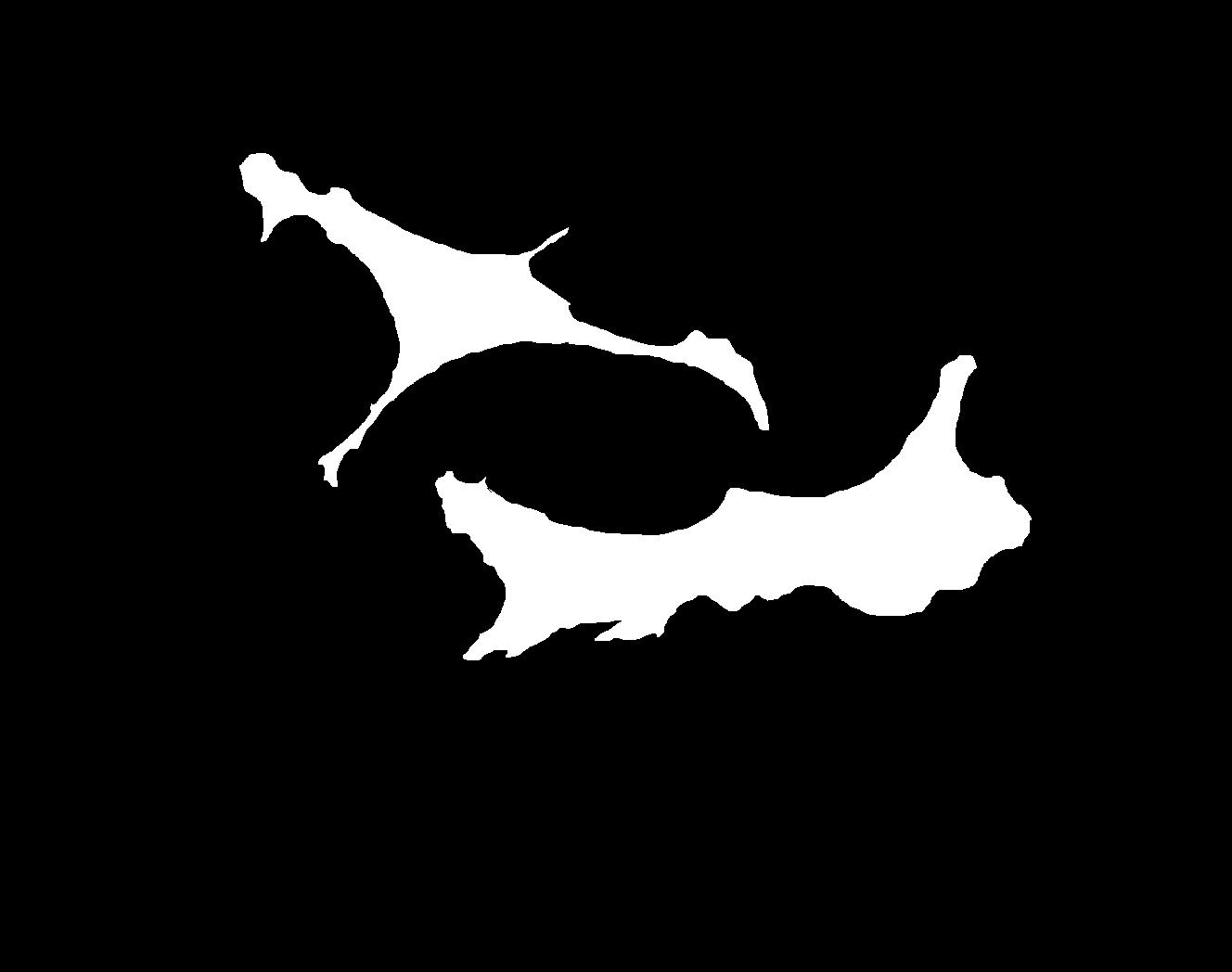

Supplement: S5 File — (ZIP) [file pone.0237972.s005.zip › S3_File IoU scores/masks/Experiment_1/cell/ground_truth/G_groundtruth.jpg]

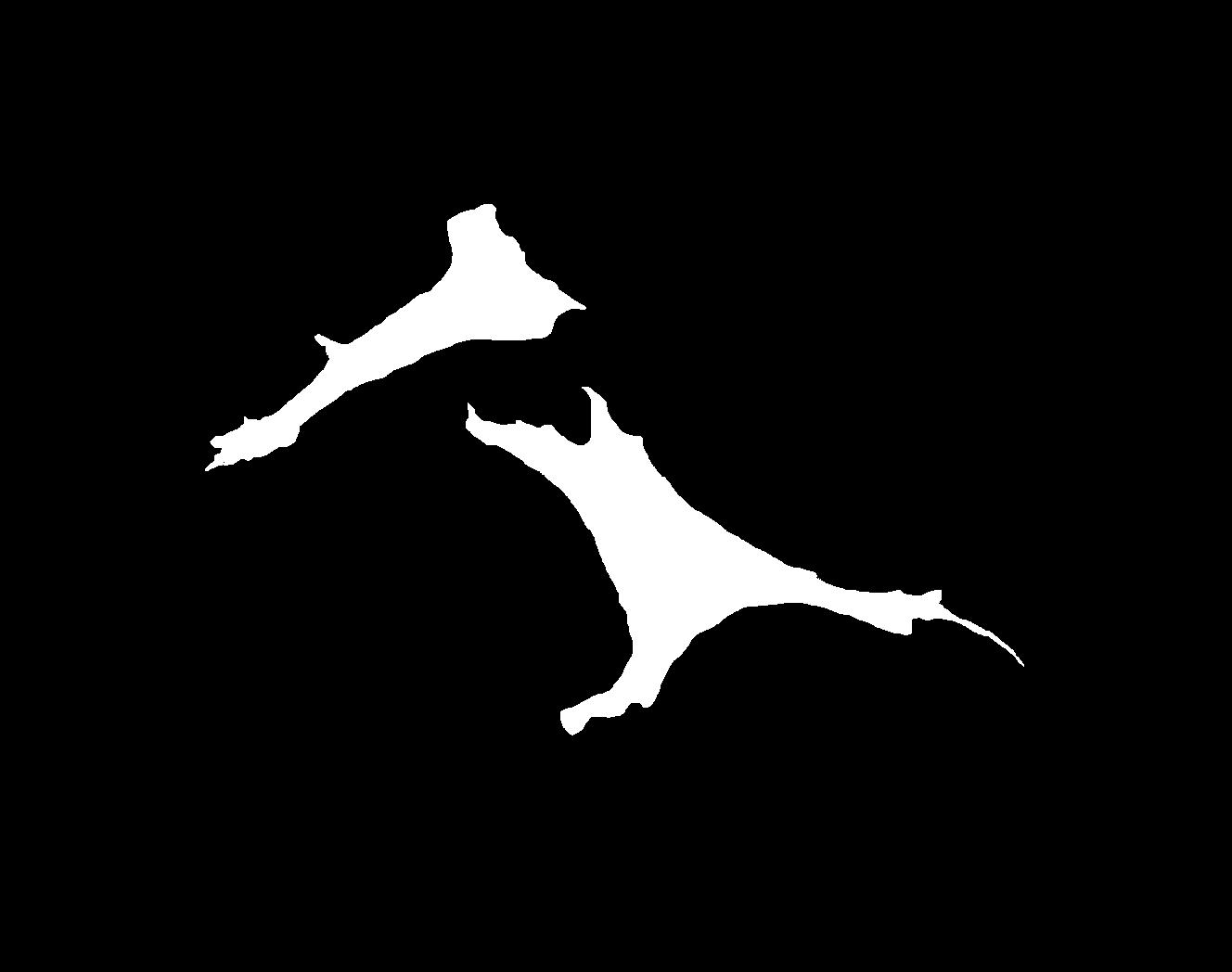

Supplement: S5 File — (ZIP) [file pone.0237972.s005.zip › S3_File IoU scores/masks/Experiment_1/cell/ground_truth/H_groundtruth.jpg]

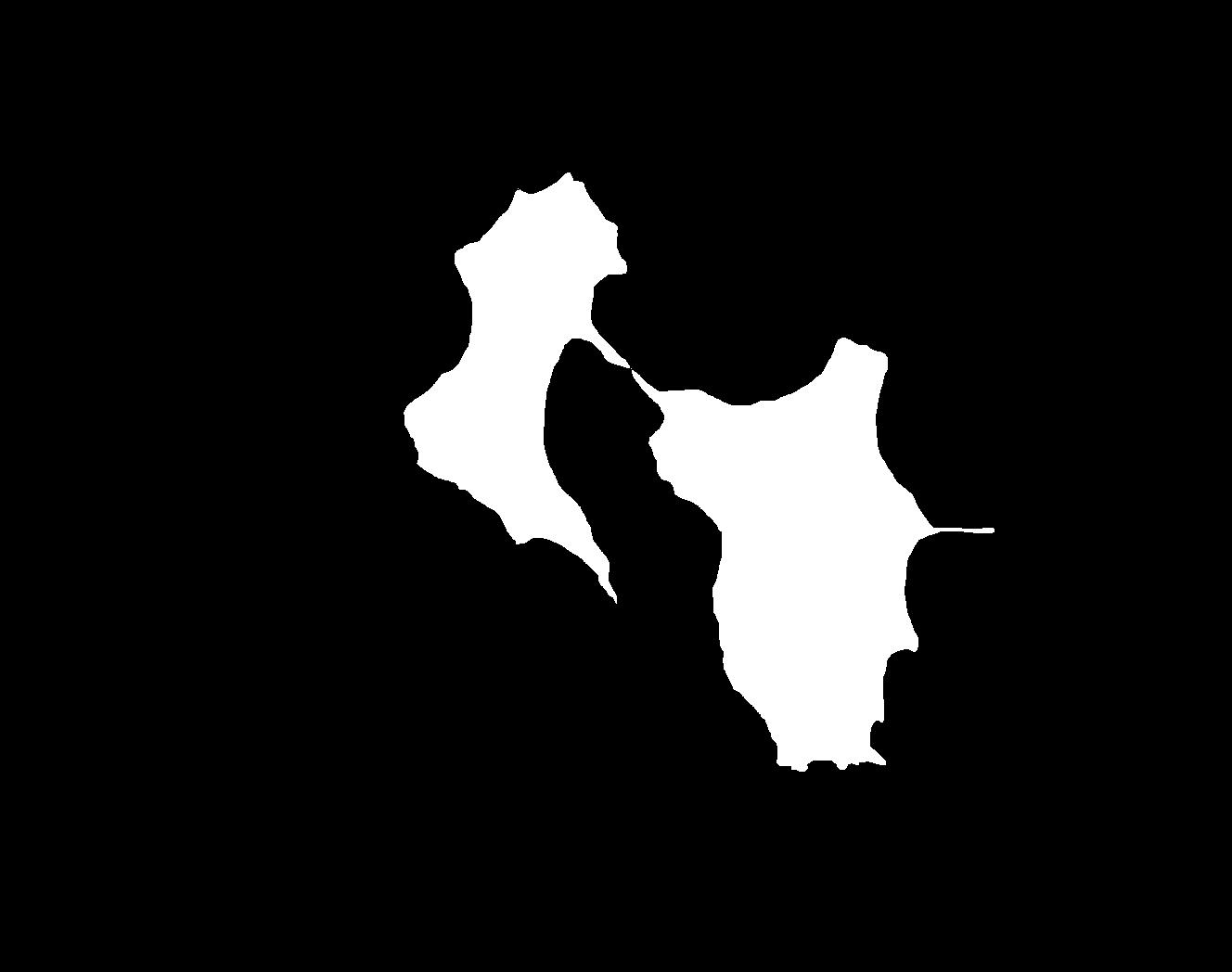

Supplement: S5 File — (ZIP) [file pone.0237972.s005.zip › S3_File IoU scores/masks/Experiment_1/cell/ground_truth/I_groundtruth.jpg]

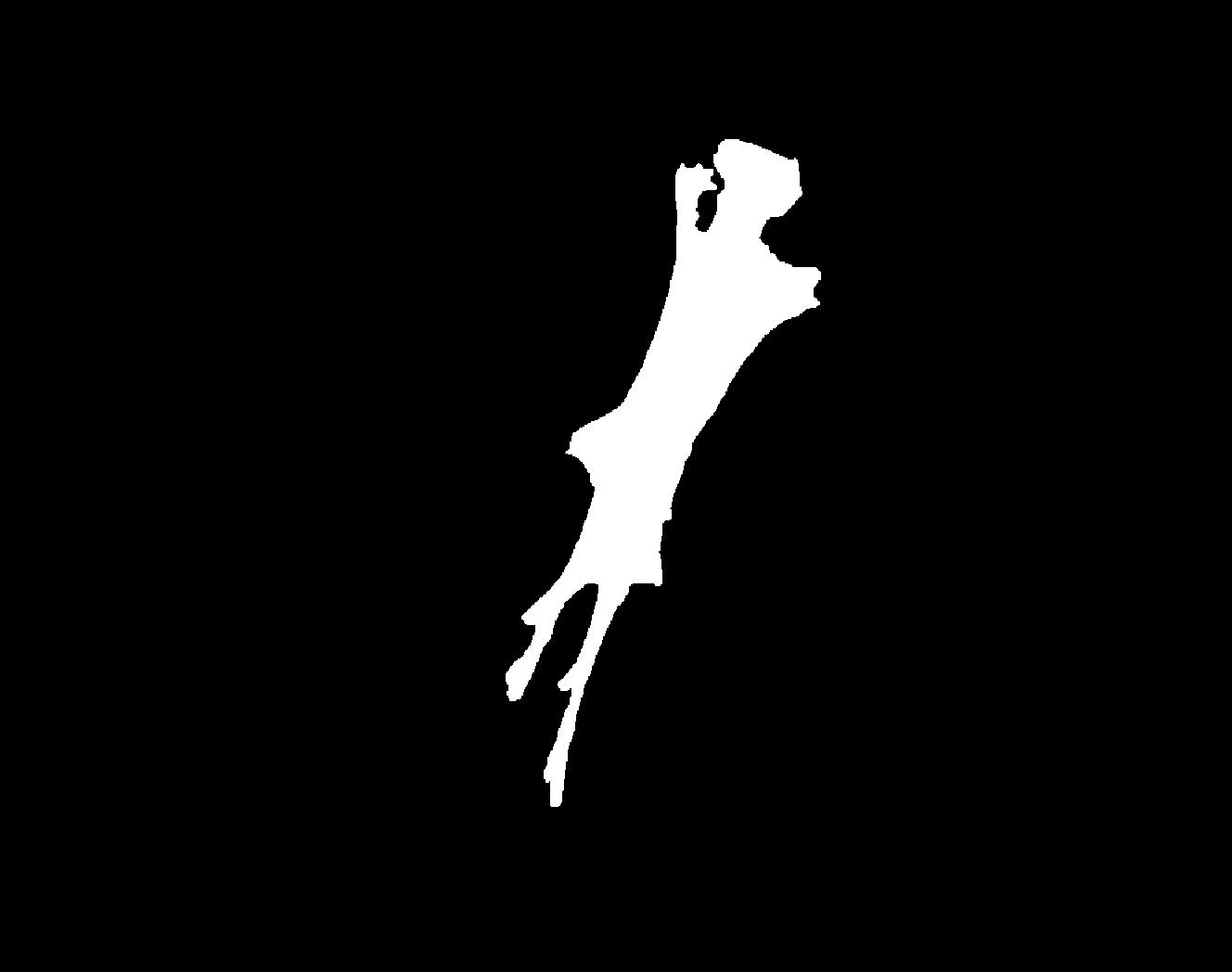

Supplement: S5 File — (ZIP) [file pone.0237972.s005.zip › S3_File IoU scores/masks/Experiment_1/cell/ground_truth/J_groundtruth.jpg]

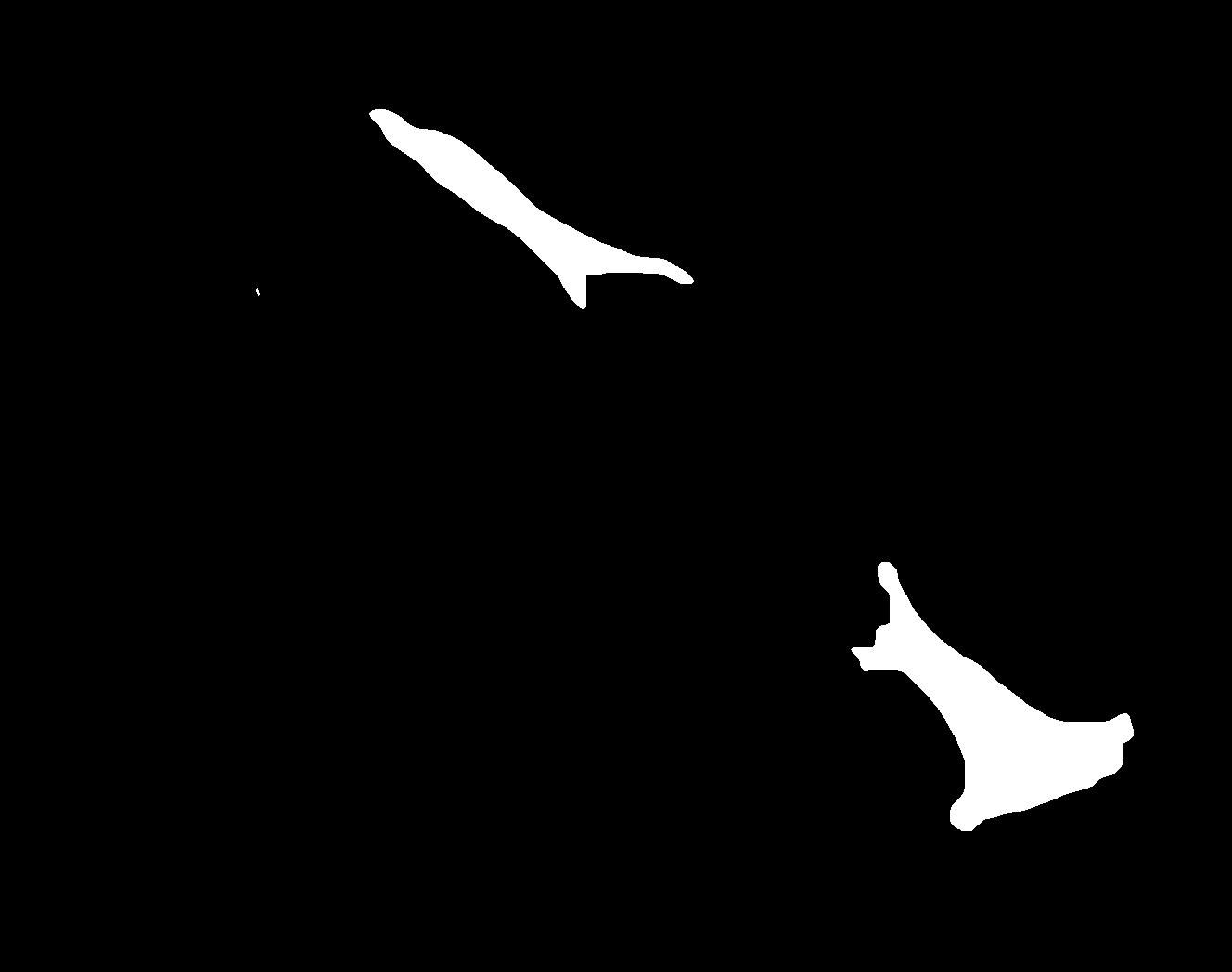

Supplement: S5 File — (ZIP) [file pone.0237972.s005.zip › S3_File IoU scores/masks/Experiment_1/cell/user_segmented/Automated/Automated_Participant10_mask_cell_A.jpg]

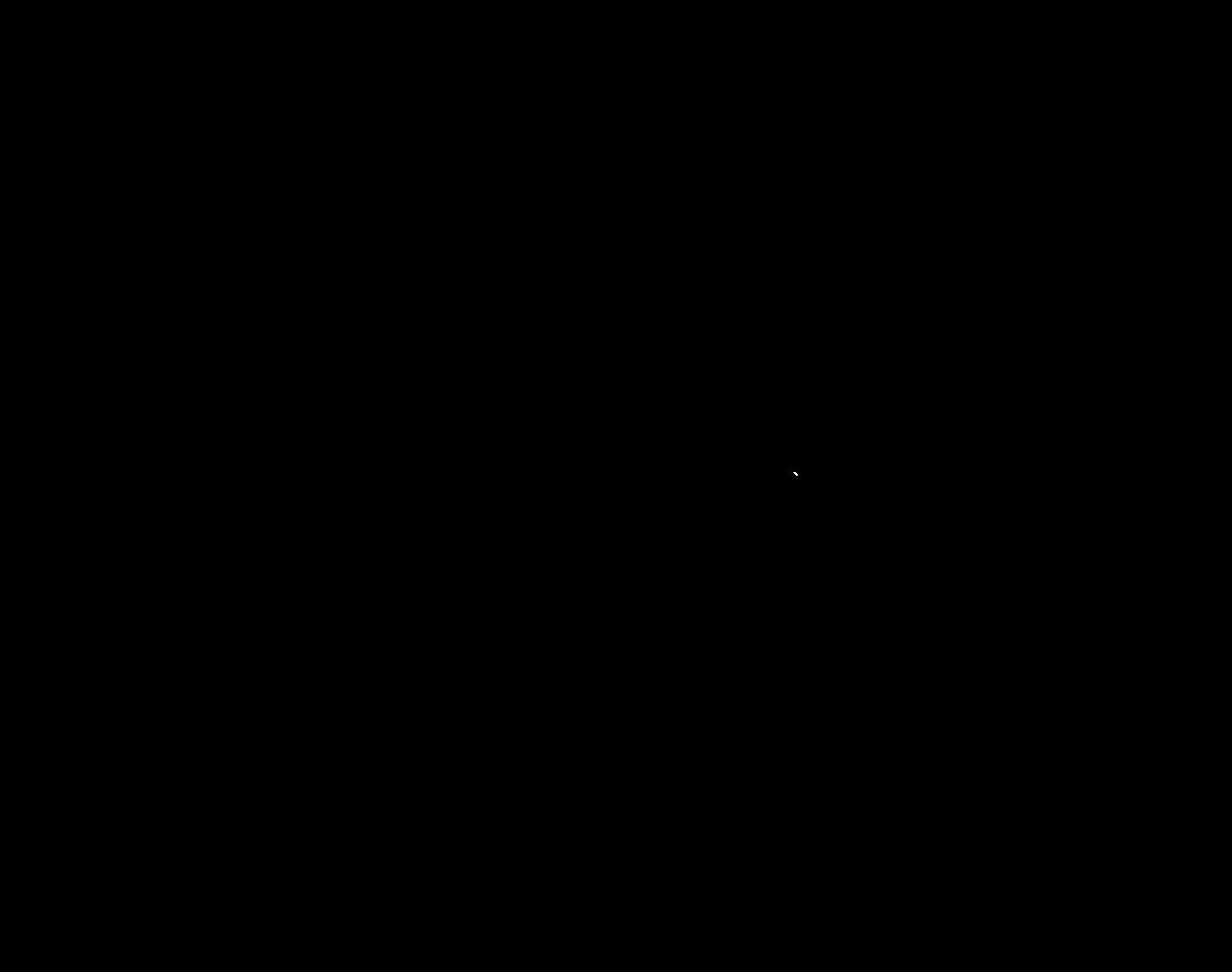

Supplement: S5 File — (ZIP) [file pone.0237972.s005.zip › S3_File IoU scores/masks/Experiment_1/cell/user_segmented/Automated/Automated_Participant10_mask_cell_B.jpg]

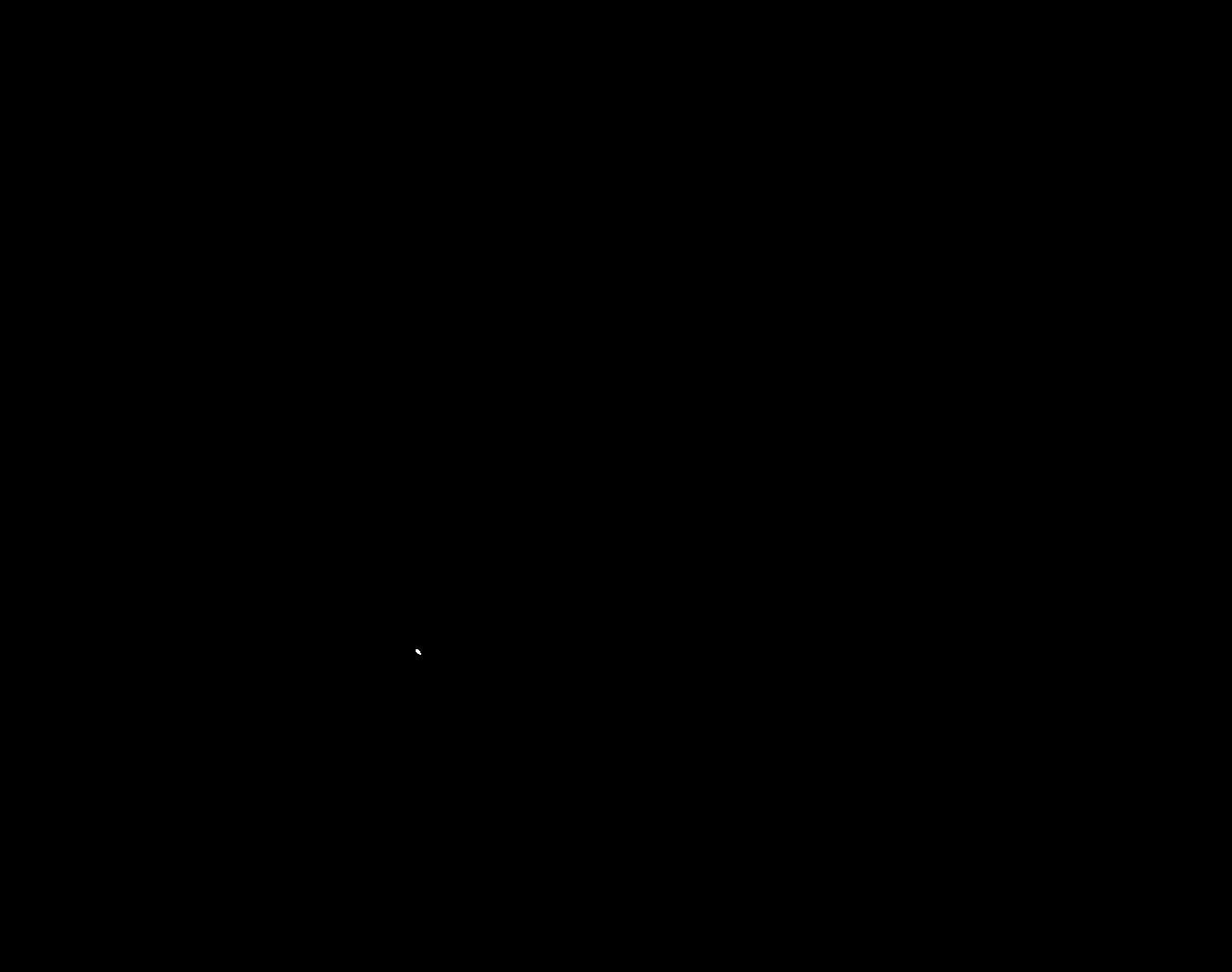

Supplement: S5 File — (ZIP) [file pone.0237972.s005.zip › S3_File IoU scores/masks/Experiment_1/cell/user_segmented/Automated/Automated_Participant10_mask_cell_C.jpg]

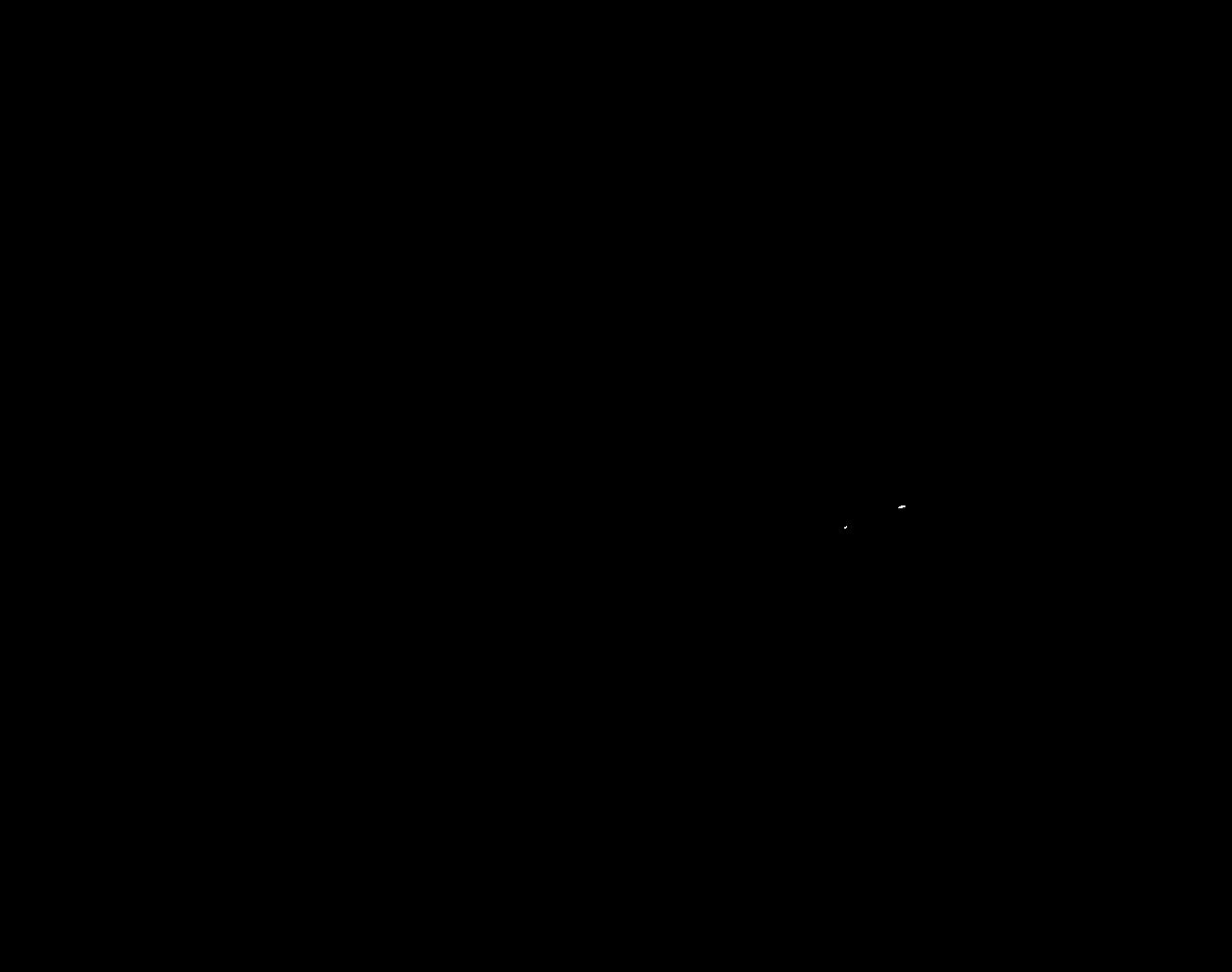

Supplement: S5 File — (ZIP) [file pone.0237972.s005.zip › S3_File IoU scores/masks/Experiment_1/cell/user_segmented/Automated/Automated_Participant10_mask_cell_D.jpg]

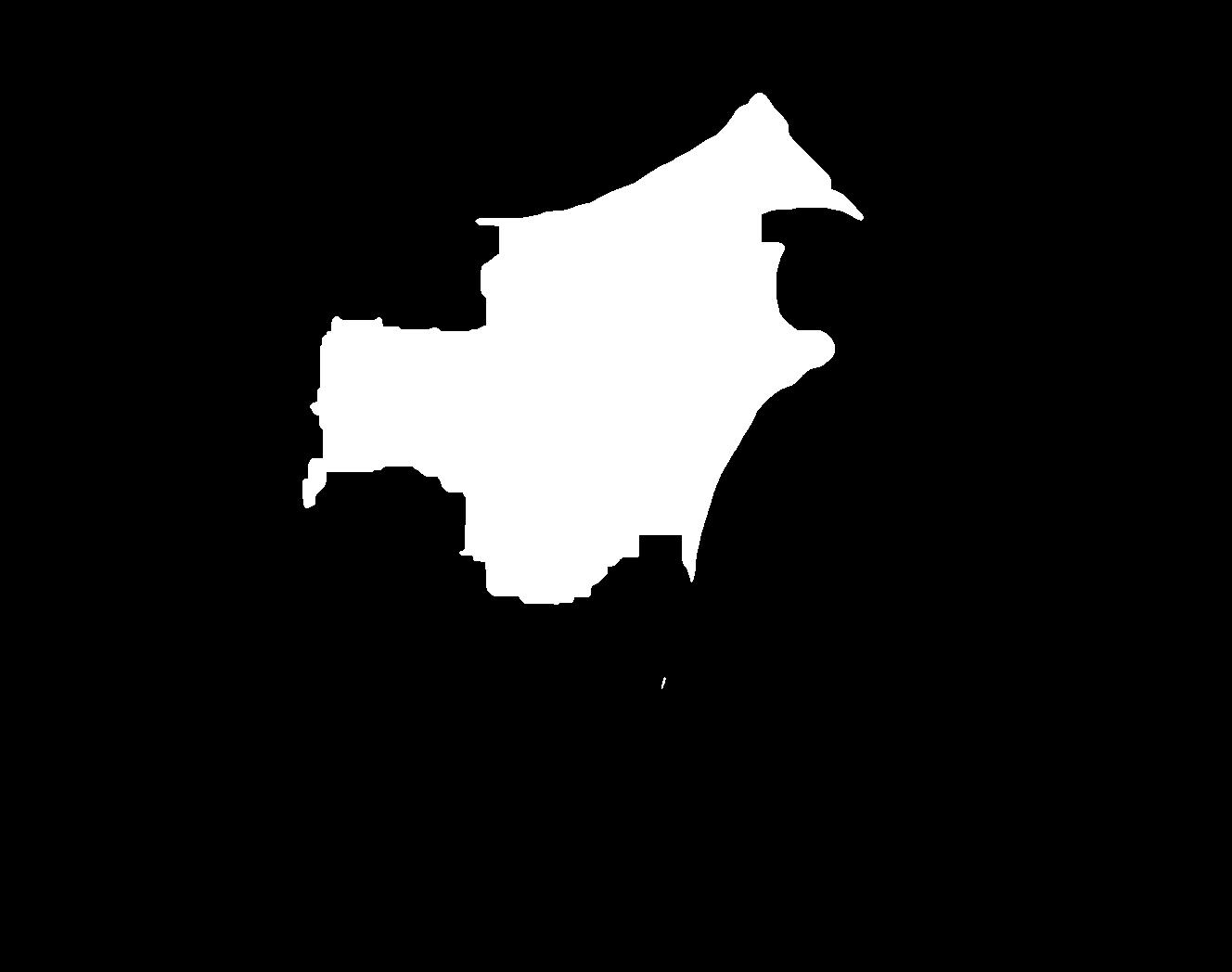

Supplement: S5 File — (ZIP) [file pone.0237972.s005.zip › S3_File IoU scores/masks/Experiment_1/cell/user_segmented/Automated/Automated_Participant10_mask_cell_E.jpg]

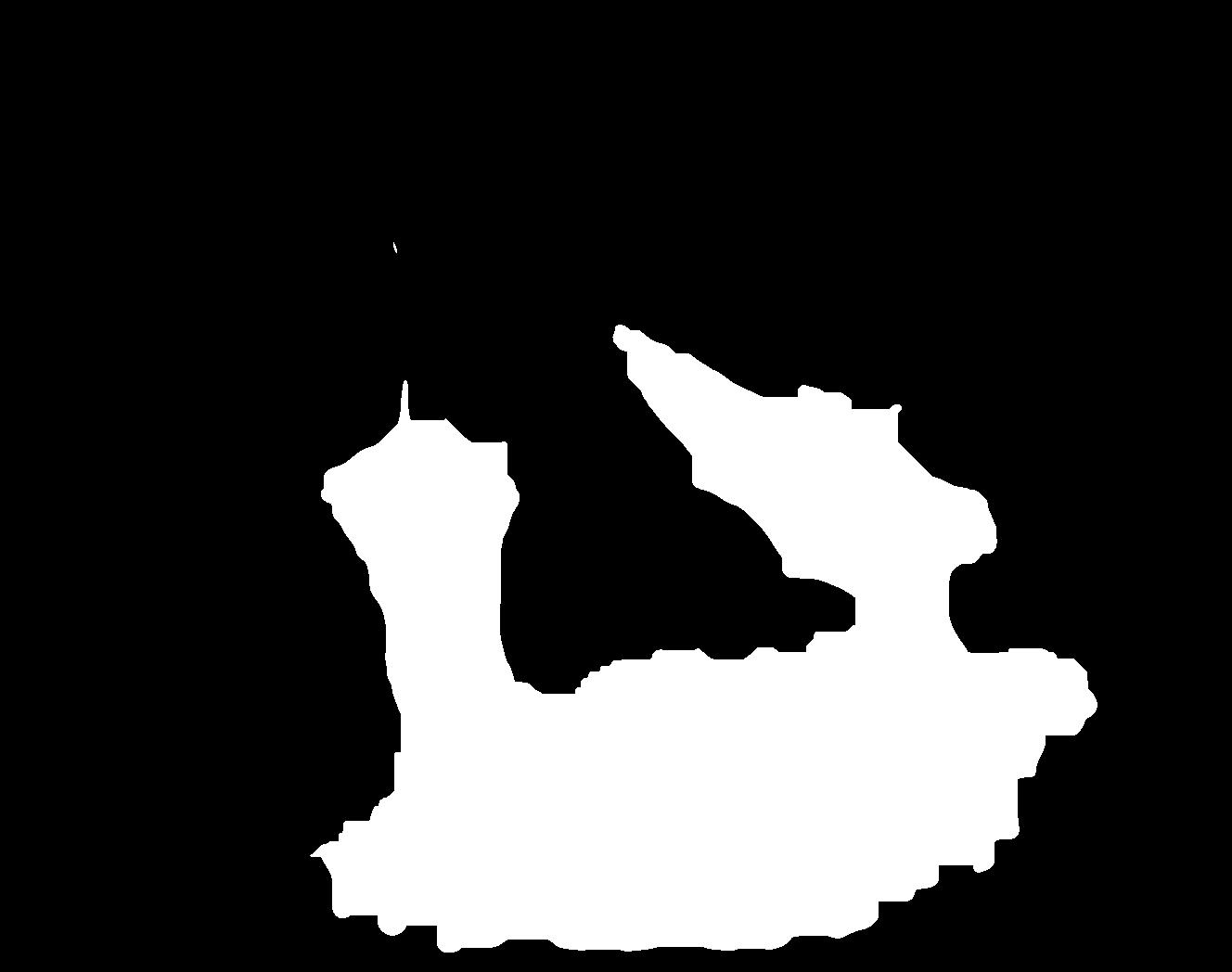

Supplement: S5 File — (ZIP) [file pone.0237972.s005.zip › S3_File IoU scores/masks/Experiment_1/cell/user_segmented/Automated/Automated_Participant10_mask_cell_F.jpg]

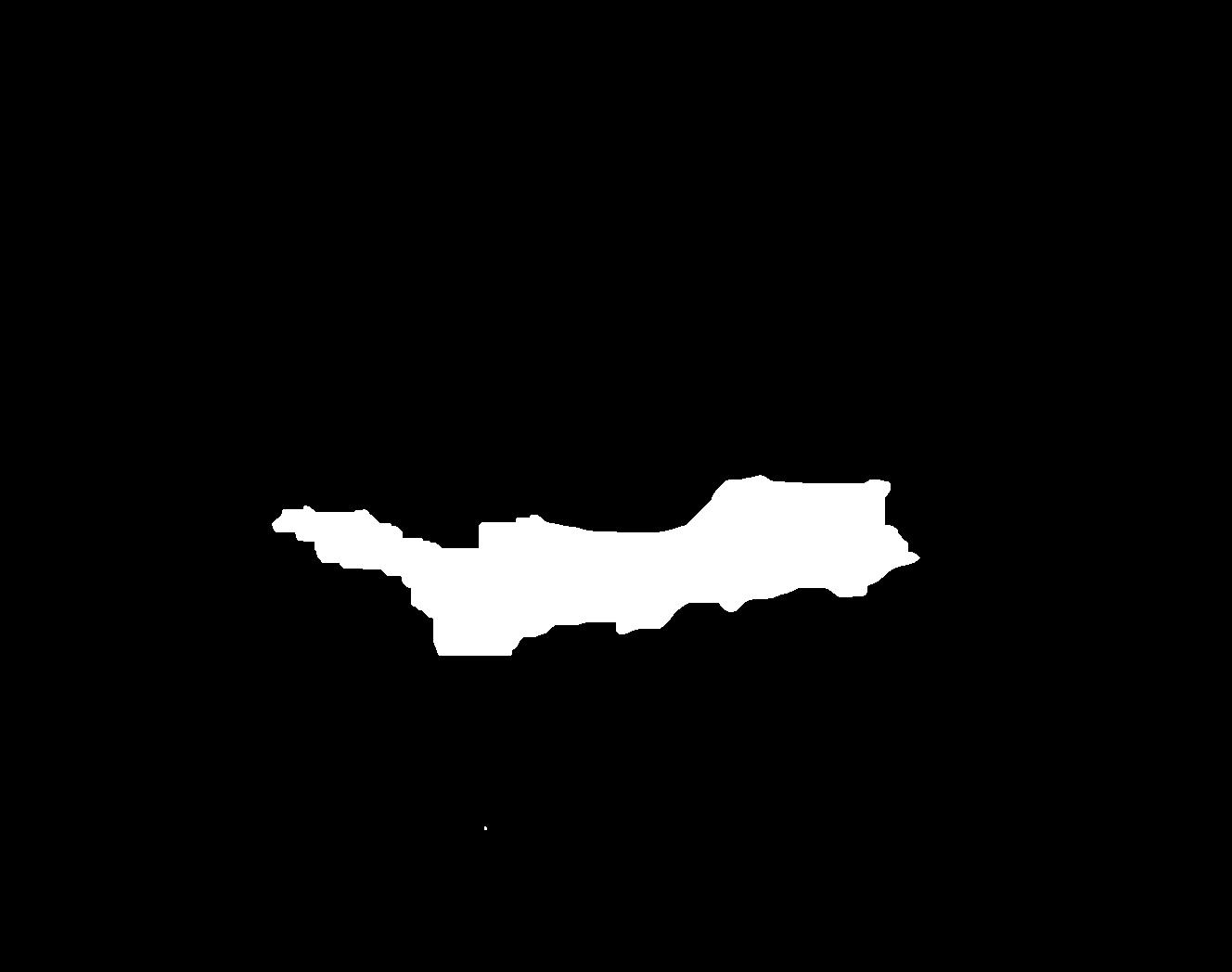

Supplement: S5 File — (ZIP) [file pone.0237972.s005.zip › S3_File IoU scores/masks/Experiment_1/cell/user_segmented/Automated/Automated_Participant10_mask_cell_G.jpg]

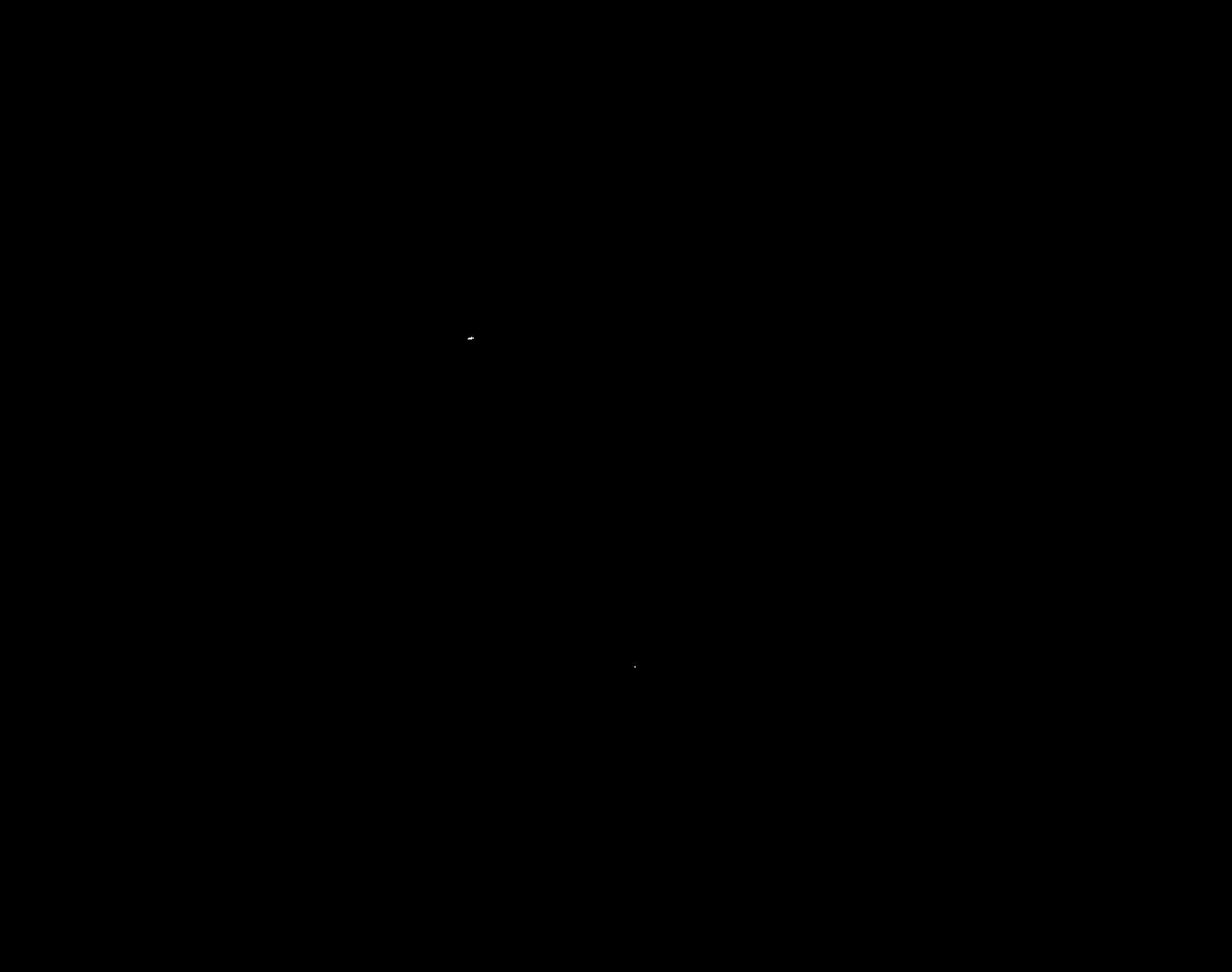

Supplement: S5 File — (ZIP) [file pone.0237972.s005.zip › S3_File IoU scores/masks/Experiment_1/cell/user_segmented/Automated/Automated_Participant10_mask_cell_H.jpg]

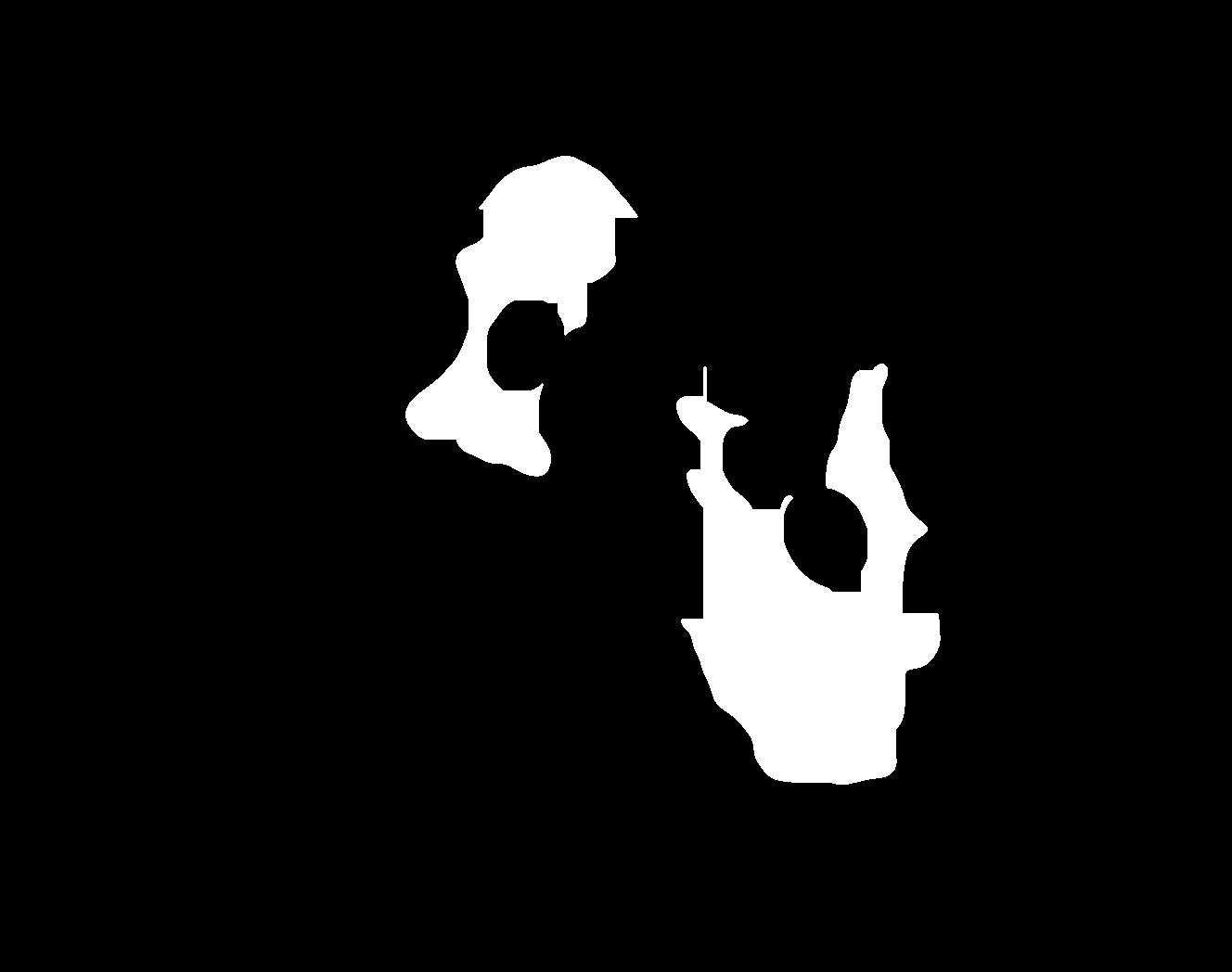

Supplement: S5 File — (ZIP) [file pone.0237972.s005.zip › S3_File IoU scores/masks/Experiment_1/cell/user_segmented/Automated/Automated_Participant10_mask_cell_I.jpg]

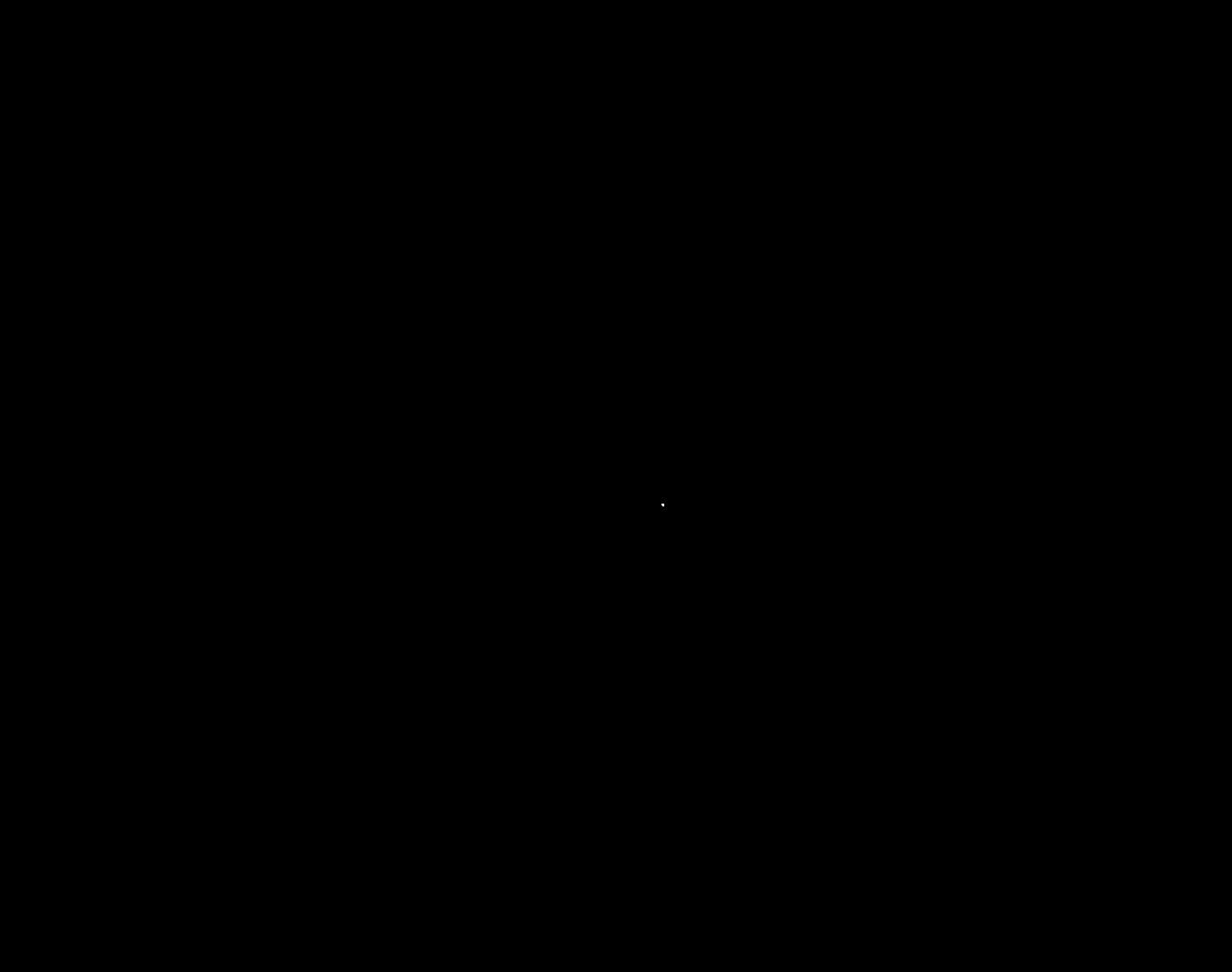

Supplement: S5 File — (ZIP) [file pone.0237972.s005.zip › S3_File IoU scores/masks/Experiment_1/cell/user_segmented/Automated/Automated_Participant10_mask_cell_J.jpg]

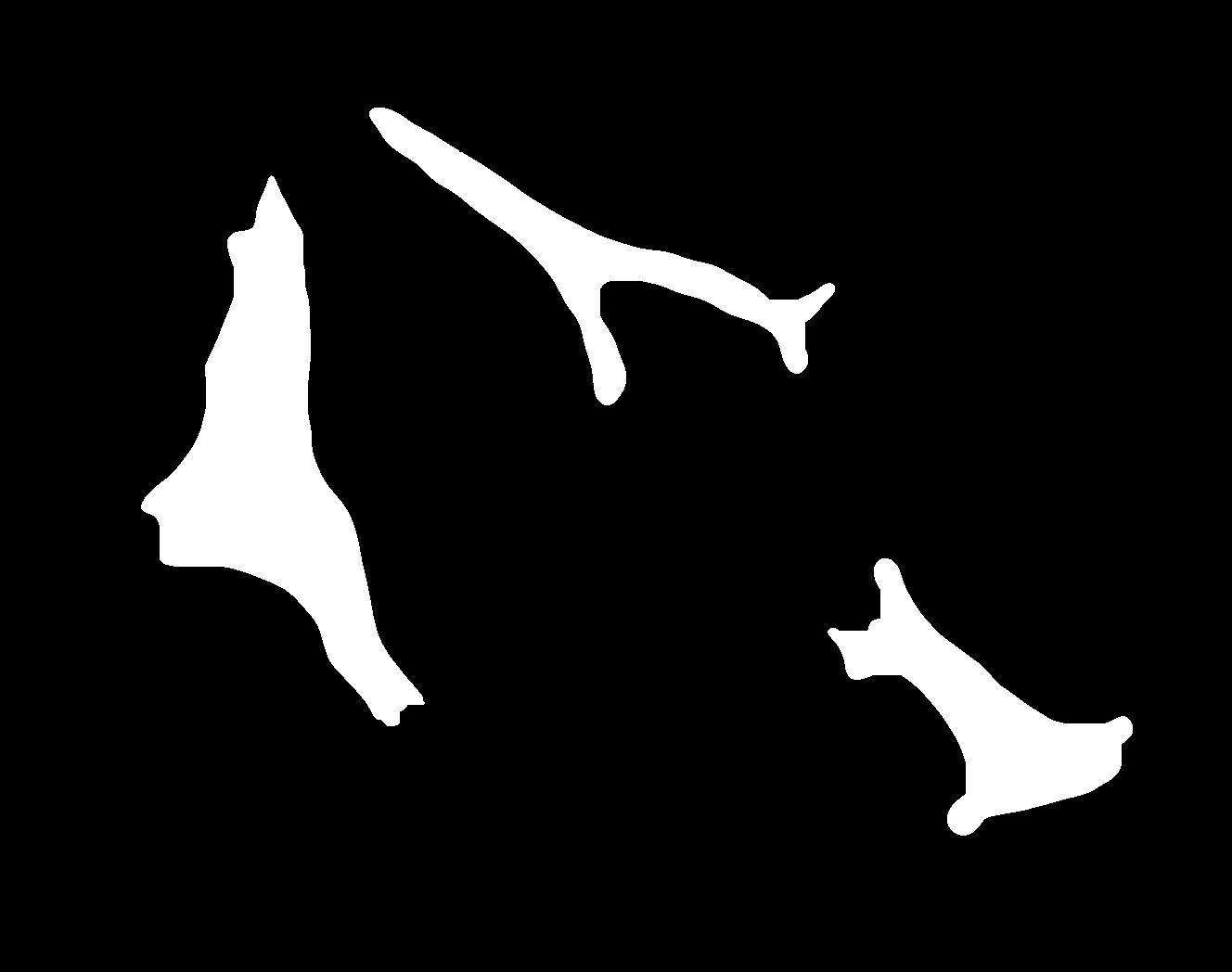

Supplement: S5 File — (ZIP) [file pone.0237972.s005.zip › S3_File IoU scores/masks/Experiment_1/cell/user_segmented/Automated/Automated_Participant13_mask_cell_A.jpg]

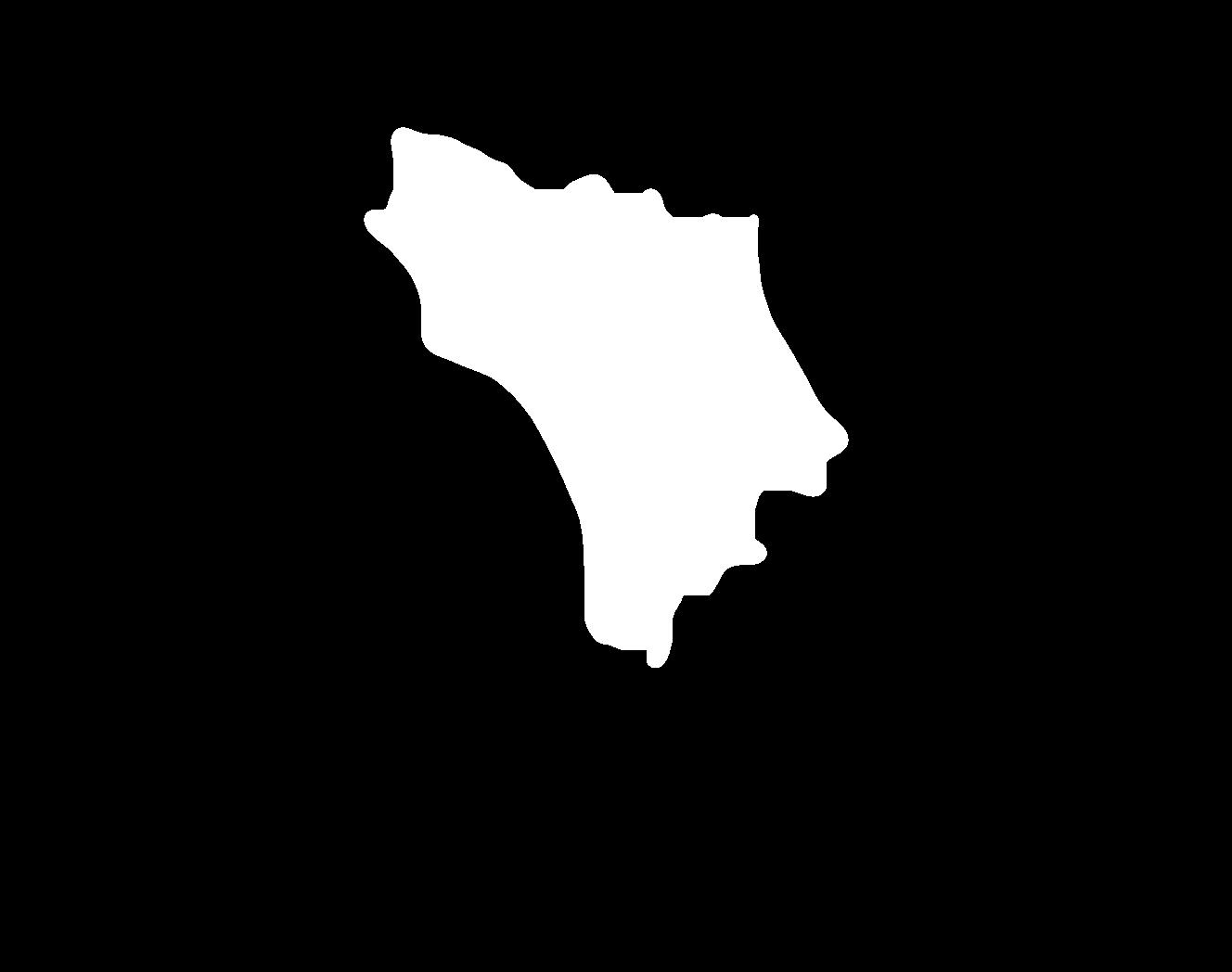

Supplement: S5 File — (ZIP) [file pone.0237972.s005.zip › S3_File IoU scores/masks/Experiment_1/cell/user_segmented/Automated/Automated_Participant13_mask_cell_B.jpg]

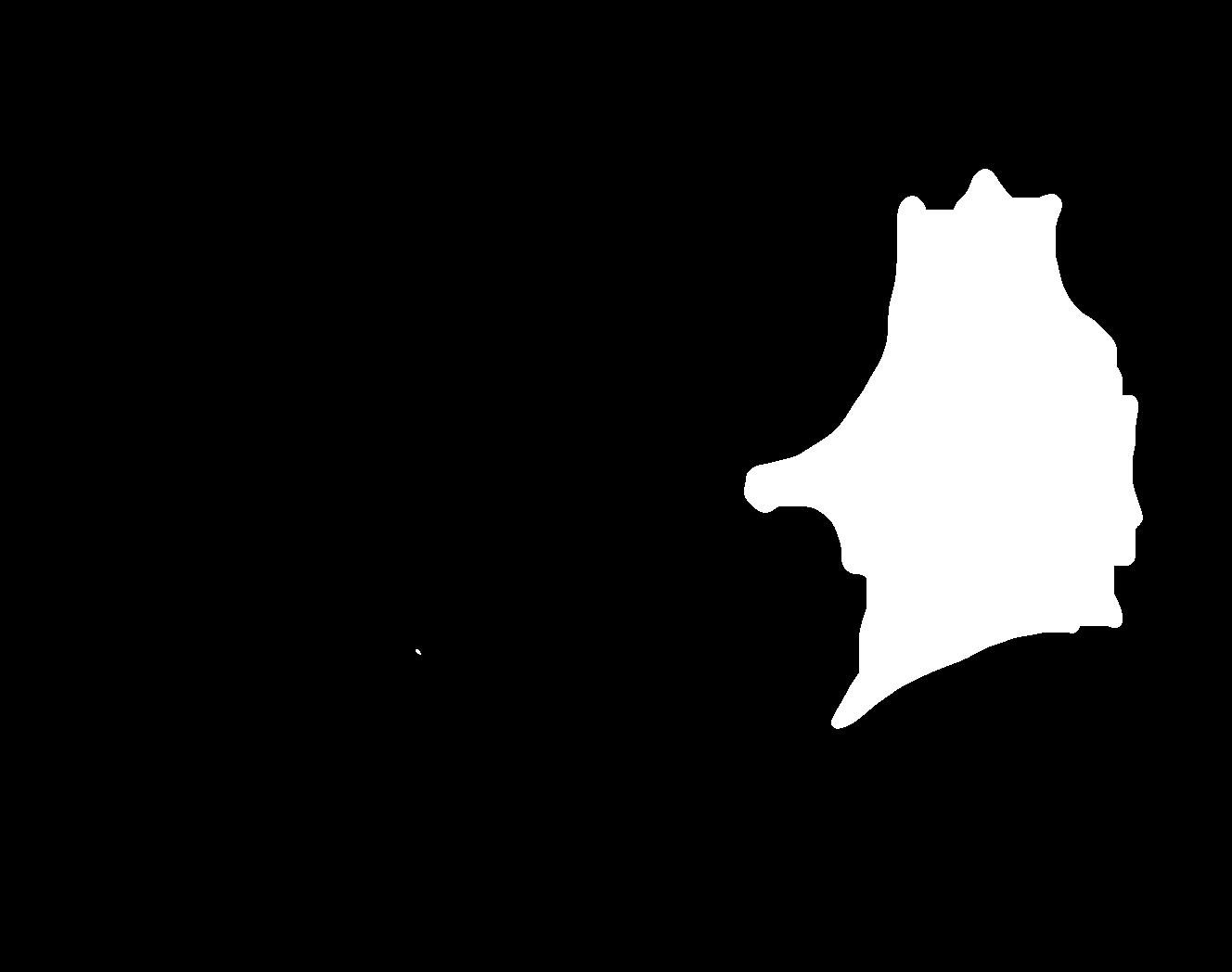

Supplement: S5 File — (ZIP) [file pone.0237972.s005.zip › S3_File IoU scores/masks/Experiment_1/cell/user_segmented/Automated/Automated_Participant13_mask_cell_C.jpg]

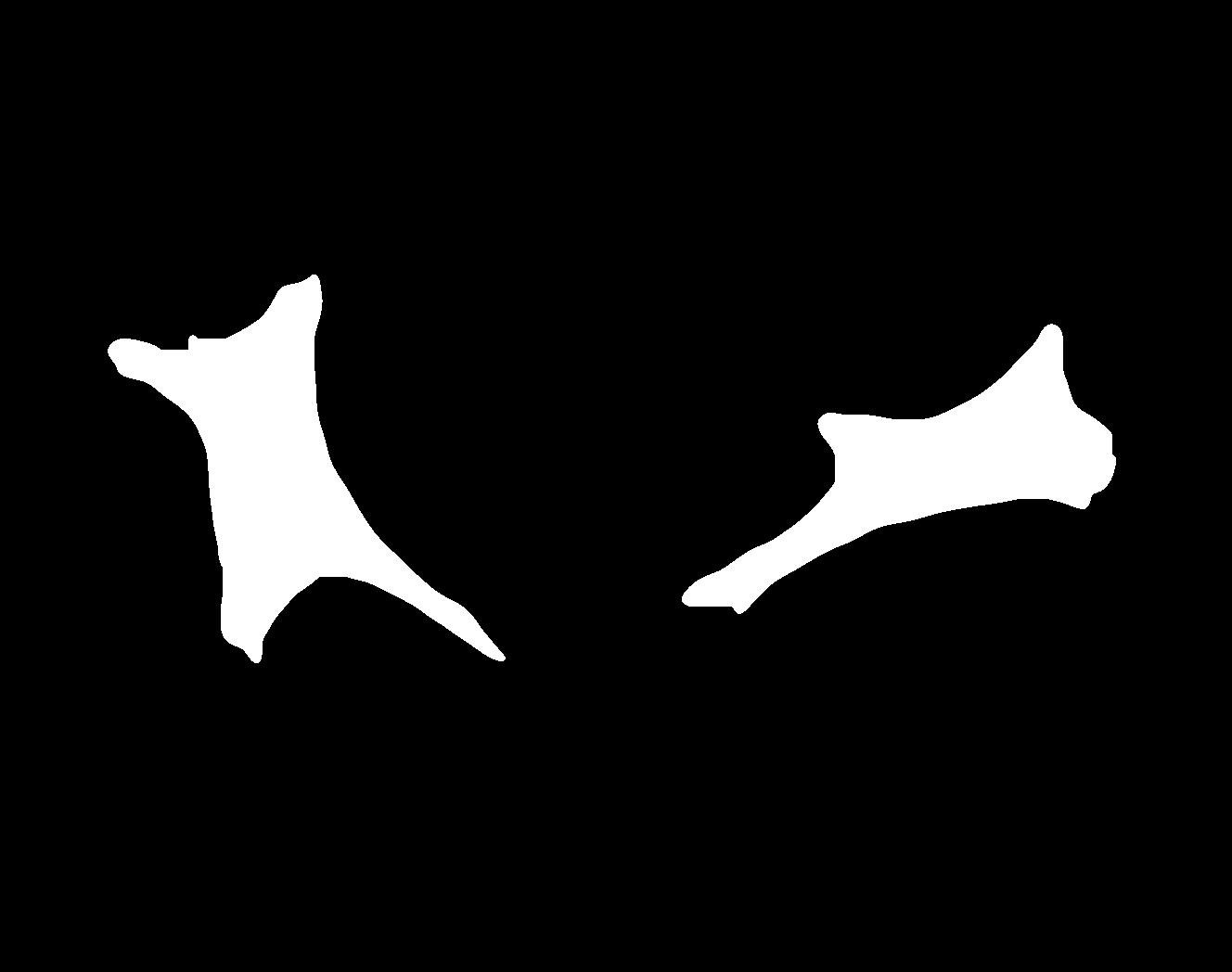

Supplement: S5 File — (ZIP) [file pone.0237972.s005.zip › S3_File IoU scores/masks/Experiment_1/cell/user_segmented/Automated/Automated_Participant13_mask_cell_D.jpg]

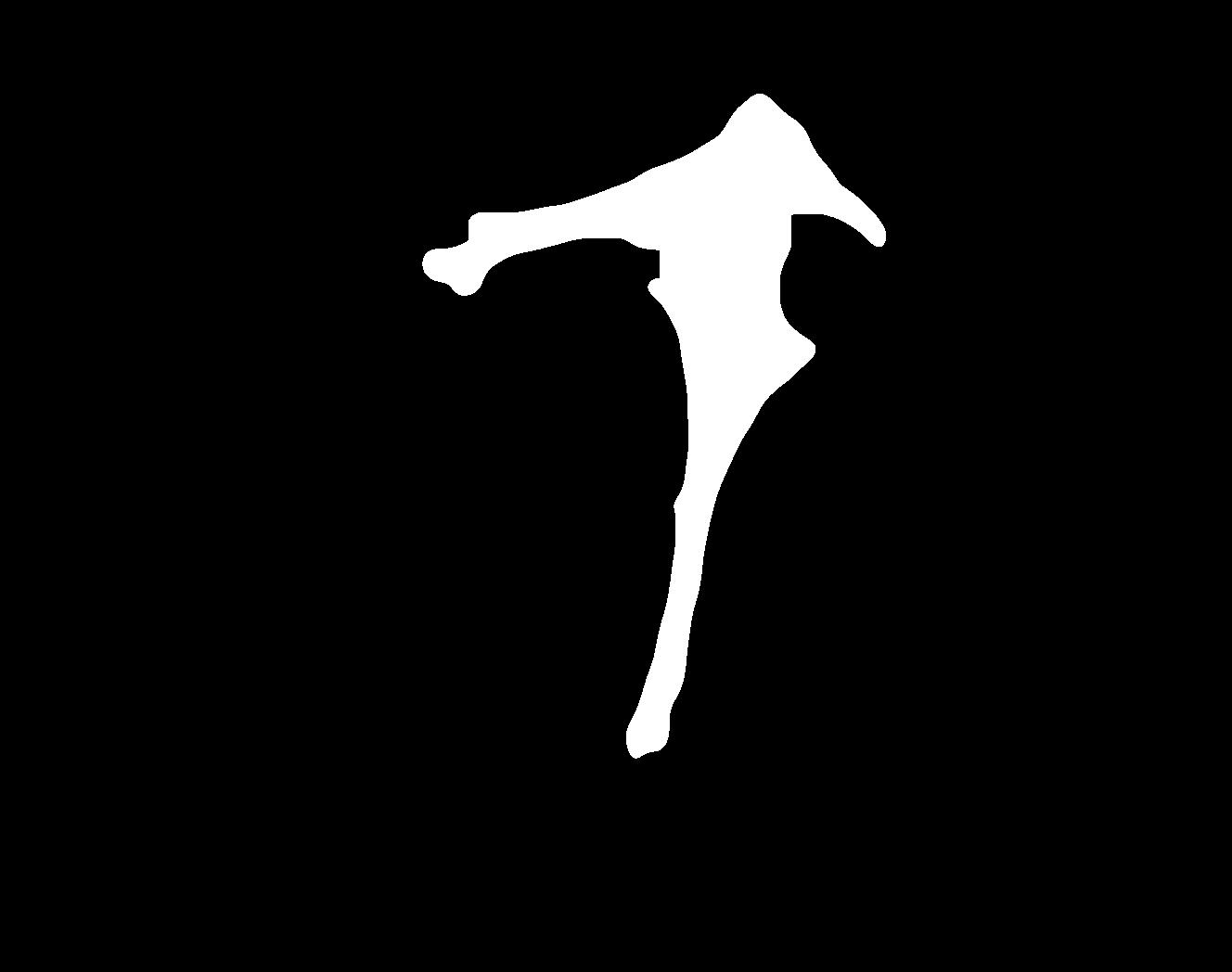

Supplement: S5 File — (ZIP) [file pone.0237972.s005.zip › S3_File IoU scores/masks/Experiment_1/cell/user_segmented/Automated/Automated_Participant13_mask_cell_E.jpg]

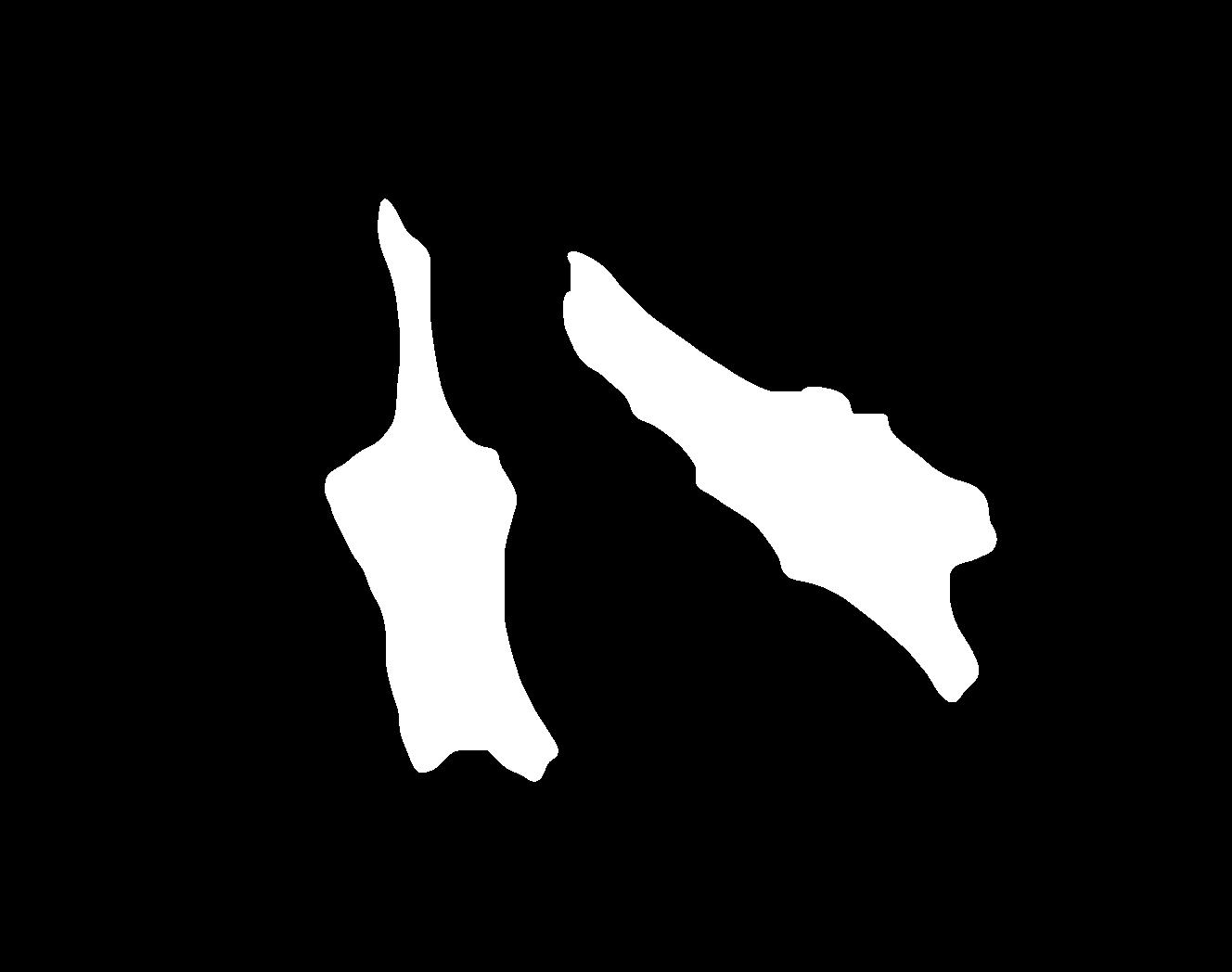

Supplement: S5 File — (ZIP) [file pone.0237972.s005.zip › S3_File IoU scores/masks/Experiment_1/cell/user_segmented/Automated/Automated_Participant13_mask_cell_F.jpg]

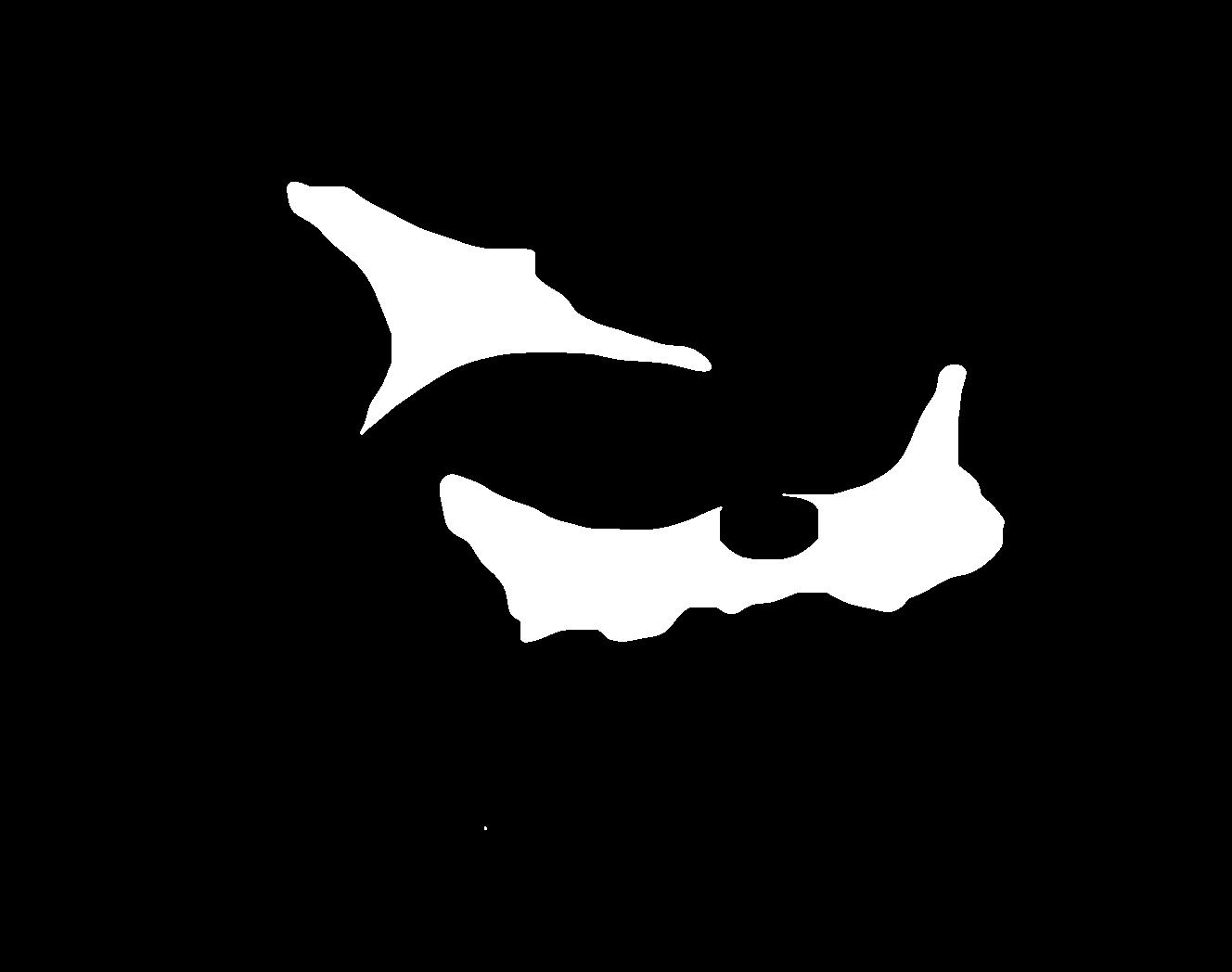

Supplement: S5 File — (ZIP) [file pone.0237972.s005.zip › S3_File IoU scores/masks/Experiment_1/cell/user_segmented/Automated/Automated_Participant13_mask_cell_G.jpg]

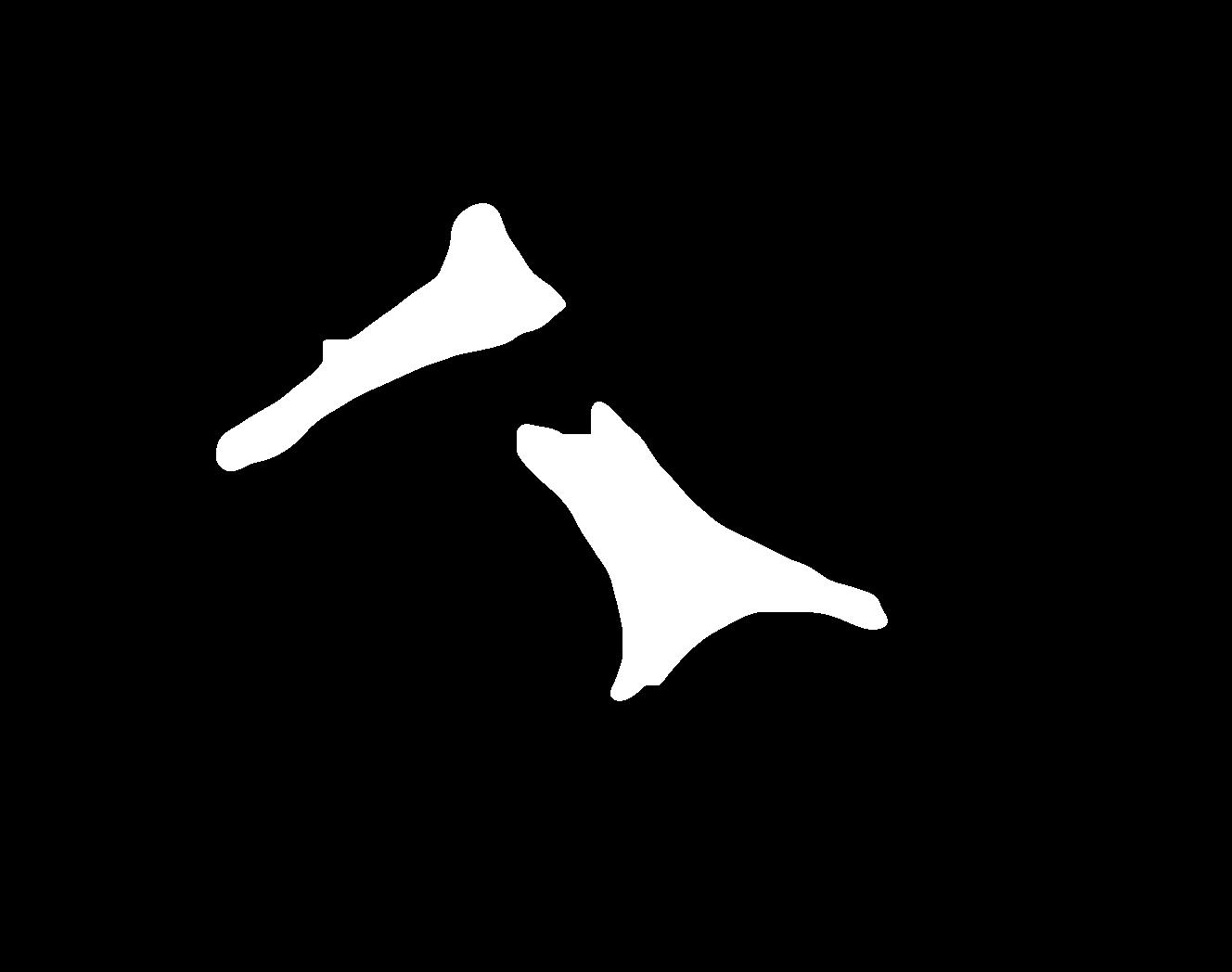

Supplement: S5 File — (ZIP) [file pone.0237972.s005.zip › S3_File IoU scores/masks/Experiment_1/cell/user_segmented/Automated/Automated_Participant13_mask_cell_H.jpg]

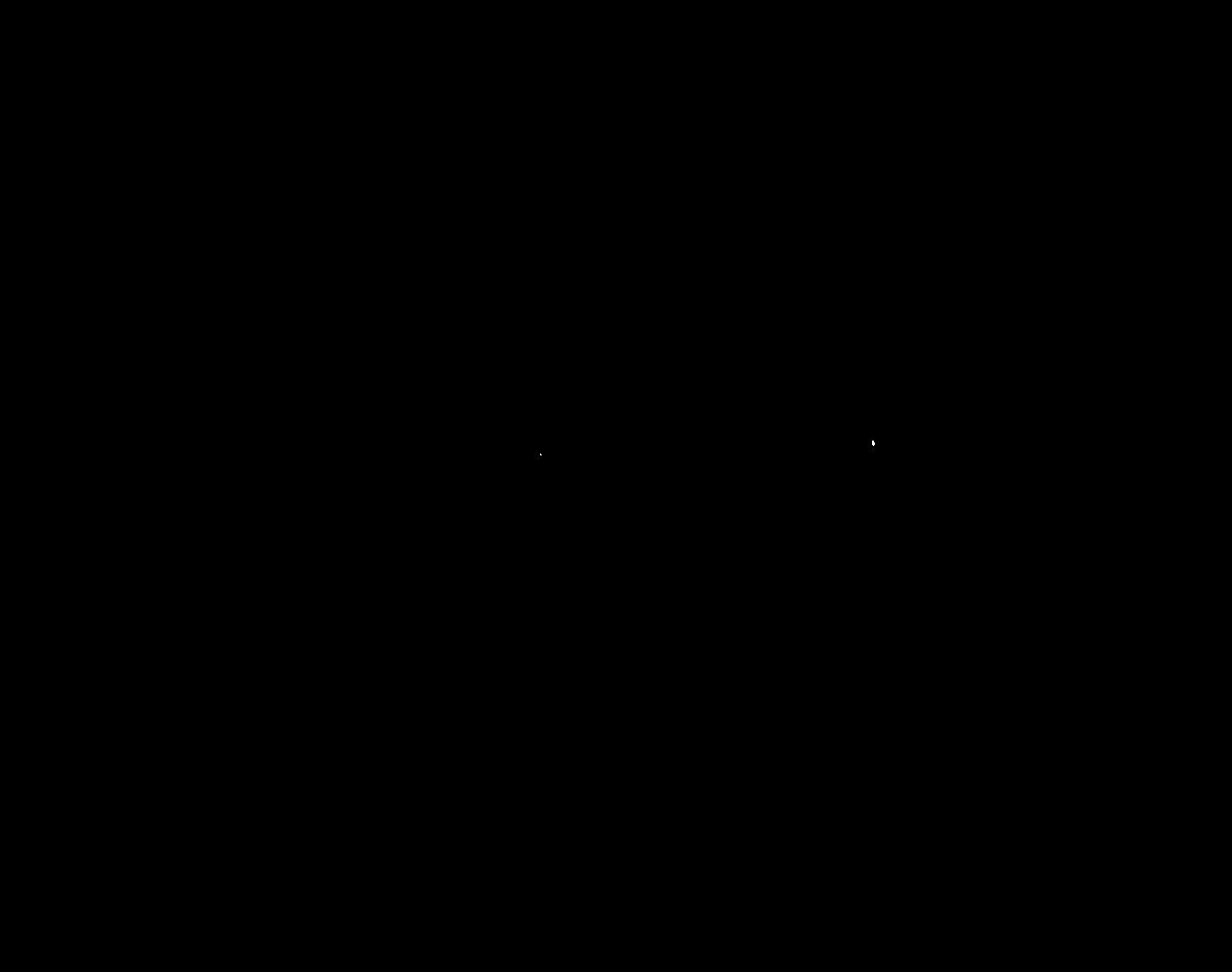

Supplement: S5 File — (ZIP) [file pone.0237972.s005.zip › S3_File IoU scores/masks/Experiment_1/cell/user_segmented/Automated/Automated_Participant13_mask_cell_I.jpg]

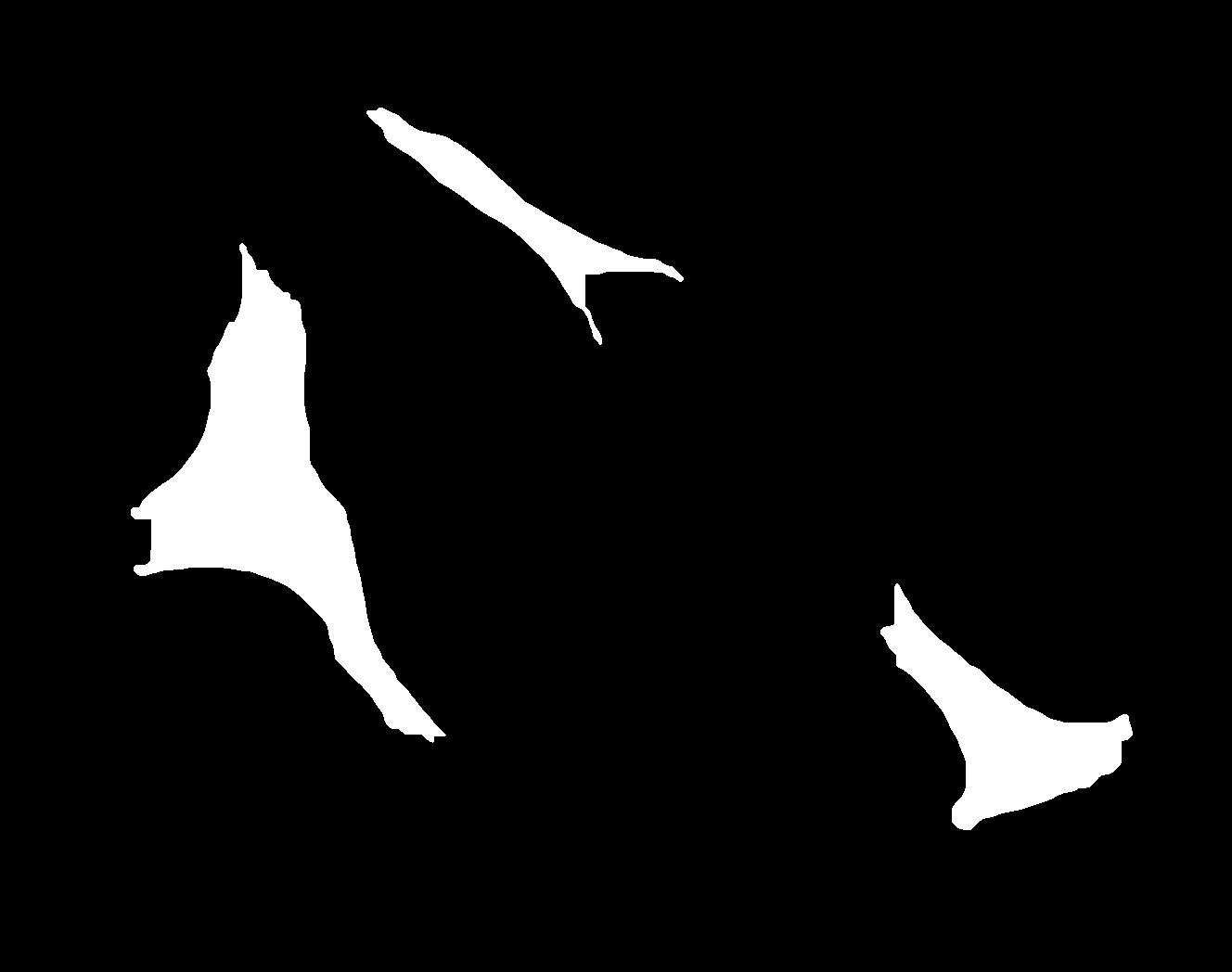

Supplement: S5 File — (ZIP) [file pone.0237972.s005.zip › S3_File IoU scores/masks/Experiment_1/cell/user_segmented/Automated/Automated_Participant15_mask_cell_A.jpg]

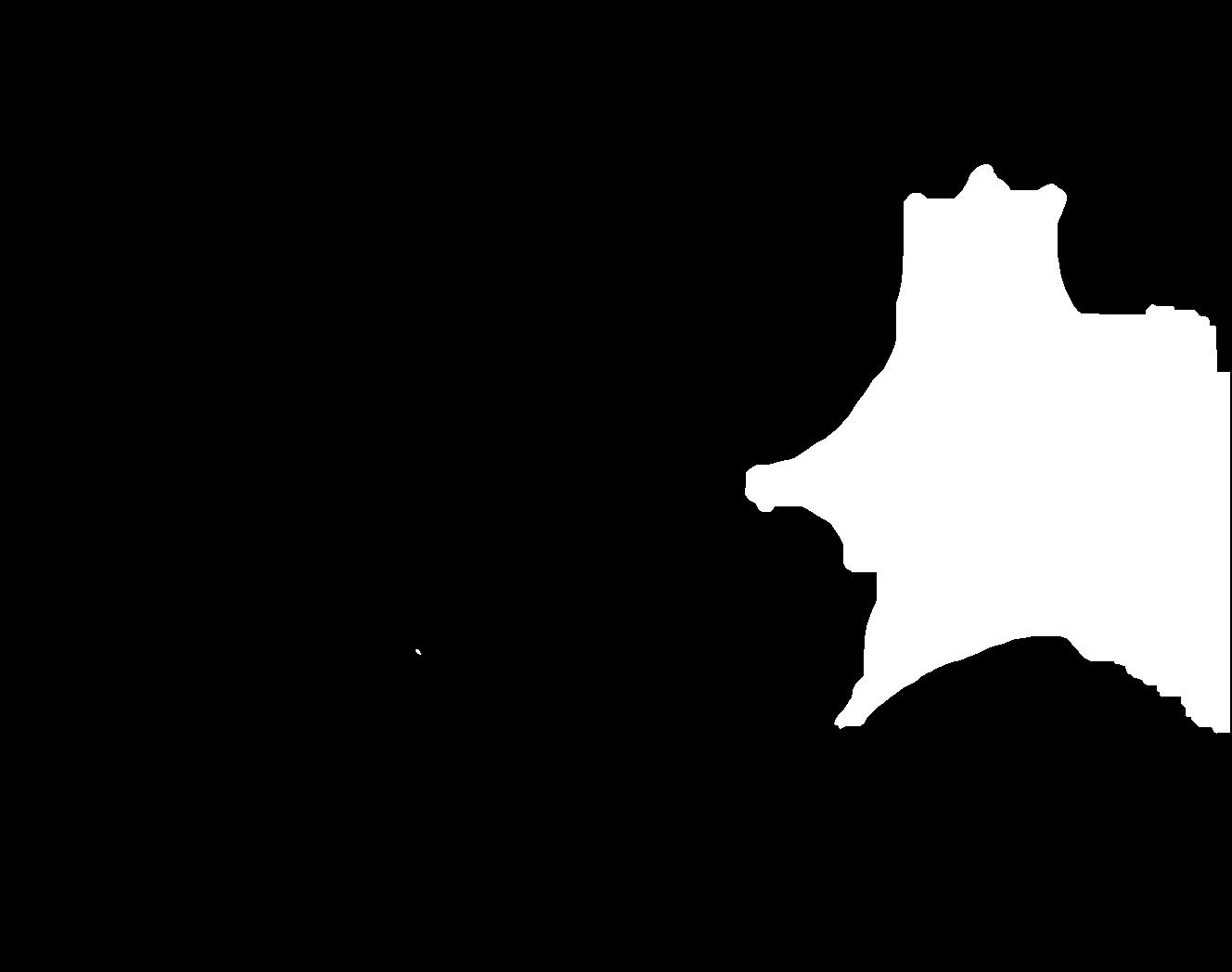

Supplement: S5 File — (ZIP) [file pone.0237972.s005.zip › S3_File IoU scores/masks/Experiment_1/cell/user_segmented/Automated/Automated_Participant15_mask_cell_C.jpg]

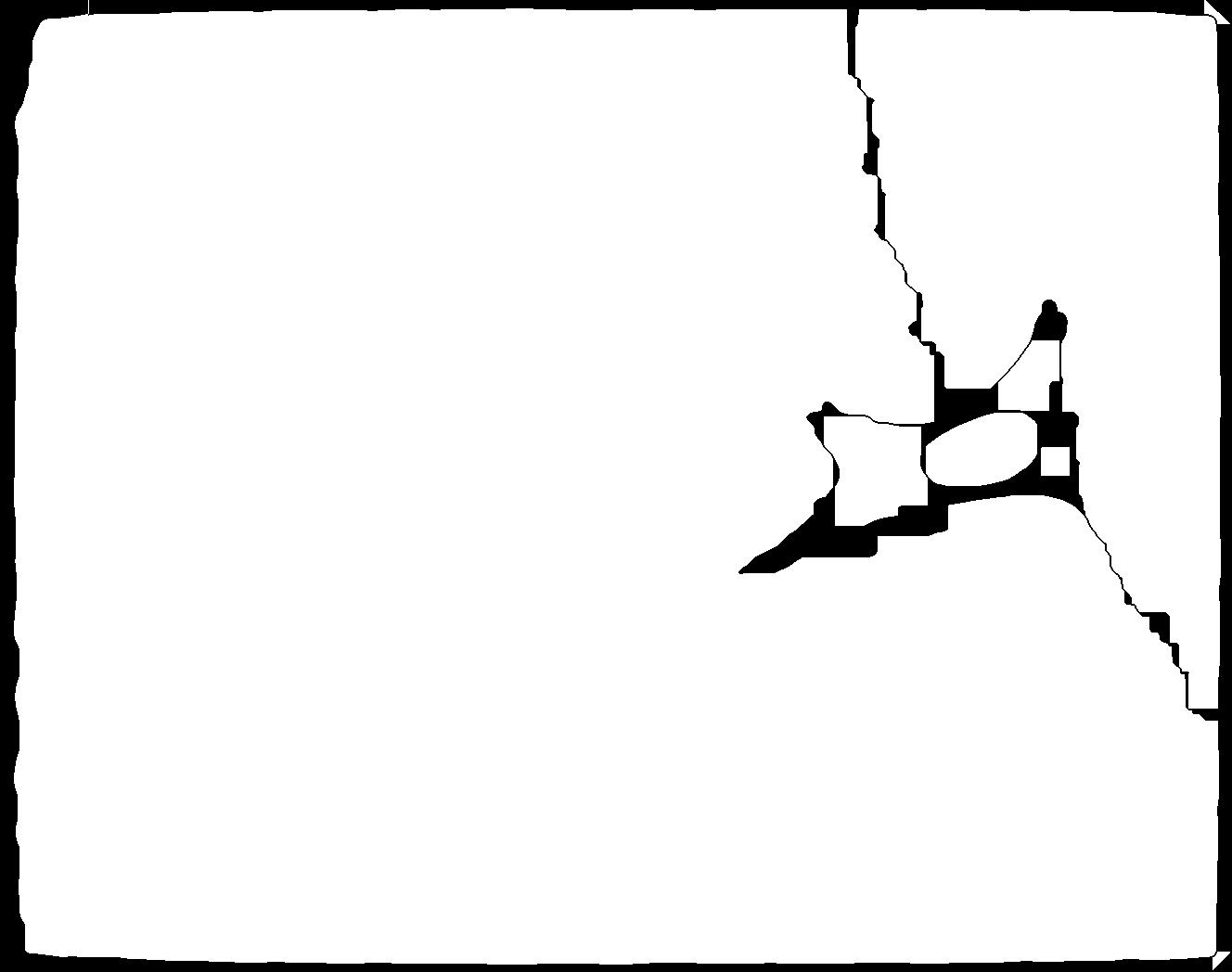

Supplement: S5 File — (ZIP) [file pone.0237972.s005.zip › S3_File IoU scores/masks/Experiment_1/cell/user_segmented/Automated/Automated_Participant15_mask_cell_D.jpg]

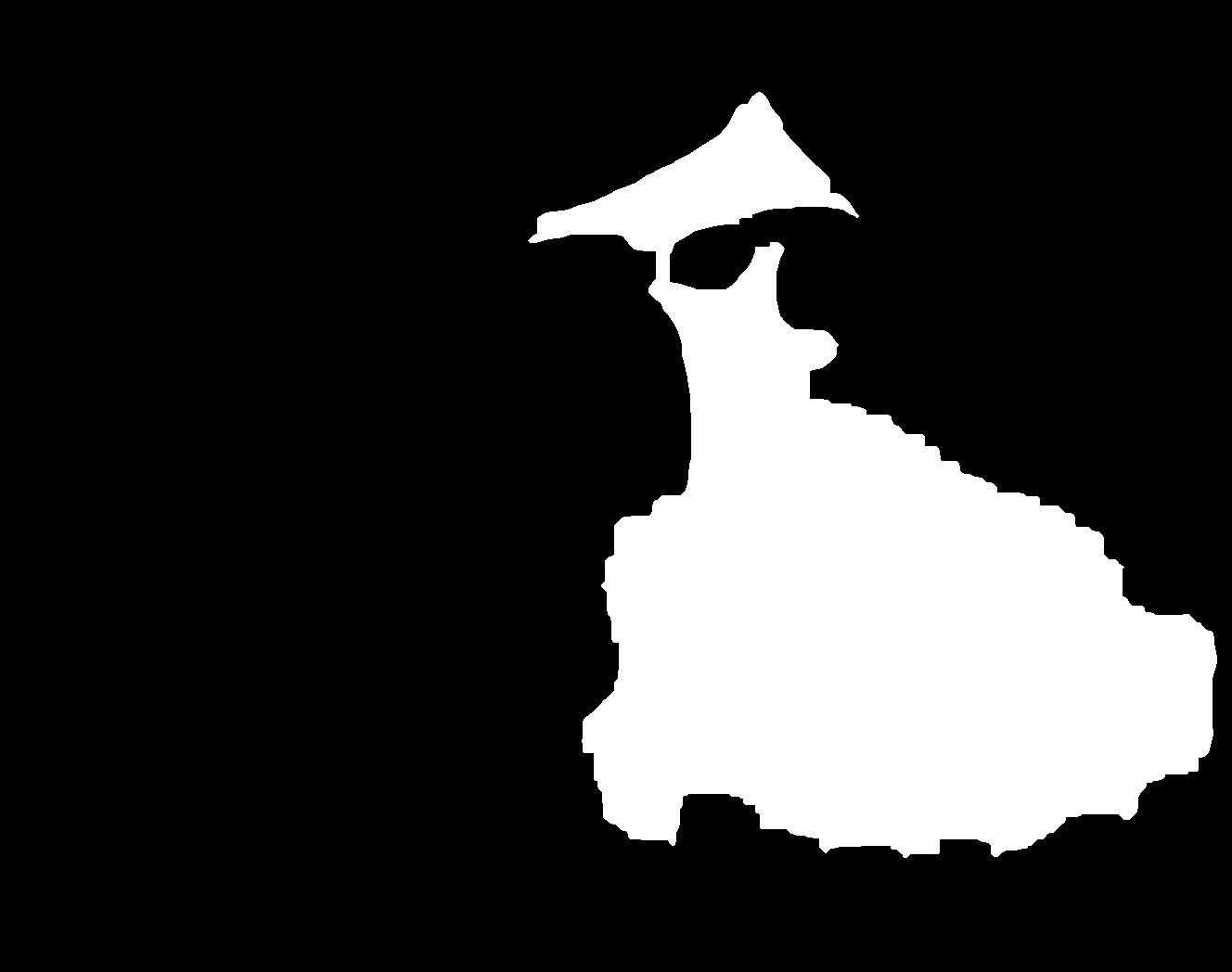

Supplement: S5 File — (ZIP) [file pone.0237972.s005.zip › S3_File IoU scores/masks/Experiment_1/cell/user_segmented/Automated/Automated_Participant15_mask_cell_E.jpg]

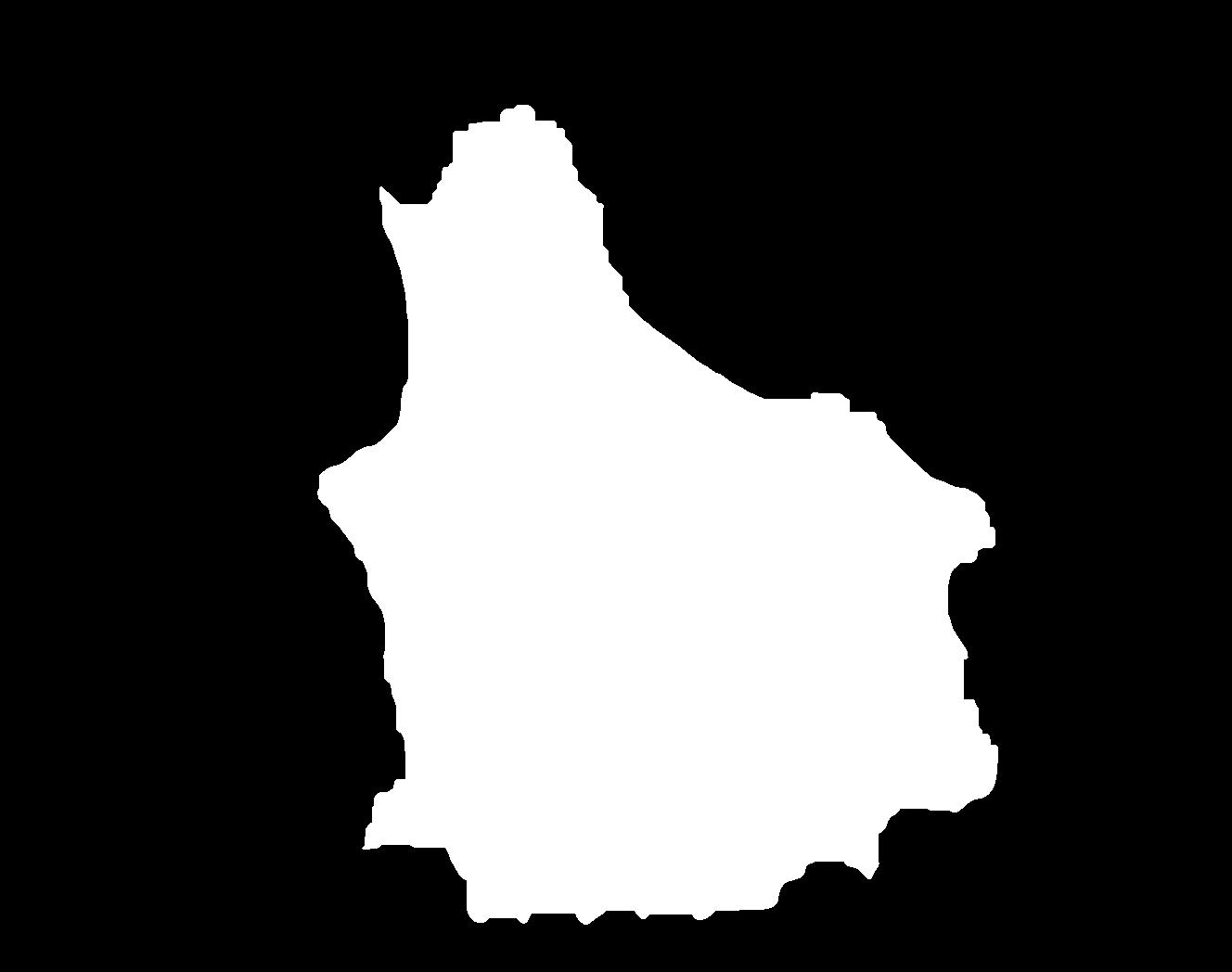

Supplement: S5 File — (ZIP) [file pone.0237972.s005.zip › S3_File IoU scores/masks/Experiment_1/cell/user_segmented/Automated/Automated_Participant15_mask_cell_F.jpg]

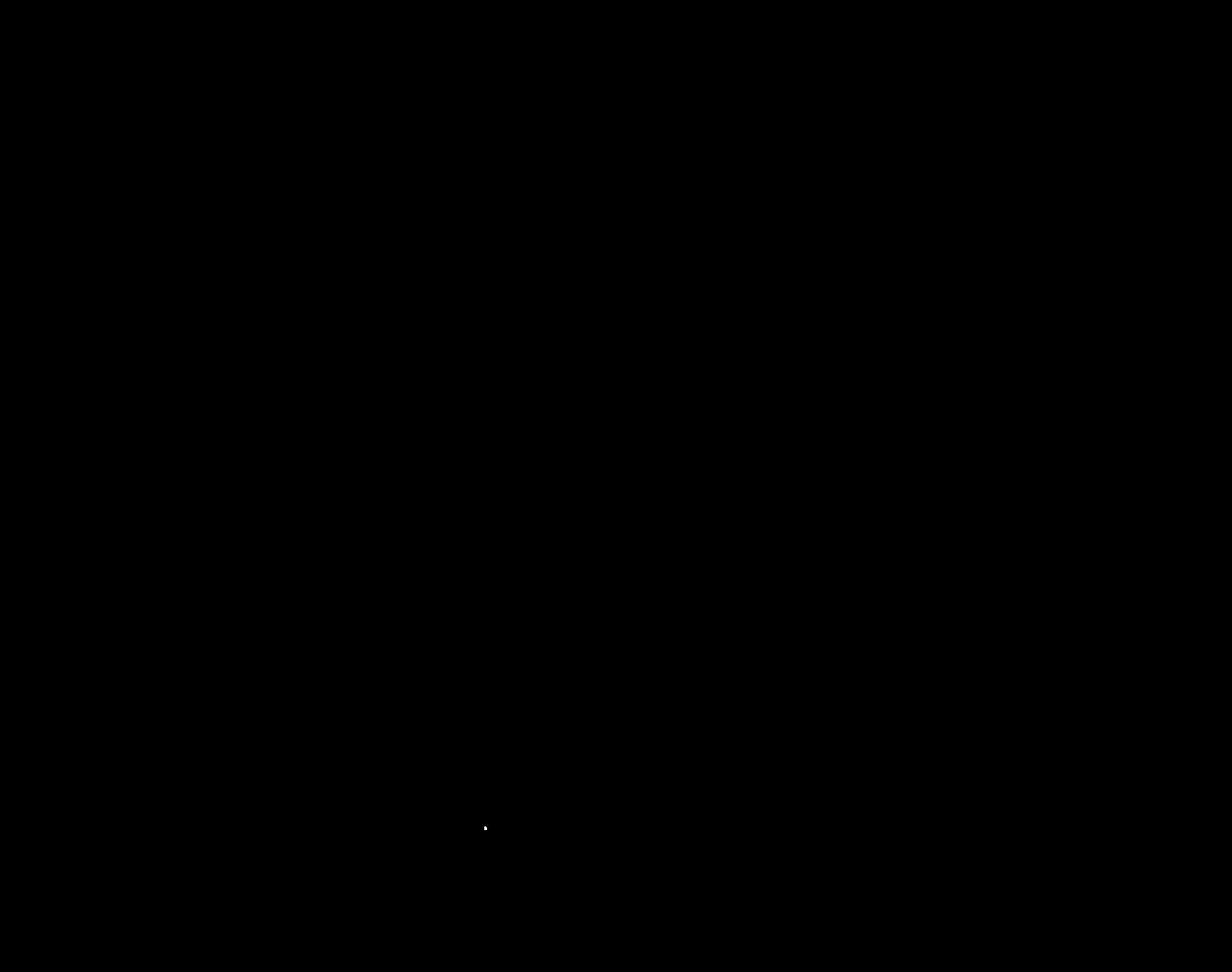

Supplement: S5 File — (ZIP) [file pone.0237972.s005.zip › S3_File IoU scores/masks/Experiment_1/cell/user_segmented/Automated/Automated_Participant15_mask_cell_G.jpg]

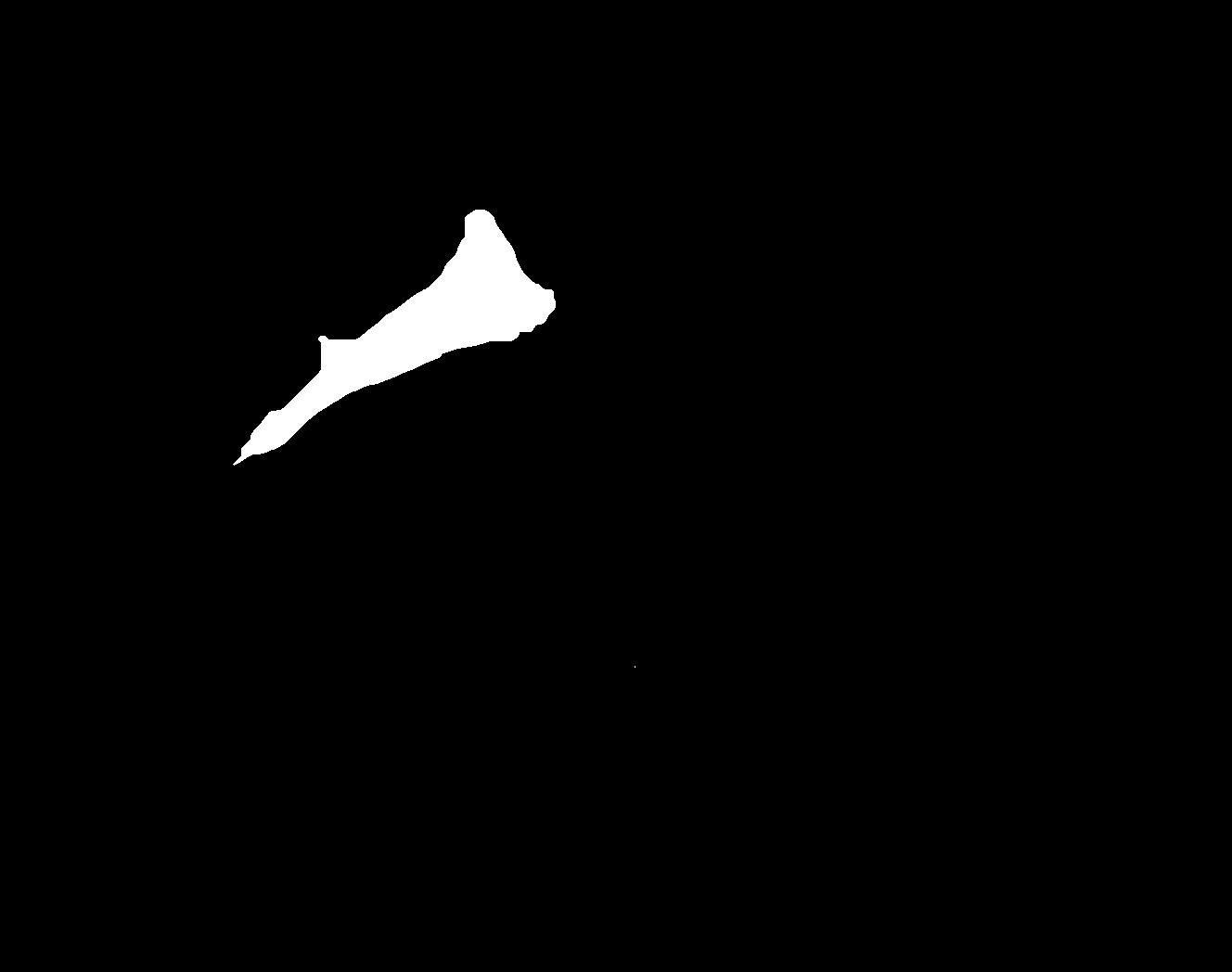

Supplement: S5 File — (ZIP) [file pone.0237972.s005.zip › S3_File IoU scores/masks/Experiment_1/cell/user_segmented/Automated/Automated_Participant15_mask_cell_H.jpg]

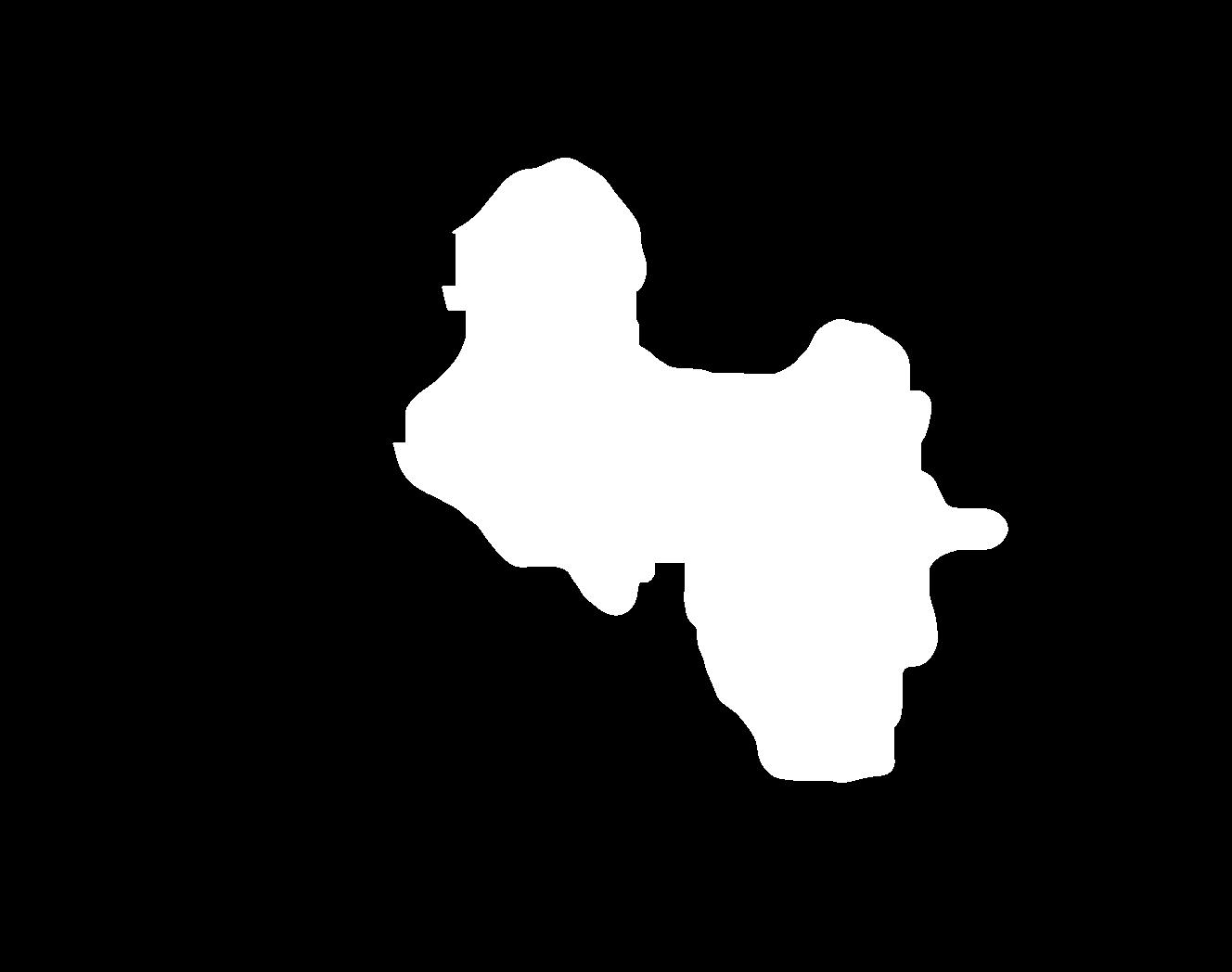

Supplement: S5 File — (ZIP) [file pone.0237972.s005.zip › S3_File IoU scores/masks/Experiment_1/cell/user_segmented/Automated/Automated_Participant15_mask_cell_I.jpg]

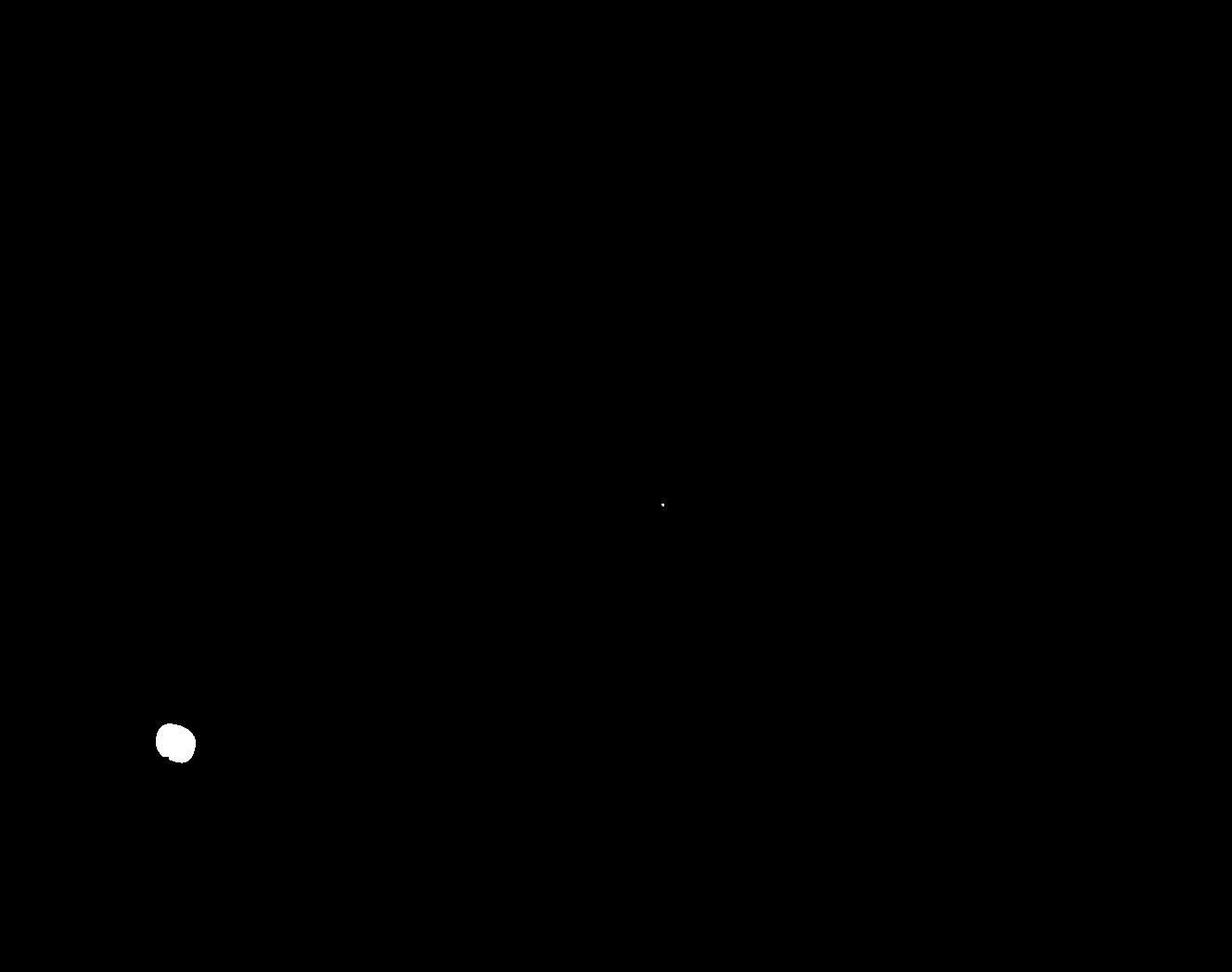

Supplement: S5 File — (ZIP) [file pone.0237972.s005.zip › S3_File IoU scores/masks/Experiment_1/cell/user_segmented/Automated/Automated_Participant15_mask_cell_J.jpg]

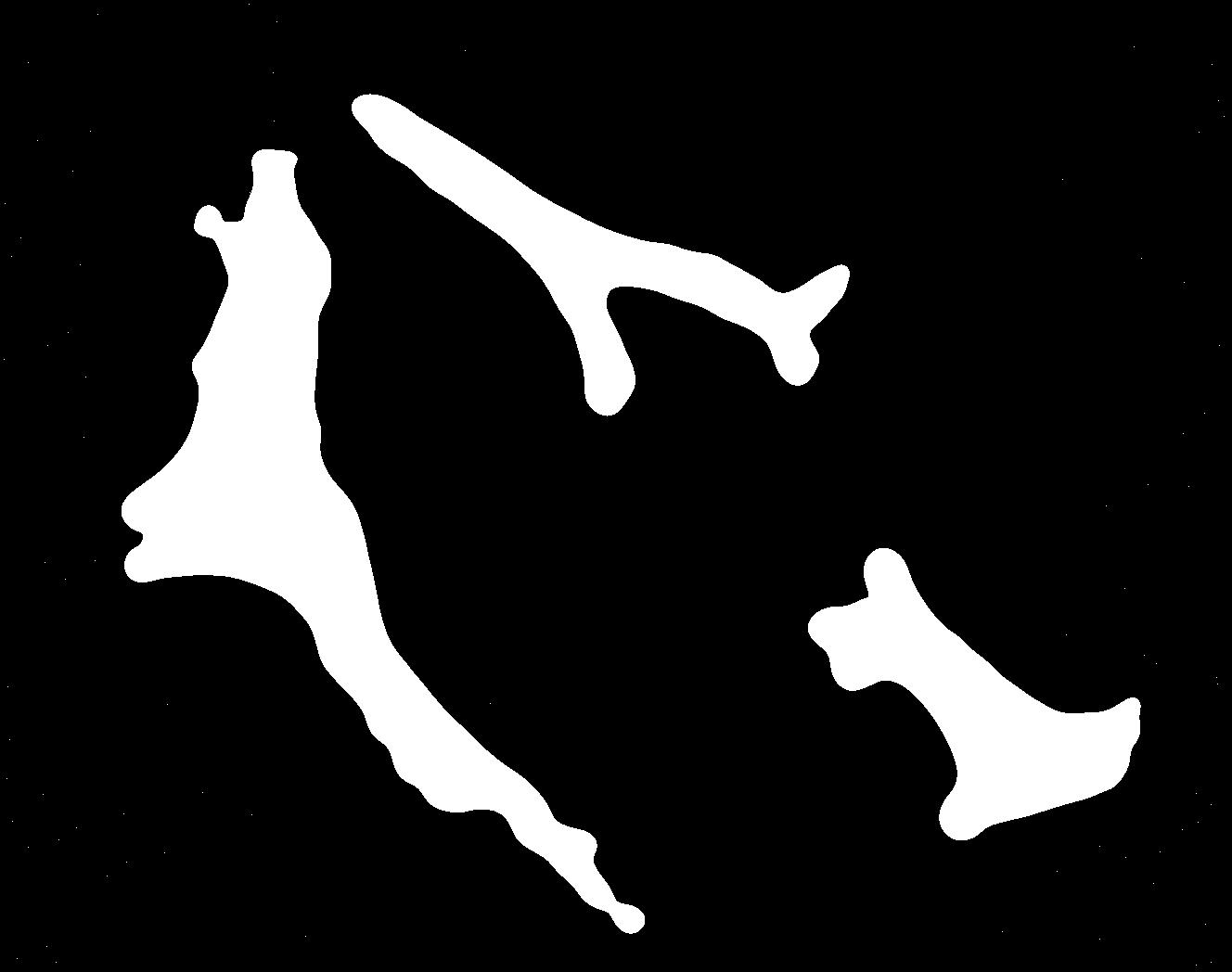

Supplement: S5 File — (ZIP) [file pone.0237972.s005.zip › S3_File IoU scores/masks/Experiment_1/cell/user_segmented/Automated/Automated_Participant1_mask_cell_A.jpg]

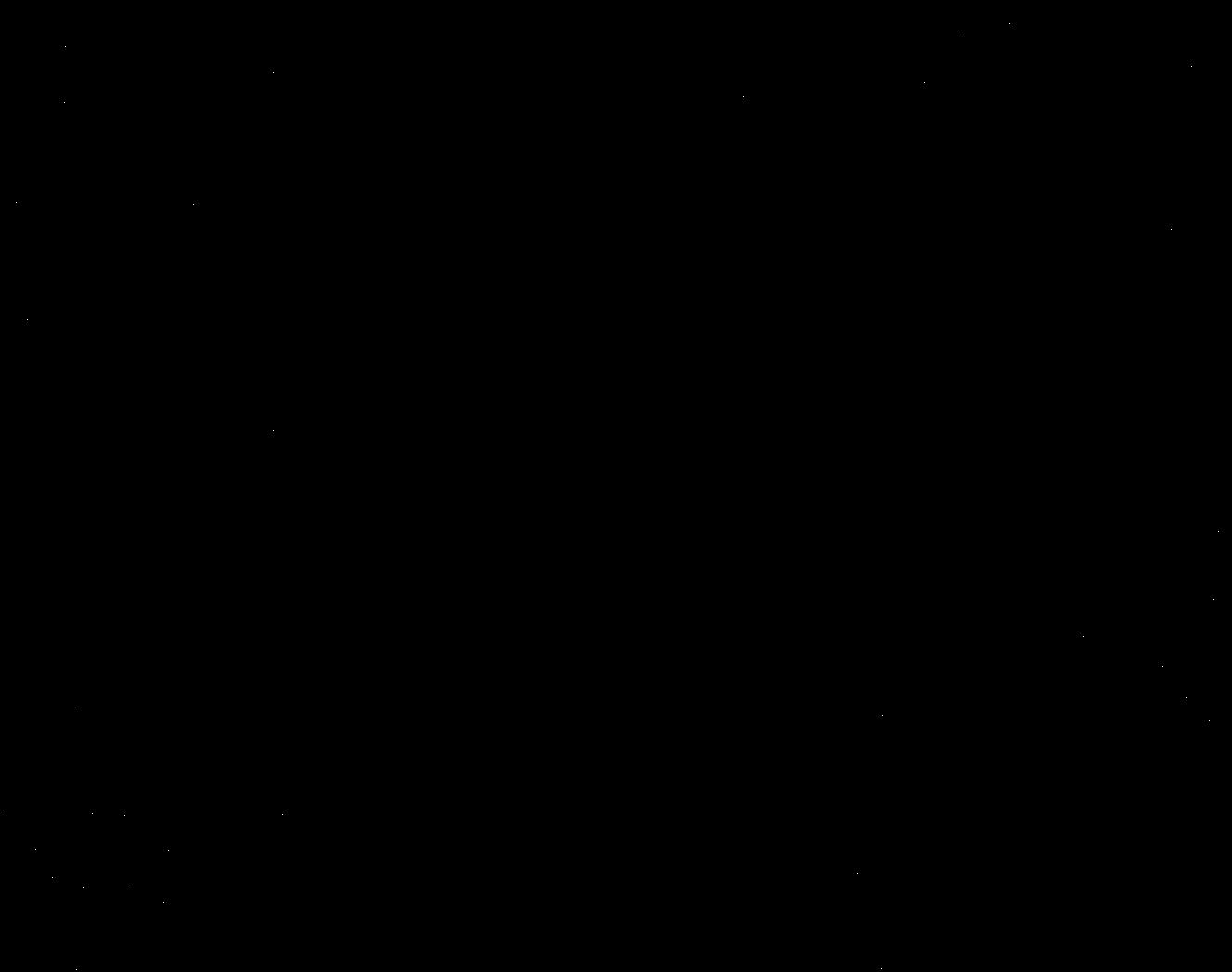

Supplement: S5 File — (ZIP) [file pone.0237972.s005.zip › S3_File IoU scores/masks/Experiment_1/cell/user_segmented/Automated/Automated_Participant1_mask_cell_B.jpg]

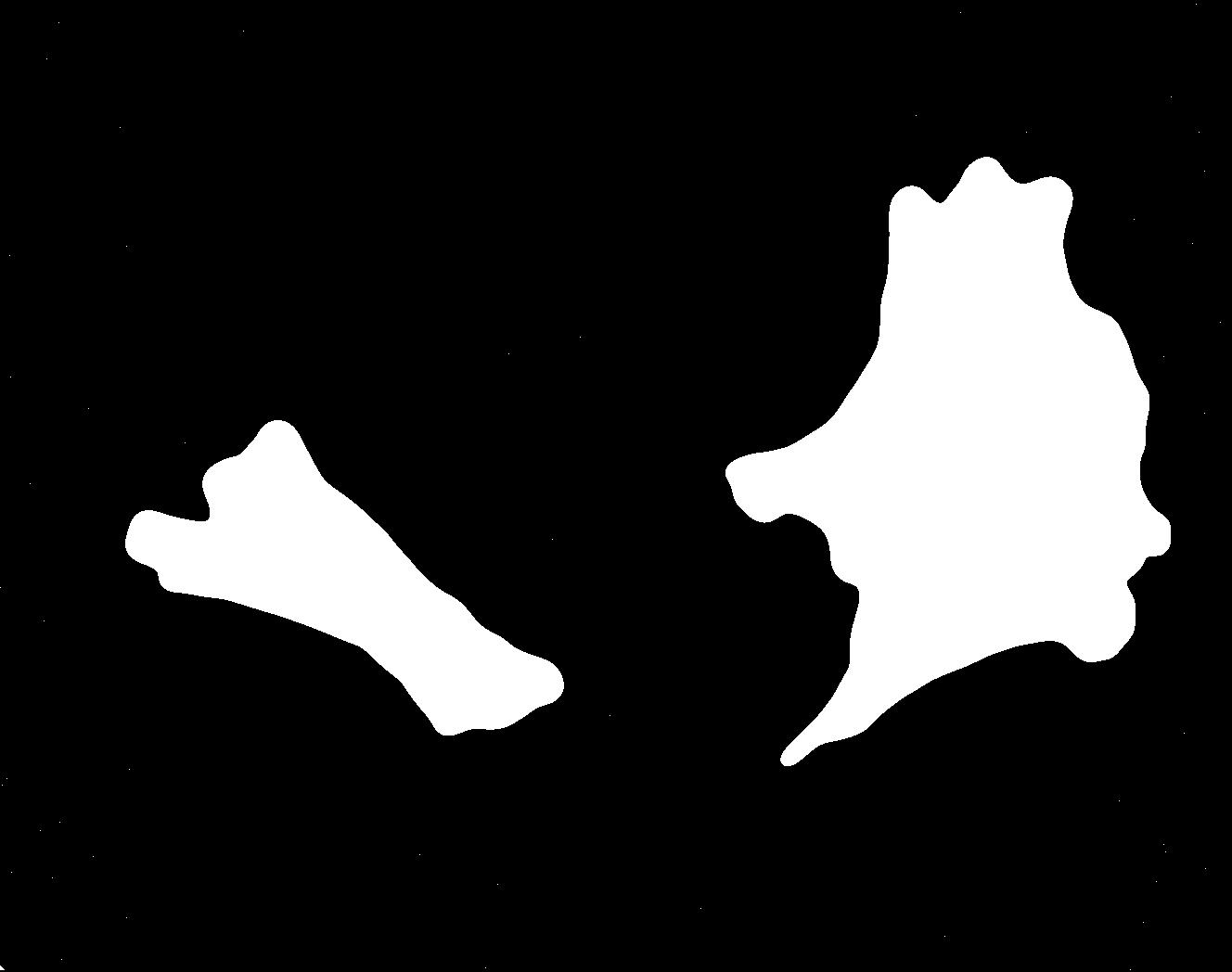

Supplement: S5 File — (ZIP) [file pone.0237972.s005.zip › S3_File IoU scores/masks/Experiment_1/cell/user_segmented/Automated/Automated_Participant1_mask_cell_C.jpg]

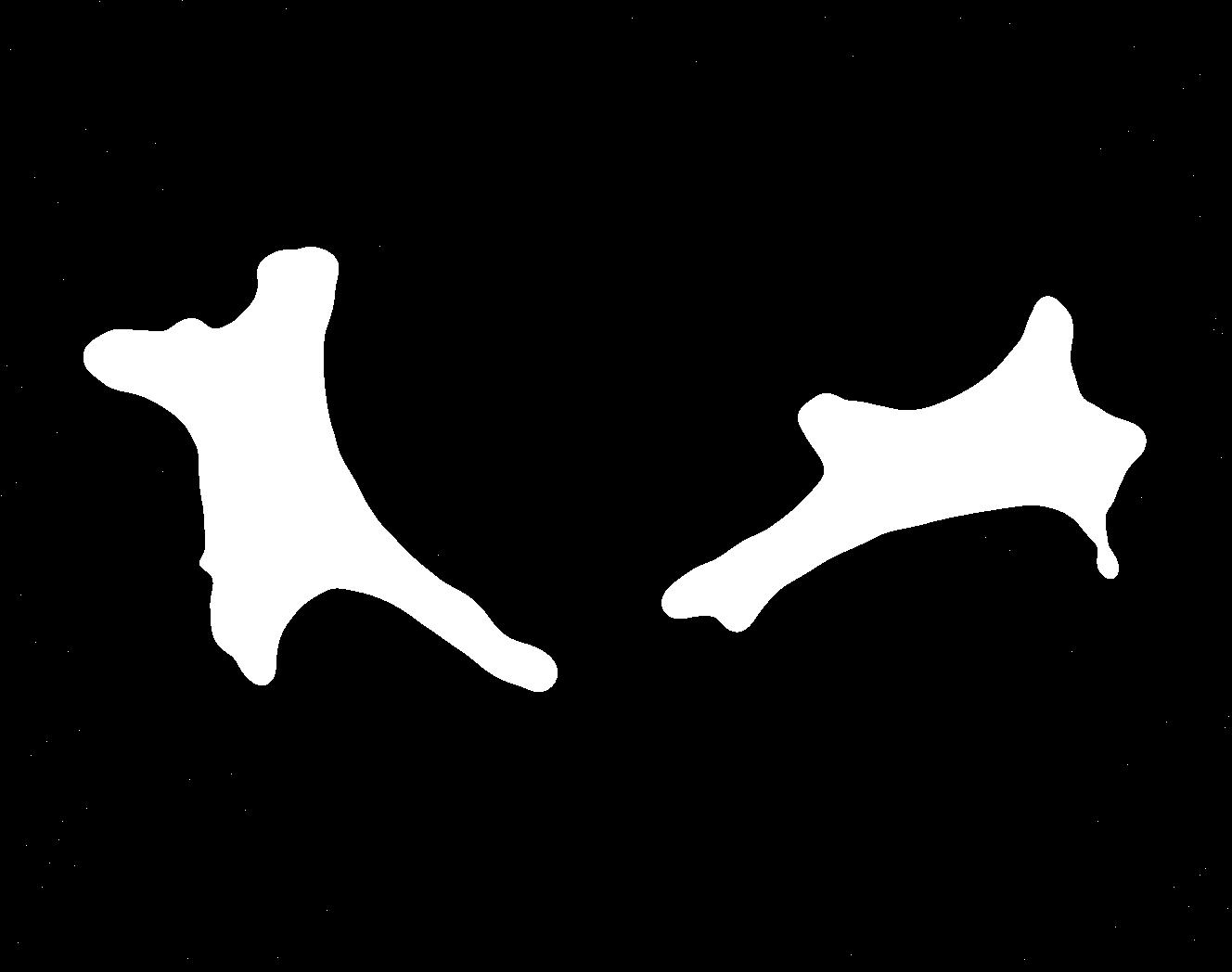

Supplement: S5 File — (ZIP) [file pone.0237972.s005.zip › S3_File IoU scores/masks/Experiment_1/cell/user_segmented/Automated/Automated_Participant1_mask_cell_D.jpg]

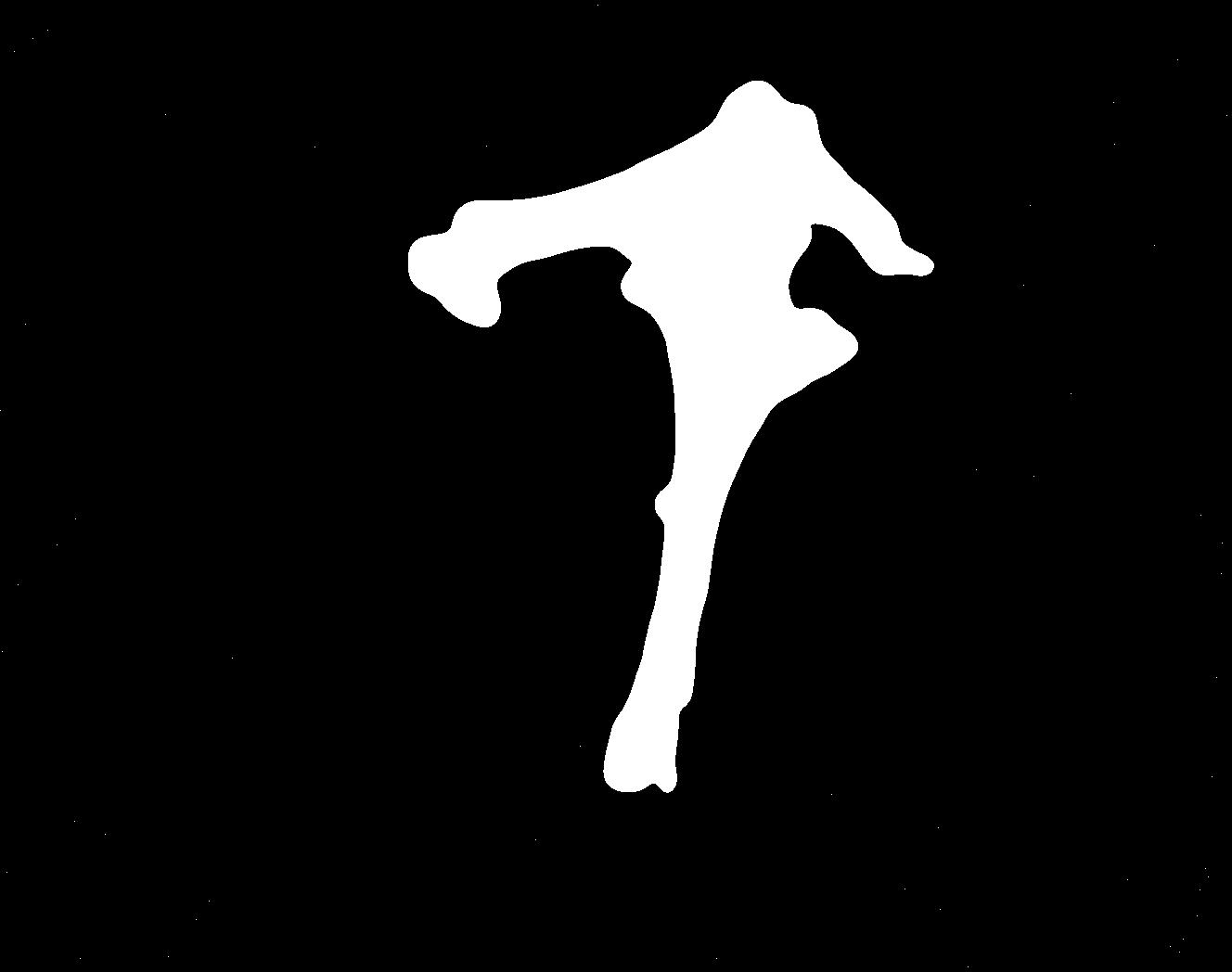

Supplement: S5 File — (ZIP) [file pone.0237972.s005.zip › S3_File IoU scores/masks/Experiment_1/cell/user_segmented/Automated/Automated_Participant1_mask_cell_E.jpg]

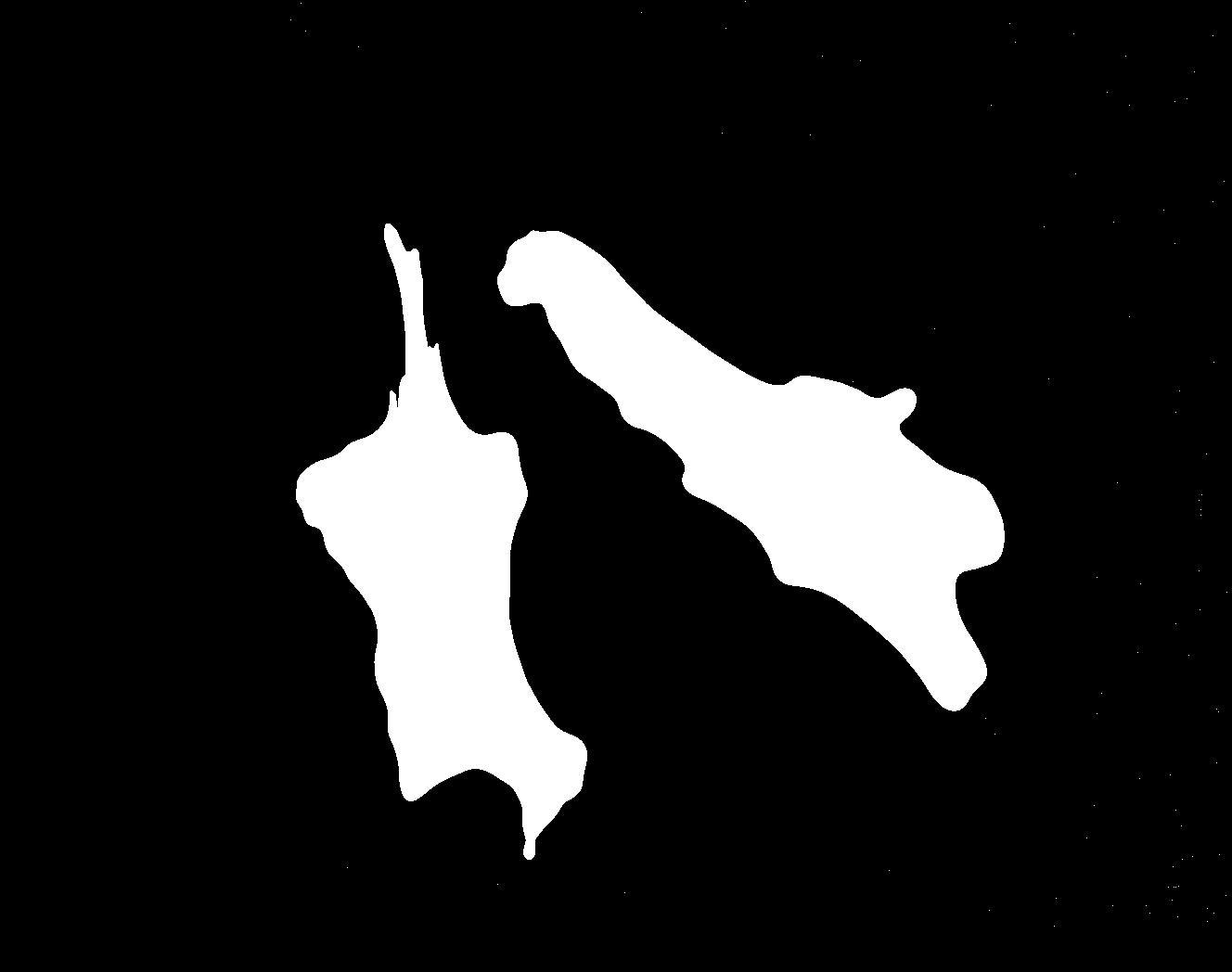

Supplement: S5 File — (ZIP) [file pone.0237972.s005.zip › S3_File IoU scores/masks/Experiment_1/cell/user_segmented/Automated/Automated_Participant1_mask_cell_F.jpg]

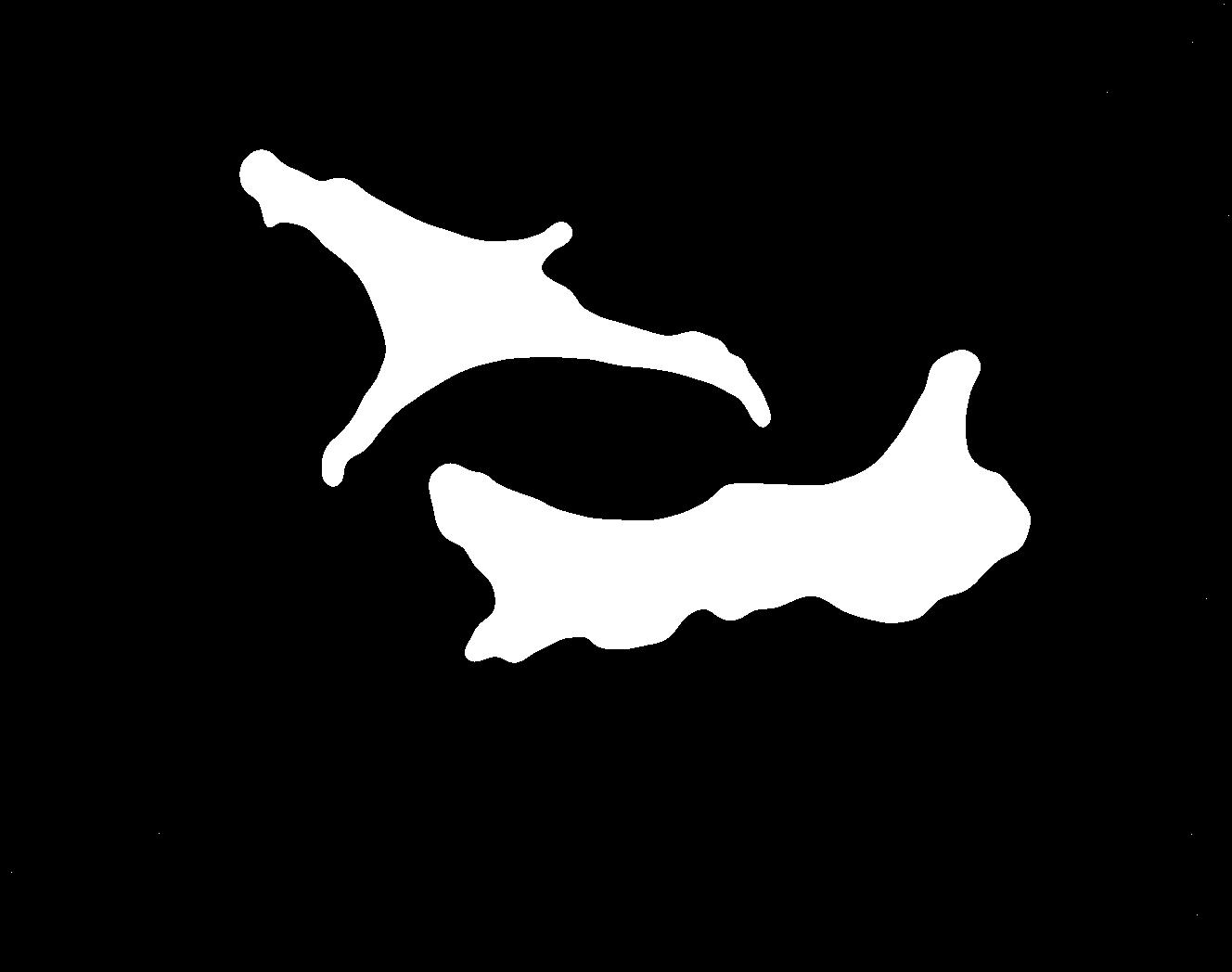

Supplement: S5 File — (ZIP) [file pone.0237972.s005.zip › S3_File IoU scores/masks/Experiment_1/cell/user_segmented/Automated/Automated_Participant1_mask_cell_G.jpg]

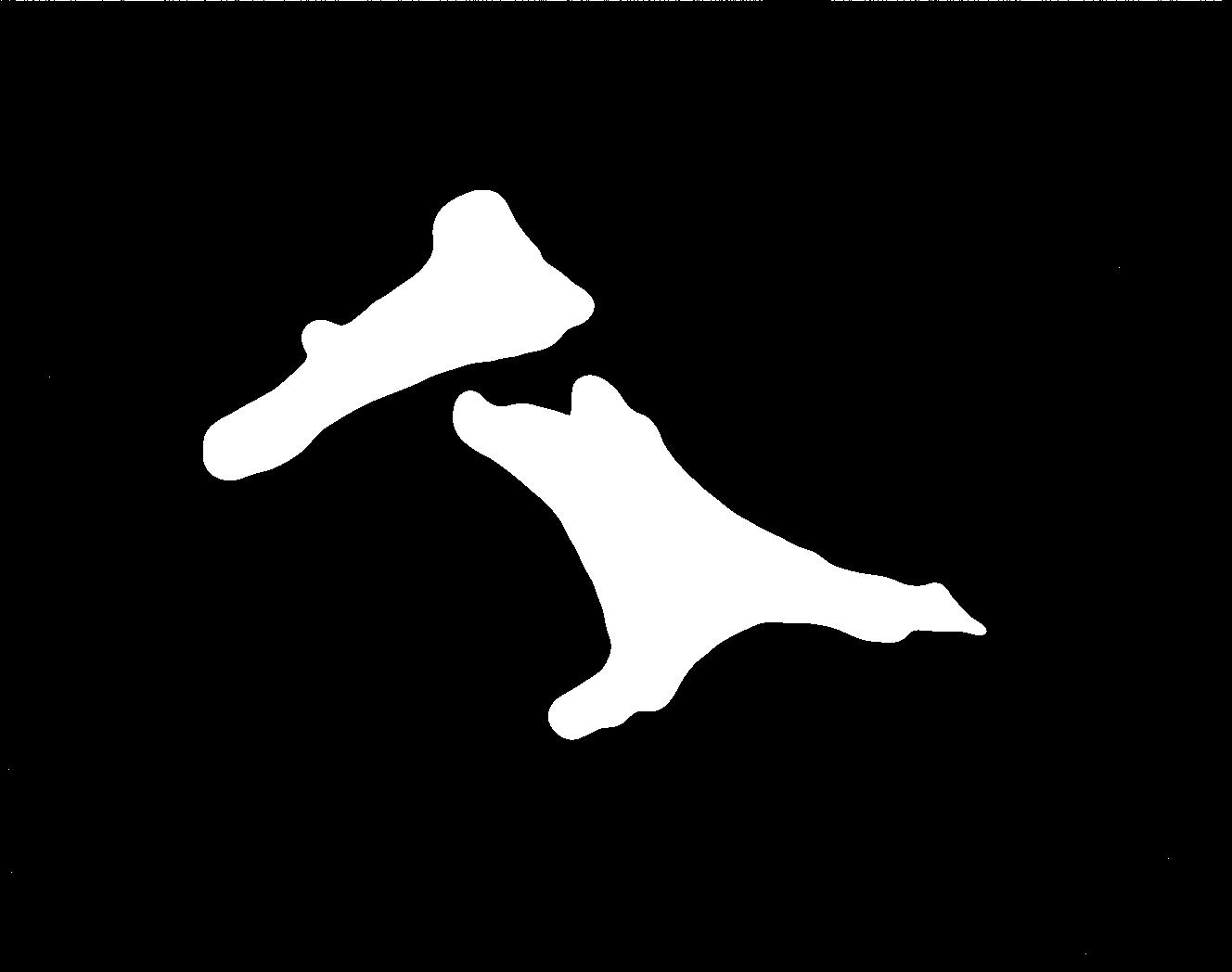

Supplement: S5 File — (ZIP) [file pone.0237972.s005.zip › S3_File IoU scores/masks/Experiment_1/cell/user_segmented/Automated/Automated_Participant1_mask_cell_H.jpg]

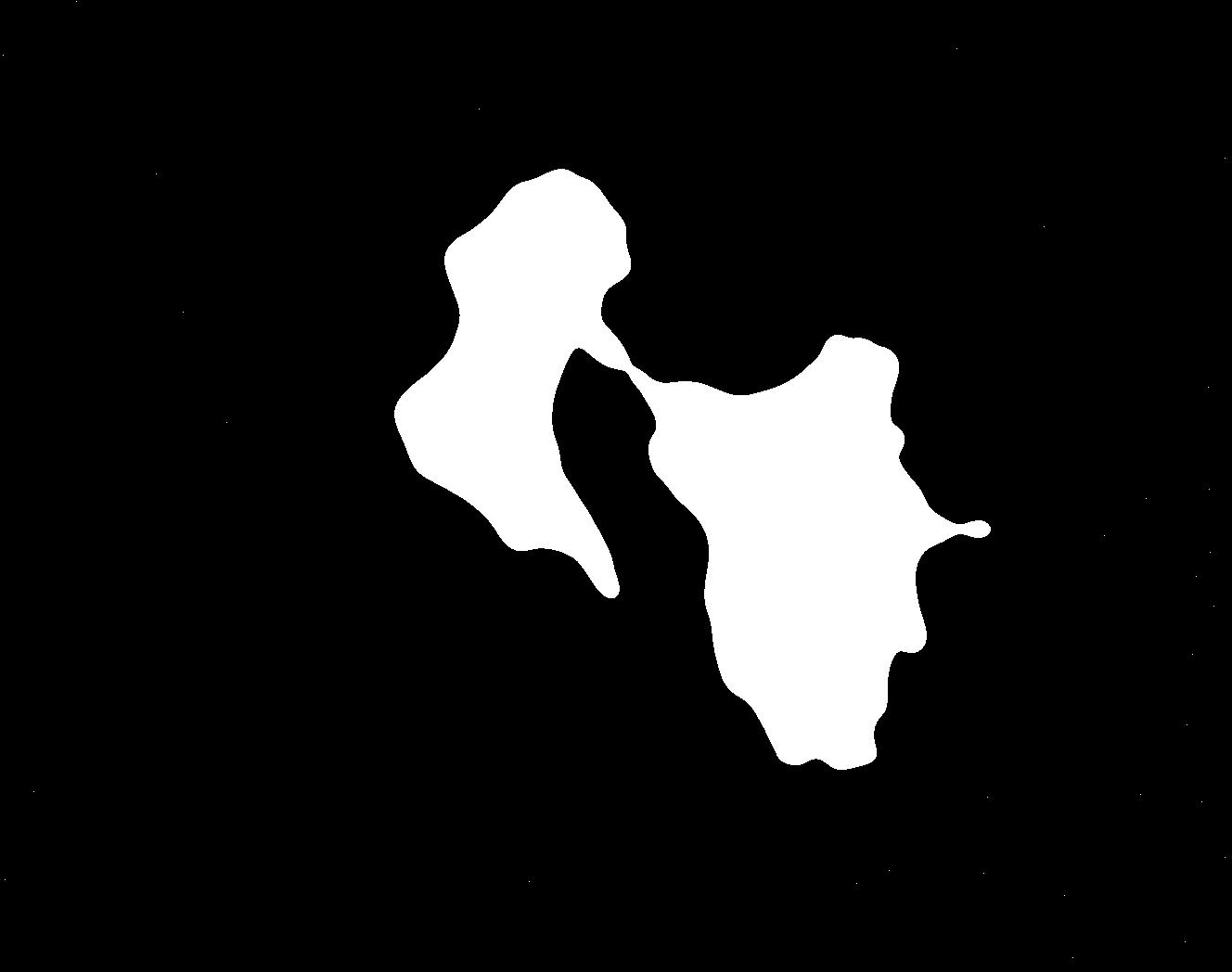

Supplement: S5 File — (ZIP) [file pone.0237972.s005.zip › S3_File IoU scores/masks/Experiment_1/cell/user_segmented/Automated/Automated_Participant1_mask_cell_I.jpg]

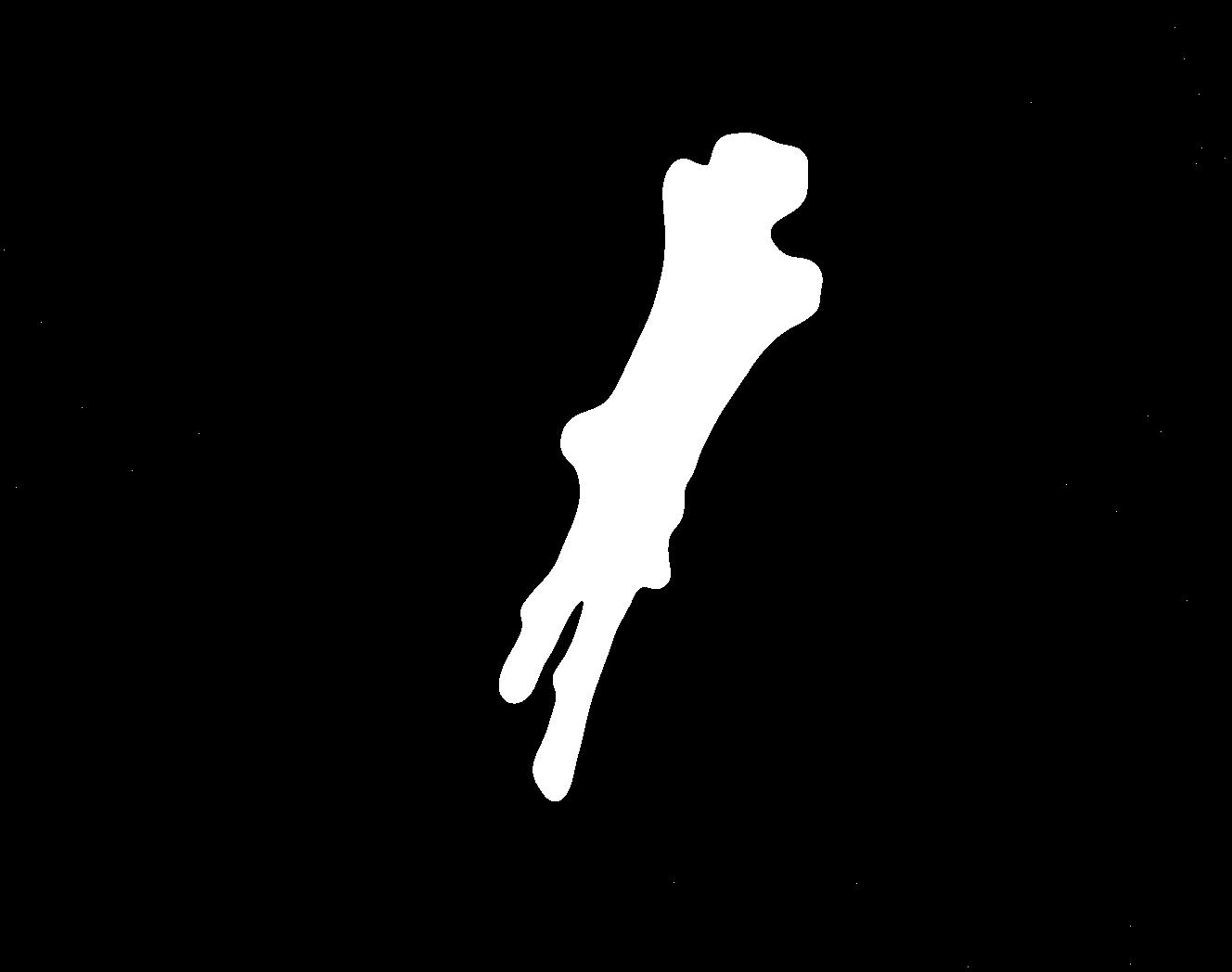

Supplement: S5 File — (ZIP) [file pone.0237972.s005.zip › S3_File IoU scores/masks/Experiment_1/cell/user_segmented/Automated/Automated_Participant1_mask_cell_J.jpg]

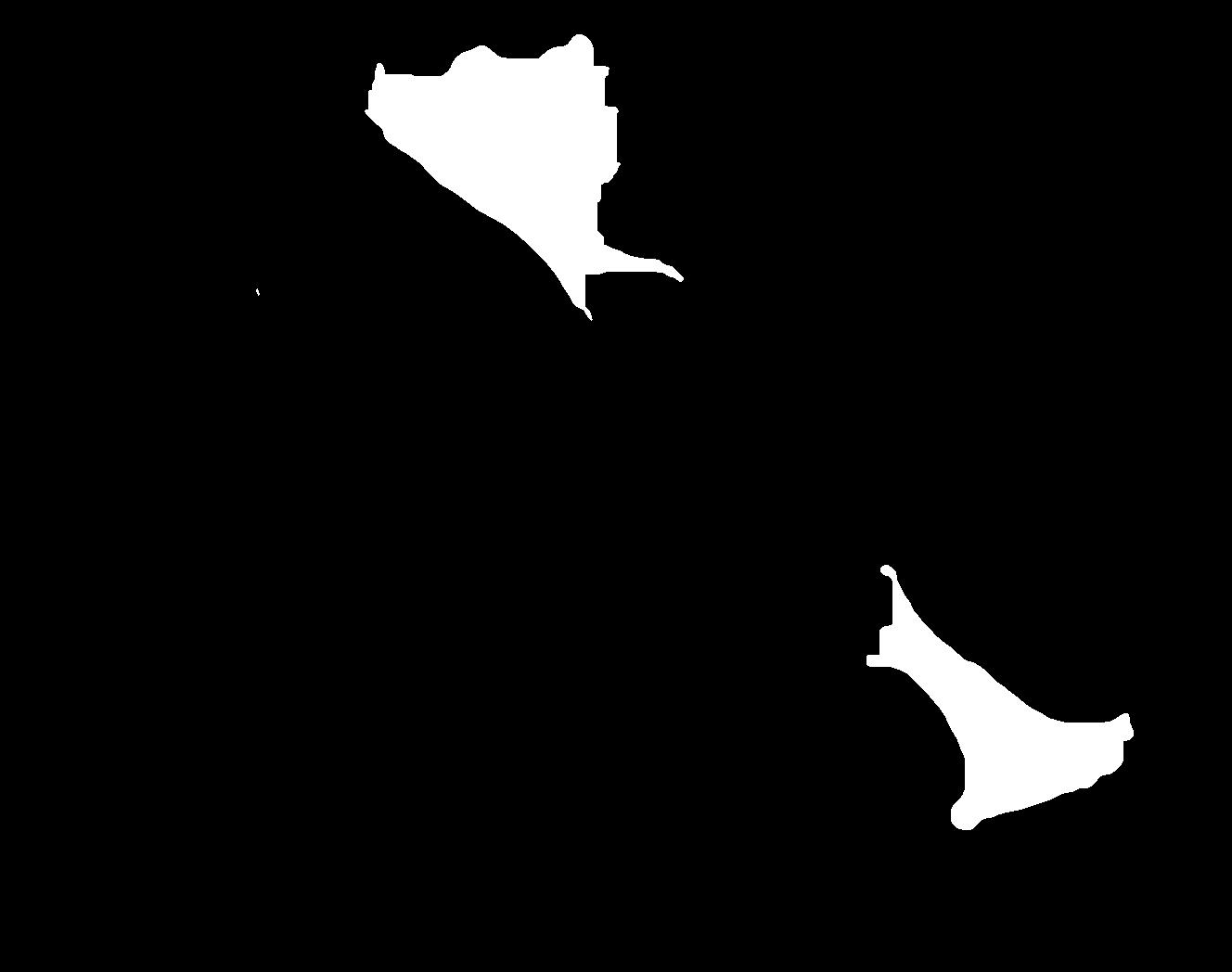

Supplement: S5 File — (ZIP) [file pone.0237972.s005.zip › S3_File IoU scores/masks/Experiment_1/cell/user_segmented/Automated/Automated_Participant4_mask_cell_A.jpg]

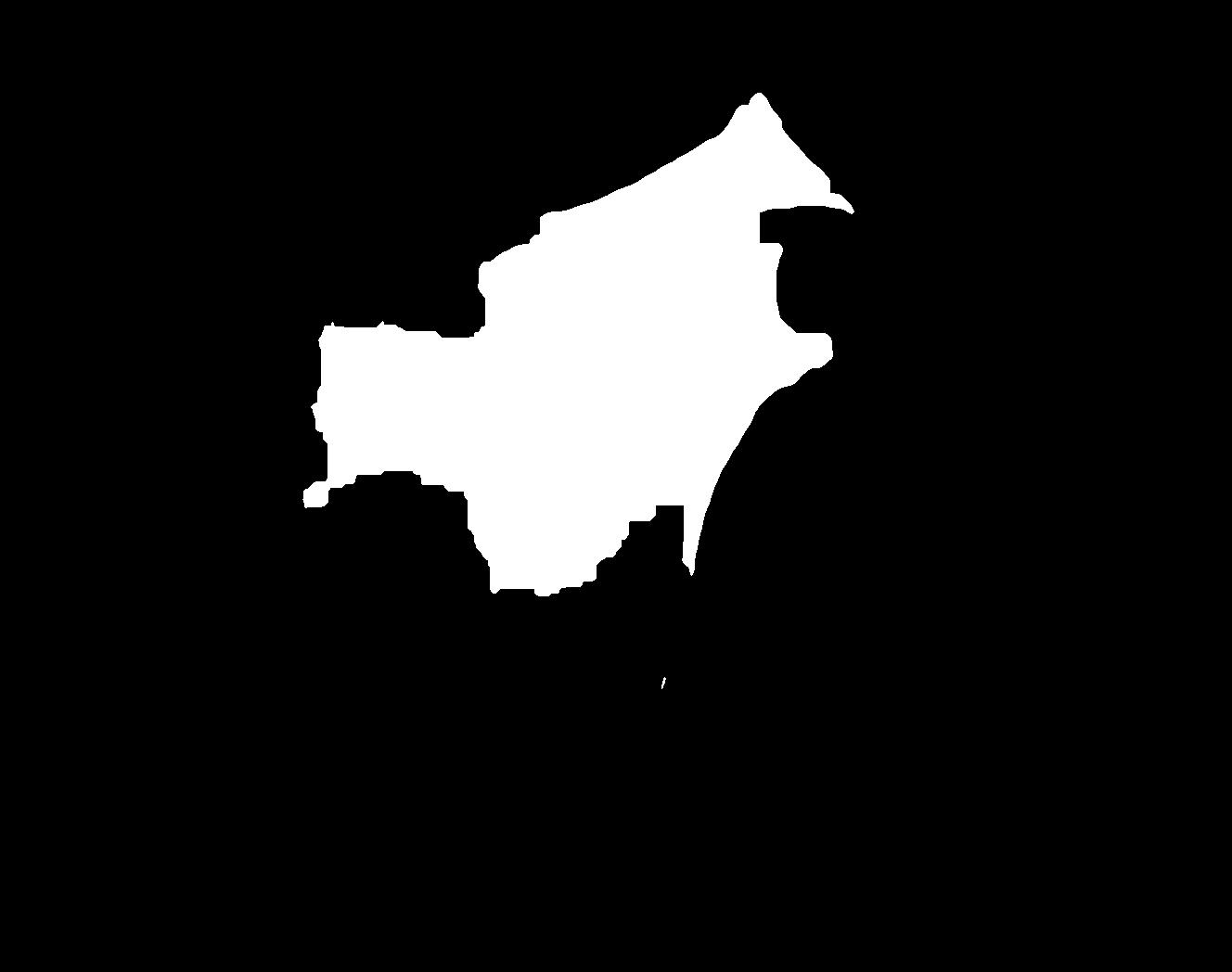

Supplement: S5 File — (ZIP) [file pone.0237972.s005.zip › S3_File IoU scores/masks/Experiment_1/cell/user_segmented/Automated/Automated_Participant4_mask_cell_E.jpg]

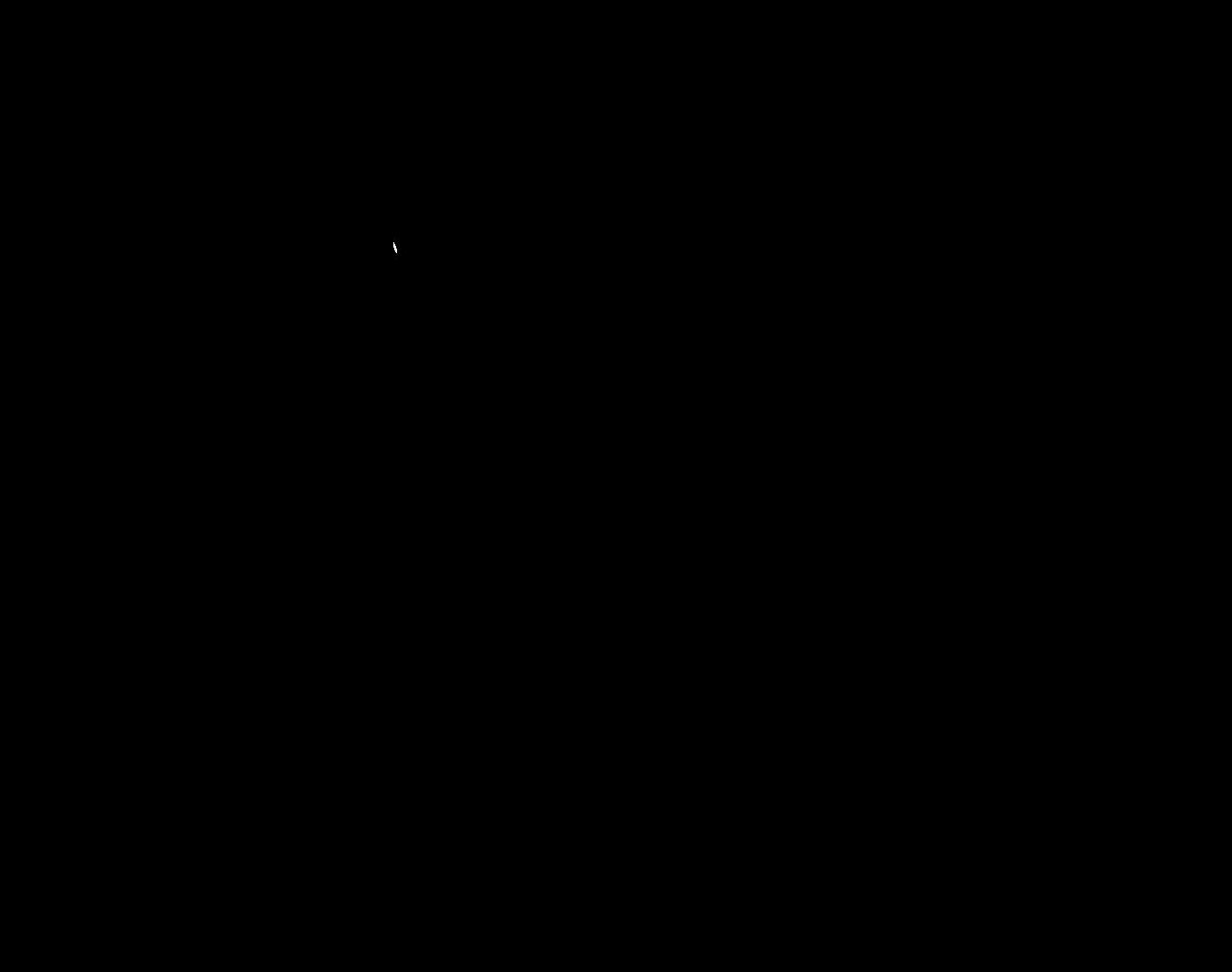

Supplement: S5 File — (ZIP) [file pone.0237972.s005.zip › S3_File IoU scores/masks/Experiment_1/cell/user_segmented/Automated/Automated_Participant4_mask_cell_F.jpg]

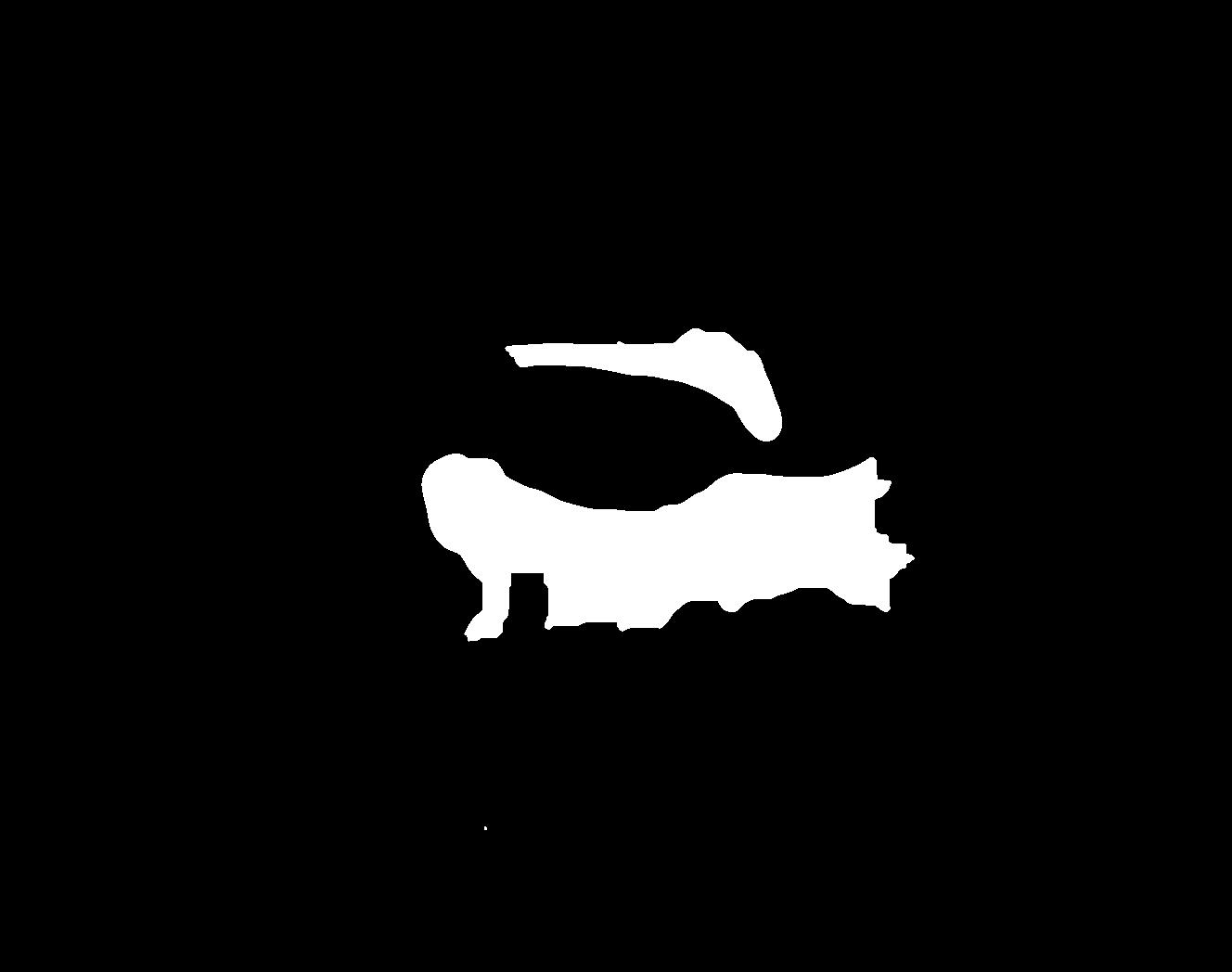

Supplement: S5 File — (ZIP) [file pone.0237972.s005.zip › S3_File IoU scores/masks/Experiment_1/cell/user_segmented/Automated/Automated_Participant4_mask_cell_G.jpg]

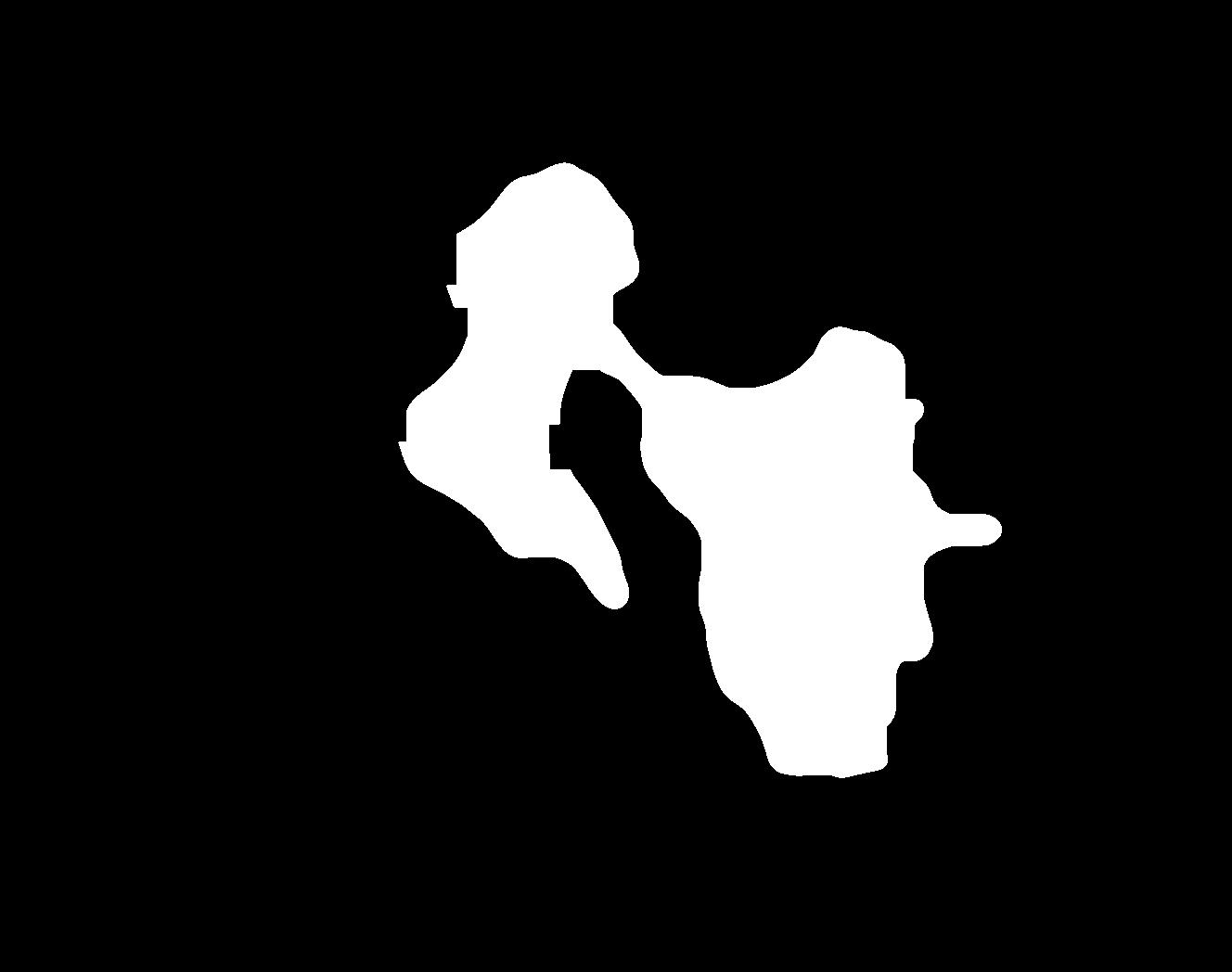

Supplement: S5 File — (ZIP) [file pone.0237972.s005.zip › S3_File IoU scores/masks/Experiment_1/cell/user_segmented/Automated/Automated_Participant4_mask_cell_I.jpg]

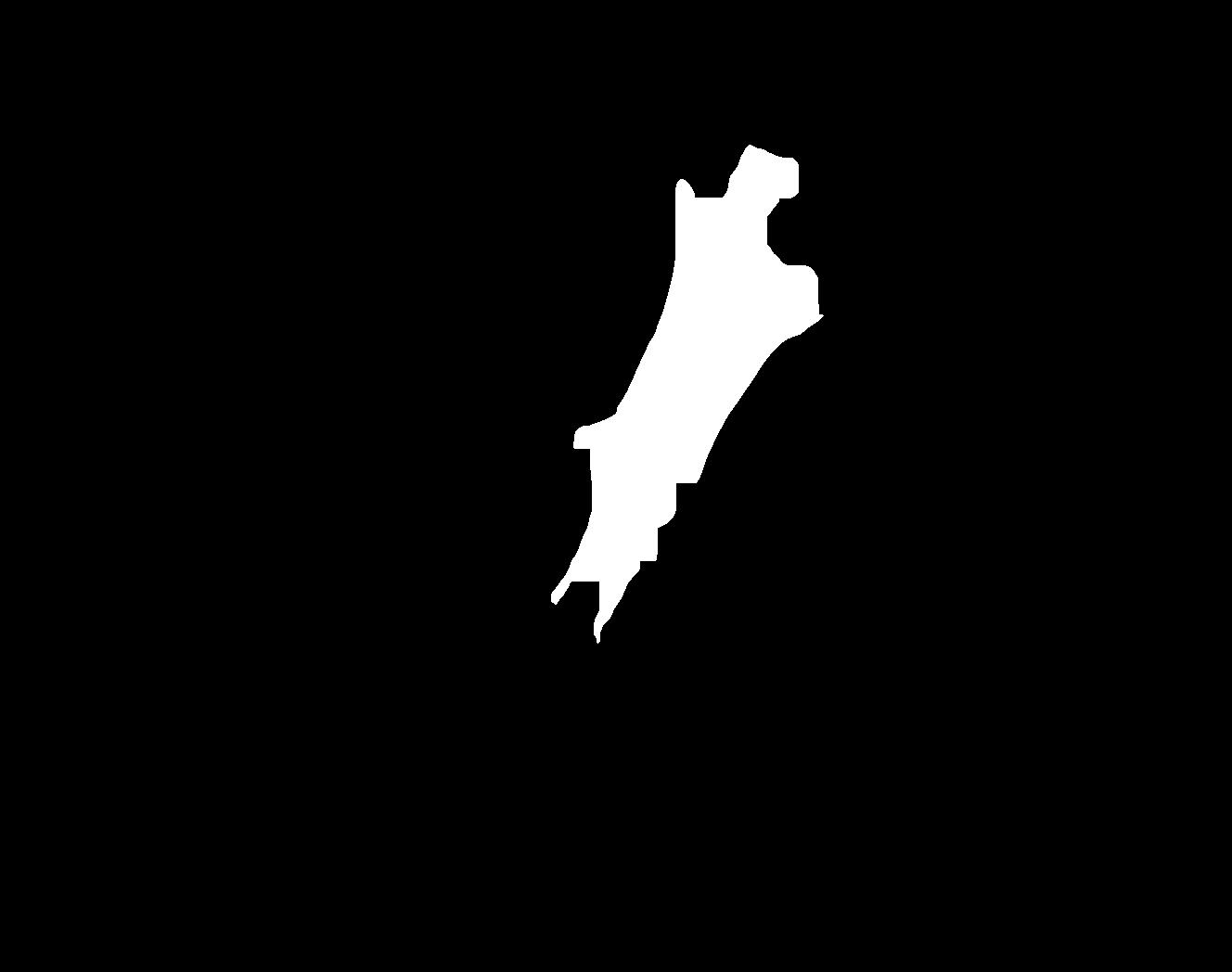

Supplement: S5 File — (ZIP) [file pone.0237972.s005.zip › S3_File IoU scores/masks/Experiment_1/cell/user_segmented/Automated/Automated_Participant4_mask_cell_J.jpg]

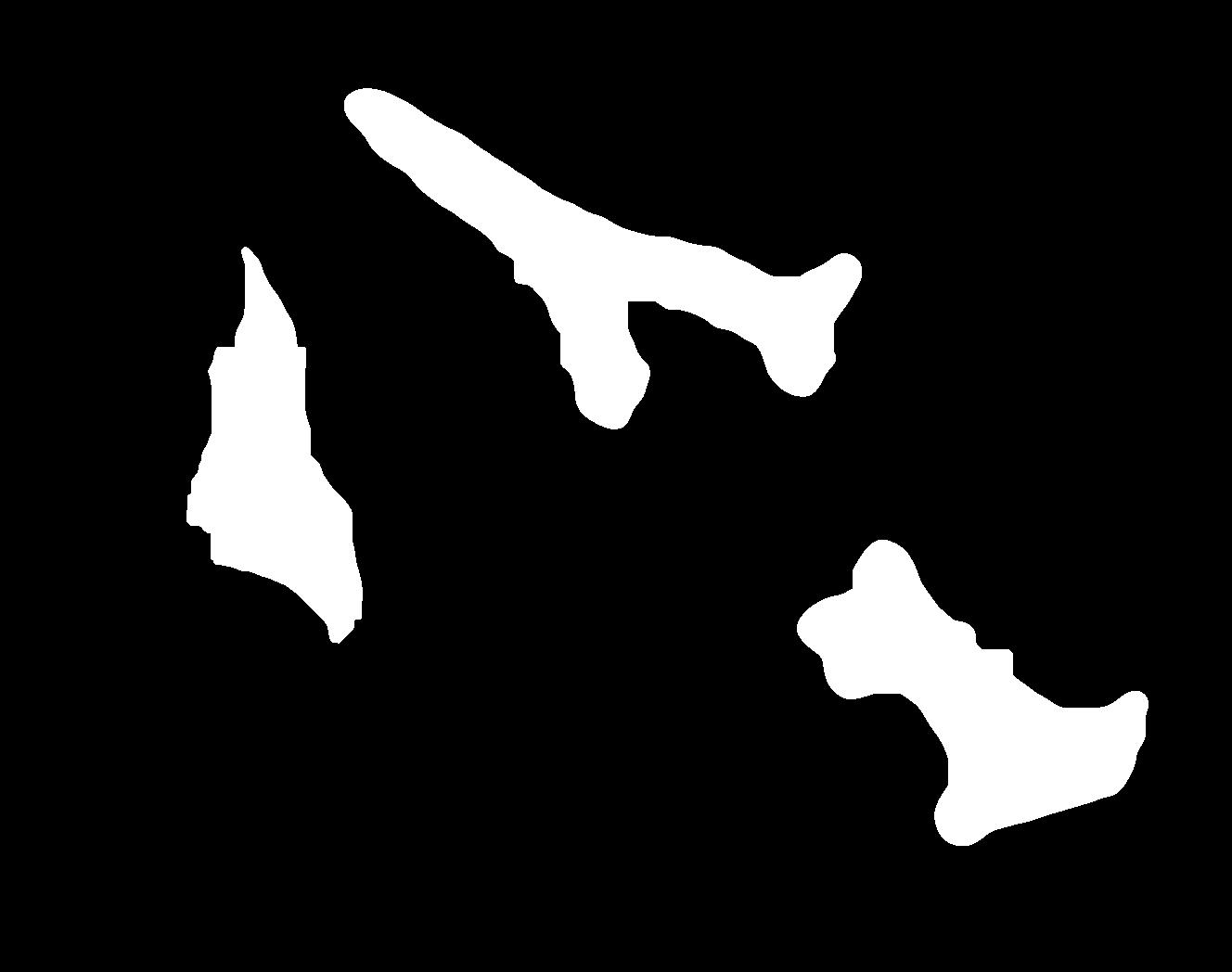

Supplement: S5 File — (ZIP) [file pone.0237972.s005.zip › S3_File IoU scores/masks/Experiment_1/cell/user_segmented/Automated/Automated_Participant6_mask_cell_A.jpg]

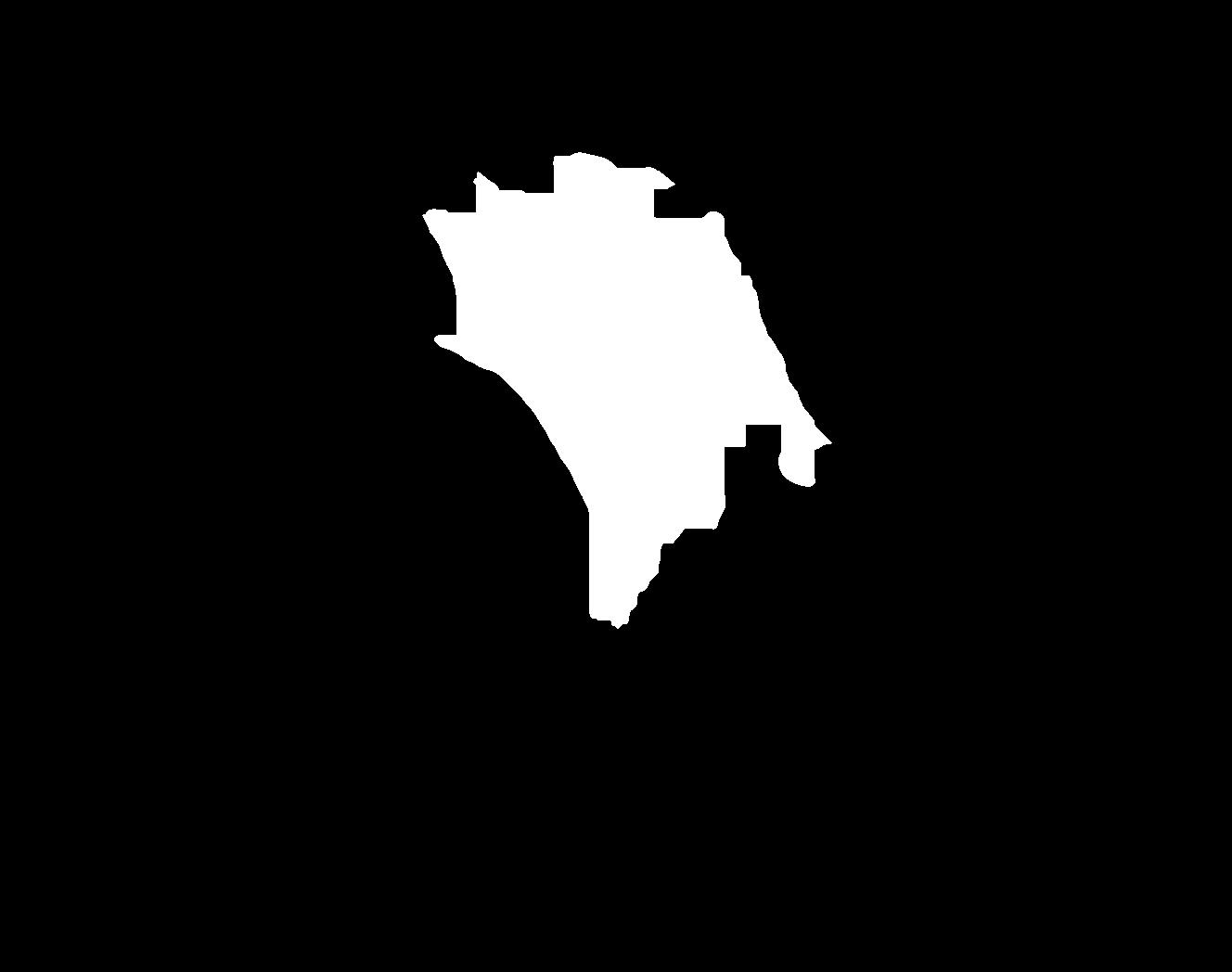

Supplement: S5 File — (ZIP) [file pone.0237972.s005.zip › S3_File IoU scores/masks/Experiment_1/cell/user_segmented/Automated/Automated_Participant6_mask_cell_B.jpg]

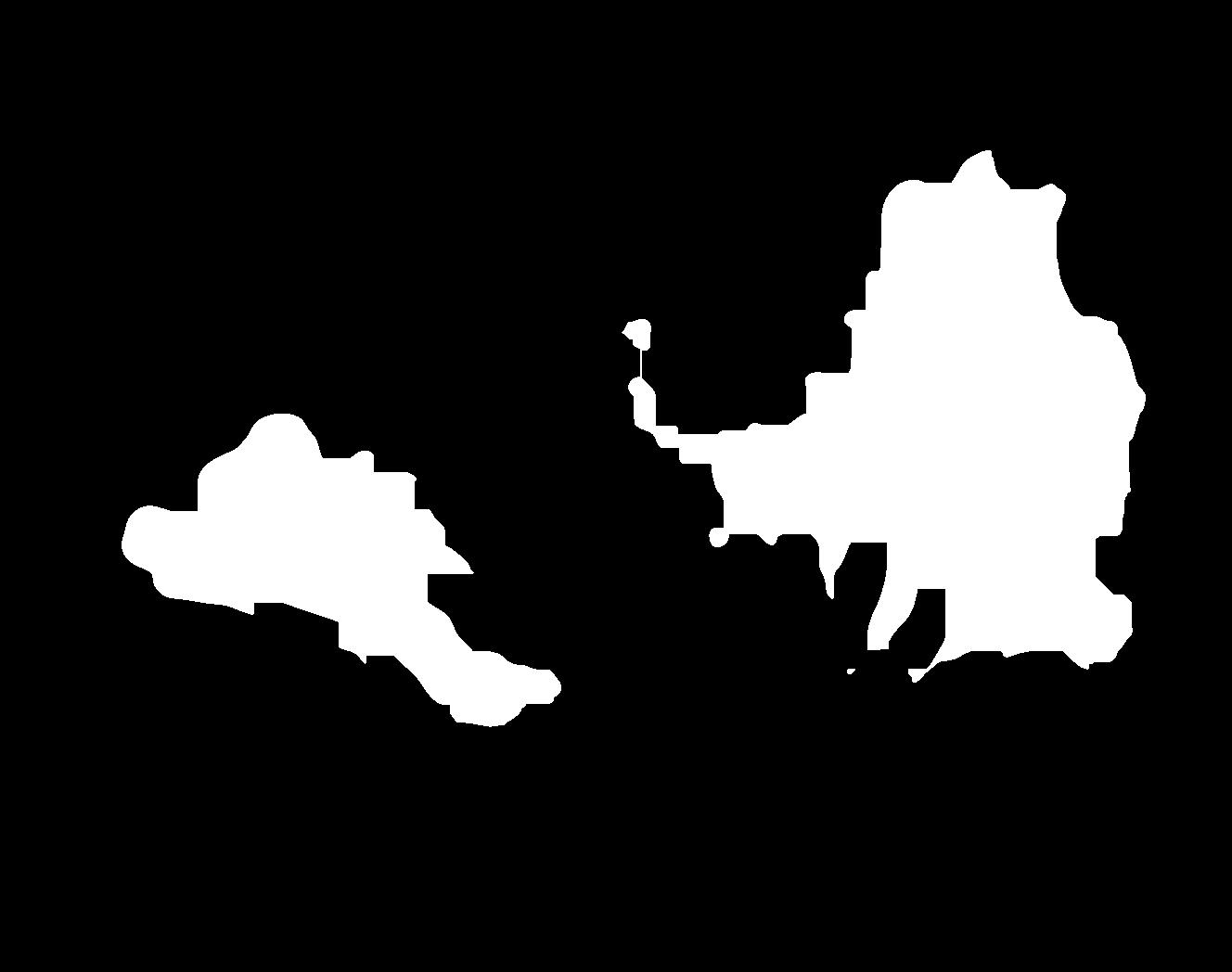

Supplement: S5 File — (ZIP) [file pone.0237972.s005.zip › S3_File IoU scores/masks/Experiment_1/cell/user_segmented/Automated/Automated_Participant6_mask_cell_C.jpg]

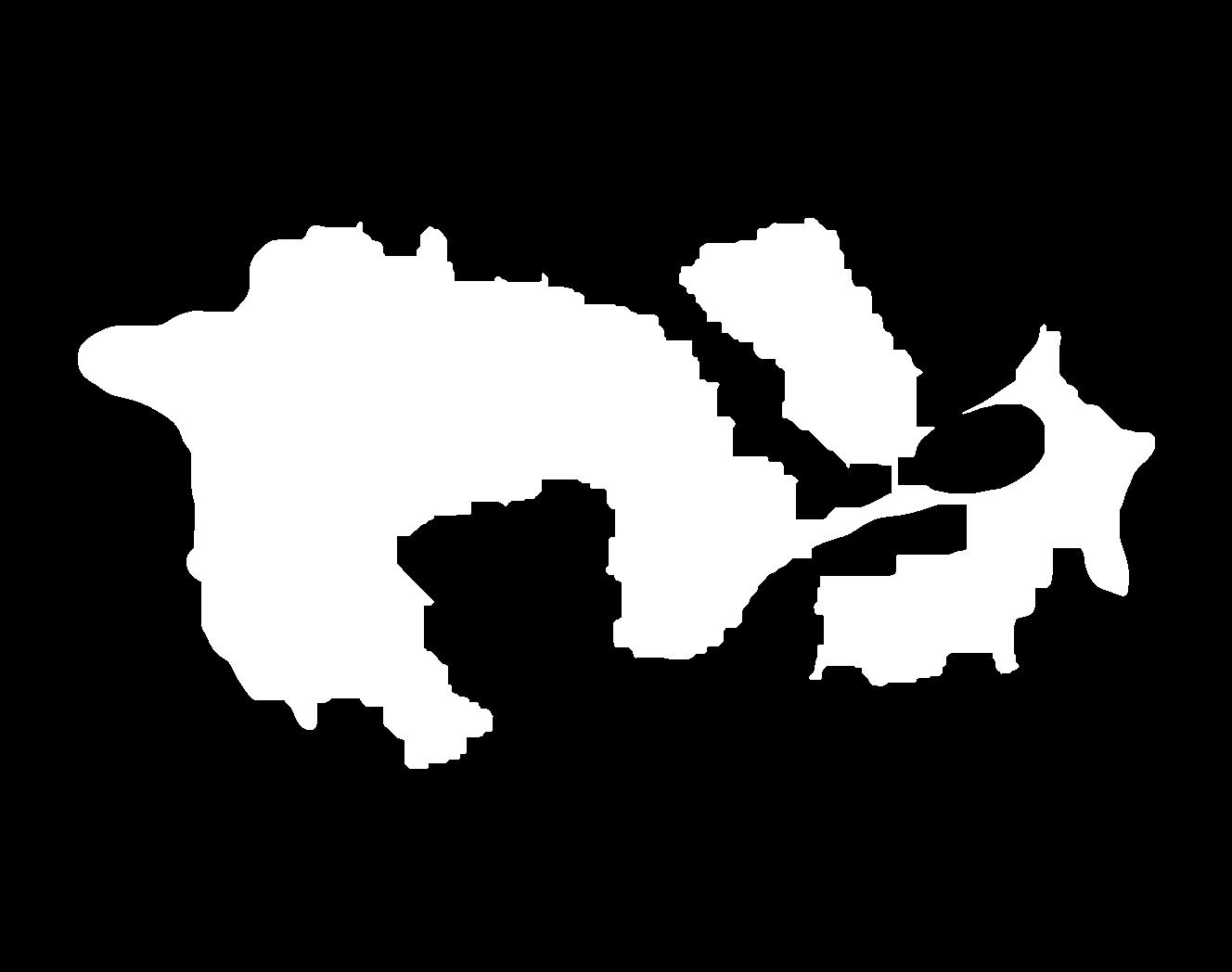

Supplement: S5 File — (ZIP) [file pone.0237972.s005.zip › S3_File IoU scores/masks/Experiment_1/cell/user_segmented/Automated/Automated_Participant6_mask_cell_D.jpg]

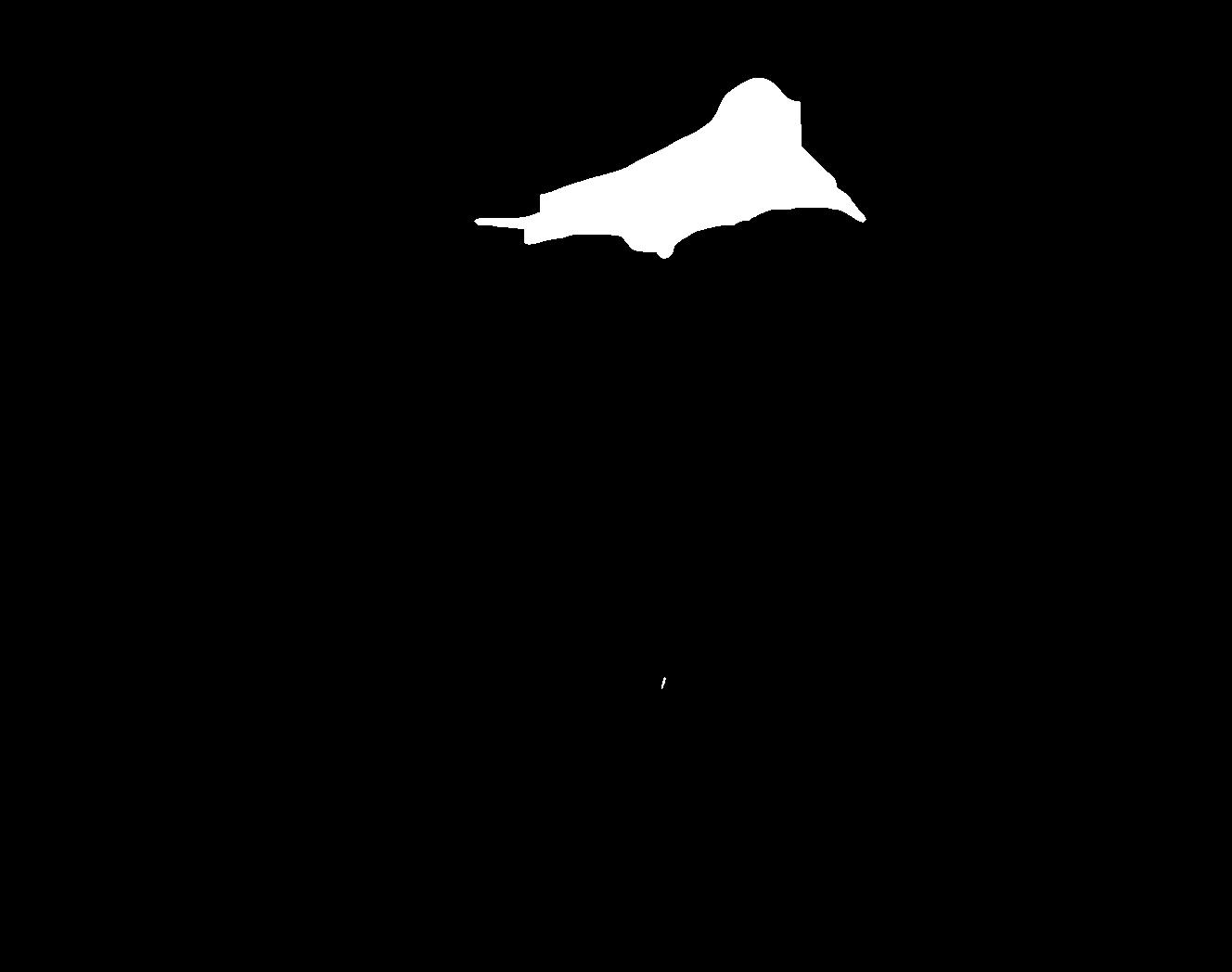

Supplement: S5 File — (ZIP) [file pone.0237972.s005.zip › S3_File IoU scores/masks/Experiment_1/cell/user_segmented/Automated/Automated_Participant6_mask_cell_E.jpg]

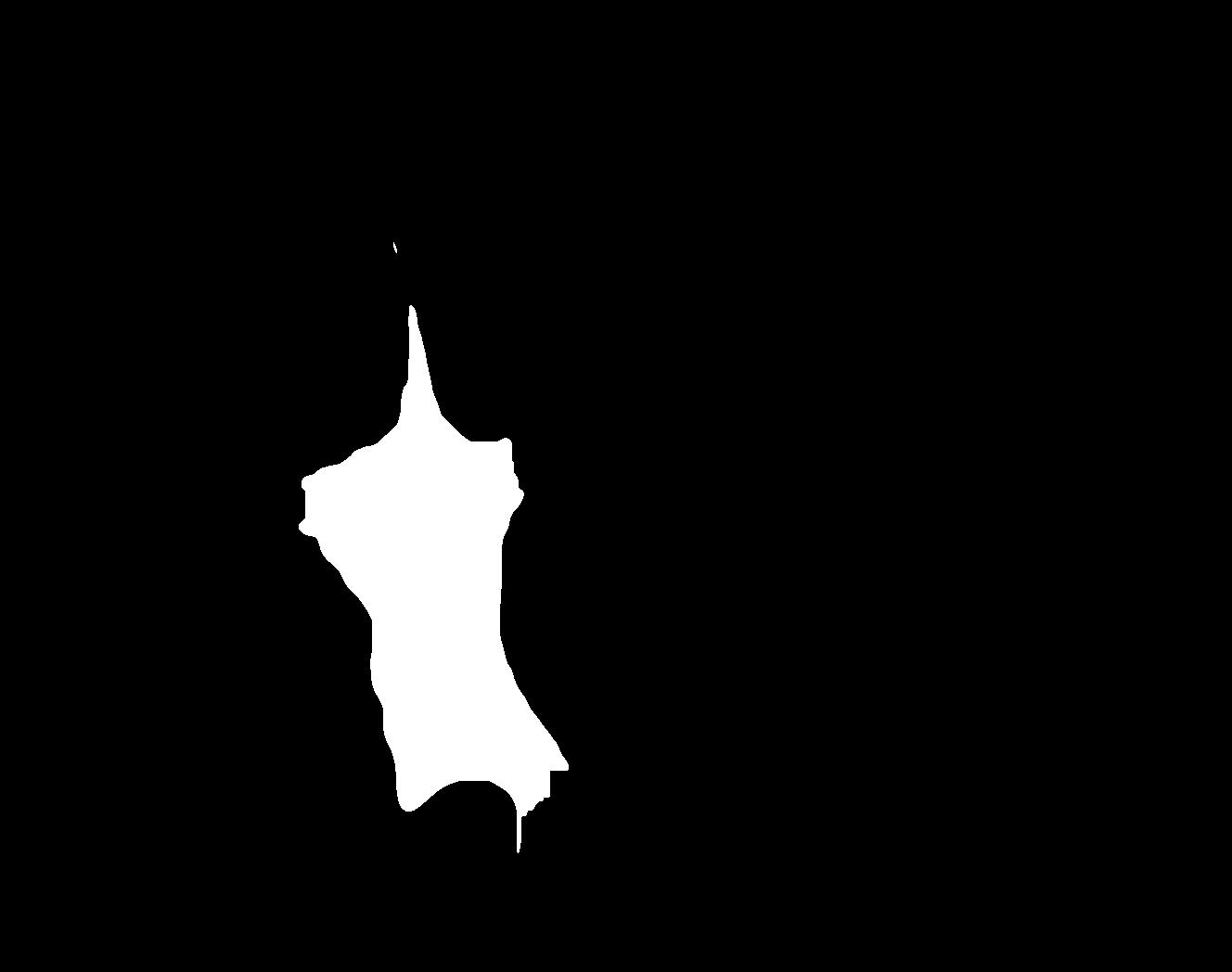

Supplement: S5 File — (ZIP) [file pone.0237972.s005.zip › S3_File IoU scores/masks/Experiment_1/cell/user_segmented/Automated/Automated_Participant6_mask_cell_F.jpg]

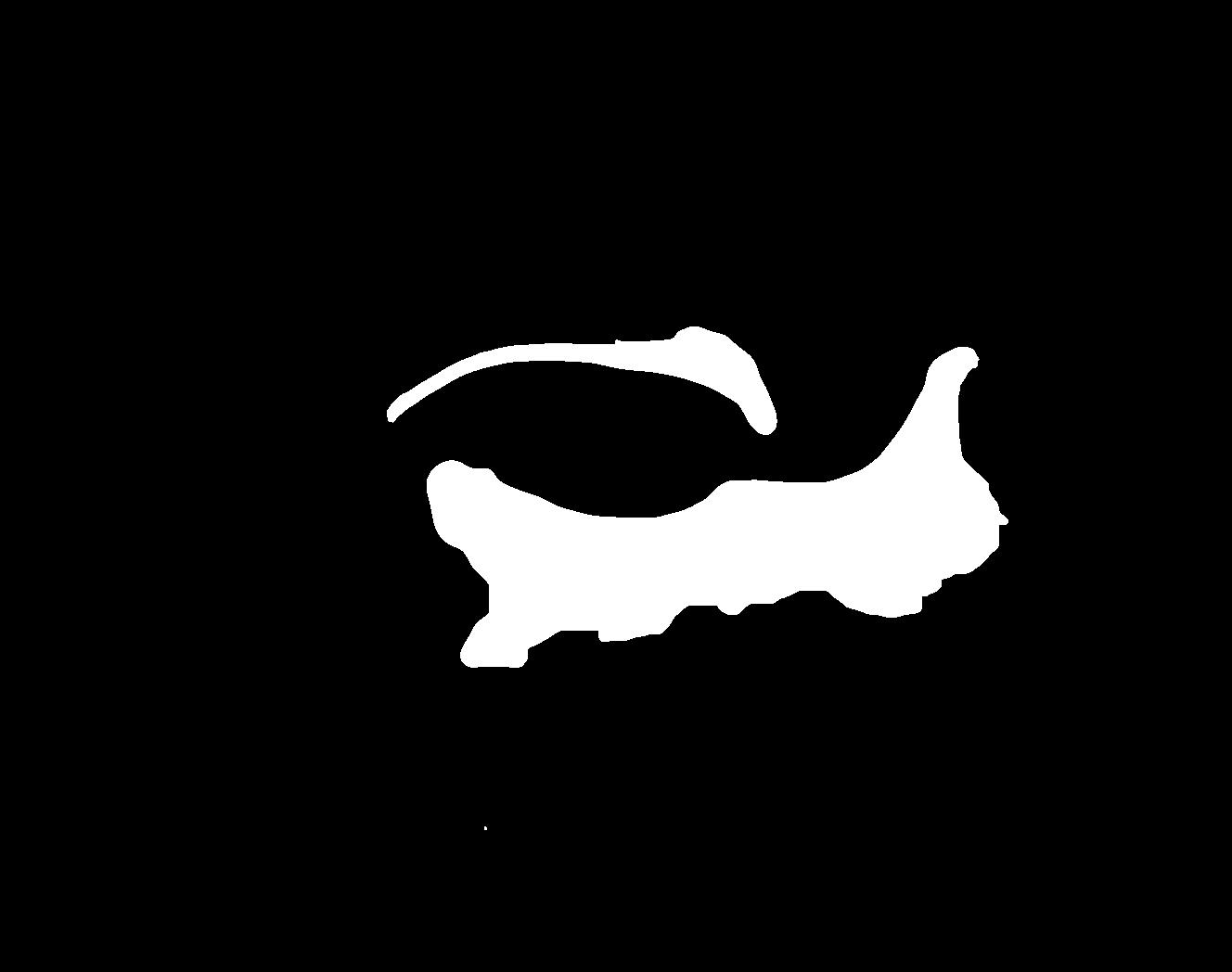

Supplement: S5 File — (ZIP) [file pone.0237972.s005.zip › S3_File IoU scores/masks/Experiment_1/cell/user_segmented/Automated/Automated_Participant6_mask_cell_G.jpg]

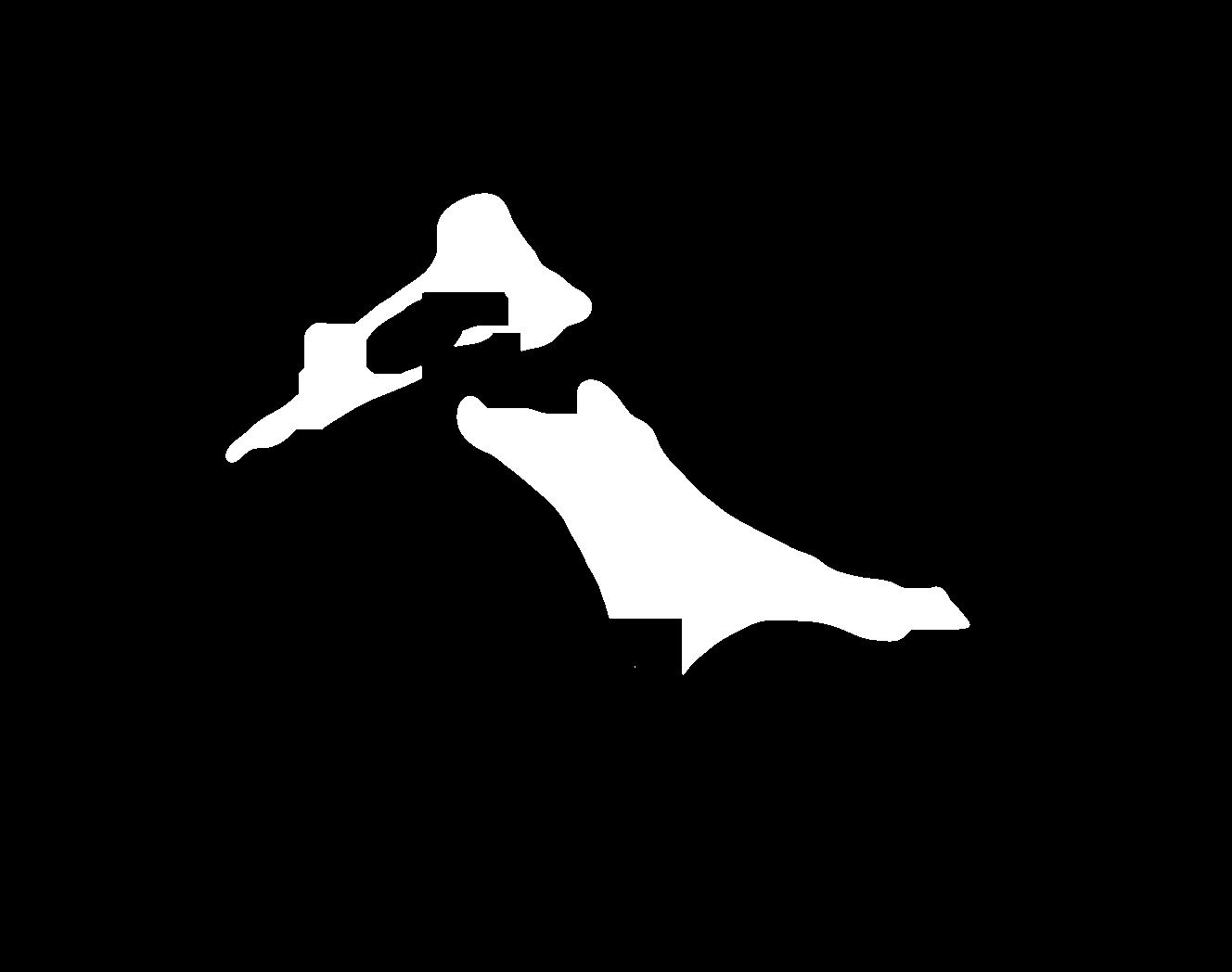

Supplement: S5 File — (ZIP) [file pone.0237972.s005.zip › S3_File IoU scores/masks/Experiment_1/cell/user_segmented/Automated/Automated_Participant6_mask_cell_H.jpg]

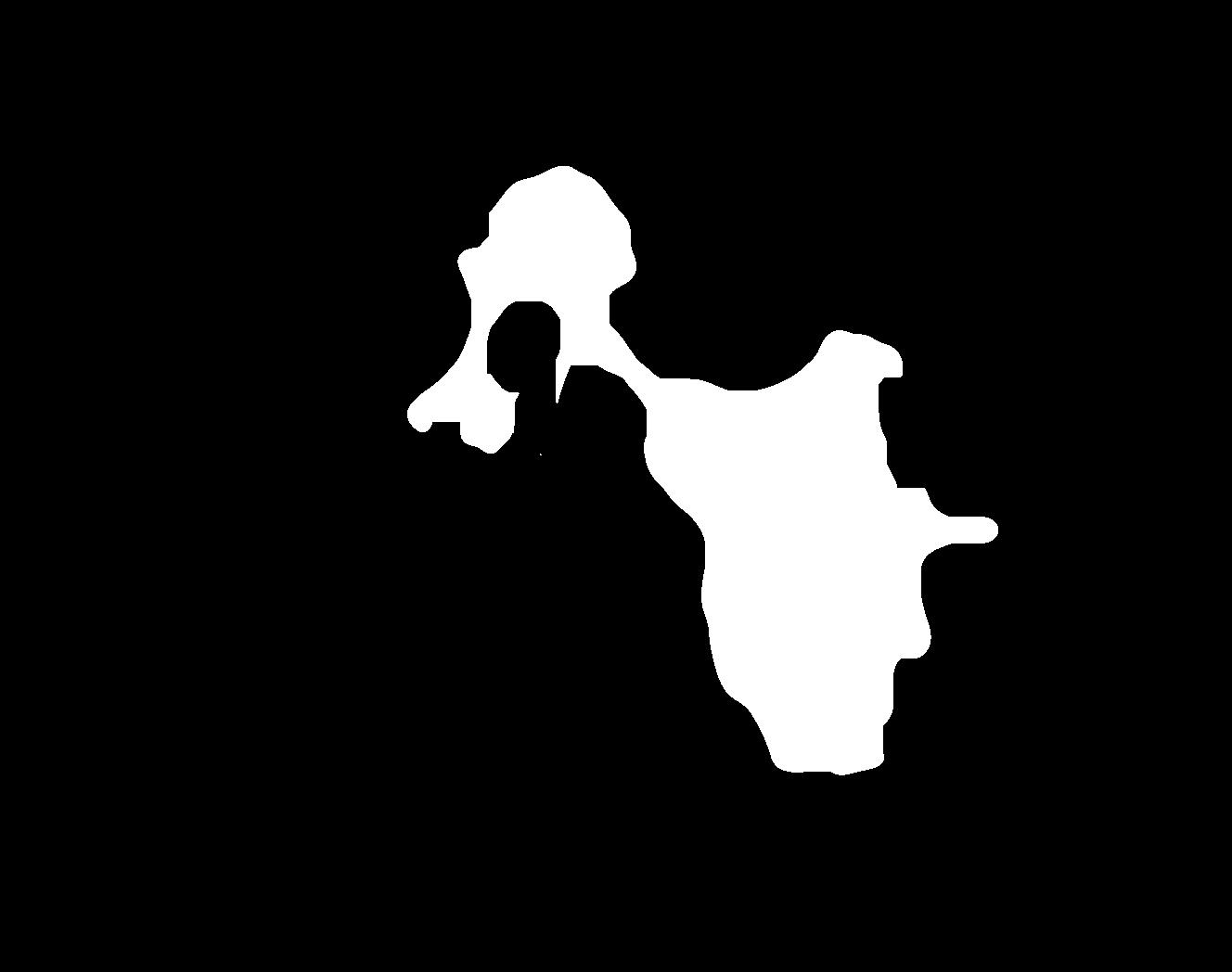

Supplement: S5 File — (ZIP) [file pone.0237972.s005.zip › S3_File IoU scores/masks/Experiment_1/cell/user_segmented/Automated/Automated_Participant6_mask_cell_I.jpg]

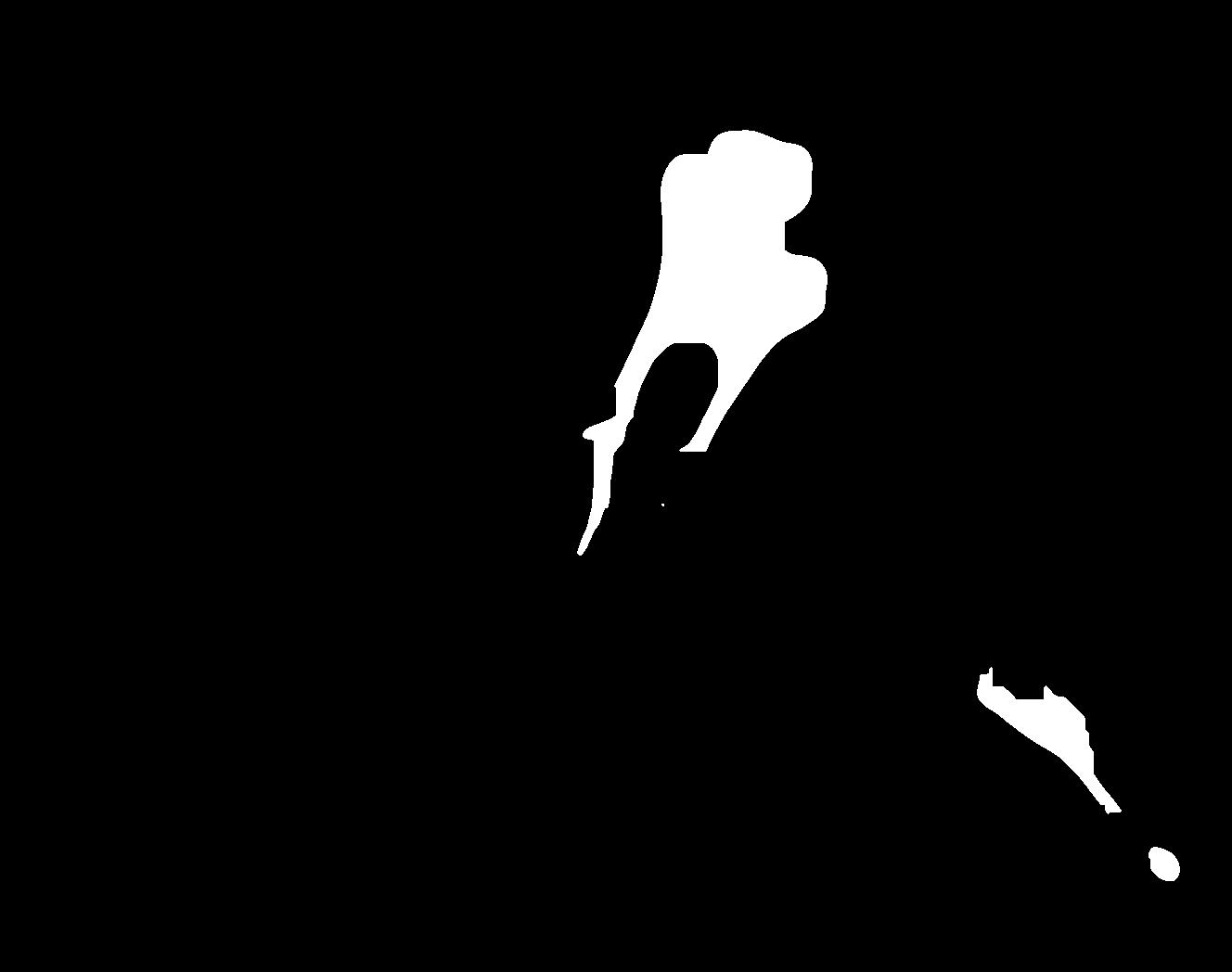

Supplement: S5 File — (ZIP) [file pone.0237972.s005.zip › S3_File IoU scores/masks/Experiment_1/cell/user_segmented/Automated/Automated_Participant6_mask_cell_J.jpg]

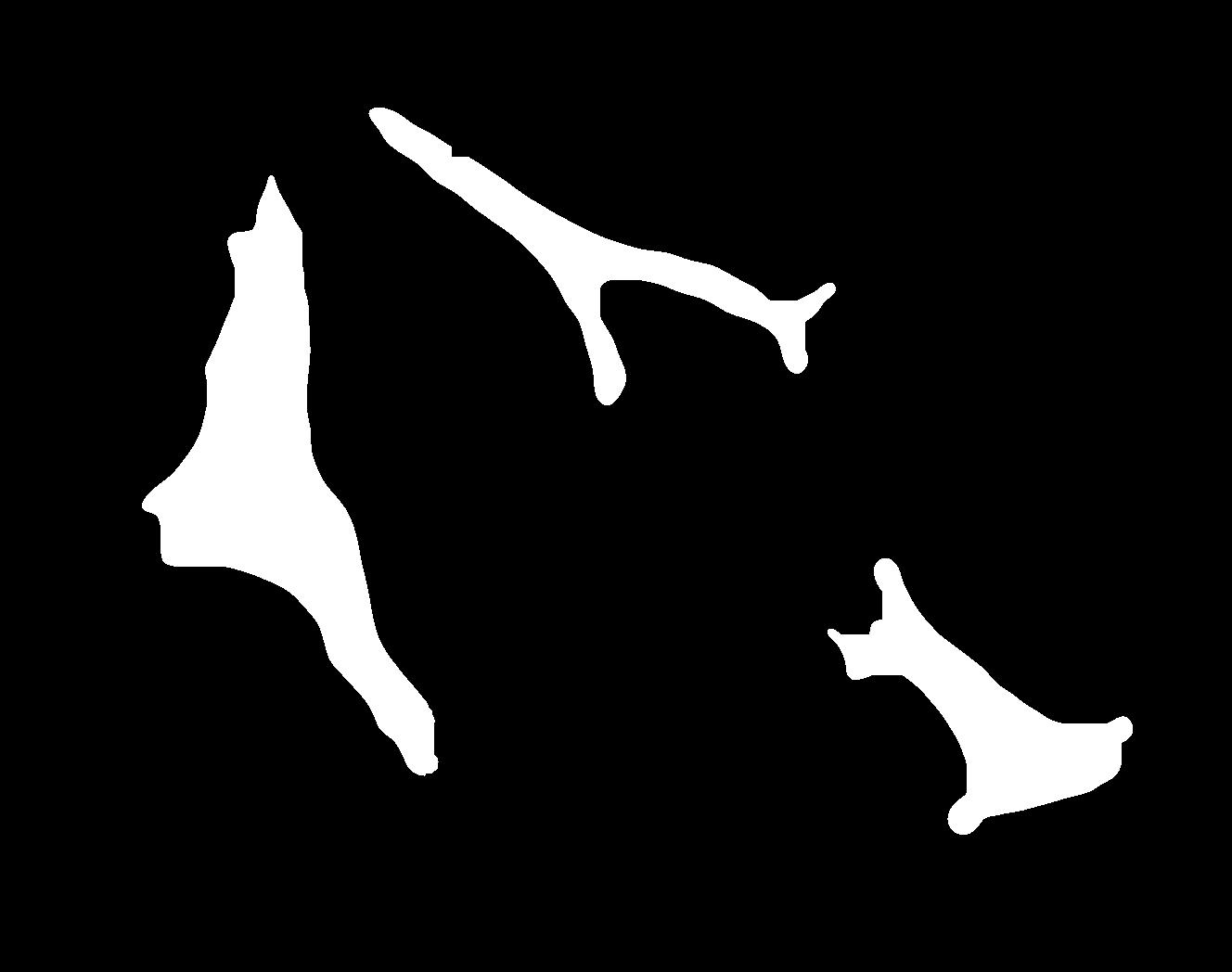

Supplement: S5 File — (ZIP) [file pone.0237972.s005.zip › S3_File IoU scores/masks/Experiment_1/cell/user_segmented/Composite/Composite_Participant10_mask_cell_A.jpg]

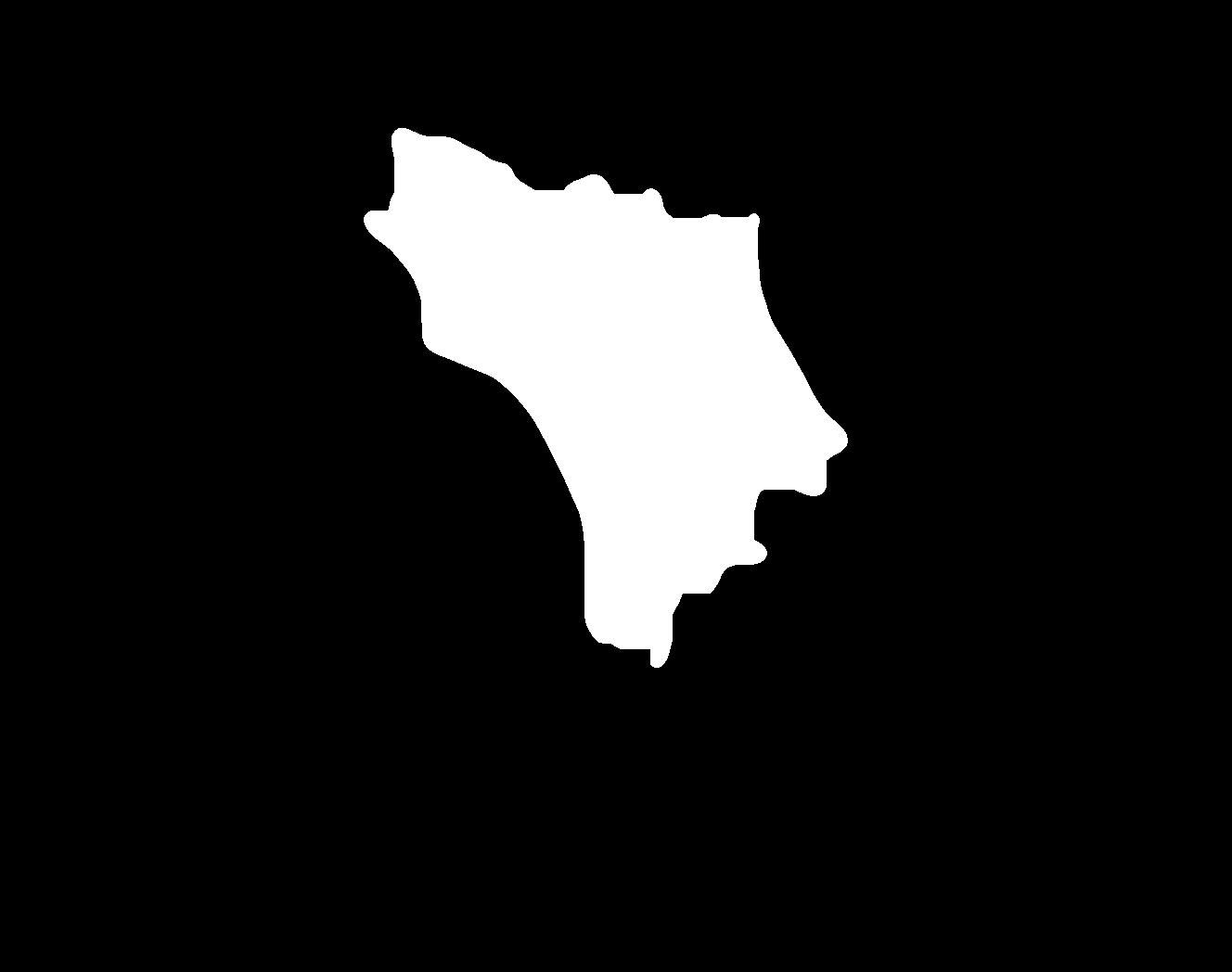

Supplement: S5 File — (ZIP) [file pone.0237972.s005.zip › S3_File IoU scores/masks/Experiment_1/cell/user_segmented/Composite/Composite_Participant10_mask_cell_B.jpg]

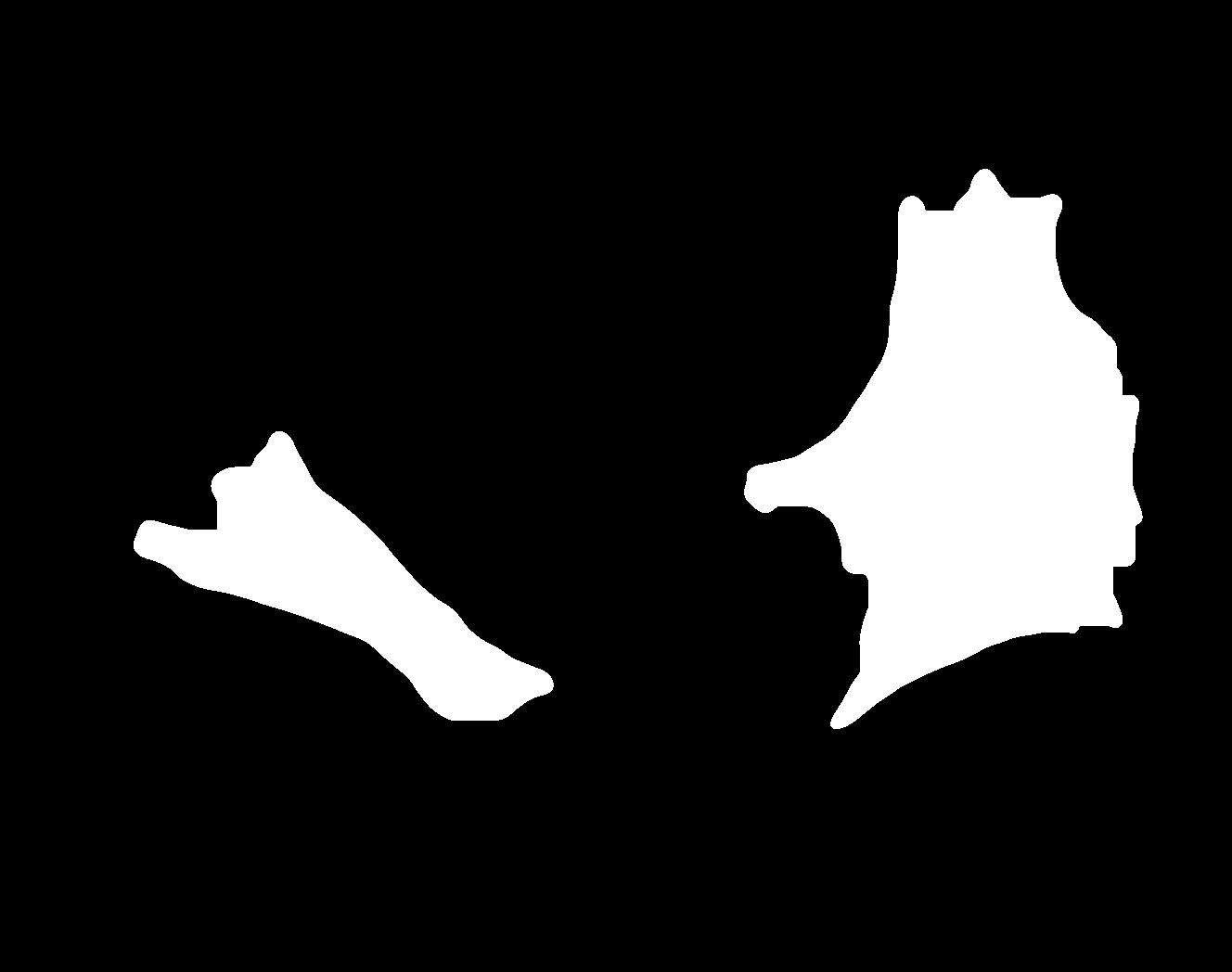

Supplement: S5 File — (ZIP) [file pone.0237972.s005.zip › S3_File IoU scores/masks/Experiment_1/cell/user_segmented/Composite/Composite_Participant10_mask_cell_C.jpg]

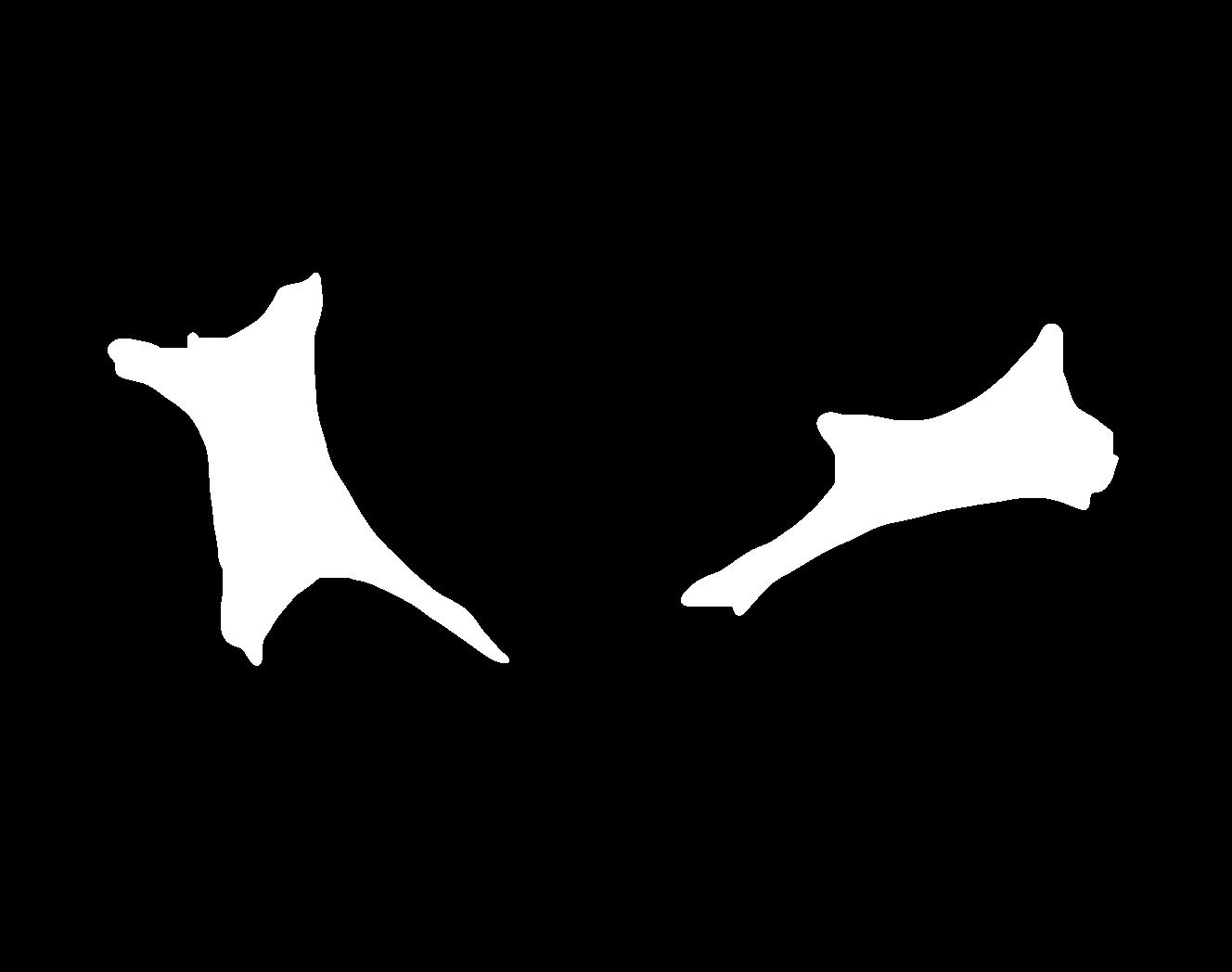

Supplement: S5 File — (ZIP) [file pone.0237972.s005.zip › S3_File IoU scores/masks/Experiment_1/cell/user_segmented/Composite/Composite_Participant10_mask_cell_D.jpg]

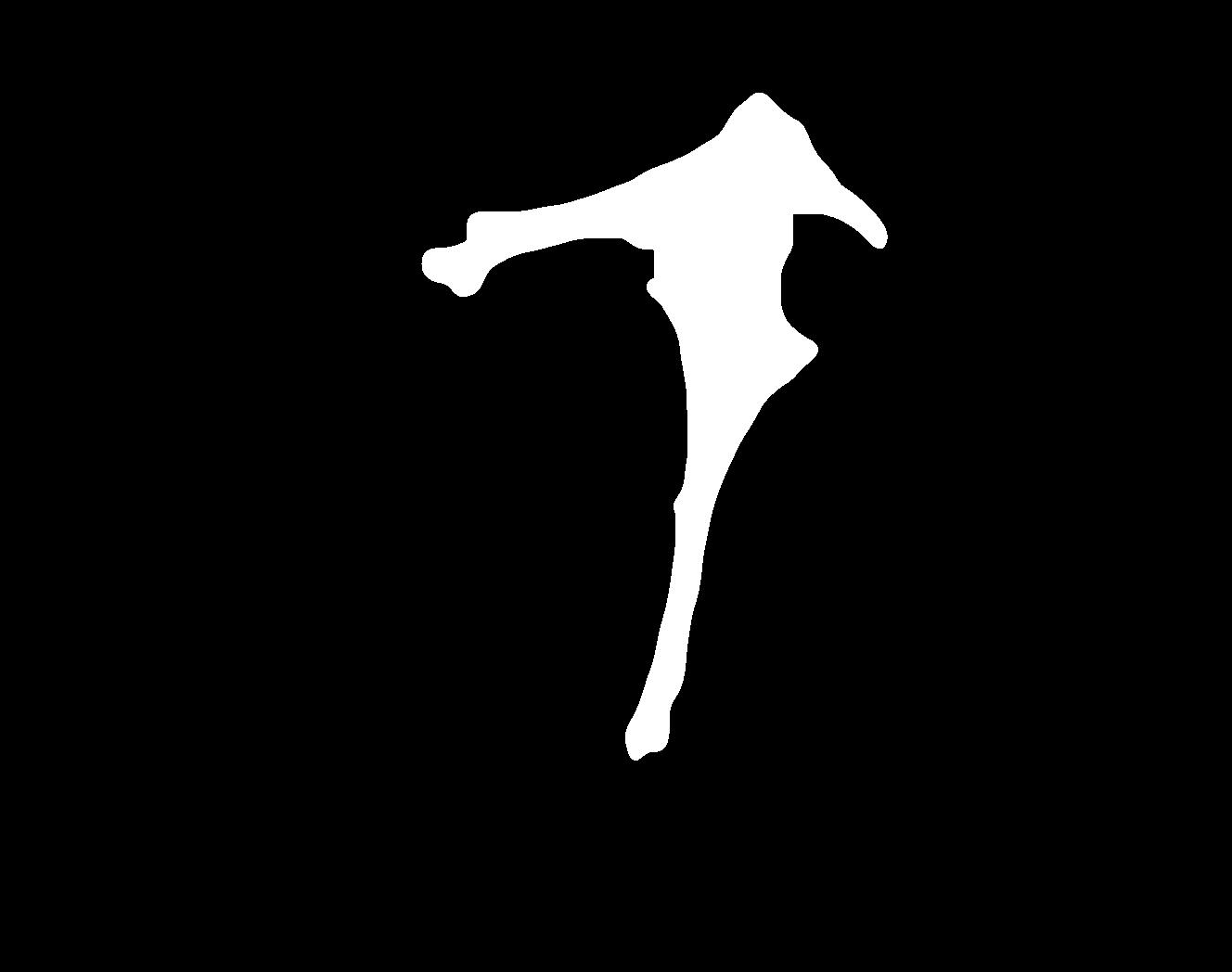

Supplement: S5 File — (ZIP) [file pone.0237972.s005.zip › S3_File IoU scores/masks/Experiment_1/cell/user_segmented/Composite/Composite_Participant10_mask_cell_E.jpg]

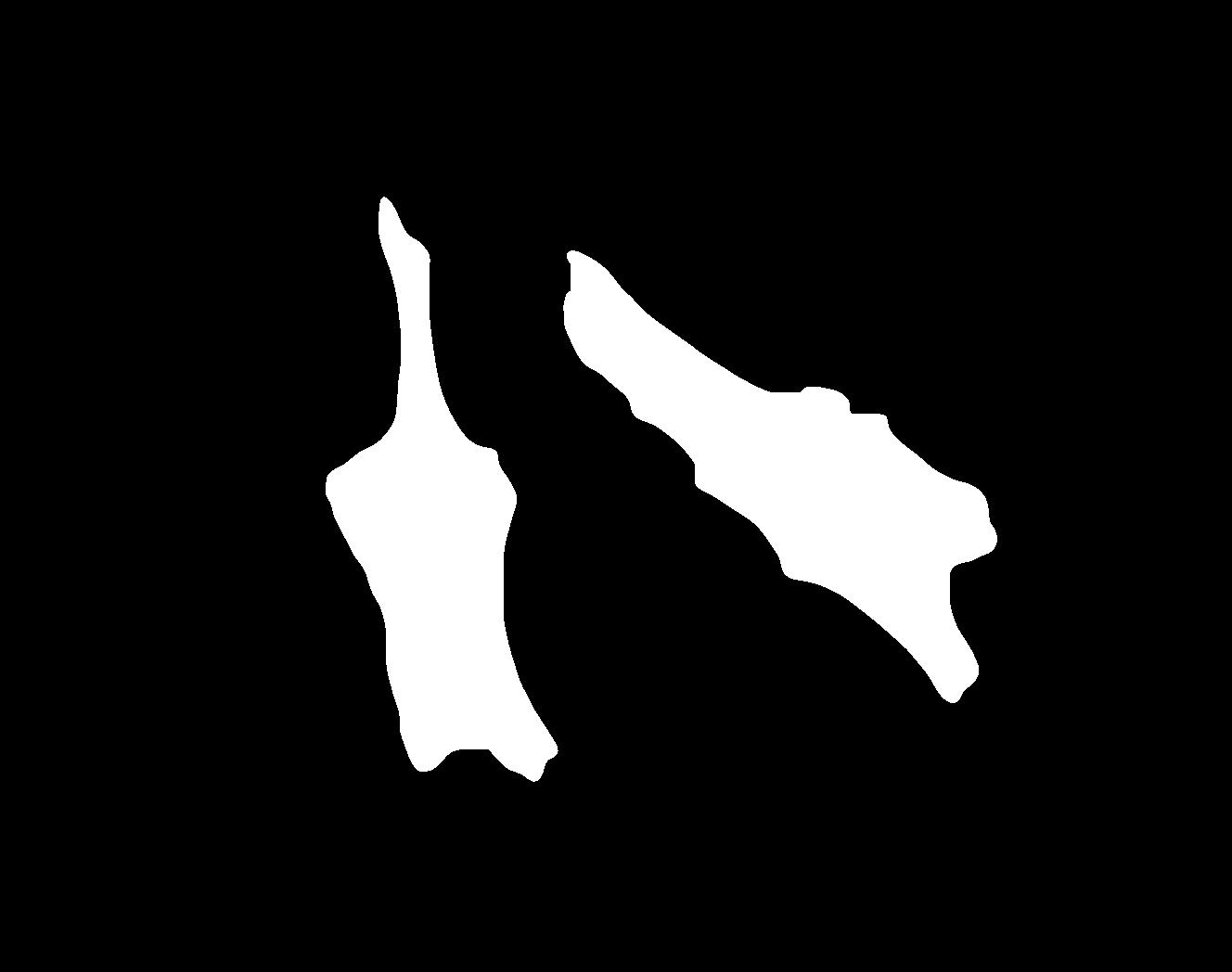

Supplement: S5 File — (ZIP) [file pone.0237972.s005.zip › S3_File IoU scores/masks/Experiment_1/cell/user_segmented/Composite/Composite_Participant10_mask_cell_F.jpg]

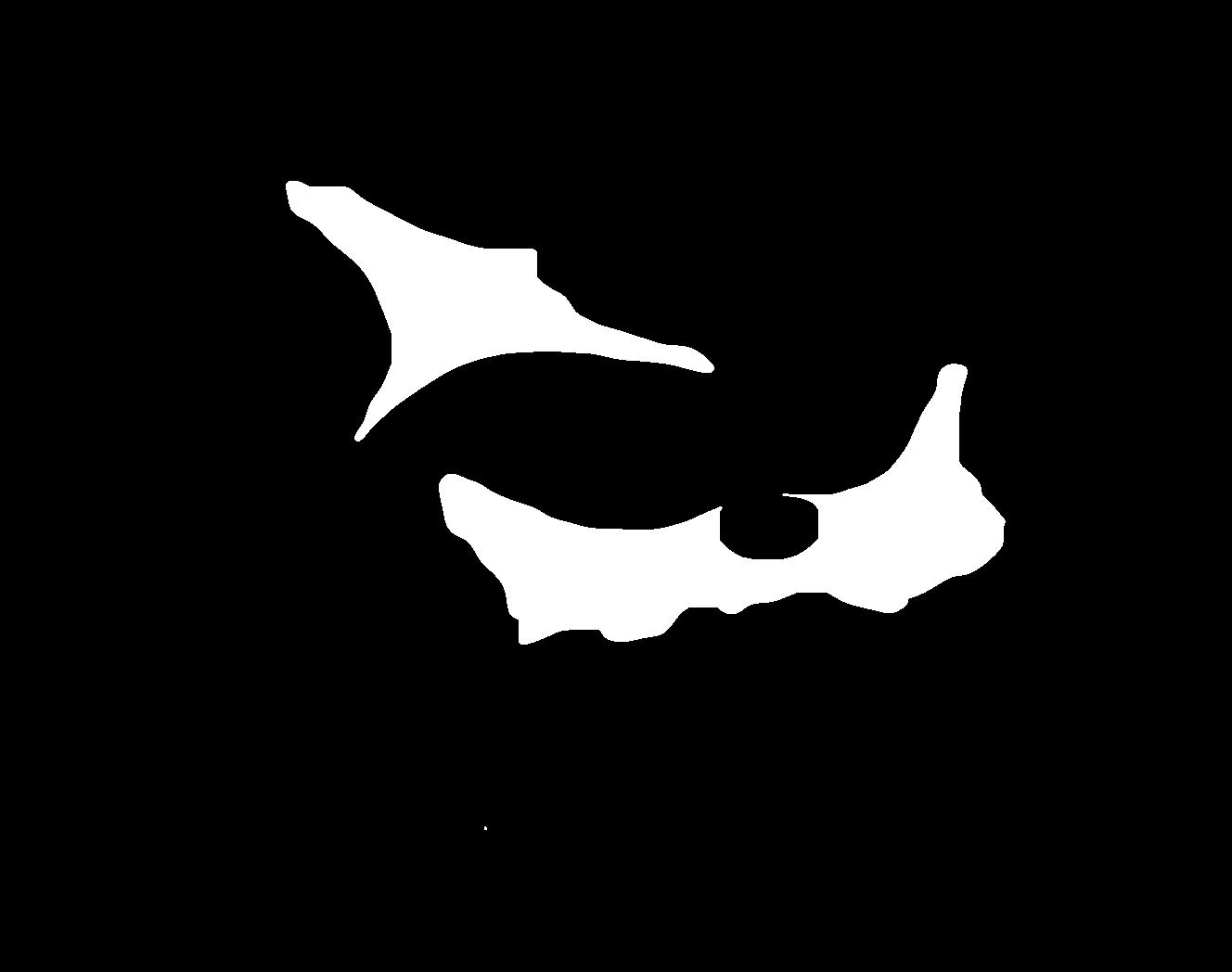

Supplement: S5 File — (ZIP) [file pone.0237972.s005.zip › S3_File IoU scores/masks/Experiment_1/cell/user_segmented/Composite/Composite_Participant10_mask_cell_G.jpg]

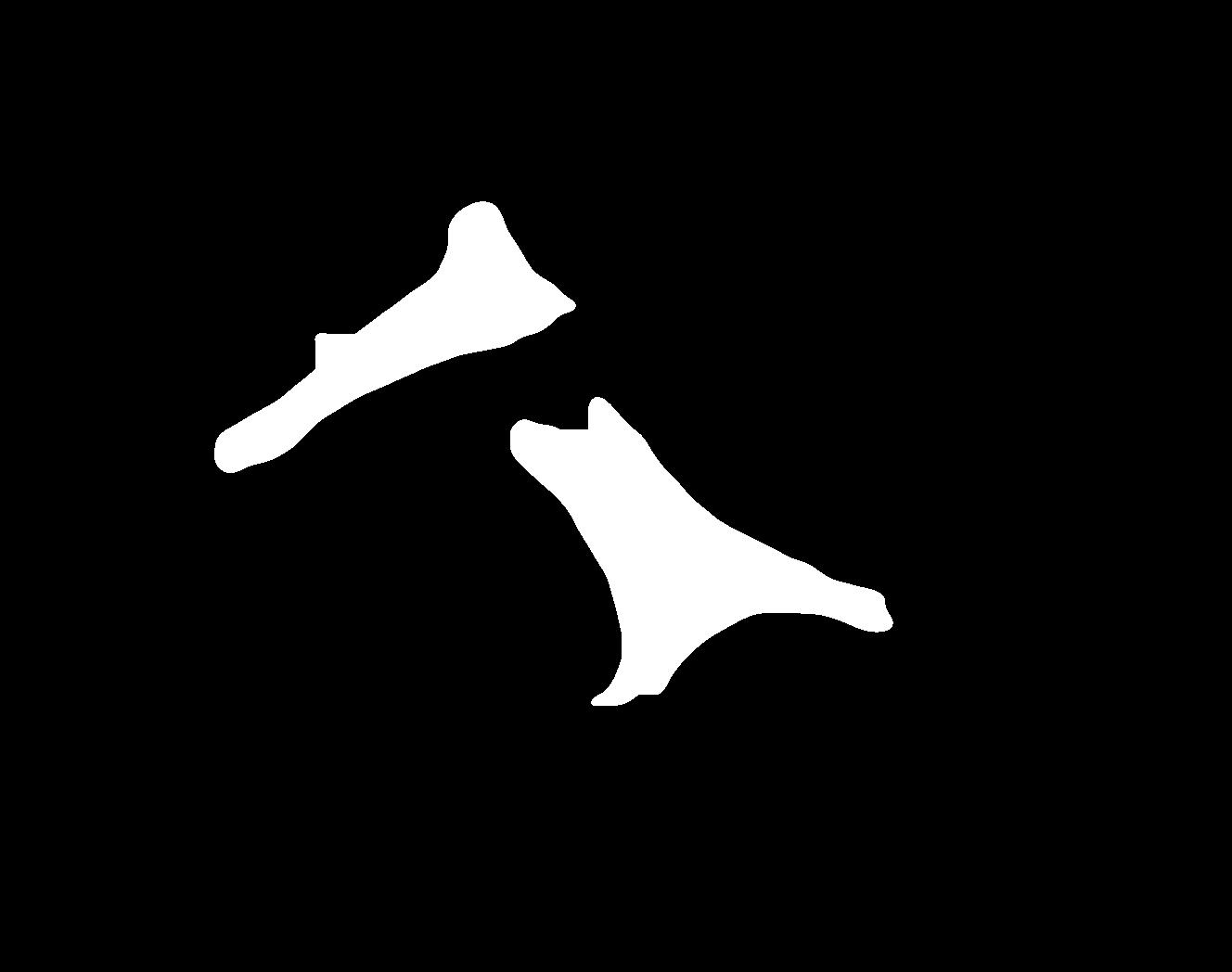

Supplement: S5 File — (ZIP) [file pone.0237972.s005.zip › S3_File IoU scores/masks/Experiment_1/cell/user_segmented/Composite/Composite_Participant10_mask_cell_H.jpg]

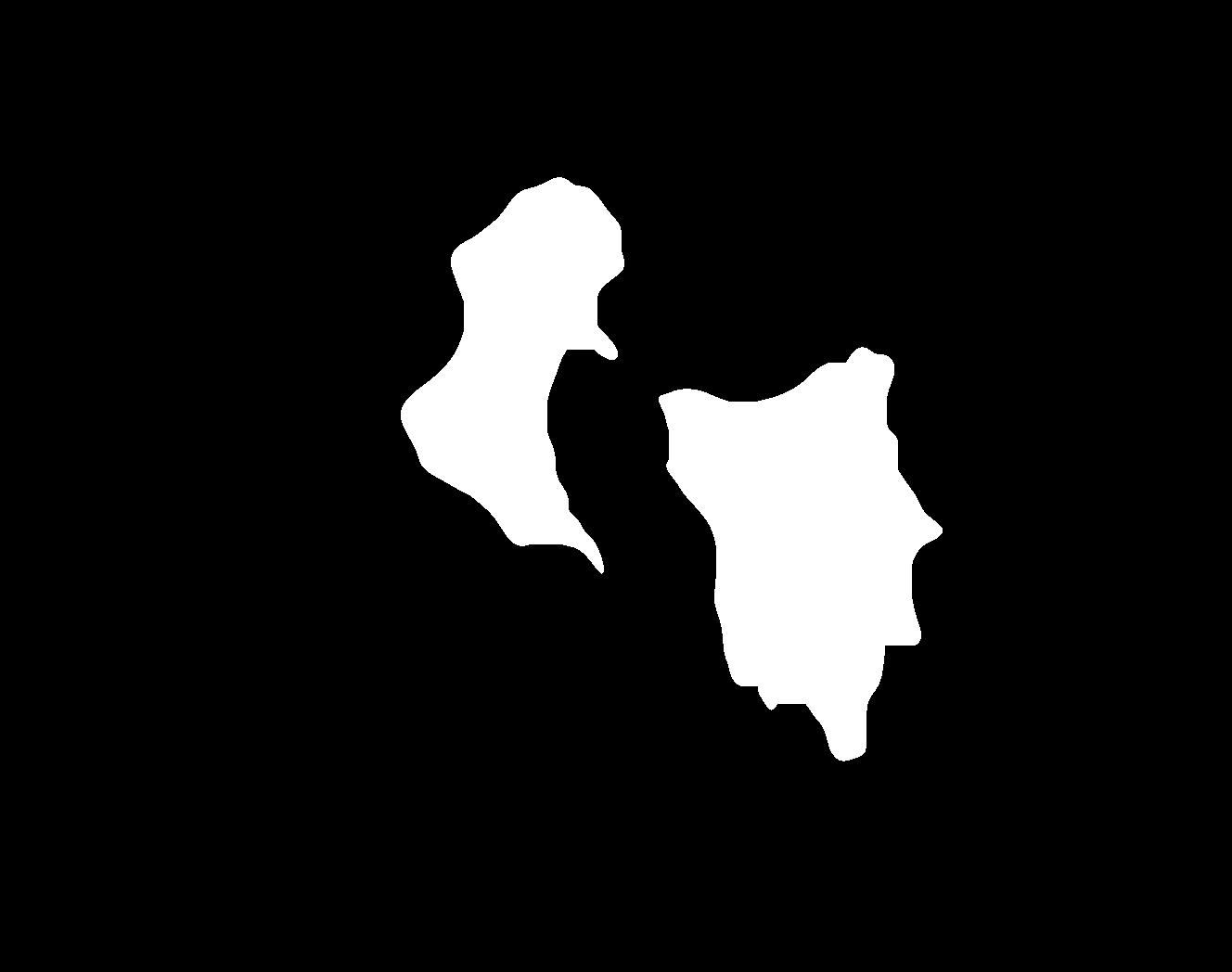

Supplement: S5 File — (ZIP) [file pone.0237972.s005.zip › S3_File IoU scores/masks/Experiment_1/cell/user_segmented/Composite/Composite_Participant10_mask_cell_I.jpg]

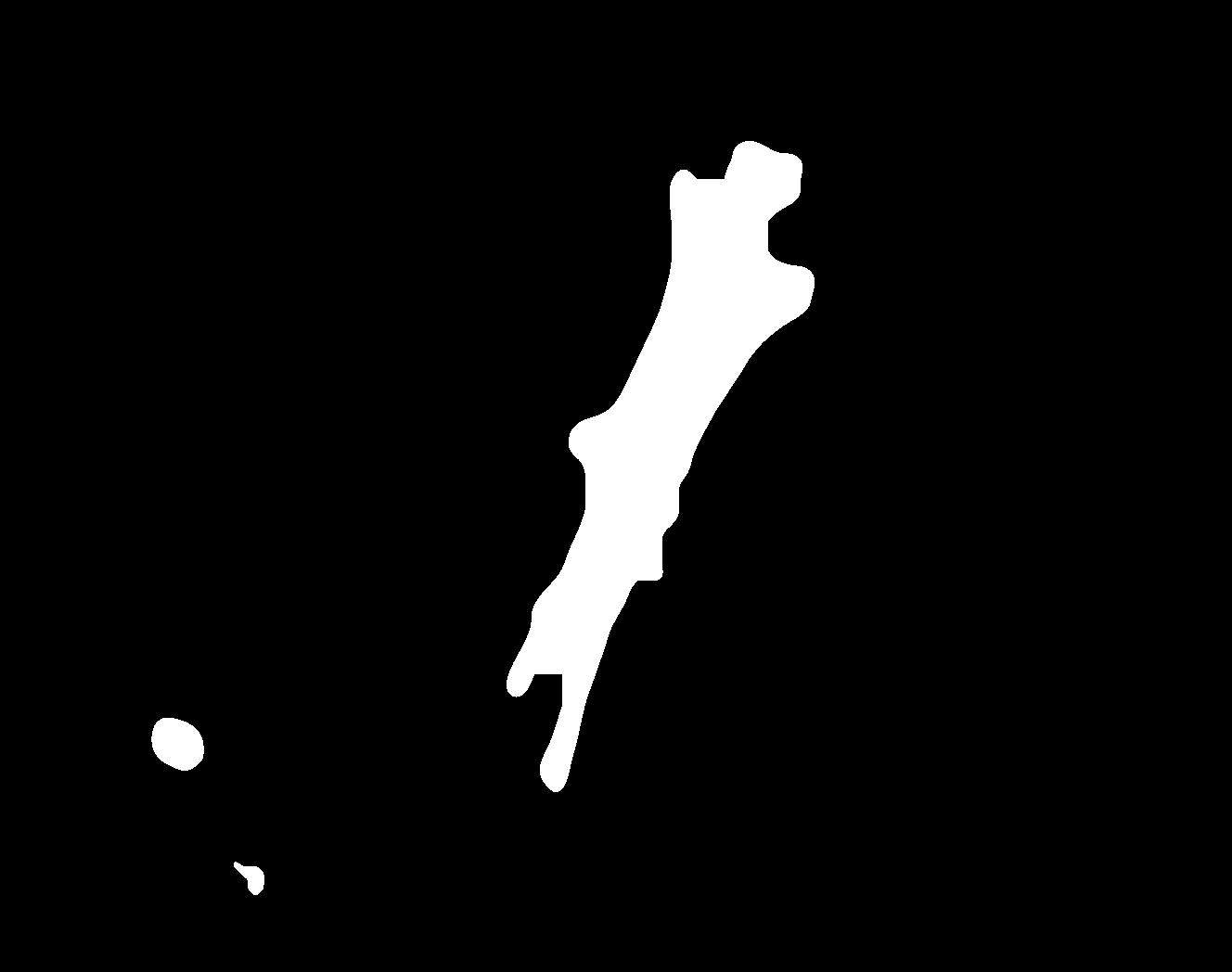

Supplement: S5 File — (ZIP) [file pone.0237972.s005.zip › S3_File IoU scores/masks/Experiment_1/cell/user_segmented/Composite/Composite_Participant10_mask_cell_J.jpg]

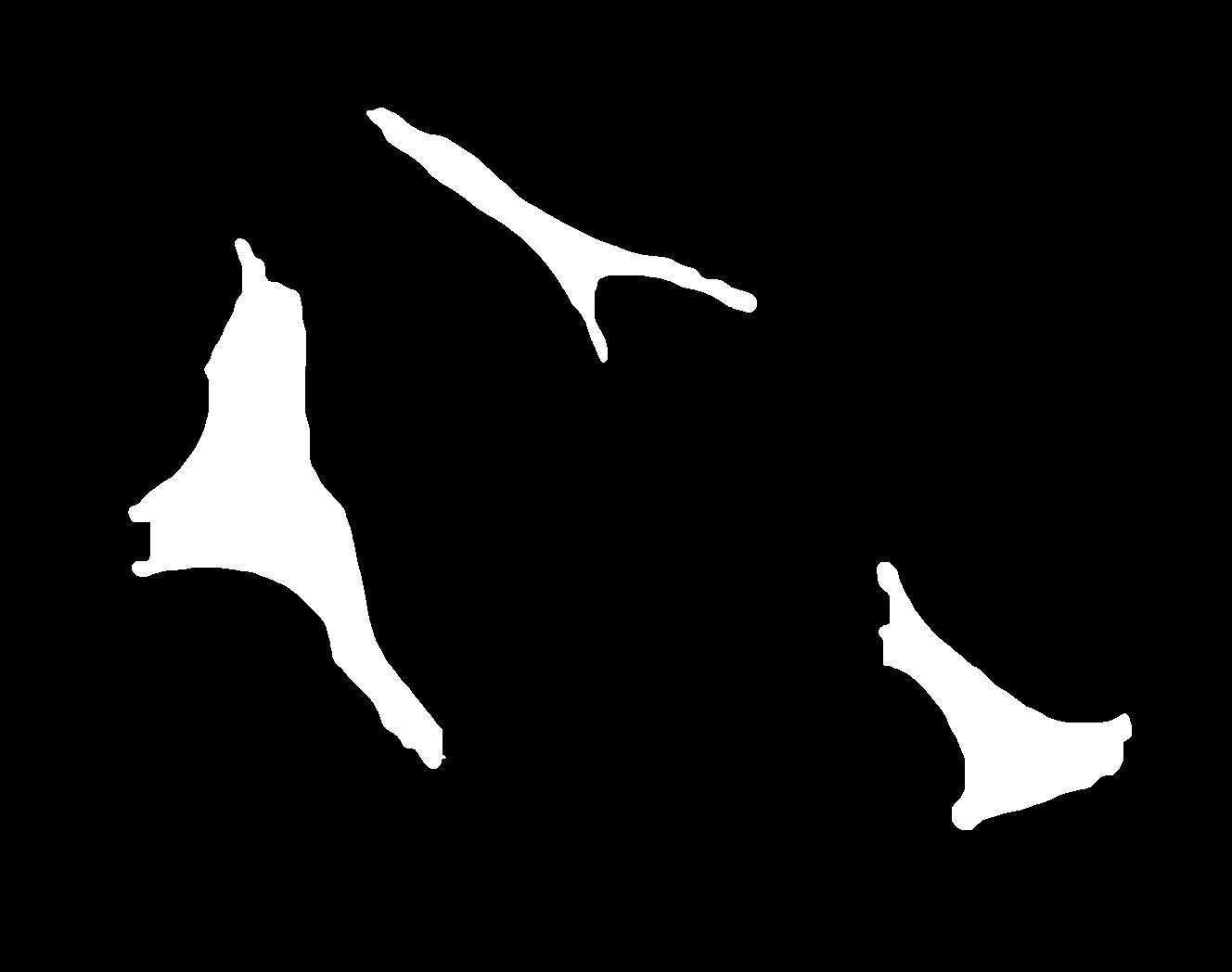

Supplement: S5 File — (ZIP) [file pone.0237972.s005.zip › S3_File IoU scores/masks/Experiment_1/cell/user_segmented/Composite/Composite_Participant13_mask_cell_A.jpg]

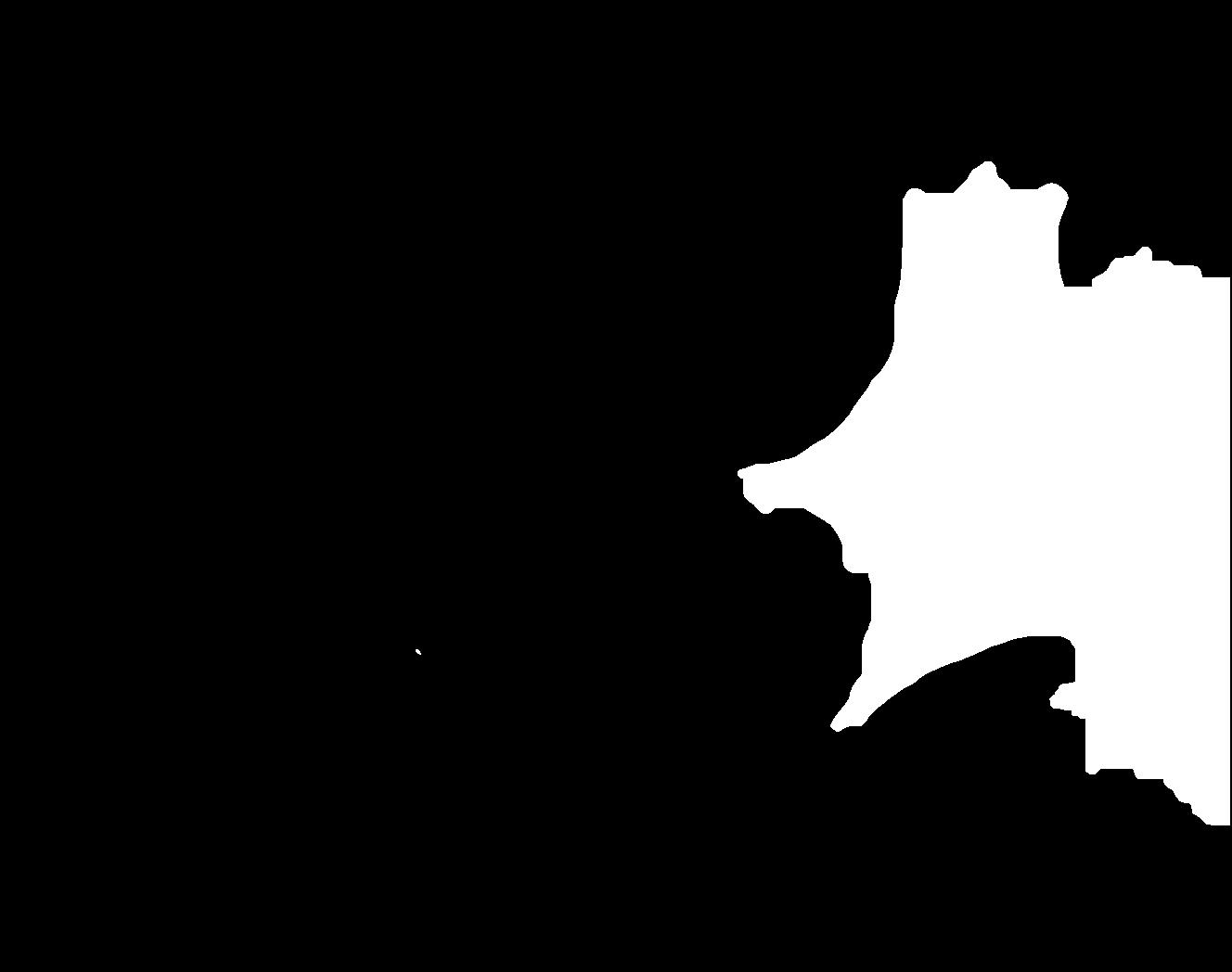

Supplement: S5 File — (ZIP) [file pone.0237972.s005.zip › S3_File IoU scores/masks/Experiment_1/cell/user_segmented/Composite/Composite_Participant13_mask_cell_C.jpg]

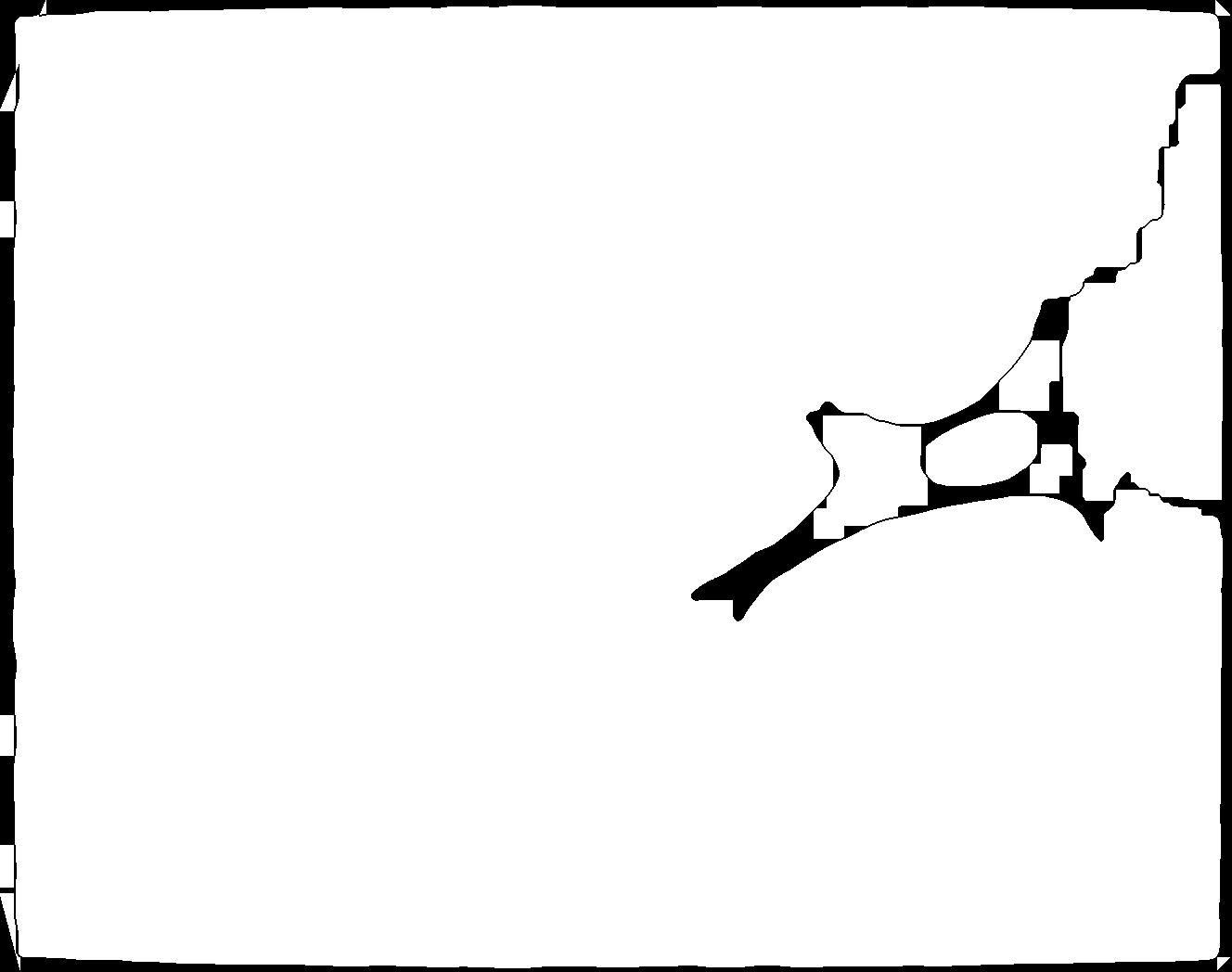

Supplement: S5 File — (ZIP) [file pone.0237972.s005.zip › S3_File IoU scores/masks/Experiment_1/cell/user_segmented/Composite/Composite_Participant13_mask_cell_D.jpg]

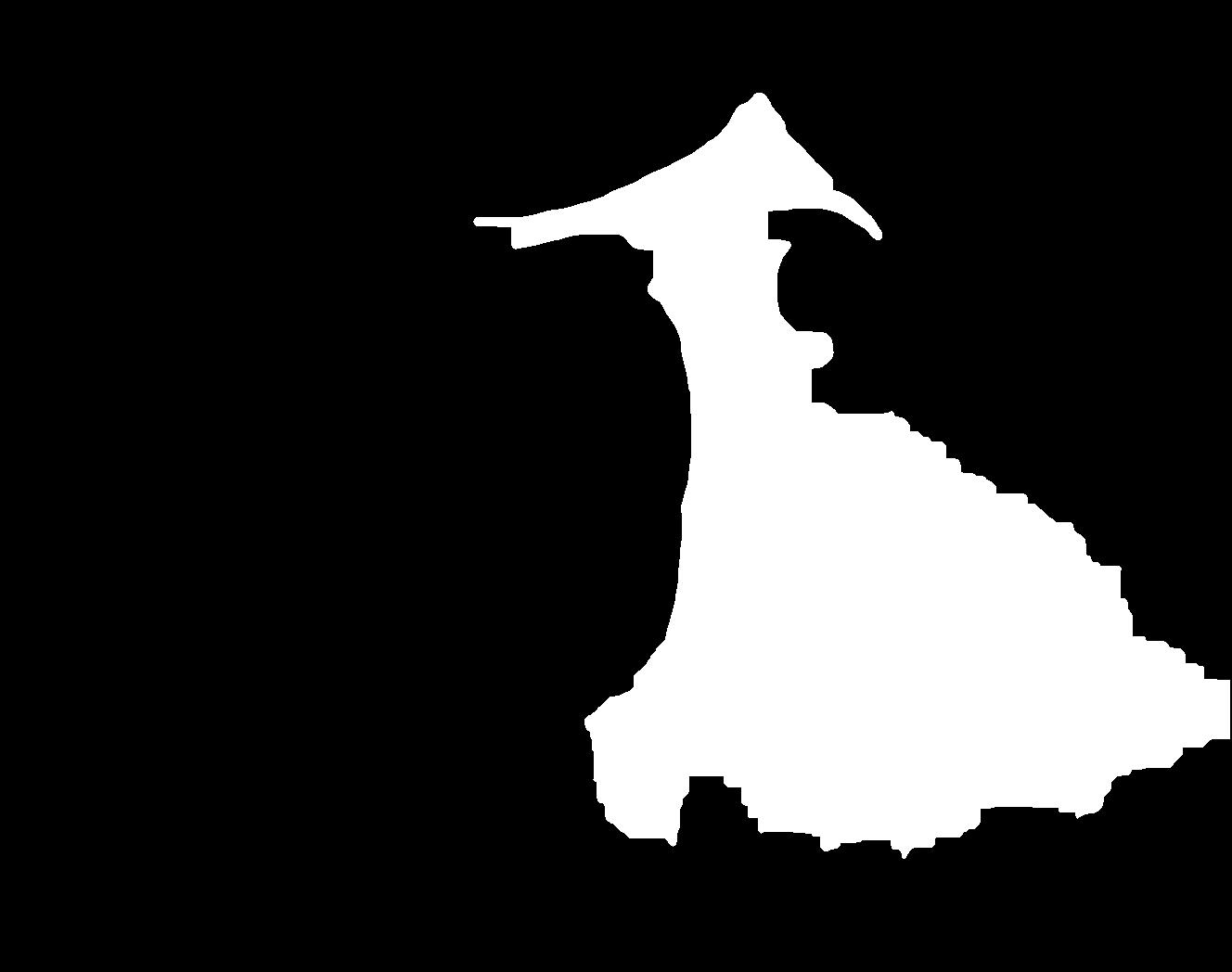

Supplement: S5 File — (ZIP) [file pone.0237972.s005.zip › S3_File IoU scores/masks/Experiment_1/cell/user_segmented/Composite/Composite_Participant13_mask_cell_E.jpg]

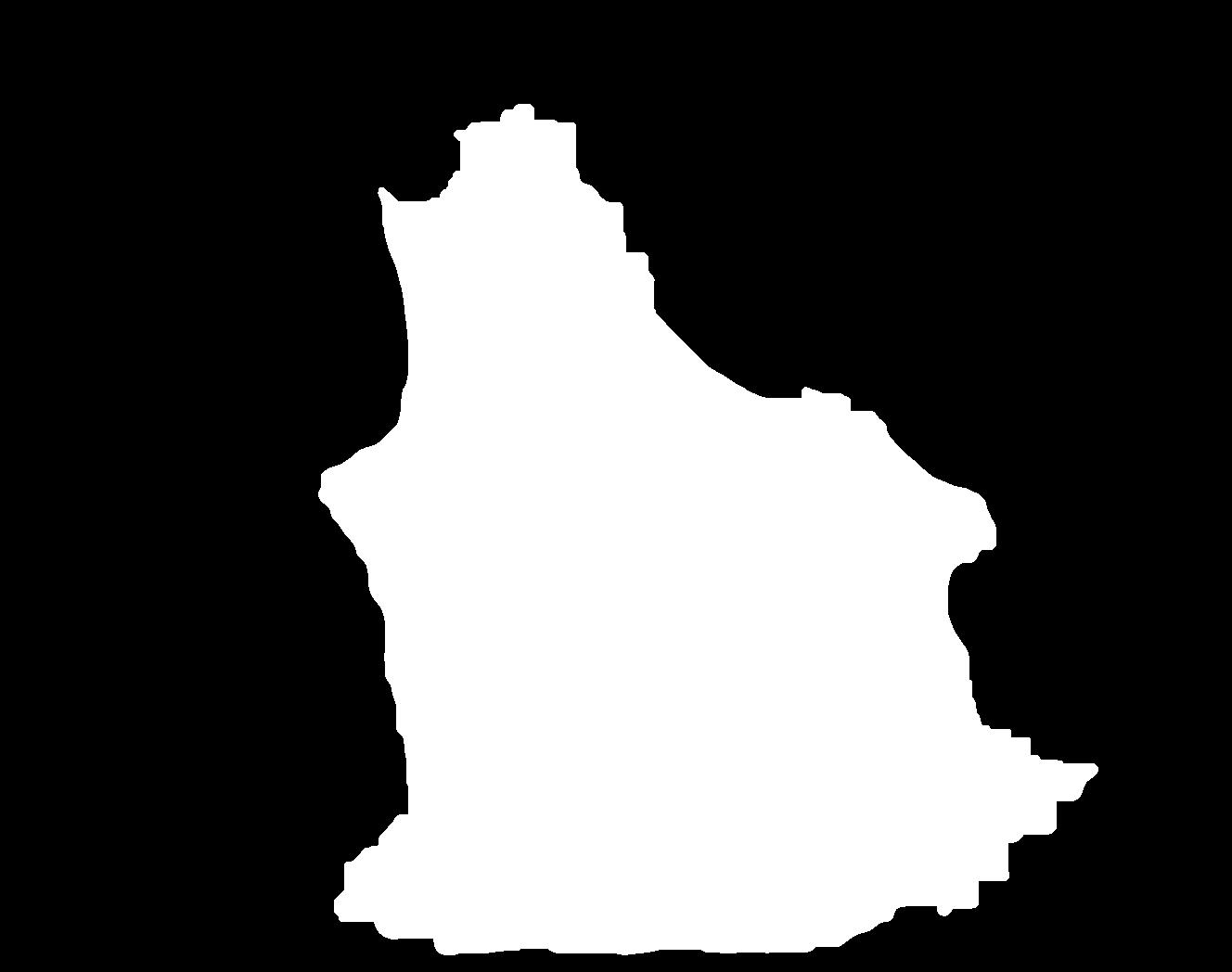

Supplement: S5 File — (ZIP) [file pone.0237972.s005.zip › S3_File IoU scores/masks/Experiment_1/cell/user_segmented/Composite/Composite_Participant13_mask_cell_F.jpg]

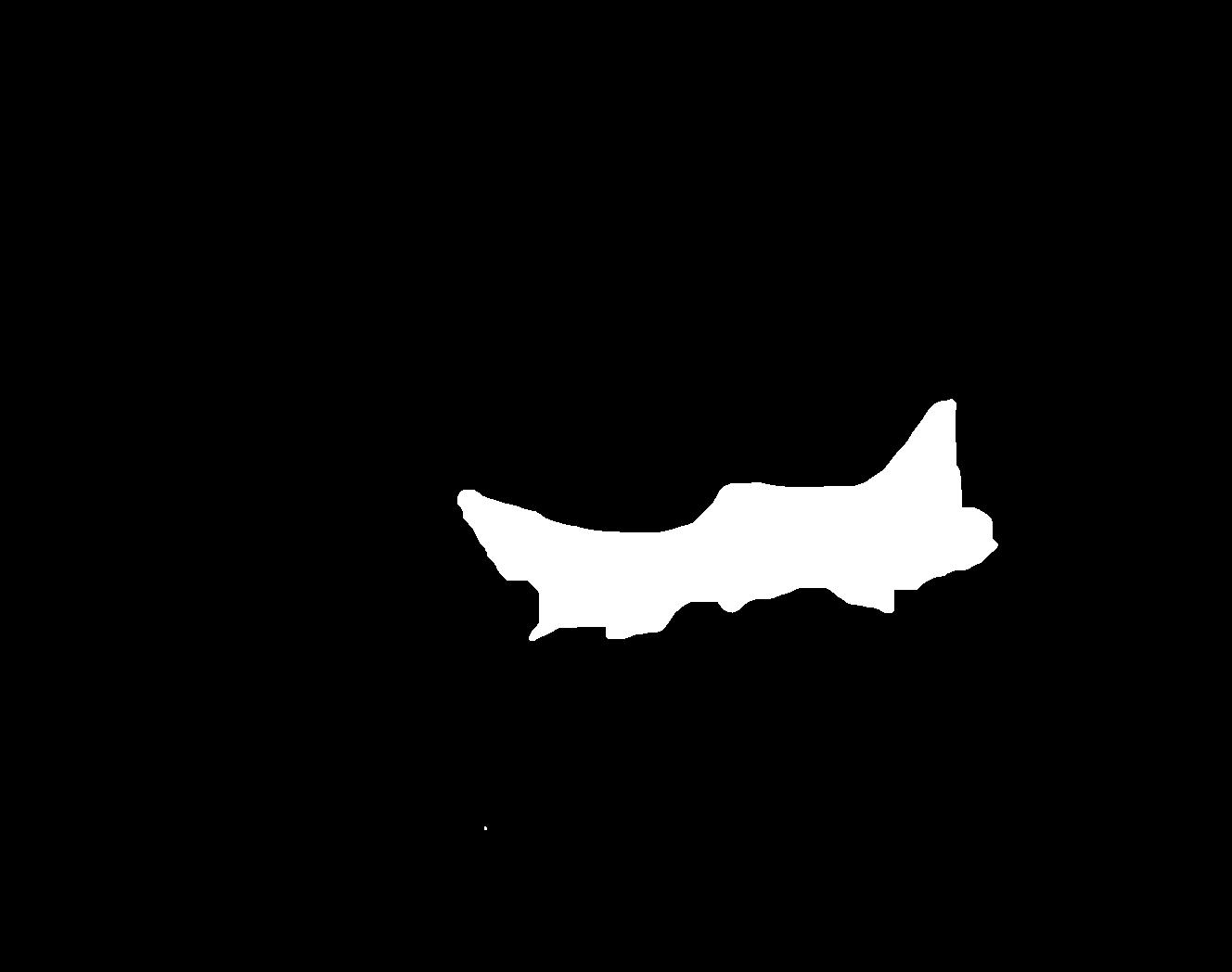

Supplement: S5 File — (ZIP) [file pone.0237972.s005.zip › S3_File IoU scores/masks/Experiment_1/cell/user_segmented/Composite/Composite_Participant13_mask_cell_G.jpg]

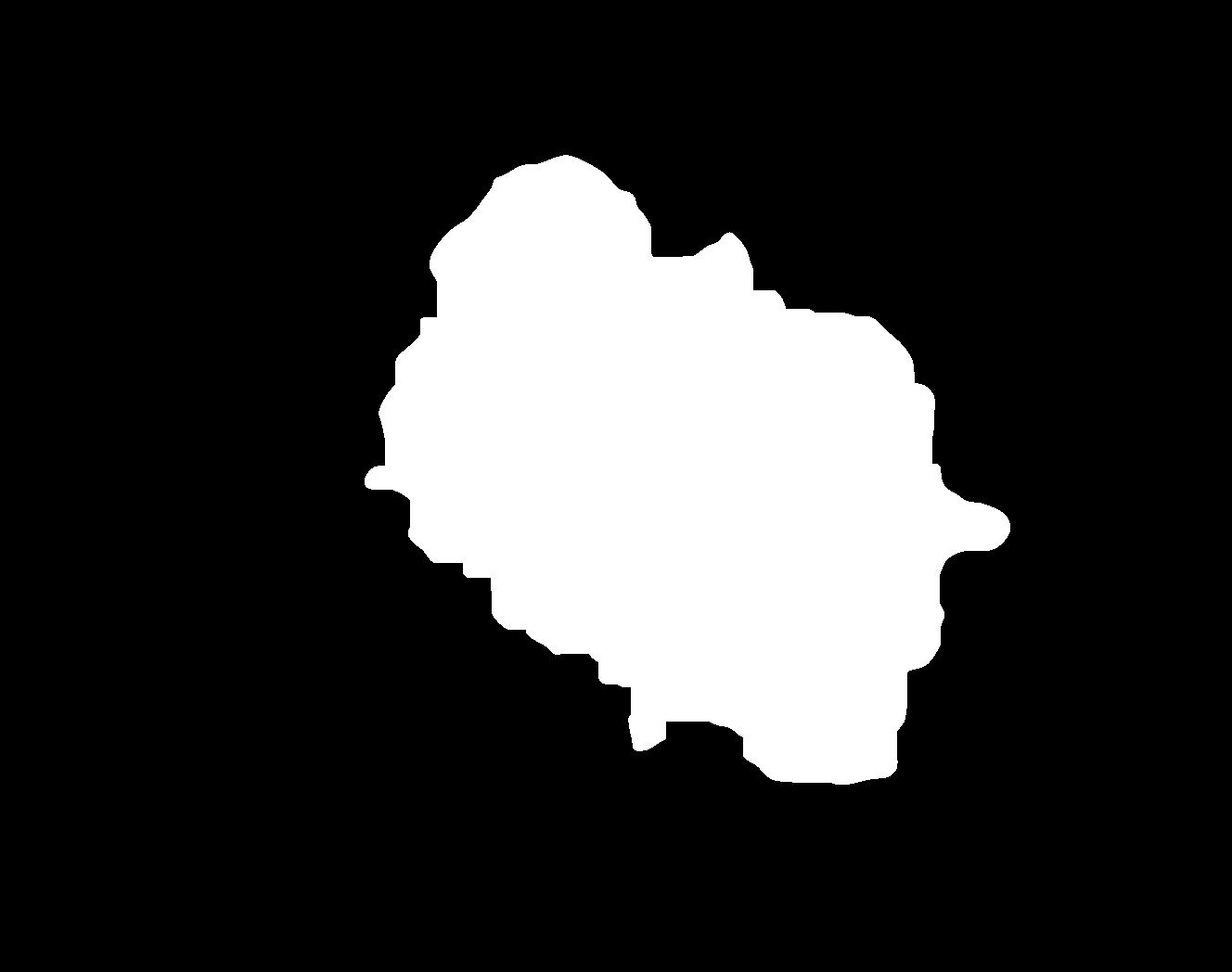

Supplement: S5 File — (ZIP) [file pone.0237972.s005.zip › S3_File IoU scores/masks/Experiment_1/cell/user_segmented/Composite/Composite_Participant13_mask_cell_I.jpg]

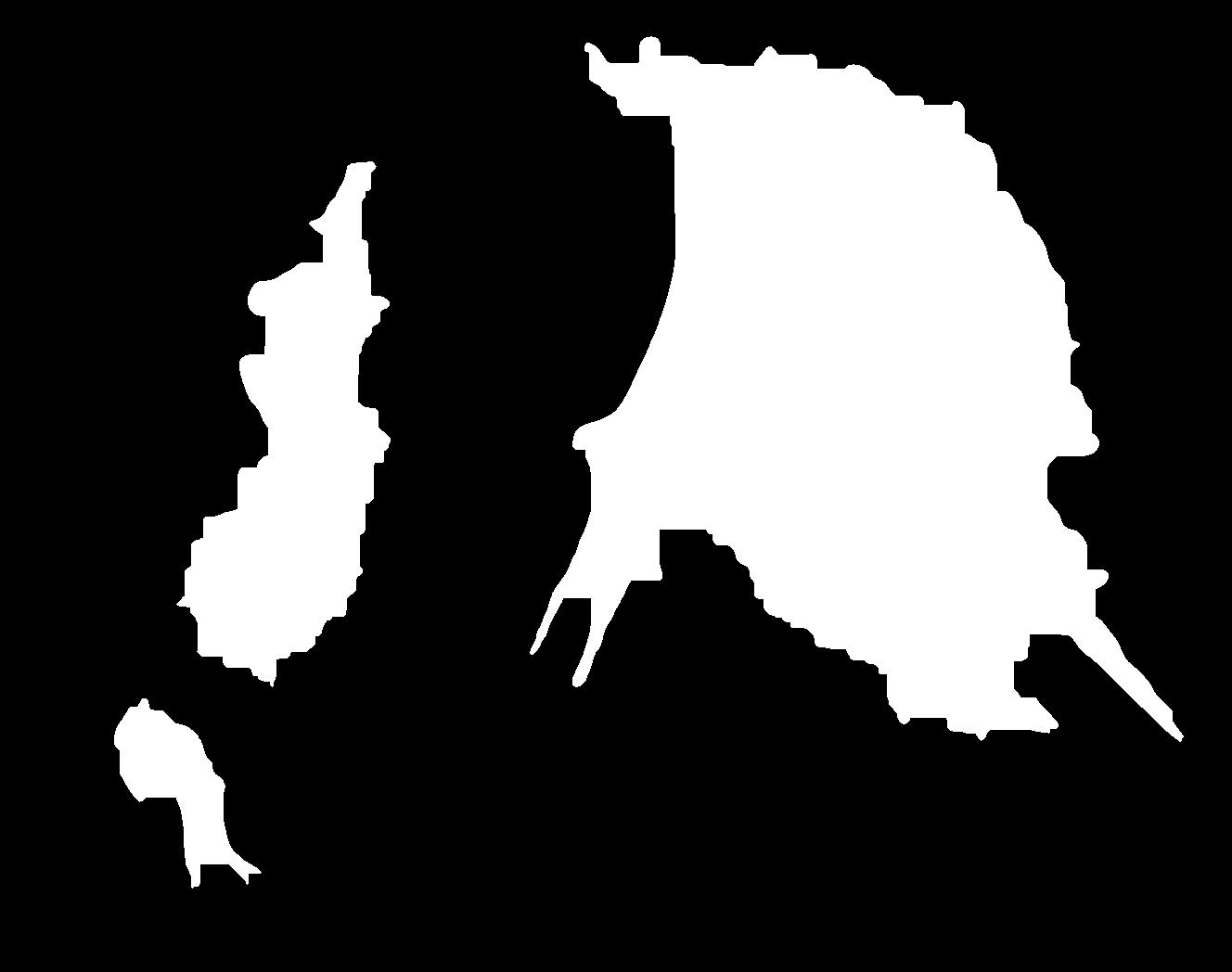

Supplement: S5 File — (ZIP) [file pone.0237972.s005.zip › S3_File IoU scores/masks/Experiment_1/cell/user_segmented/Composite/Composite_Participant13_mask_cell_J.jpg]

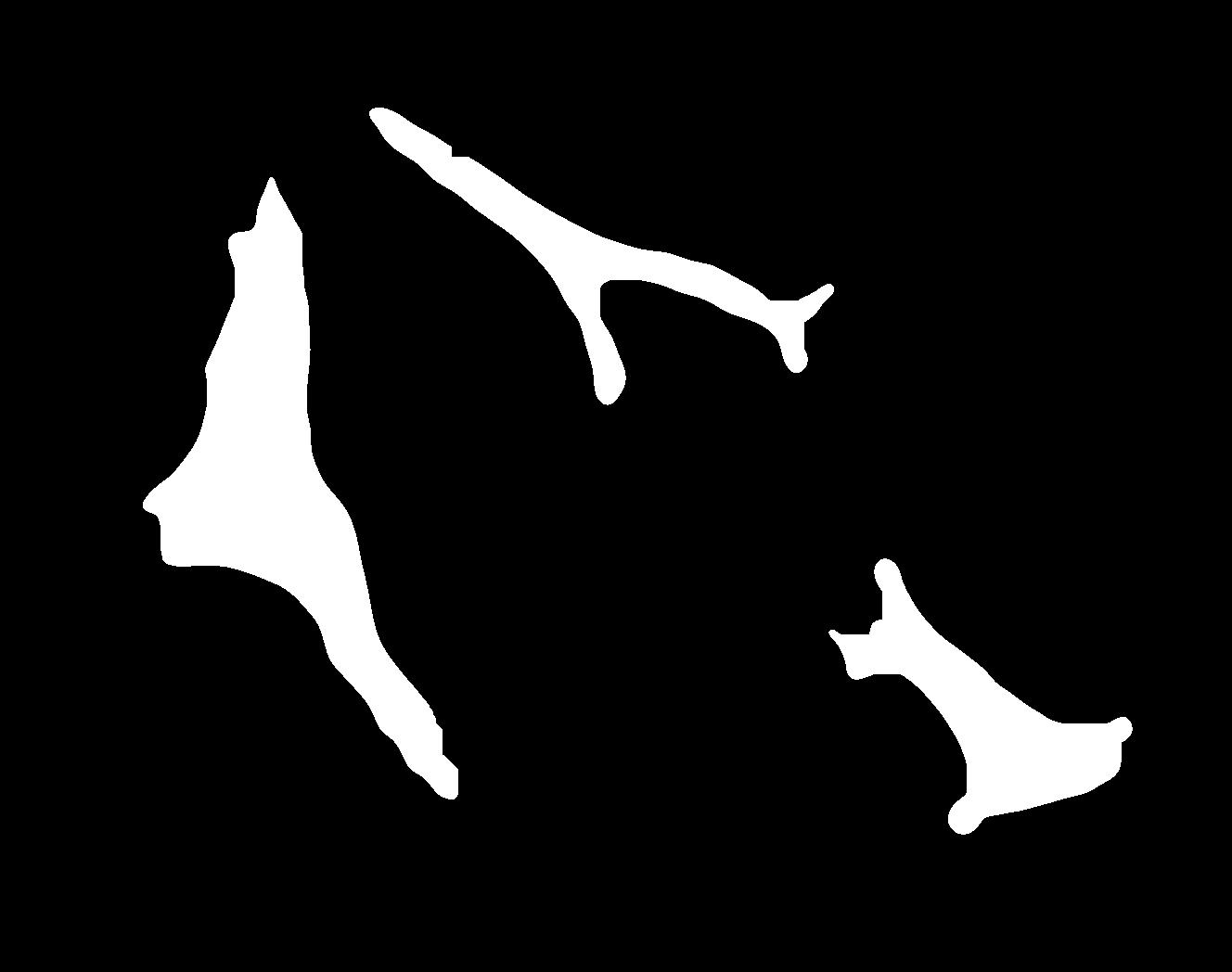

Supplement: S5 File — (ZIP) [file pone.0237972.s005.zip › S3_File IoU scores/masks/Experiment_1/cell/user_segmented/Composite/Composite_Participant15_mask_cell_A.jpg]

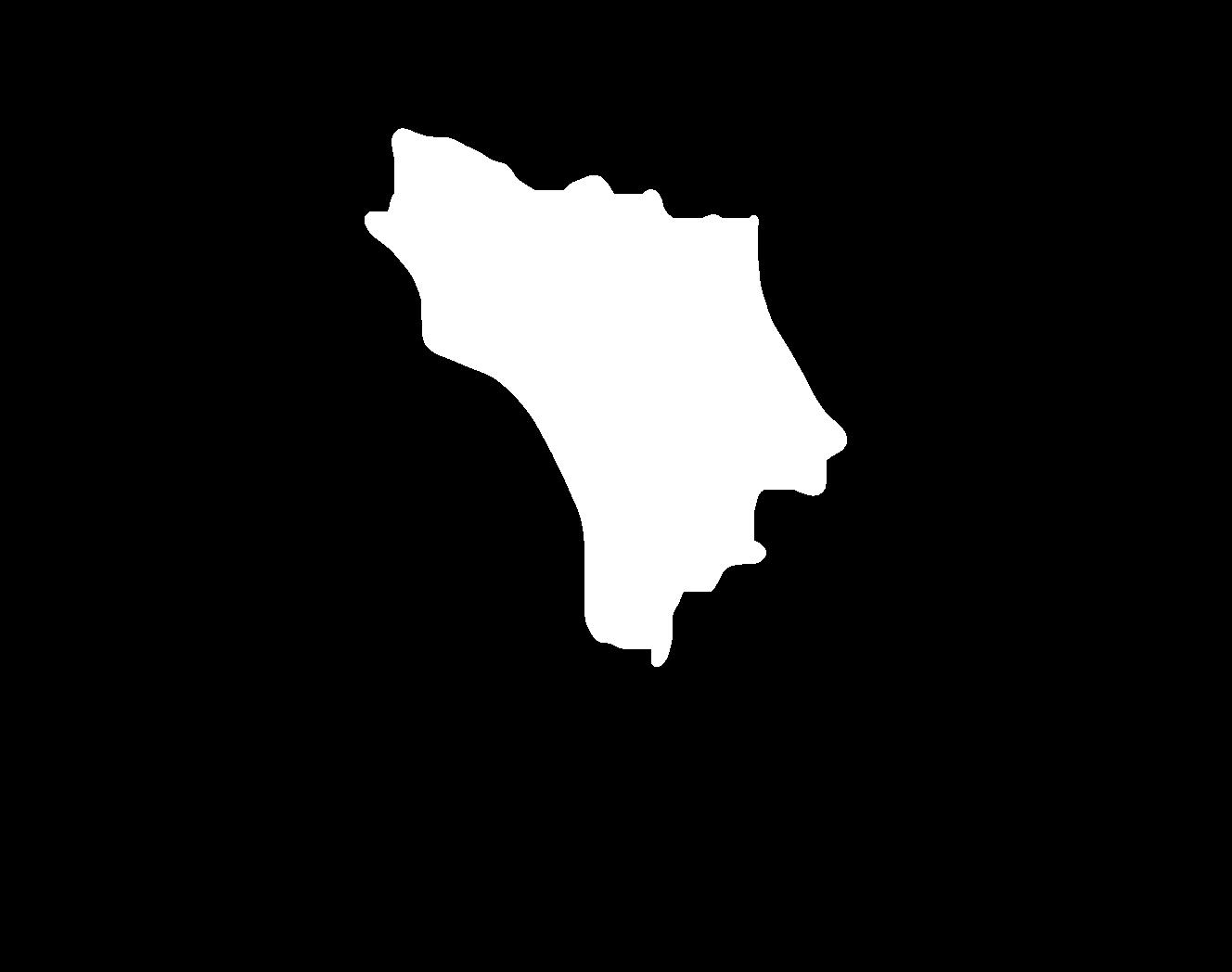

Supplement: S5 File — (ZIP) [file pone.0237972.s005.zip › S3_File IoU scores/masks/Experiment_1/cell/user_segmented/Composite/Composite_Participant15_mask_cell_B.jpg]

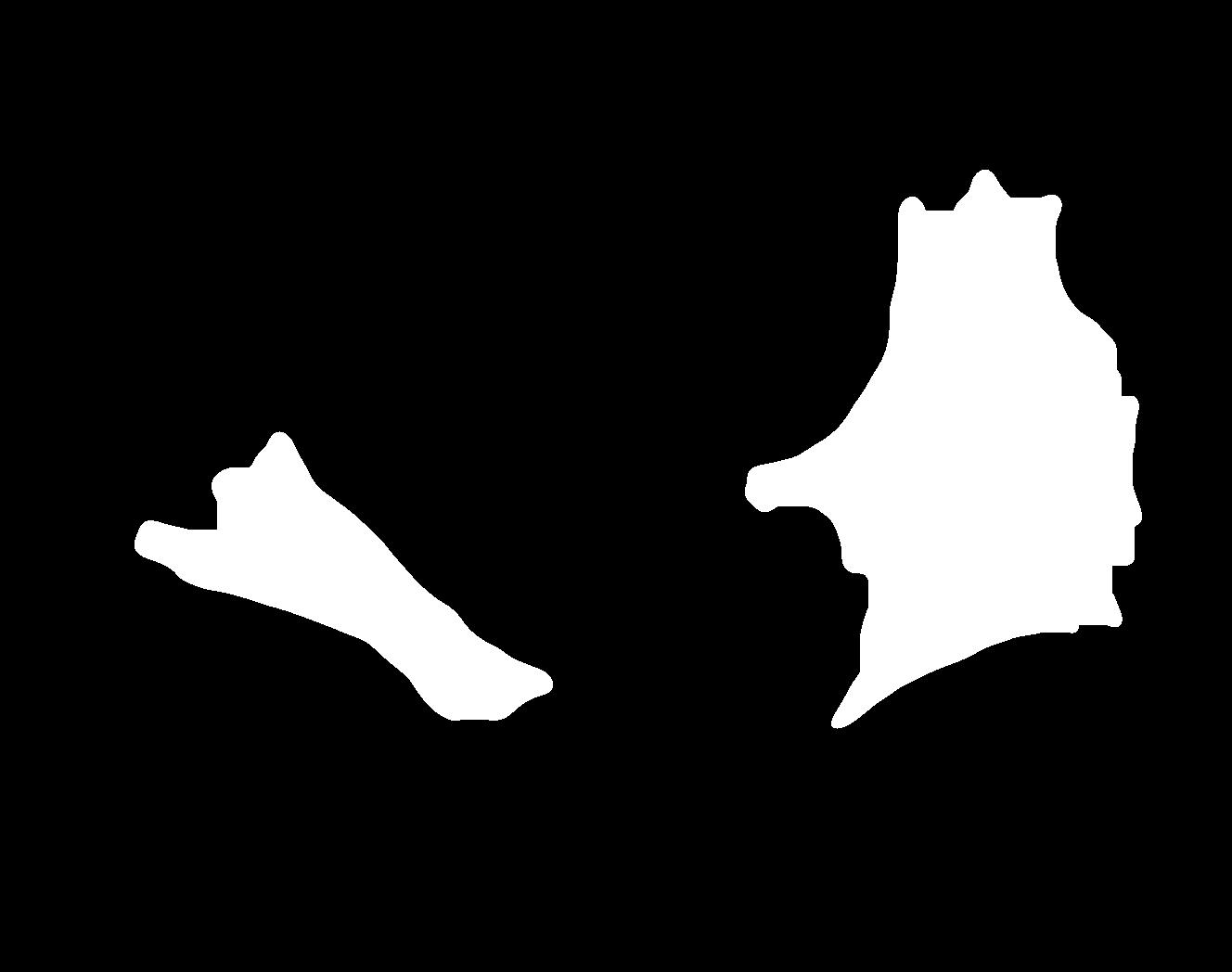

Supplement: S5 File — (ZIP) [file pone.0237972.s005.zip › S3_File IoU scores/masks/Experiment_1/cell/user_segmented/Composite/Composite_Participant15_mask_cell_C.jpg]

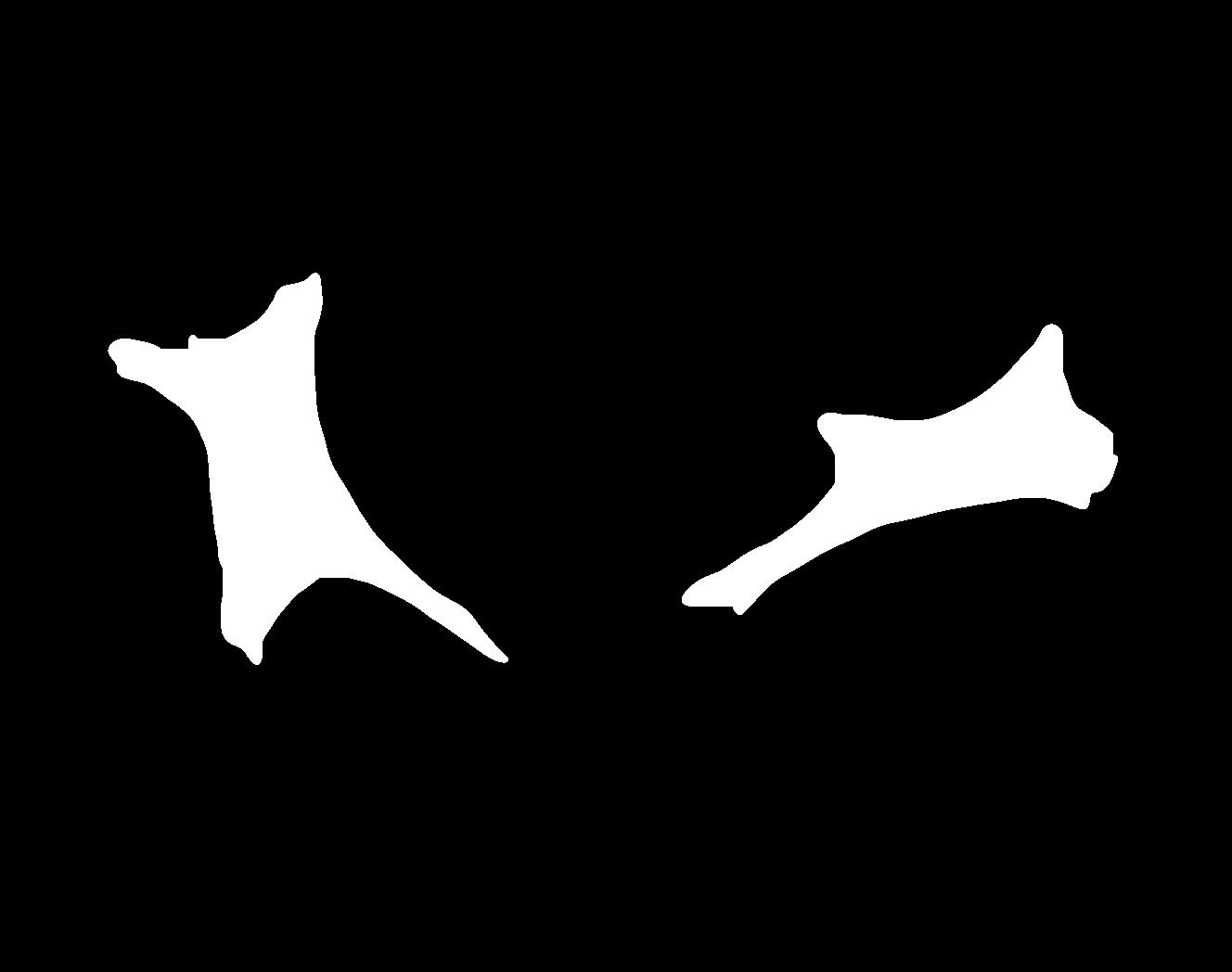

Supplement: S5 File — (ZIP) [file pone.0237972.s005.zip › S3_File IoU scores/masks/Experiment_1/cell/user_segmented/Composite/Composite_Participant15_mask_cell_D.jpg]

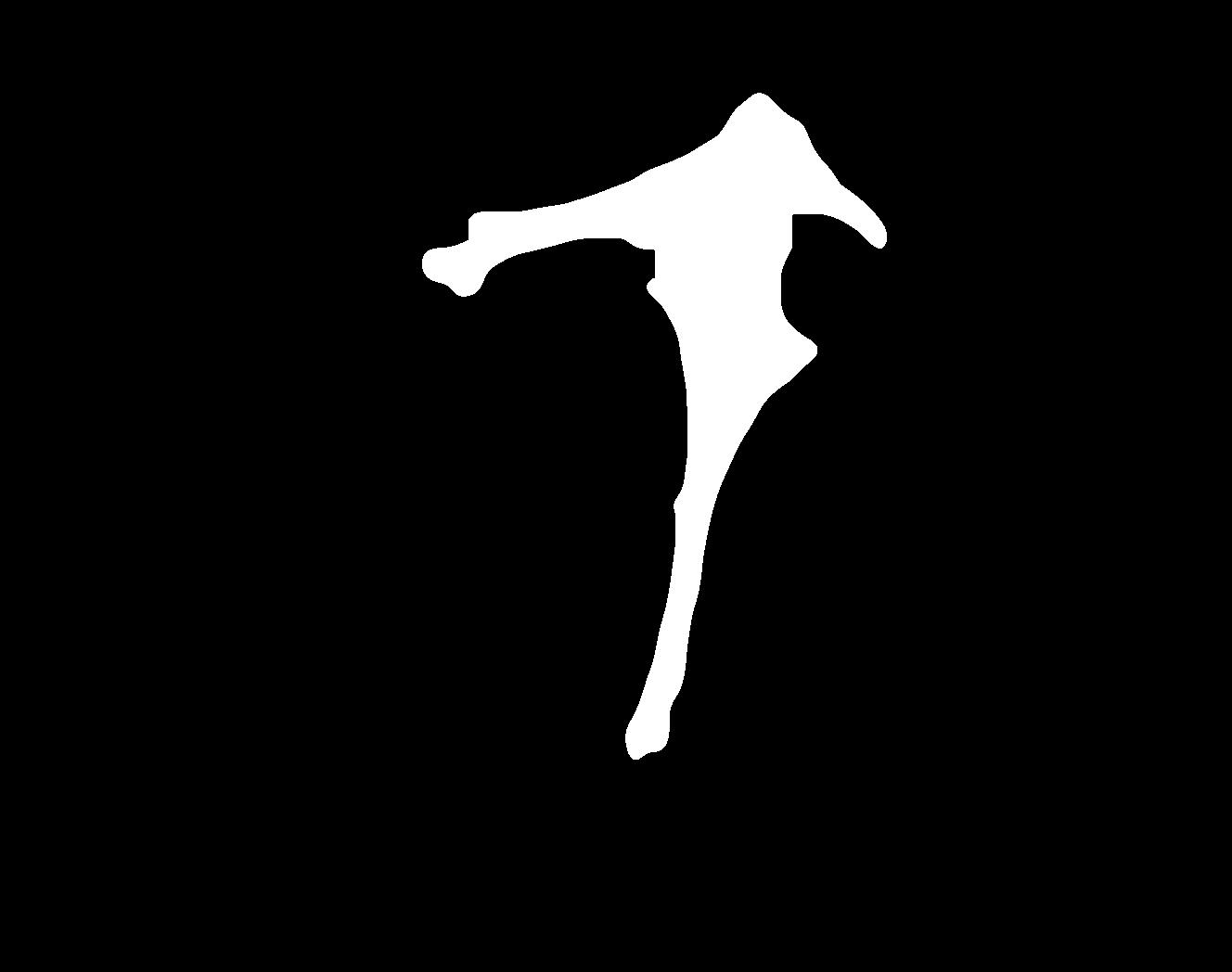

Supplement: S5 File — (ZIP) [file pone.0237972.s005.zip › S3_File IoU scores/masks/Experiment_1/cell/user_segmented/Composite/Composite_Participant15_mask_cell_E.jpg]

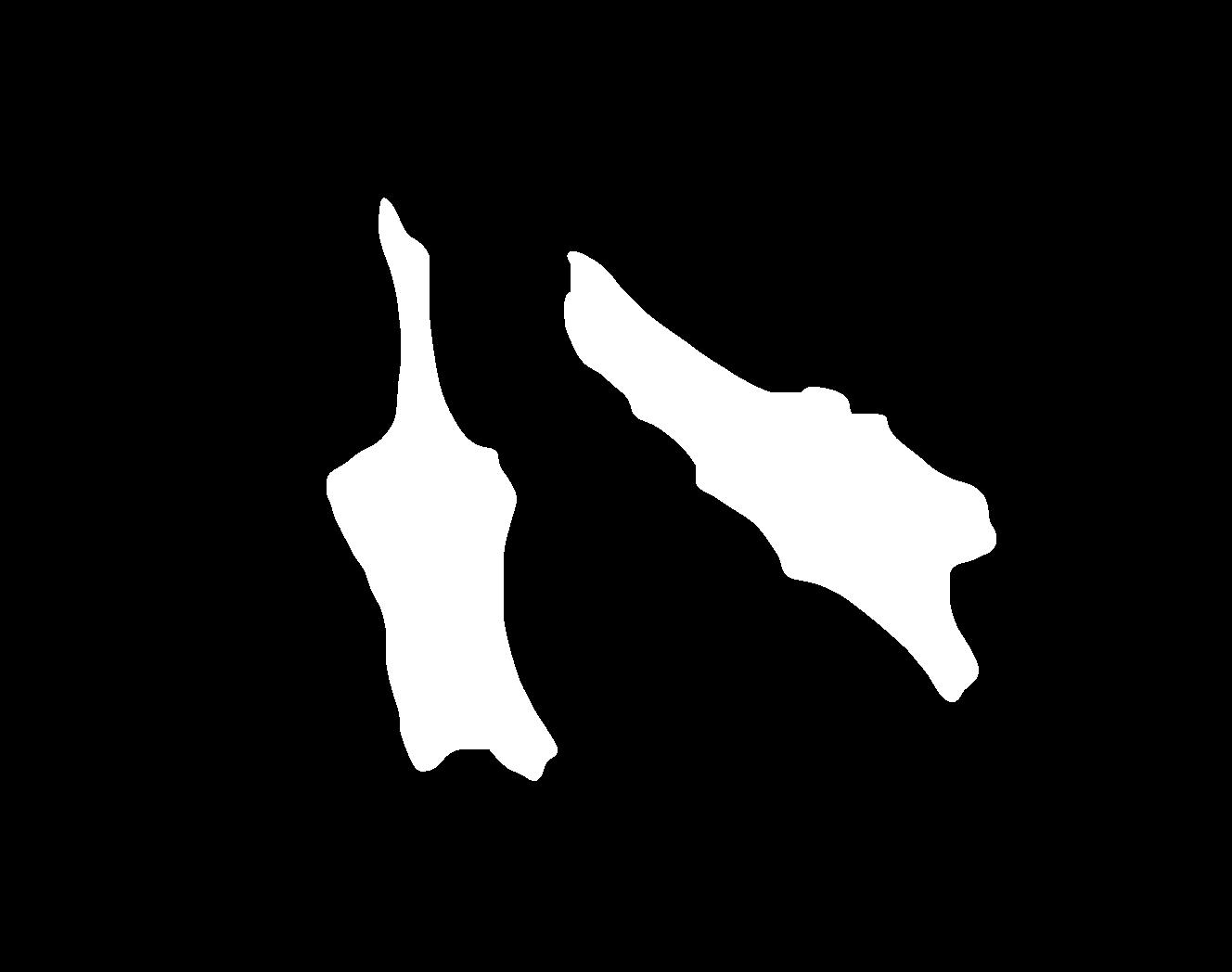

Supplement: S5 File — (ZIP) [file pone.0237972.s005.zip › S3_File IoU scores/masks/Experiment_1/cell/user_segmented/Composite/Composite_Participant15_mask_cell_F.jpg]

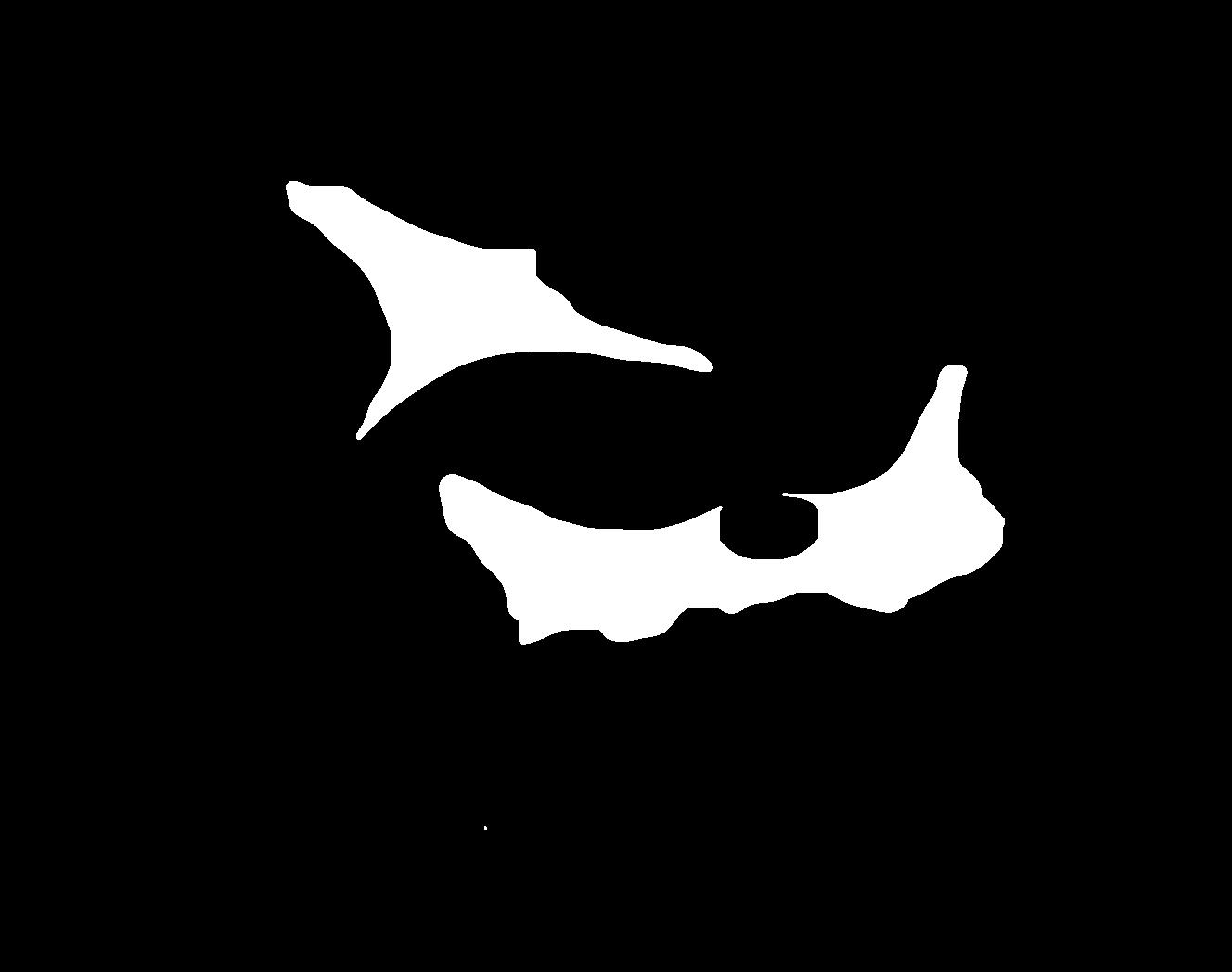

Supplement: S5 File — (ZIP) [file pone.0237972.s005.zip › S3_File IoU scores/masks/Experiment_1/cell/user_segmented/Composite/Composite_Participant15_mask_cell_G.jpg]

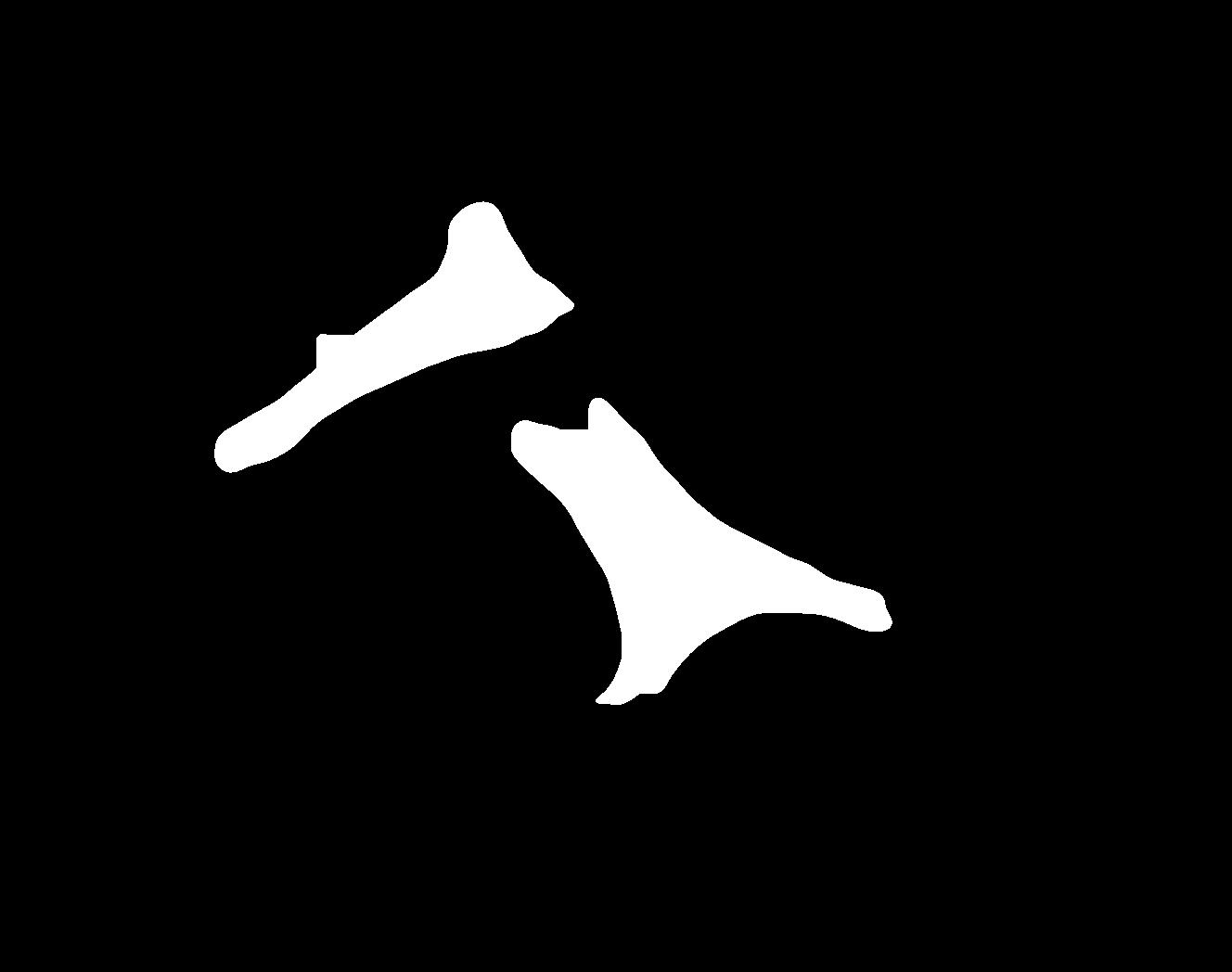

Supplement: S5 File — (ZIP) [file pone.0237972.s005.zip › S3_File IoU scores/masks/Experiment_1/cell/user_segmented/Composite/Composite_Participant15_mask_cell_H.jpg]
