# Supplementary material for: Interactive machine learning for fast and robust cell profiling
Source: PLoS One. 2020 Sep 11;15(9):e0237972. doi: 10.1371/journal.pone.0237972 (PMC7485821; doi:10.1371/journal.pone.0237972)
Supplement: S1 Method — (PDF) [file pone.0237972.s007.pdf]

S1 Methods

Image acquisition

Cell culture

Pre-osteoblast MC3T3 cells (passages 10-12, obtained directly from ATCC) were cultured using standard cell culture practice. Cells were grown in growth media comprised of α-MEM with nucleosides and L-glutamine without ascorbic acid and supplemented with 10% FBS and 1% penicillin- streptomycin [13]. Cells were seeded at a density of 4000 cells/cm^2 on injection moulded and surface-texturised polycarbonate substrates [3, 14].

Immunofluorescence staining

MC3T3 cells were cultured for 2 days before fixation using 4% paraformaldehyde. Cells were then stained with DAPI, AlexaFluor conjugated-phalloidin (ThermoFisher, 1:200) to detect the nucleus and the actin cytoskeleton, respectively. On the same cells, focal adhesions were visualised using an anti-talin1 (Abcam 71333, 1:200) and an appropriate AlexaFluor conjugated secondary antibody (ThermoFisher, 1:500). Cells were then mounted on 0.17 µm thick glass coverslips before imaging.

Fluorescence microscopy

Images of fluorescently stained cells were obtained using an EVOS FL2 Auto system (ThermoFisher) with 40X magnification (numerical aperture = 1.3). Image sets of the nucleus, the cell (visualised using the actin cytoskeleton) and focal adhesions were used to test the newly developed CellProfiler modules.

Participant recruitment

Participants were informed in writing that the purpose of the study was to evaluate the performance of our CP modules for object segmentation. Participants provided consent to participate by signing the informed consent forms. The participants were required to have a basic understanding of computer aided image analysis. Participants were offered minor monetary compensation (10 GBP), and the possibility to win a larger amount (50 GBP) in a raffle. A total of 16 participants were recruited, all of whom showed varying levels of experience in image analysis. The participant information sheet, consent form and task sheets are provided in S4 File.
