# Supplementary material for: Interactive machine learning for fast and robust cell profiling
Source: PLoS One. 2020 Sep 11;15(9):e0237972. doi: 10.1371/journal.pone.0237972 (PMC7485821; doi:10.1371/journal.pone.0237972)
Supplement: S1 Table — (PDF) [file pone.0237972.s014.pdf]

S1 **Table.** Pipeline parameters automatically optimised in cell segmentation using the interactive machine learning approach.

Object	Module	Setting	Minimum value	Maximum Value	Interval
Nucleus	Identify Primary Objects	Threshold correction factor	0.9	1.5	0.1
Cell body	Identify Secondary Objects	Size of adaptive window	50.0	350.0	25.0
		Threshold correction factor	0.9	1.5	0.05
	Smooth	Typical artifact diameter	2.0	10.0	1.0
