# Supplementary material for: Interactive machine learning for fast and robust cell profiling
Source: PLoS One. 2020 Sep 11;15(9):e0237972. doi: 10.1371/journal.pone.0237972 (PMC7485821; doi:10.1371/journal.pone.0237972)
Supplement: S2 Table — (PDF) [file pone.0237972.s015.pdf]

S2 **Table.** Pipeline parameters automatically optimised for focal adhesion segmentation using the interactive machine learning approach.

**Object**	**Module**	**Setting**	**Minimum value**	**Maximum Value**	**Interval**
Cell	Identify Secondary Objects	Size of adaptive window	50	350	50
Focal adhesions	Identify Primary Objects	Threshold correction factor	0.8	1.5	0.05
		Size of smoothing	0	20	1
		Suppress local maxima that are closer than the minimum allowed distance	0	20	1
